# Supplementary material for: MS-DIAL 5 multimodal mass spectrometry data mining unveils lipidome complexities
Source: Nat Commun. 2024 Nov 28;15:9903. doi: 10.1038/s41467-024-54137-w (PMC11605090; doi:10.1038/s41467-024-54137-w)
Supplement: Supplementary file 1 — Supplementary Information [file 41467_2024_54137_MOESM1_ESM.pdf]

Supplementary Information

MS-DIAL 5 multimodal mass spectrometry data mining unveils lipidome complexities

Authors

Hiroaki Takeda[1,$], Yuki Matsuzawa[1,$], Manami Takeuchi[1,$], Mikiko Takahashi[2], Kozo Nishida[1], Takeshi Harayama[3,4,*], Yoshimasa Todoroki[1], Kuniyoshi Shimizu[1], Nami Sakamoto[1], Takaki Oka[1], Masashi Maekawa[5], Mi Hwa Chung[1], Yuto Kurizaki[1], Saki Kiuchi[1], Kanako Tokiyoshi[1], Bujinlkham Buyantogtokh[1], Misaki Kurata[1], Aleš Kvasnička[6,7,8], Ushio Takeda[9], Haruki Uchino[5,10], Mayu Hasegawa[11], Junki Miyamoto[11], Kana Tanabe[12], Shigenori Takeda[12], Tetsuya Mori[2], Ryota Kumakubo[1], Tsuyoshi Tanaka[1], Tomoko Yoshino[1], Mami Okamoto[13], Hidenori Takahashi[13], Makoto Arita[5,10,14,*], Hiroshi Tsugawa[1,2,10,14,*]

Affiliations

1. Department of Biotechnology and Life Science, Tokyo University of Agriculture and Technology, 2-24-16 Naka-cho, Koganei-shi, Tokyo 184-8588, Japan
2. RIKEN Center for Sustainable Resource Science, 1-7-22 Suehiro-cho, Tsurumi-ku, Yokohama, Kanagawa 230-0045, Japan
3. Institut de Pharmacologie Moléculaire et Cellulaire, Université Côte d'Azur - CNRS UMR7275 - Inserm U1323, 660 Route des Lucioles, 06560 Valbonne, France
4. Institute of Global Innovation Research, Tokyo University of Agriculture and Technology, 2-24-16 Nakamachi, Koganei-shi, Tokyo 184-8588, Japan
5. Graduate School of Pharmaceutical Sciences, Keio University, Minato-ku, Tokyo, 105-8512, Japan
6. Faculty of Medicine and Dentistry, Palacký University Olomouc, Hněvotínská 3, 779 00 Olomouc, Czech Republic
7. Laboratory for Inherited Metabolic Disorders, Department of Clinical Biochemistry, University Hospital Olomouc, Zdravotníků 248/7, 779 00 Olomouc, Czech Republic
8. Department of Medical Biochemistry, Oslo University Hospital, Sognsvannsveien 20, 0372 Oslo, Norway
9. K.K. ABSciex Japan, Shinagawa Tokyo 140-0001, Japan
10. RIKEN Center for Integrative Medical Sciences, 1-7-22 Suehiro-cho, Tsurumi-ku, Yokohama, Kanagawa 230-0045, Japan
11. Department of Applied Biological Science, Tokyo University of Agriculture and Technology, 3-5-8 Saiwai-cho, Fuchu, Tokyo 183-8509, Japan
12. Innovative Technology Laboratories, AGC Inc., 1-1 Suehiro-cho, Tsurumi-ku, Yokohama 230-0045, Japan
13. Shimadzu Corporation, 1 Nishinokyo Kuwabara-cho, Nakagyo-ku, Kyoto 604-8511, Japan
14. Graduate School of Medical Life Science, Yokohama City University, Yokohama, Japan

$ Hiroaki Takeda, Yuki Matsuzawa, and Manami Takeuchi contributed equally to this work.

Corresponding Authors

*E-mail: T.H. (harayama@ipmc.cnrs.fr), M.A. (marita@keio.jp) and Hiroshi T. (htsugawa@go.tuat.ac.jp)

Contents

Supplementary Figures 1-12
Supplementary Notes 1-6

Supplementary Figure legends

Supplementary Figure 1. Results of molecular spectrum networking of 716 metabolites based on the spectra from collision-induced dissociation (CID) and electron-activated dissociation (EAD). (a) Summary of nodes connectivity. If two nodes have the same ontology term, the count is incremented. The ontology terms were generated by the ClassyFire program. The term "parent" means the direct parent term of metabolite defined by the ClassyFire program. (b) Molecular networks based on the spectra of CID 40±15 V (left panel) and kinetic energy (KE) 15 eV with CID 10 V.

Supplementary Figure 2. Relationships between the kinetic energy 14 eV spectrum and the lipid structure in the glycerophospholipids category. The top and bottom panels show the experimental- and computer-generated spectrum in MS-DIAL, respectively. NL means neutral loss. The spectra of cardiolipin (CL) and hemi-BMP are also described to clarify the inadequate information in the product ion spectra for the determination of *sn*- and C=C-positions.

Supplementary Figure 3. EAD-MS/MS with kinetic energy at 10 eV, 14 eV, and 18 eV for phospholipids containing polyunsaturated fatty acids with more than three double bonds. EAD-MS/MS spectra of PC 18:3(9,12,15)/18:3(9,12,15), PC 22:6(4,7,10,13,16,19)/22:6(4,7,10,13,16,19), PC O-16:0/20:4(5,8,11,14), PE 18:0/20:4(5,8,11,14), PE 22:6(4,7,10,13,16,19)/22:6(4,7,10,13,16,19), and PG 22:6(4,7,10,13,16,19)/22:6(4,7,10,13,16,19) are shown with the same description method as described in **Figure 1d**.

Supplementary Figure 4. Relationships between the kinetic energy 14 eV spectrum and the lipid structure in the glycerolipids and fatty acyls categories. The layout and the terms used are the same as those in Supplementary Figure 2. For free fatty acid (FA) and FAHFA, the spectra of the derived forms using 2-dimethylaminoethylamine (DMED) are described.

Supplementary Figure 5. Relationships between the kinetic energy 14 eV spectrum and the lipid structure in the sphingolipids category. The layout and the terms used are the same as those in Supplementary Figure 2. For the ceramide-AS type containing alpha-hydroxy fatty acid as the *N*-acyl chain, the OH position in the *N*-acyl chain was not characterized, while the OH positions in the sphingobase moiety were characterized.

Supplementary Figure 6. Details of misannotations in the molecules of phosphatidylcholine (PC), phosphatidylinositol (PI), and triacylglycerol (TG). (a) The MS/MS spectrum of the protonated form of PC-d5 17:0/16:1(9). The spectra of the entire (bottom panel) and zoomed regions (top panel) are shown, where the diagnostic ions of *m/z* 466.3246 and *m/z* 482.3559 that determine the *sn*-position for *sn1*-17:0 and *sn1*-16:1, respectively, are described. The annotation was incorrect due to a lower abundance of the ion related to sn1-17:0 than that of sn1-16:1. (b) The MS/MS spectrum of the protonated form of PC-d5 17:0/ 22:4(7,10,13,16) was correctly annotated, although the contamination of sn1-22:4 related ion existed.

The spectra of the entire (bottom panel) and zoomed regions (top panel) are shown, where the diagnostic ions of *m/z* 544.3715 and *m/z* 482.3559 that determine the *sn*-position for *sn1*-17:0 and *sn1*-22:4, respectively, are described. (c) MS/MS spectrum of the ammonium adduct form of PI 18:1(9)/18:1(9). The upper and lower panels show the experimental- and *in silico* MS/MS spectra. The V-shape pattern of the product ion spectrum determining the C=C-position as C9 is described. (d) MS/MS spectrum of the ammonium adduct form of PI-d5 17:0/16:1(9). The correct annotation is PI-d5 17:0/16:1(9), while the MS-DIAL program annotated the spectrum as PI-d5 17:0/16:1(7) due to the absence of a C=C high peak for 16:1(9). (e) MS/MS spectrum of the ammonium adduct form of TG 18:1(9)_18:1(9)_18:1(9), where the V-shape pattern for 18:1(9) is also described. (f) MS/MS spectrum of the ammonium adduct form of TG-d5 16:0_16:0_17:1(10). The spectrum was misannotated because the local correlation value for 17:1(5) was higher than that of 17:1(9). The V-shape patterns for 17:1(5) and 17:1(9) are described while the in silico MS/MS spectrum of TG-d5 16:0_16:0_17:1(10) is described in the lower panel.

Supplementary Figure 7. Data set construction for MS-DIAL evaluation using lipid isomers, PC 16:0/18:1(9), PC 16:0/18:1(11), and PC 18:1(9)/16:0. (a) Extracted ion chromatograms (EICs) of PC 16:0/18:1(9Z), PC 16:0/18:1(11Z), and PC 18:1(9Z)/16:0, abbreviated as POPC, PVPC, and OPPC, respectively, in the LC-MS method used in this study. (b) MS/MS patterns of PC 16:0/18:1(9), PC 18:1(9)/16:0, and PC 16:0/18:1(11). (c) Calibration curves for PC 16:0/18:1(9), PC 18:1(9)/16:0, and PC 16:0/18:1(11). The calibration curves were constructed for each lipid isomer using MS1 peak height and normalized MS1 peak height by the internal standard of PC 15:0_18:1(d7). The calibration curves were also constructed using MS2 peak height of *sn*- and C=C position diagnostic ions. (d) Relationship between concentration ratio and MS2 peak height ratio in PC mixture. The mixture of POPC and PVPC and the mixture of POPC and OPPC were prepared. The calibration curves were constructed by the MS2 peak height ratios of H-loss diagnostic fragment ions and *sn1*-CH2 loss diagnostic fragment ions which are used to distinguish the C=C- and *sn*-positions, respectively. The calibration curve using the OAD2 fragment ions generated by OAD-MS/MS technique was also constructed. The definition of OAD2 is described in **Supplementary Figure 7f**. (e) Isomer ratio estimations for *Δ9*/*Δ*11 and *sn1*-16:0/*sn1*-18:1 in mouse brain and human plasma using EAD and OAD. The MS2 peak heights of MS/MS chromatograms were used to calculate the intensity ratios. The intensity ratio was then normalized by the calibration curve created in **Supplementary Figure 7d**. (f) OAD-MS/MS fragmentation patterns of PC 16:0/18:1(9) and PC 16:0/18:1(11). The fragment ions of *m/z* 622.4442 and *m/z* 650.4755 were defined as "OAD15" which is the specific fragment ion to determine the C=C position of PC 16:0/18:1(9) and PC 16:0/18:1(11), respectively. The fragment ions of *m/z* 664.4548 and *m/z* 692.4861 were defined as "OAD2" which is also the specific fragment ion to determine the C=C position of PC 16:0/18:1(9) and PC 16:0/18:1(11), respectively. The terminology of OAD2 and OAD15 follows the definition of the original publication using OAD-MS/MS technique for lipid profiling. (g) Calibration curves using MS2 peak height of C=C position diagnostic ions based on OAD-MS/MS for PC 16:0/18:1(9). (h) OAD-MS/MS in the mixture of PC isomers, plasma lipid extract, and brain lipid extract.

Supplementary Figure 8. Annotation results in the co-elution situation of two lipids having the same *m/z* value. The left panel shows the result of "DLPC-fixed," where PAPC concentrations varied at 0.1, 0.2, 0.5, 1.0, 2.0, 5.0, and 10 µM. The right panel shows the result of "PAPC" fixed where DLPC concentrations adjusted to 0.1, 0.2, 0.5, 1.0, 2.0, 5.0, and 10 µM. The term "Full description" means that both sn- and C=C-positions were characterized by the MS-DIAL program. The black and gray colors indicate the mis-annotation of not providing DLPC or PAPC.

Supplementary Figure 9. Fragment annotations for the top 5 abundant phosphatidylcholine (PC) molecules containing polyunsaturated fatty acid (PUFA) with more than three double bonds. One of the five molecules is described in **Figure 4**. EAD-MS/MS spectra for PC 16:0/22:6(4,7,10,13,16,19), PC 18:0/22:6(4,7,10,13,16,19), PC 16:0/20:4(5,8,11,14), and PC 34:5(19,22,25,28,31)/22:6(4,7,10,13,16,19) are described following the annotation in Figure 4.

Supplementary Figure 10. EAD-MS/MS spectra for VLC-PUFA PC in HeLa cells with an abundance of sn1-VLC-PUFA higher than that of sn1-16:0 or sn1-18:1. (a) EAD-MS/MS spectrum annotated as PC 32:6/18:1 by MS-DIAL 5 as the major product. (b) EAD-MS/MS spectrum annotated as PC 32:6/16:0 by MS-DIAL 5 as the major product.

Supplementary Figure 11. Expression levels of GPAT genes. The GPAT expression levels were downloaded from http://biogps.org/?full#goto=welcome on January 21, 2024. When multiple data sets were available for the expression tables, the data set with the highest expression level for each GPAT enzyme was selected.

Supplementary Figure 12. Coomassie brilliant blue (CBB) staining for recombinant proteins.

Supplementary Note legends

Note 1. Lipidomics minimal reporting checklist for MS-DIAL EAD spectral annotation.

Note 2. Lipidomics minimal reporting checklist for characterization of very long chain PUFA (VLC-PUFA) containing PC in the eye tissue of mice using EAD.

Note 3. Lipidomics minimal reporting checklist for HeLa lipid profiling for HeLa cells with the supplementation of VLC-PUFA (FA 32:6).

Note 4. Lipidomics minimal reporting checklist for lysophospholipid profiling by using trimethylsilyl-diazomethane for HeLa cells.

Note 5. Lipidomics minimal reporting checklist for lysophospholipid profiling by using trimethylsilyl-diazomethane for GPAT enzyme assay.

Note 6. Lipidomics minimal reporting checklist for acyl-CoA profiling for GPAT enzyme assay.

Supplementary Figure 1. Results of molecular spectrum networking of 716 metabolites based on the spectra from collision-induced dissociation (CID) and electron-activated dissociation (EAD). (a) Summary of nodes connectivity. If two nodes have the same ontology term, the count is incremented. The ontology terms were generated by the ClassyFire program. The term "parent" means the direct parent term of metabolite defined by the ClassyFire program. (b) Molecular networks based on the spectra of CID 40±15 V (left panel) and kinetic energy (KE) 15 eV with CID 10 V.

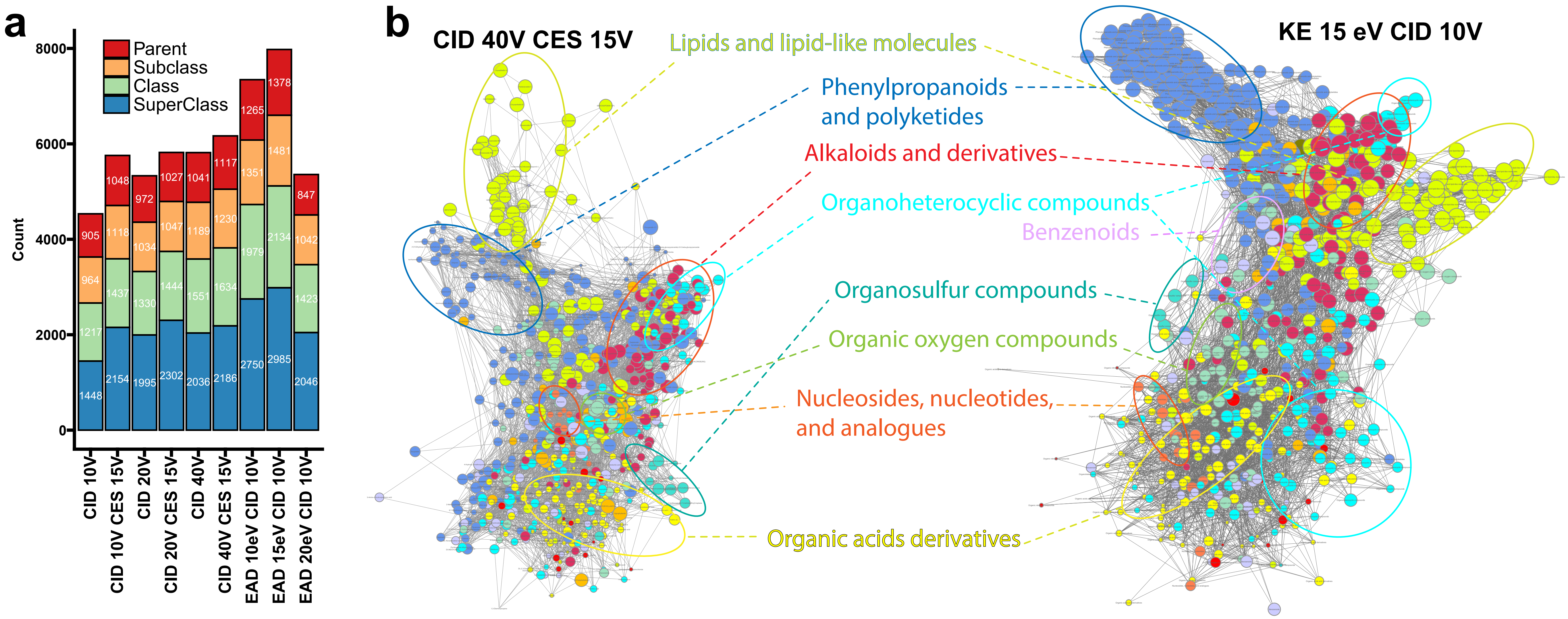
a
Count
Parent
Subclass
Class
SuperClass
8000
6000
4000
2000
0
905
964
1217
1448
1048
1118
1437
2154
972
1034
1330
1995
1027
1047
1444
2302
1041
1189
1551
2036
1117
1230
1634
2186
1265
1351
1979
2750
1378
1481
2134
2985
847
1042
1423
2046
CID 10V
CID 10V CES 15V
CID 20V
CID 20V CES 15V
CID 40V
CID 40V CES 15V
EAD 10eV CID 10V
EAD 15eV CID 10V
EAD 20eV CID 10V
b
CID 40V CES 15V
KE 15 eV CID 10V
Lipids and lipid-like molecules
Phenylpropanoids and polyketides
Alkaloids and derivatives
Organoheterocyclic compounds
Benzenoids
Organosulfur compounds
Organic oxygen compounds
Nucleosides, nucleotides, and analogues
Organic acids derivatives

Supplementary Figure 2. Relationships between the kinetic energy 14 eV spectrum and the lipid structure in the glycerophospholipids category. The top and bottom panels show the experimental- and computer-generated spectrum in MS-DIAL, respectively. NL means neutral loss. The spectra of cardiolipin (CL) and hemi-BMP are also described to clarify the inadequate information in the product ion spectra for the determination of *sn*- and C=C-positions.

PC 18:1(9)/18:1(9) as [M+H]+

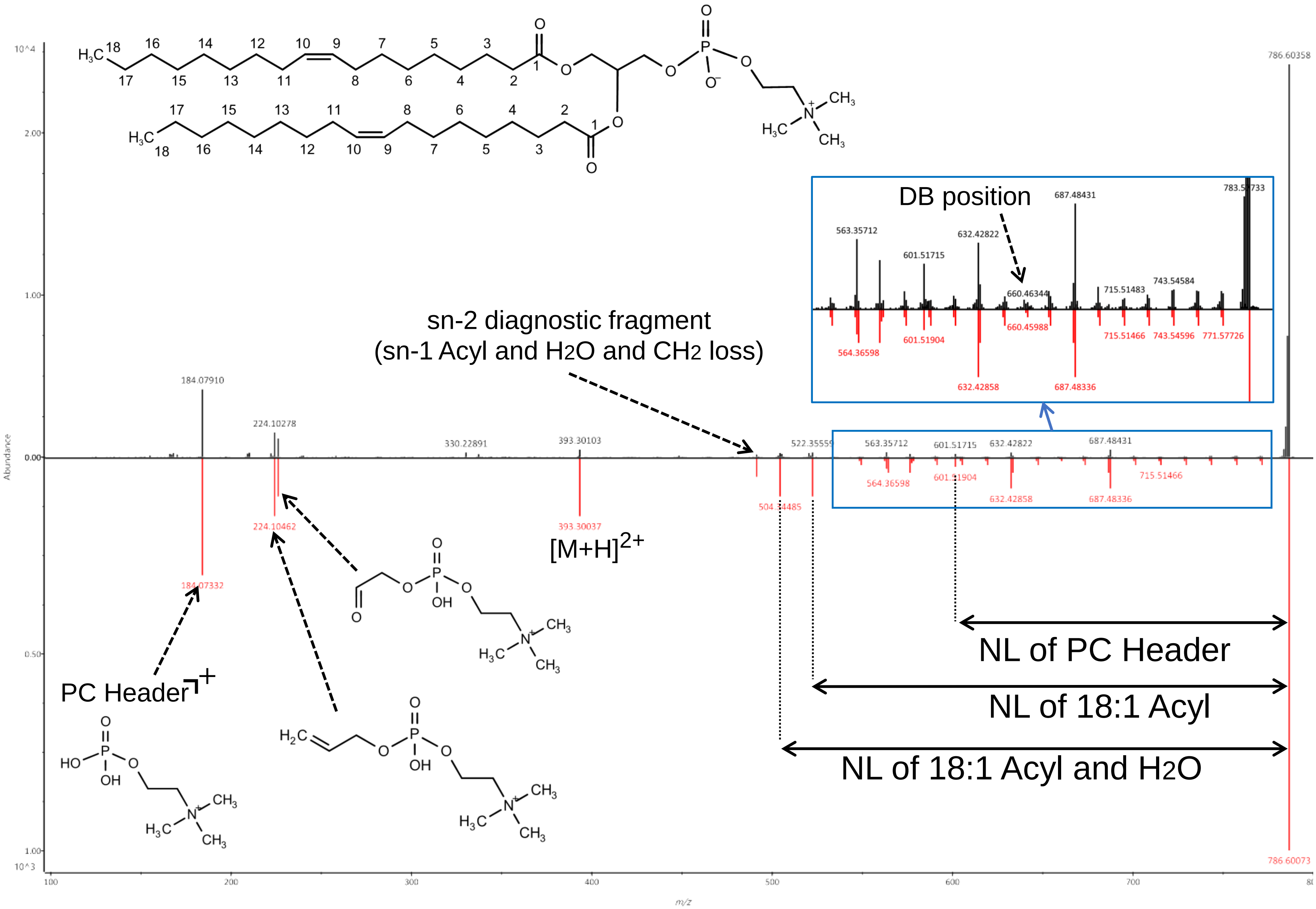

PC 18:1(9)/18:1(9) as [M+Na]+

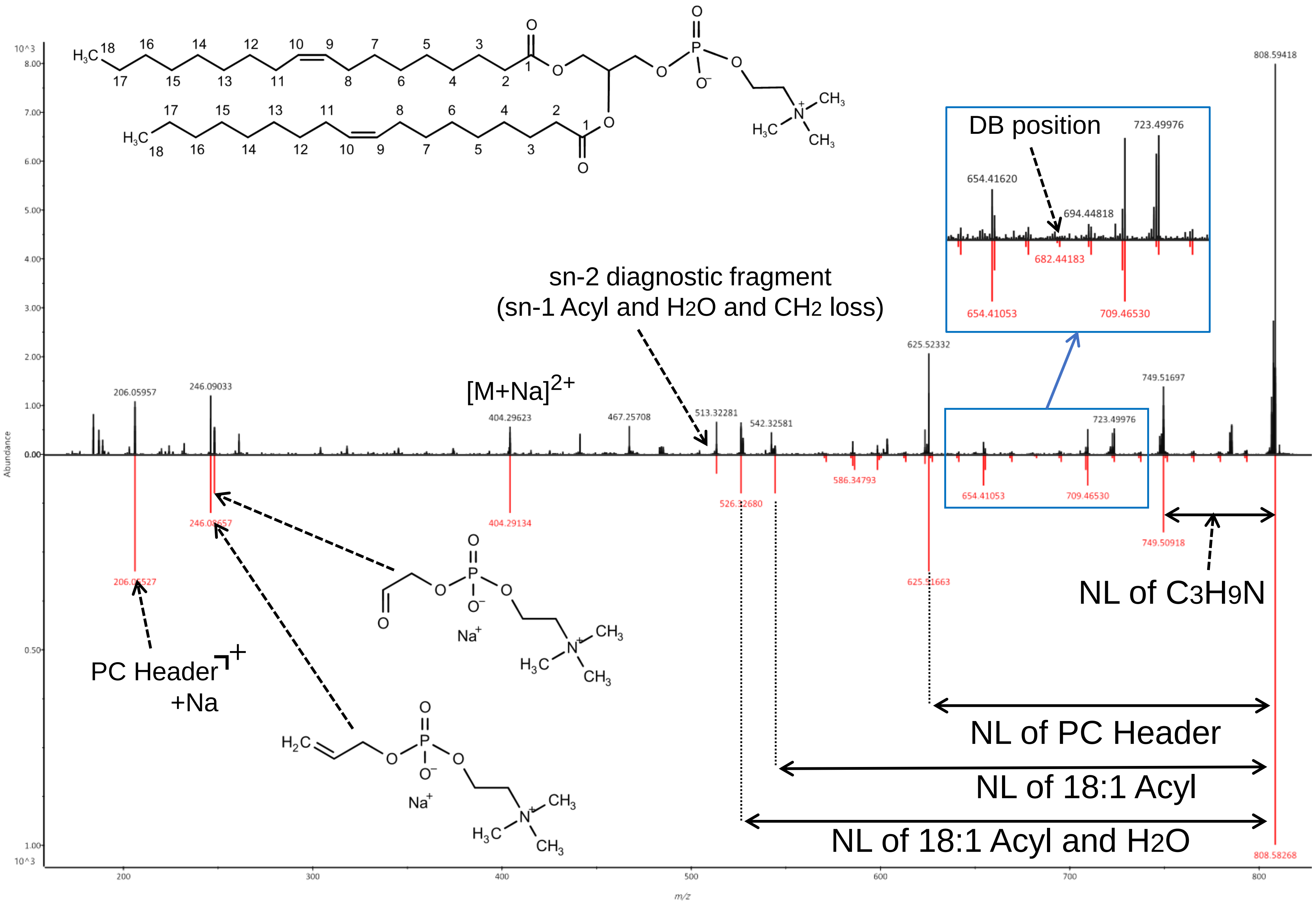

PE 18:1(9)_18:1(9) as [M+H]+

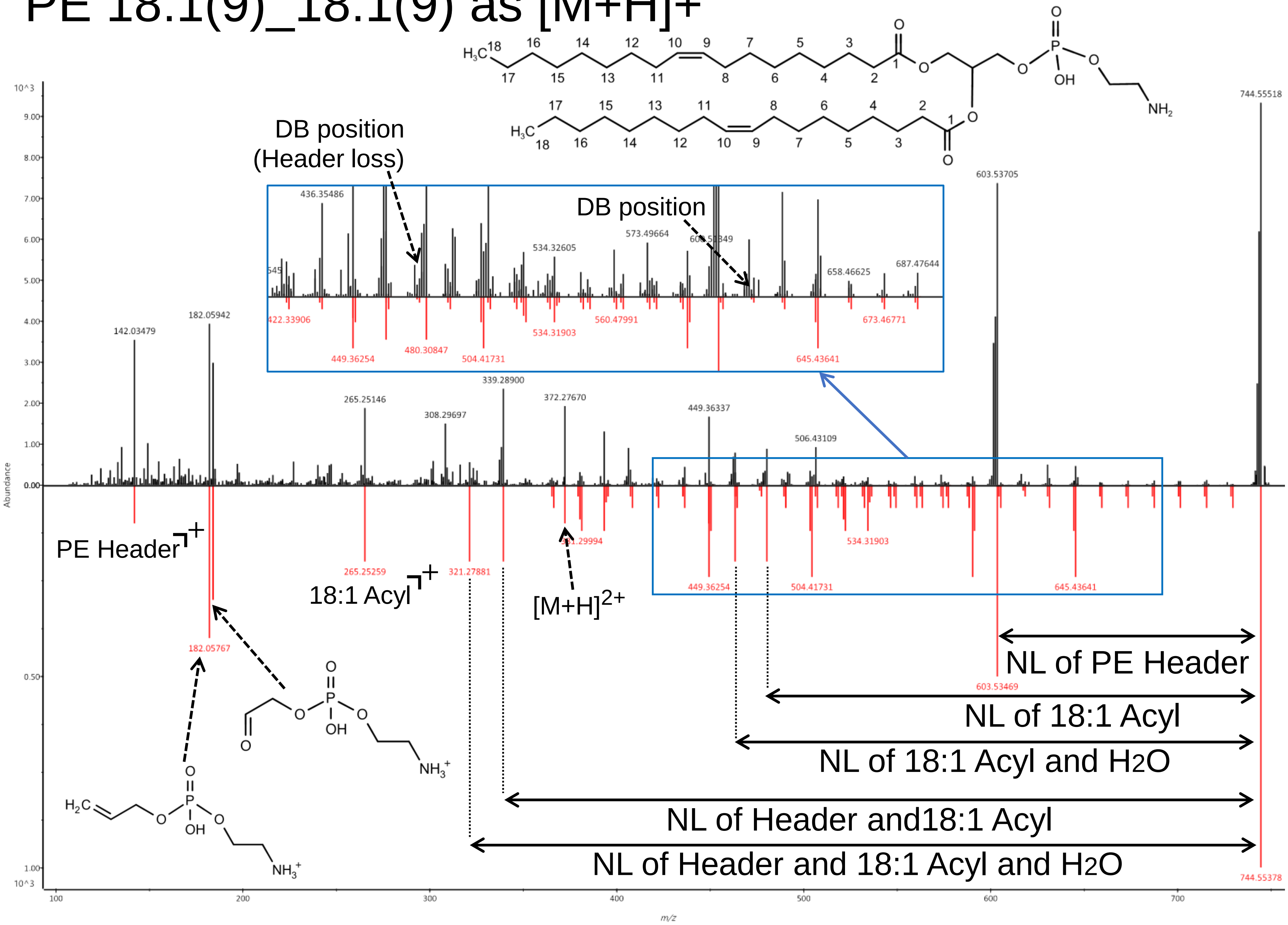

PE 18:1(9)/18:1(9) as [M+Na]+

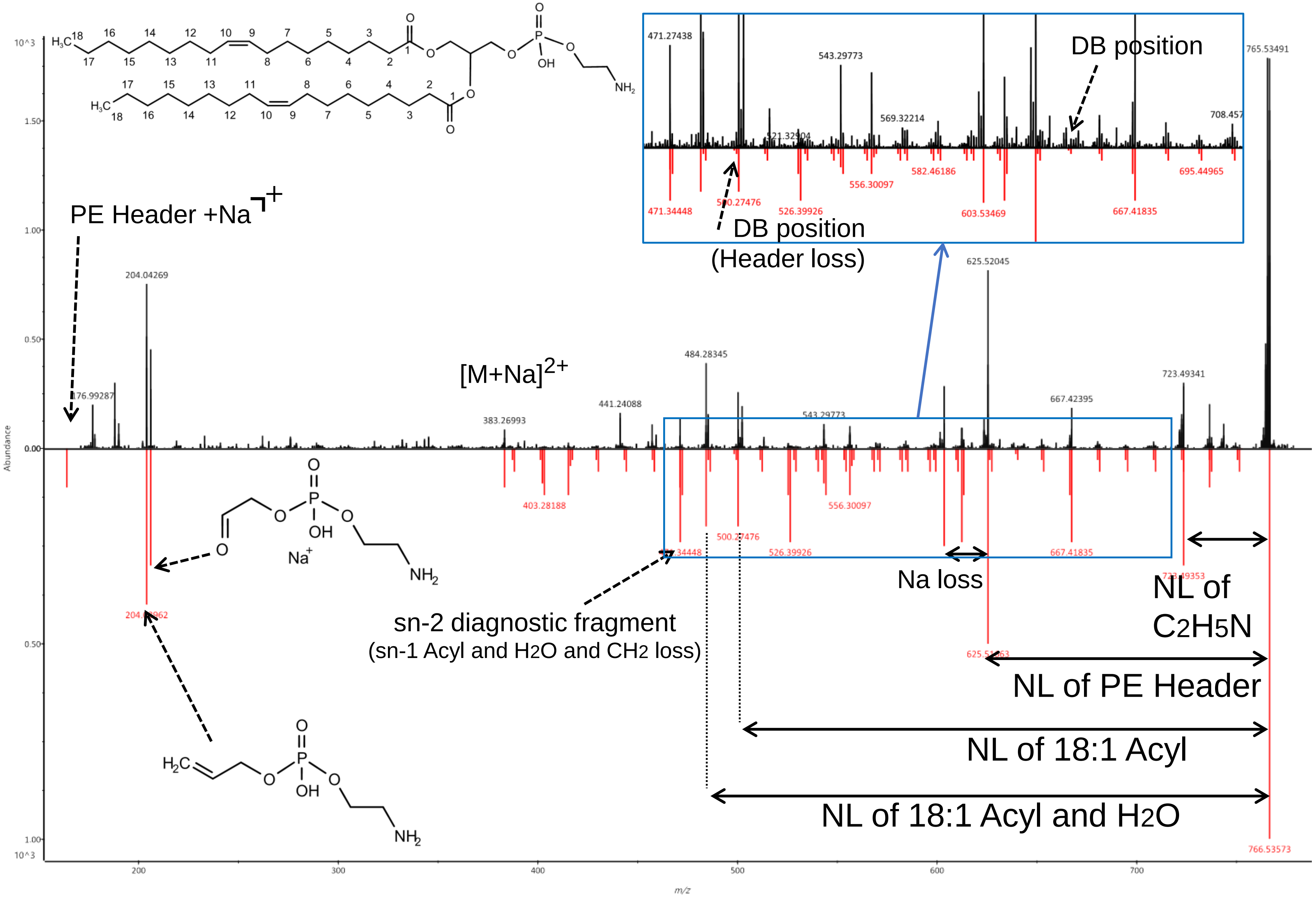

PG 18:1(9)_18:1(9) as [M+NH4]+

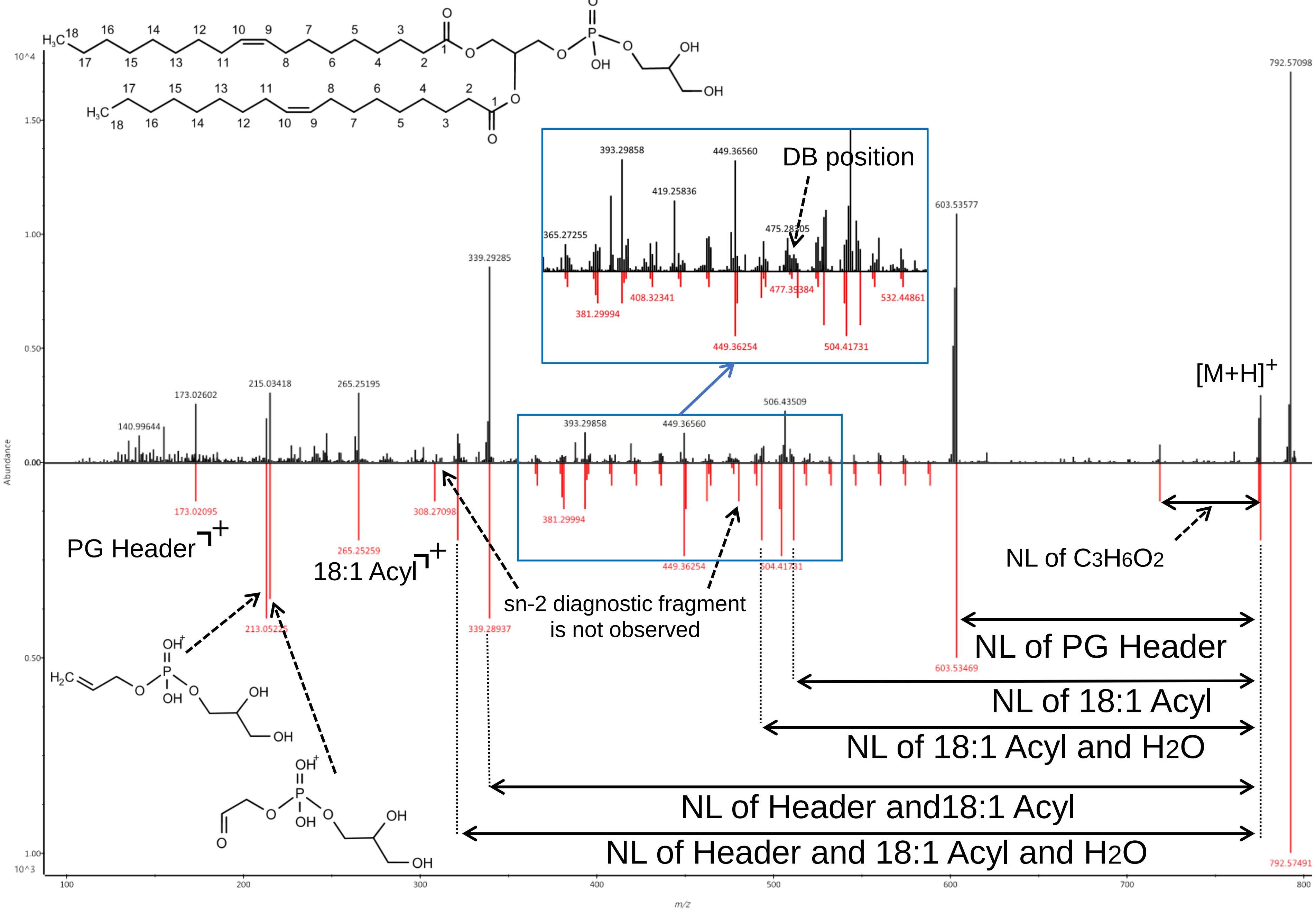

PG 18:1(9)/18:1(9) as [M+Na]+

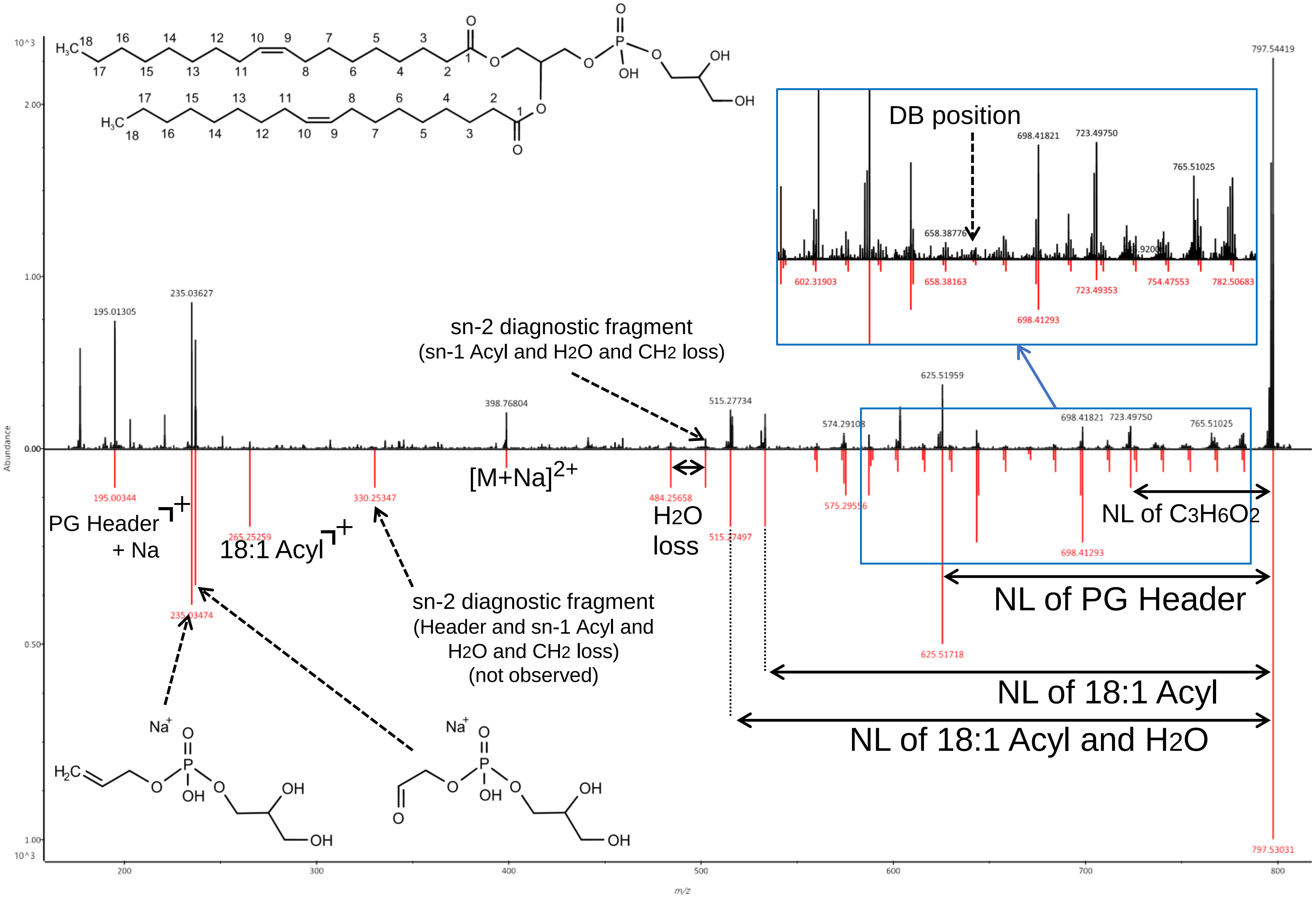

PI 18:1(9)_18:1(9) as [M+NH4]+

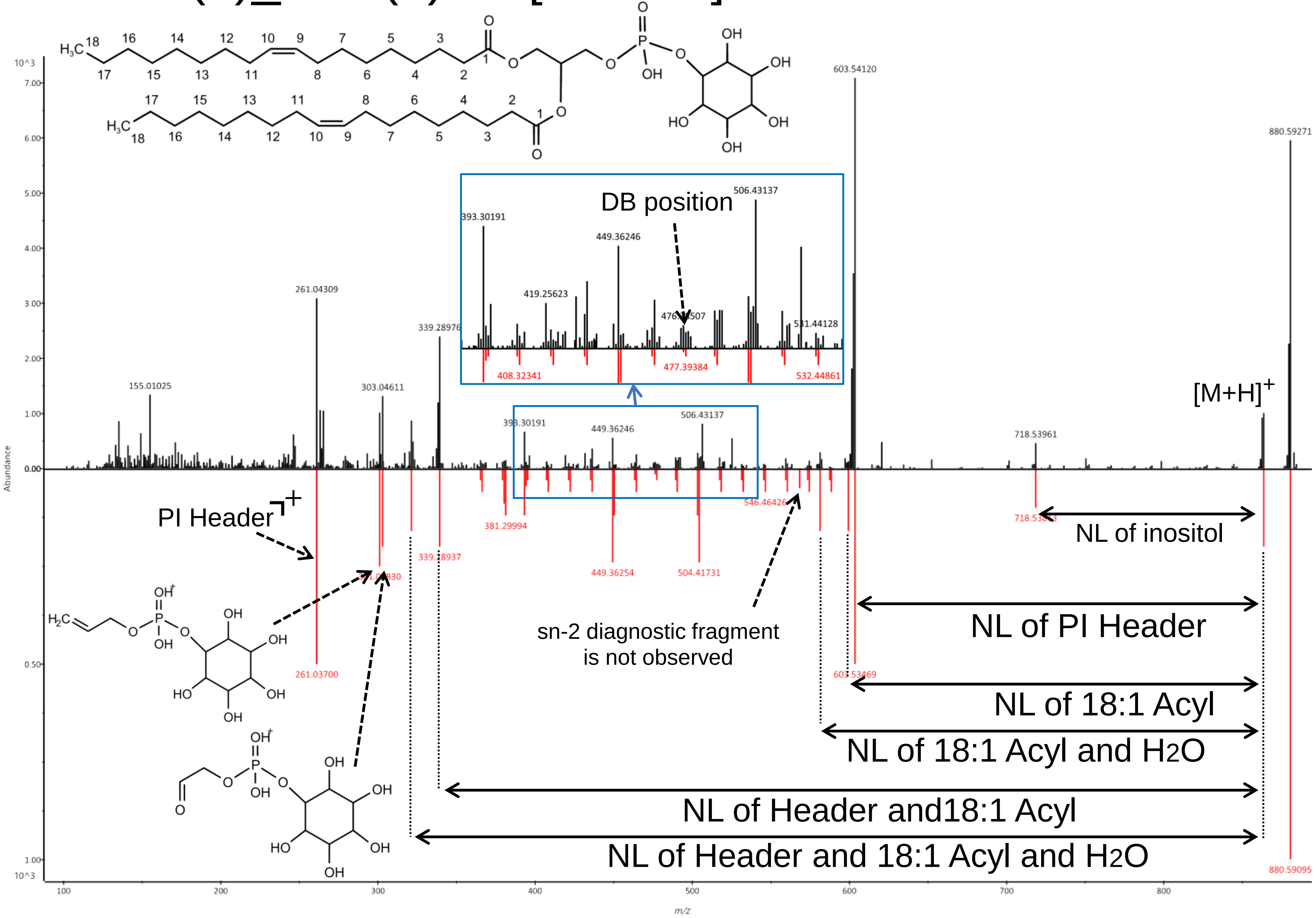

PI 18:0/20:4 as [M+Na]+

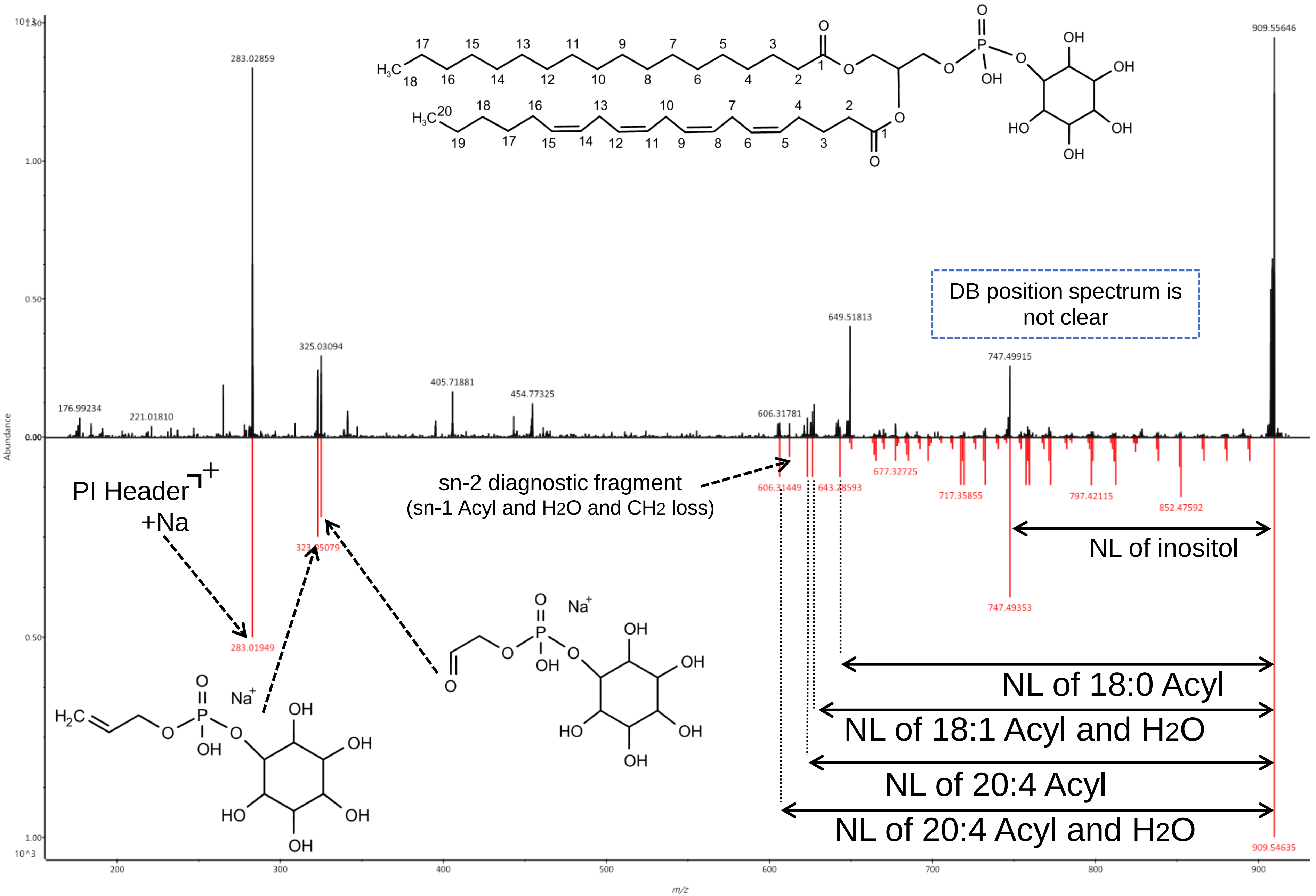

PS 18:1(9)_18:1(9) as [M+H]+

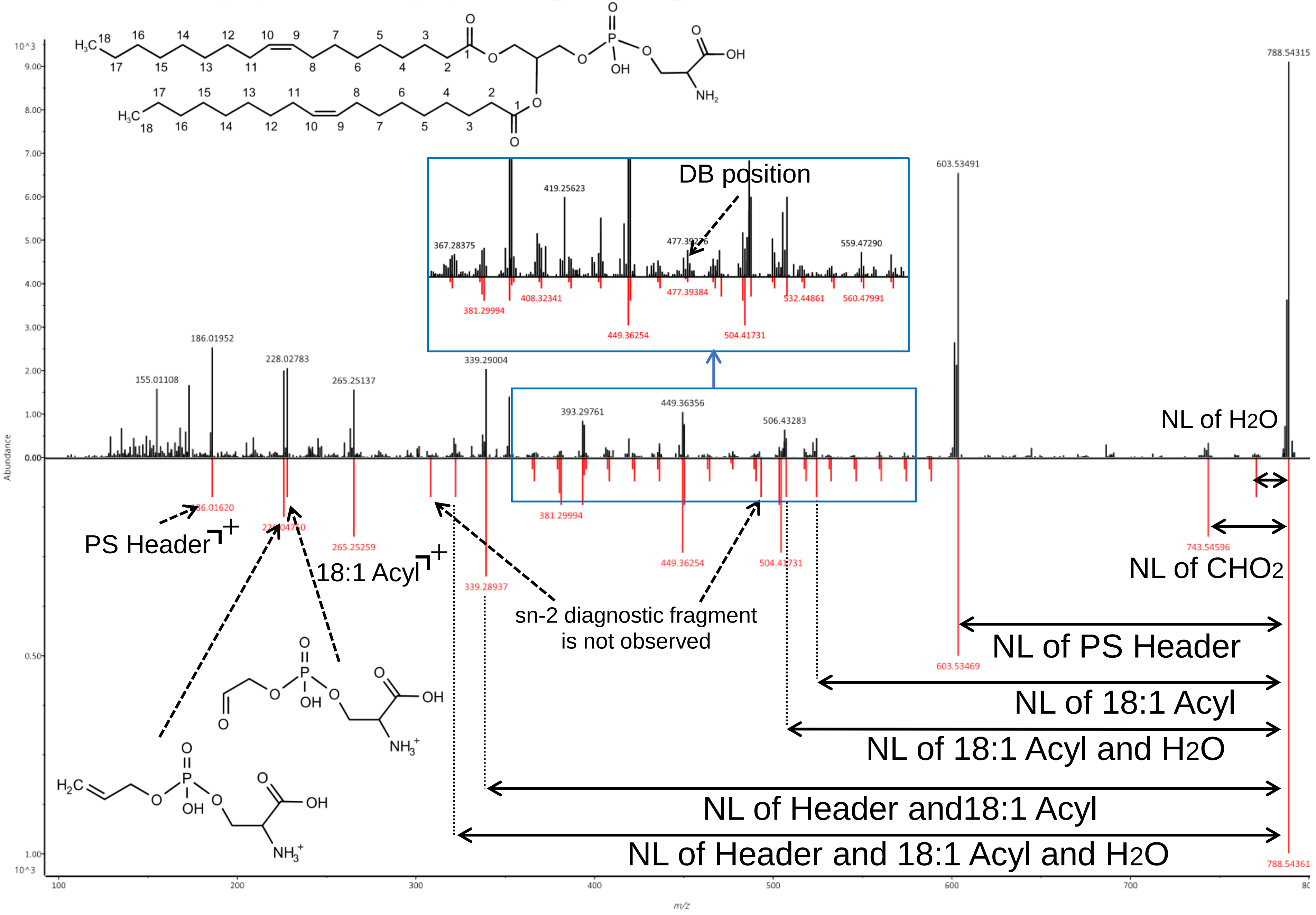

PS 18:1/18:1 as [M+Na]+

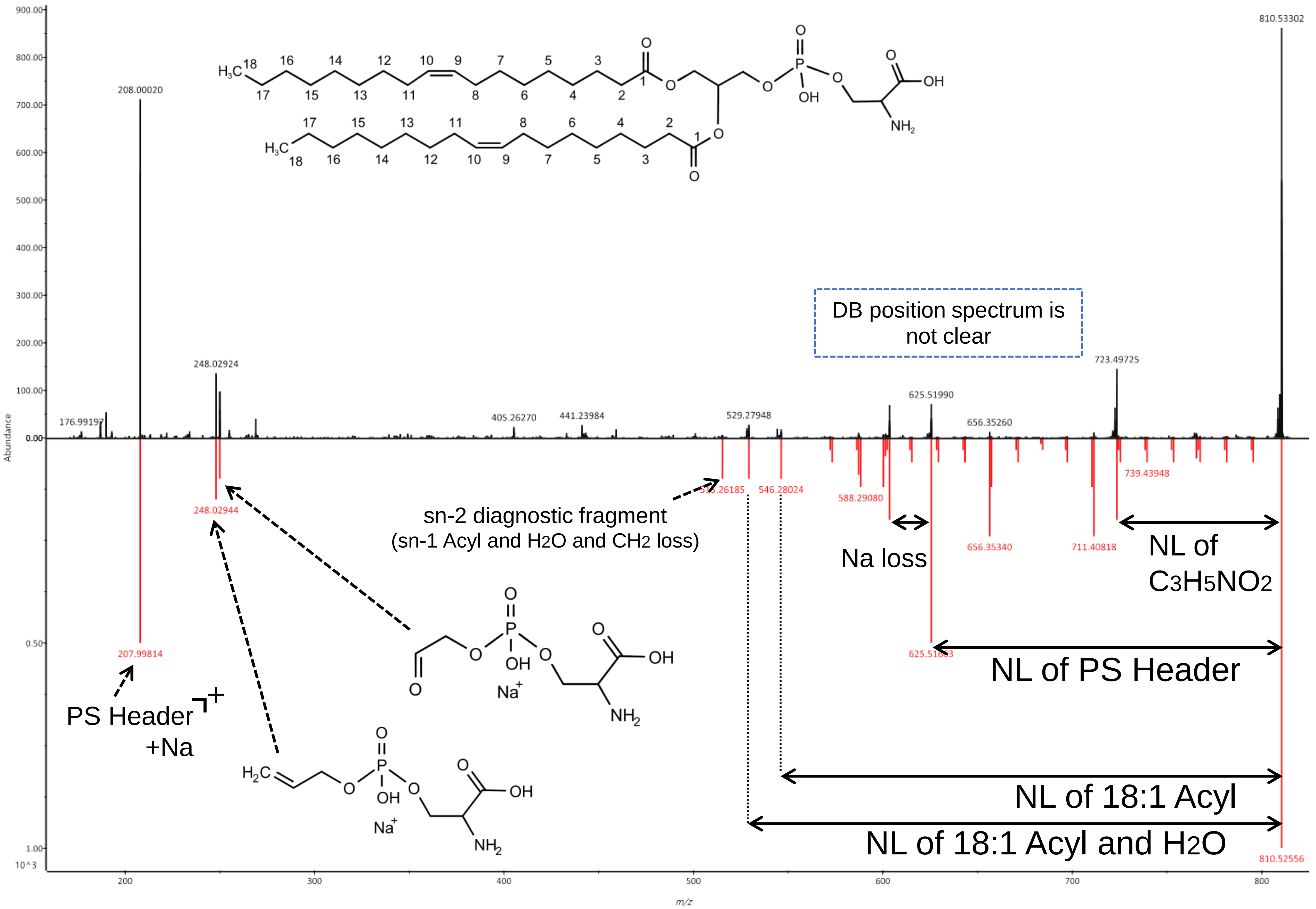

LPC 18:1(9)/0:0 as [M+H]+

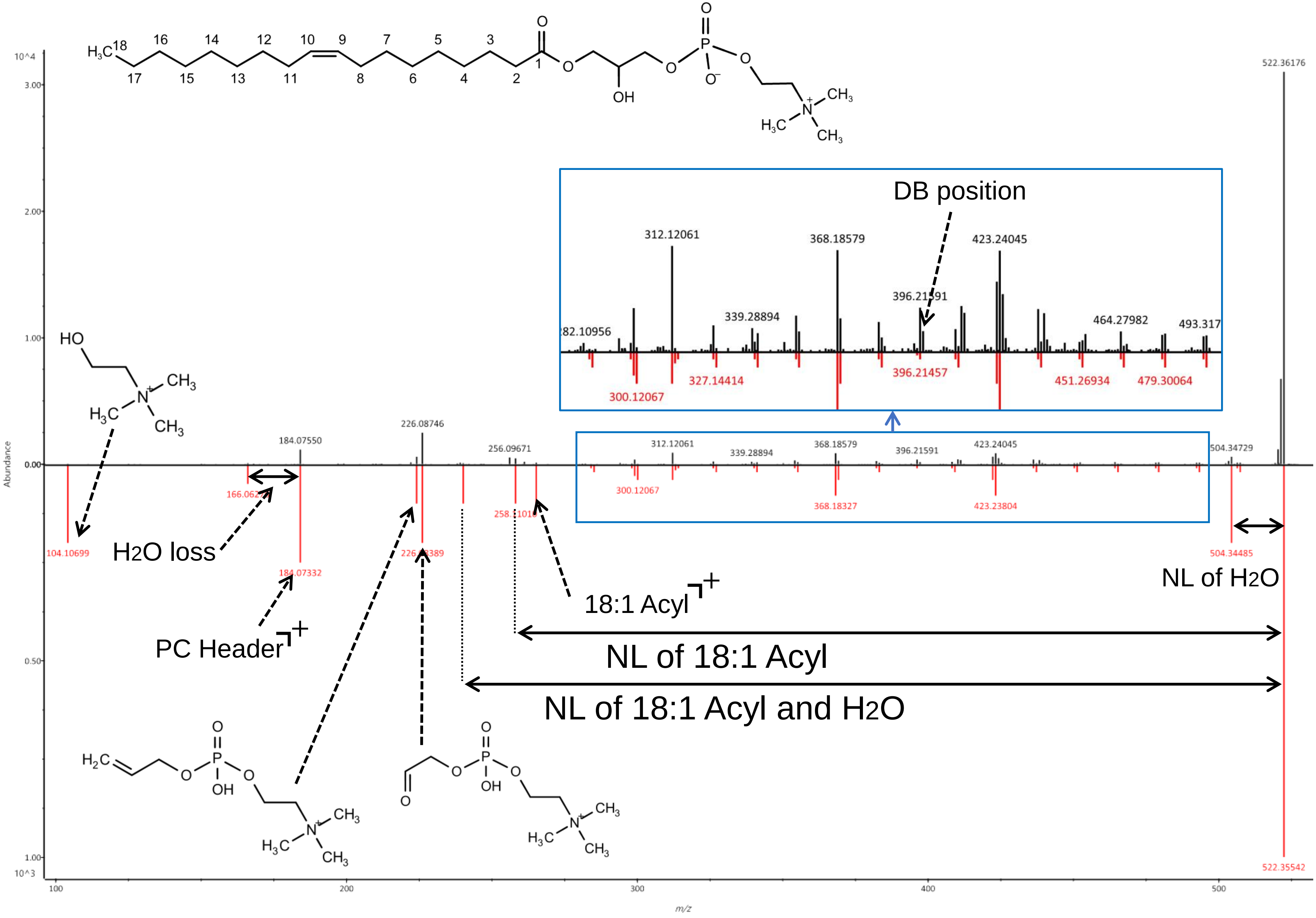

LPE 18:1(9)/0:0 as [M+H]+

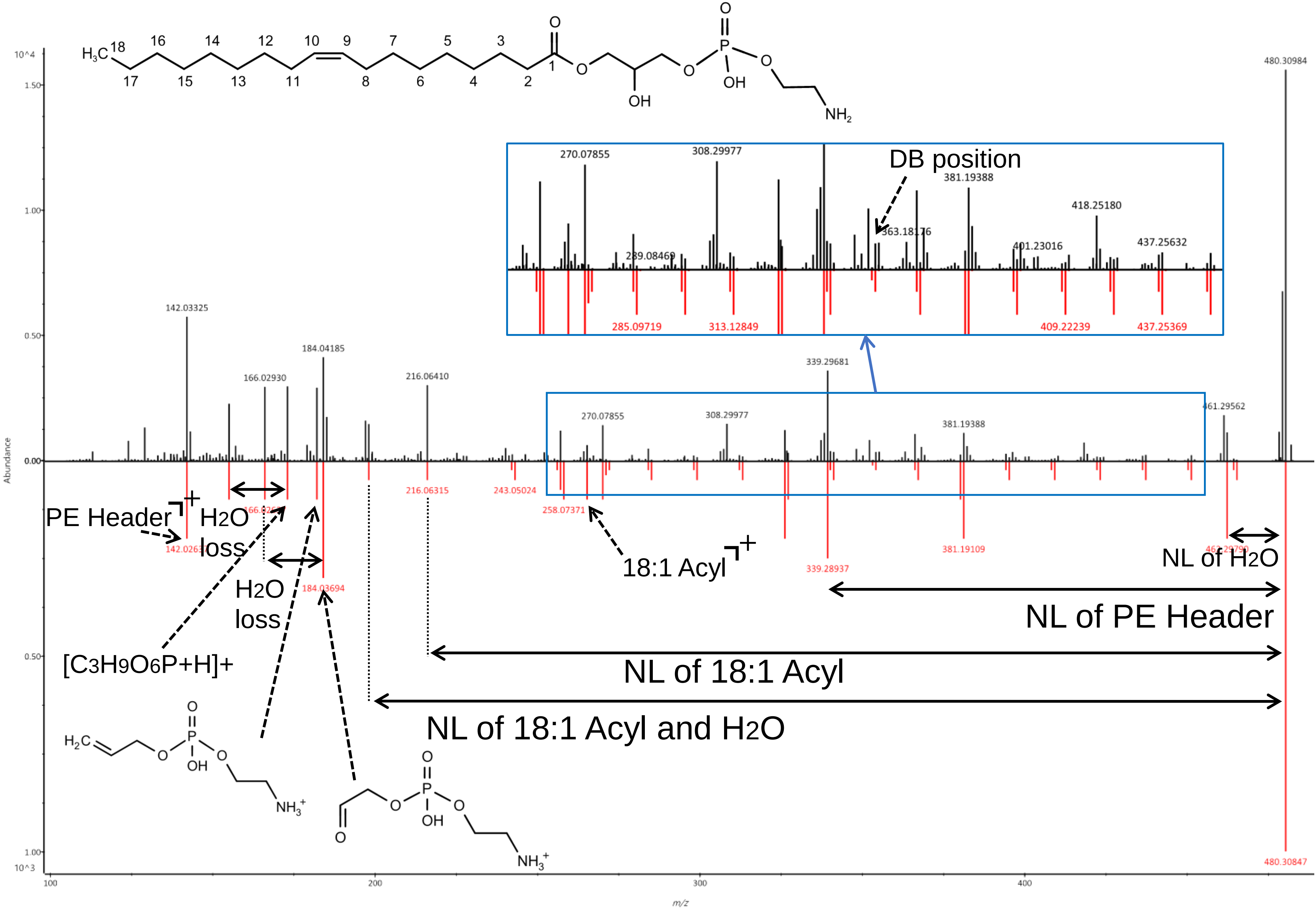

LPG 18:1/0:0 as [M+H]+

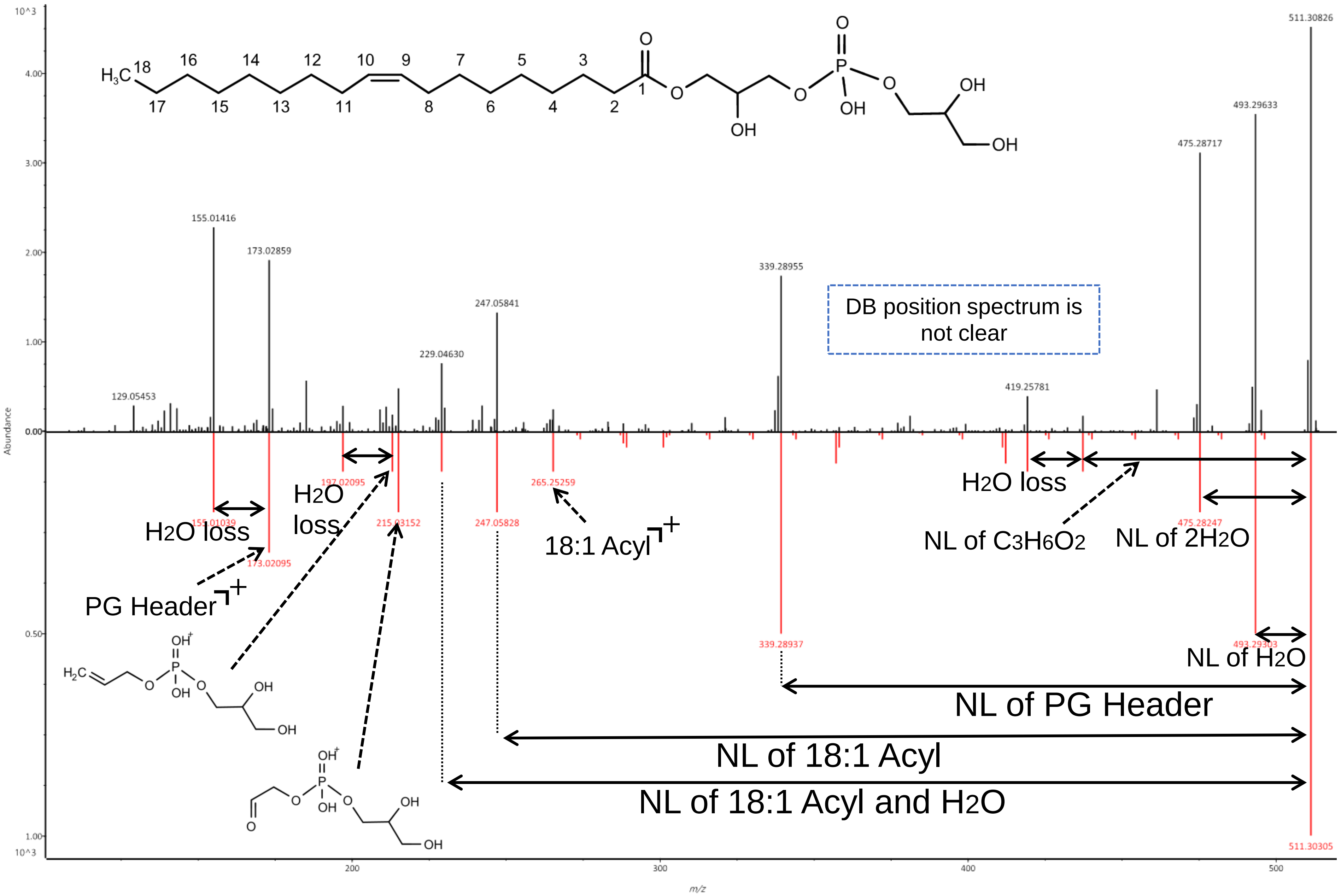

LPI 18:1/0:0 as [M+NH4]+

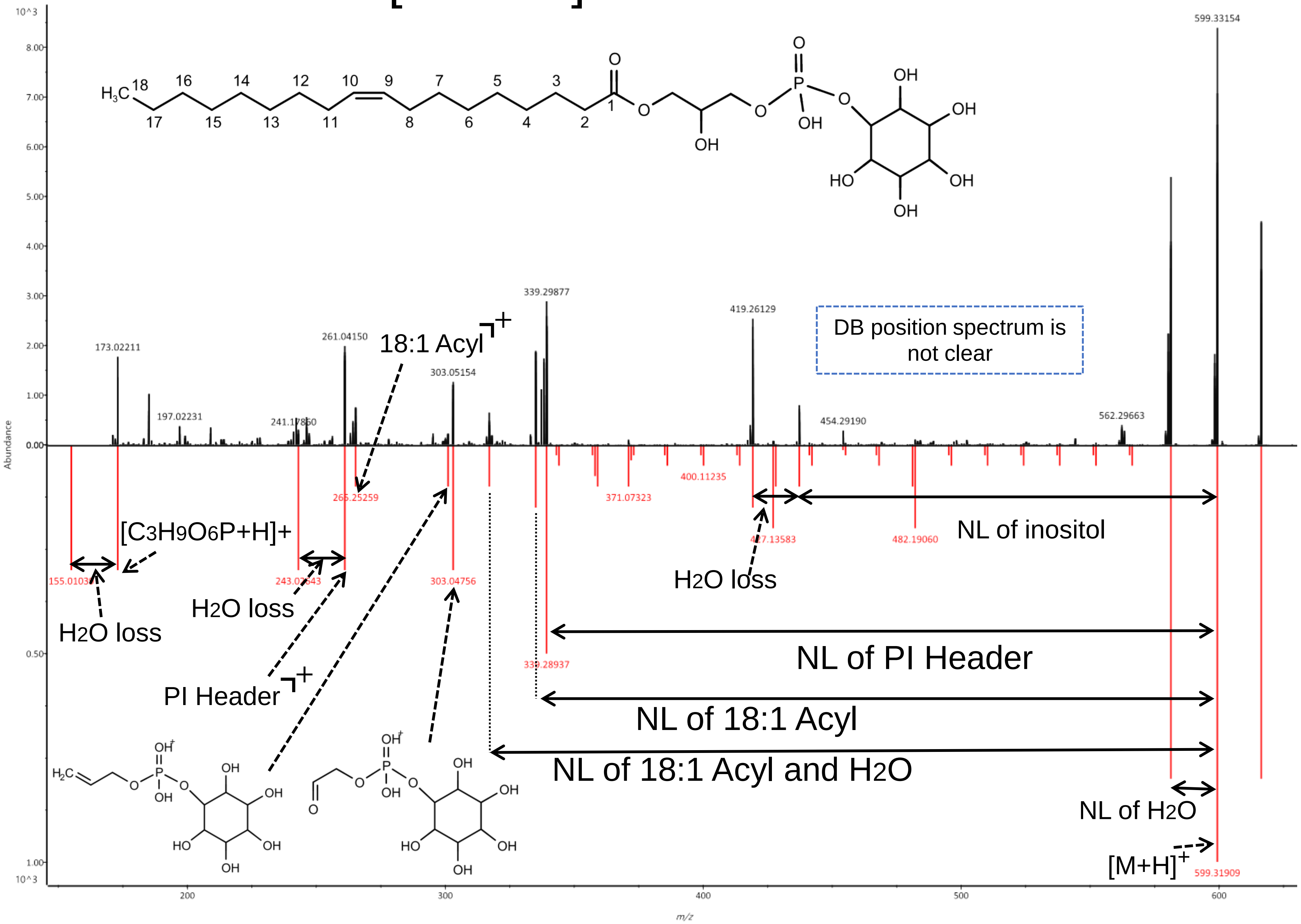

LPS 18:1(9)/0:0 as [M+H]+

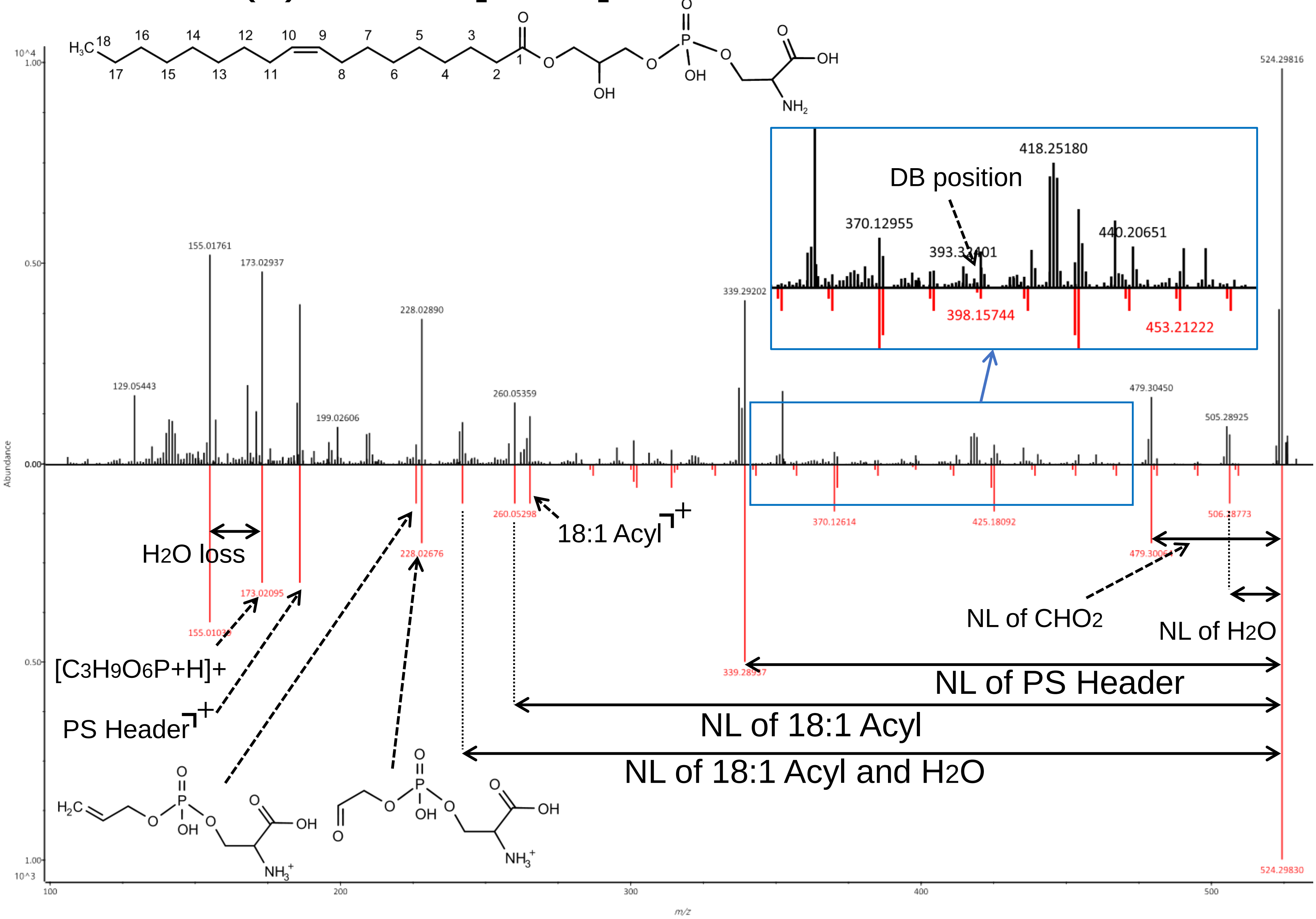

PC O-16:0/18:1(9) as [M+H]+

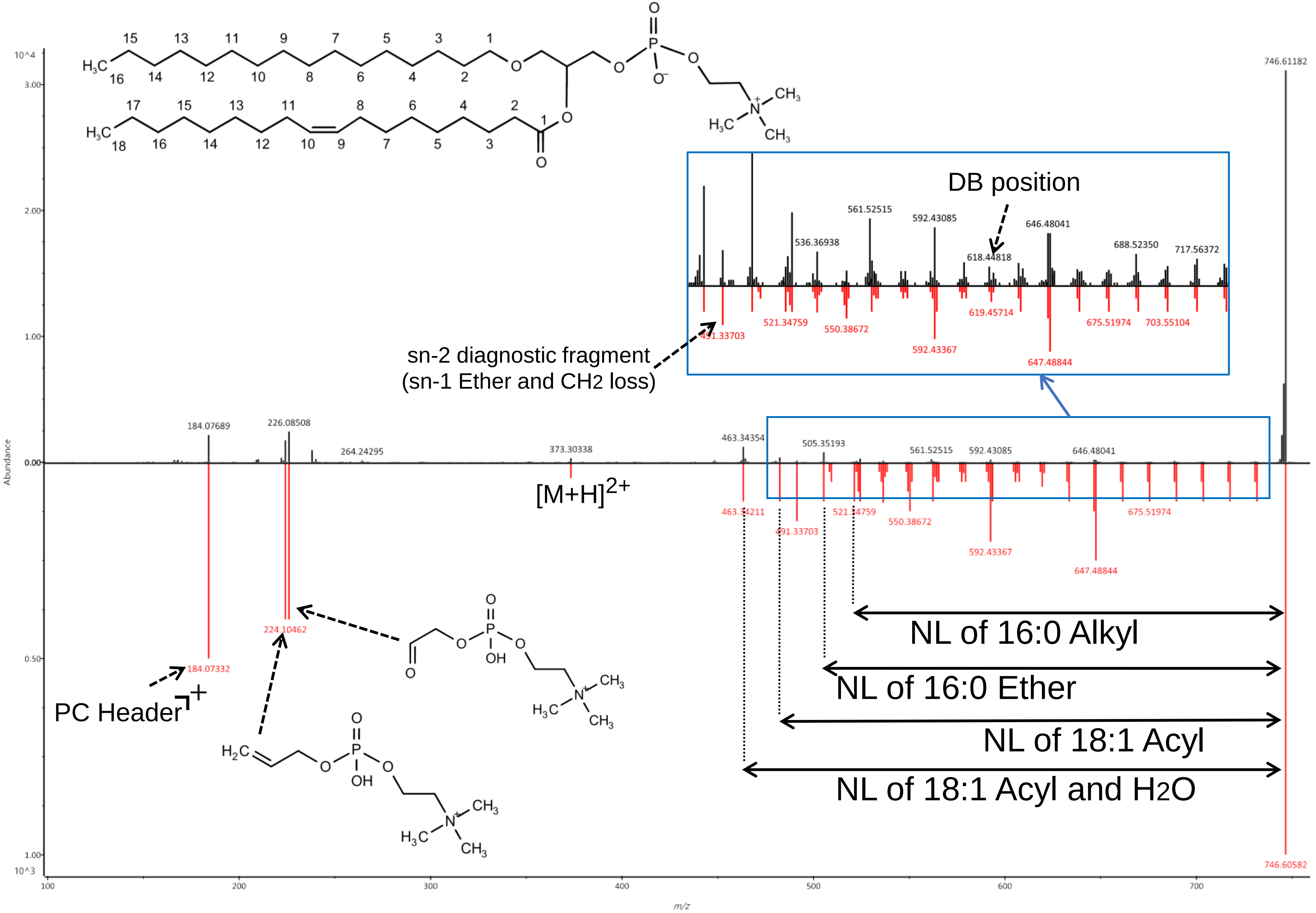

PC P-18:0/18:1(9) as [M+H]+

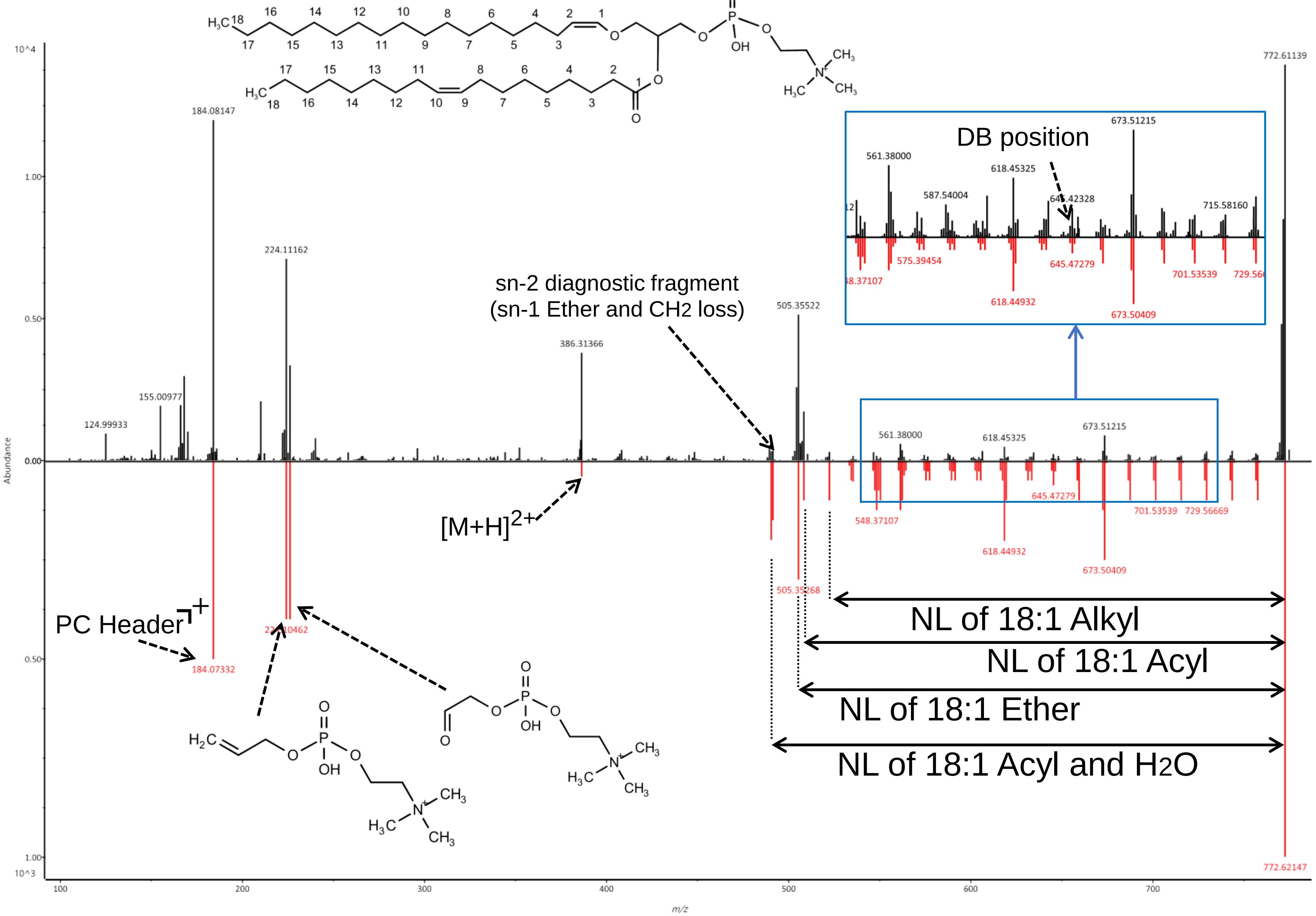

PE O-16:0_18:1(9) as [M+H]+

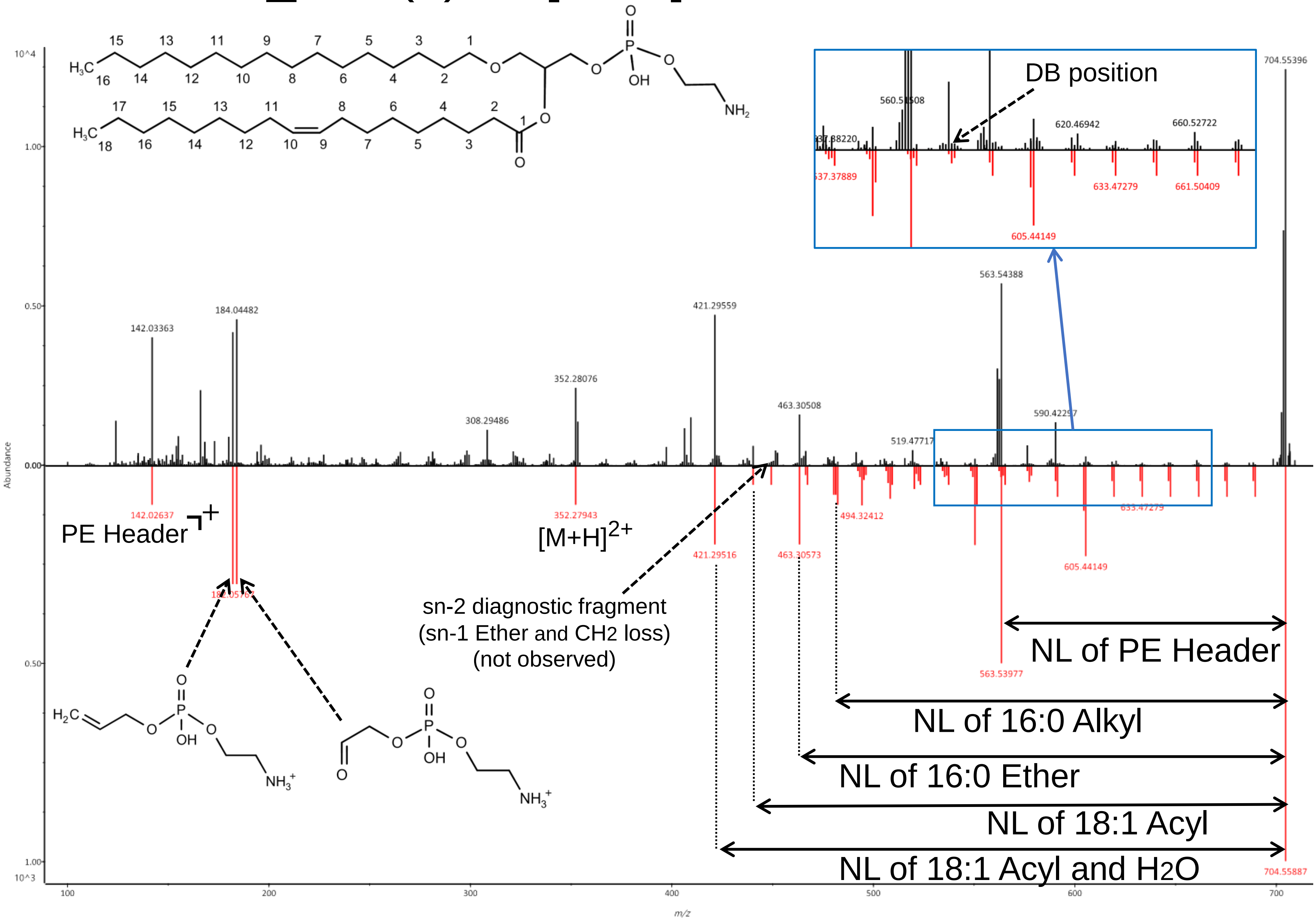

PE P-18:0_18:1(9) as [M+H]+

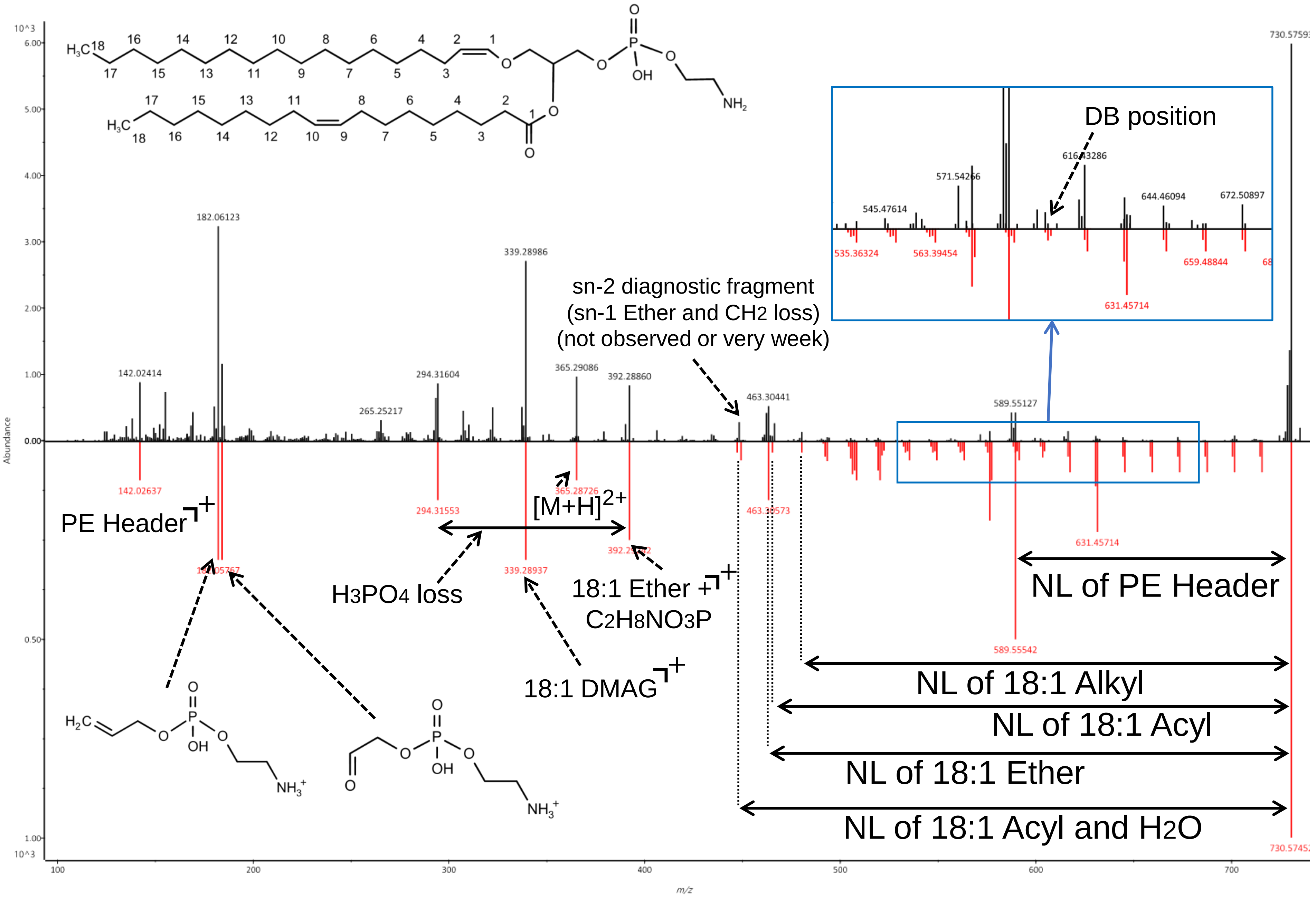

BMP 18:1(9)_18:1(9) as [M+NH4]+

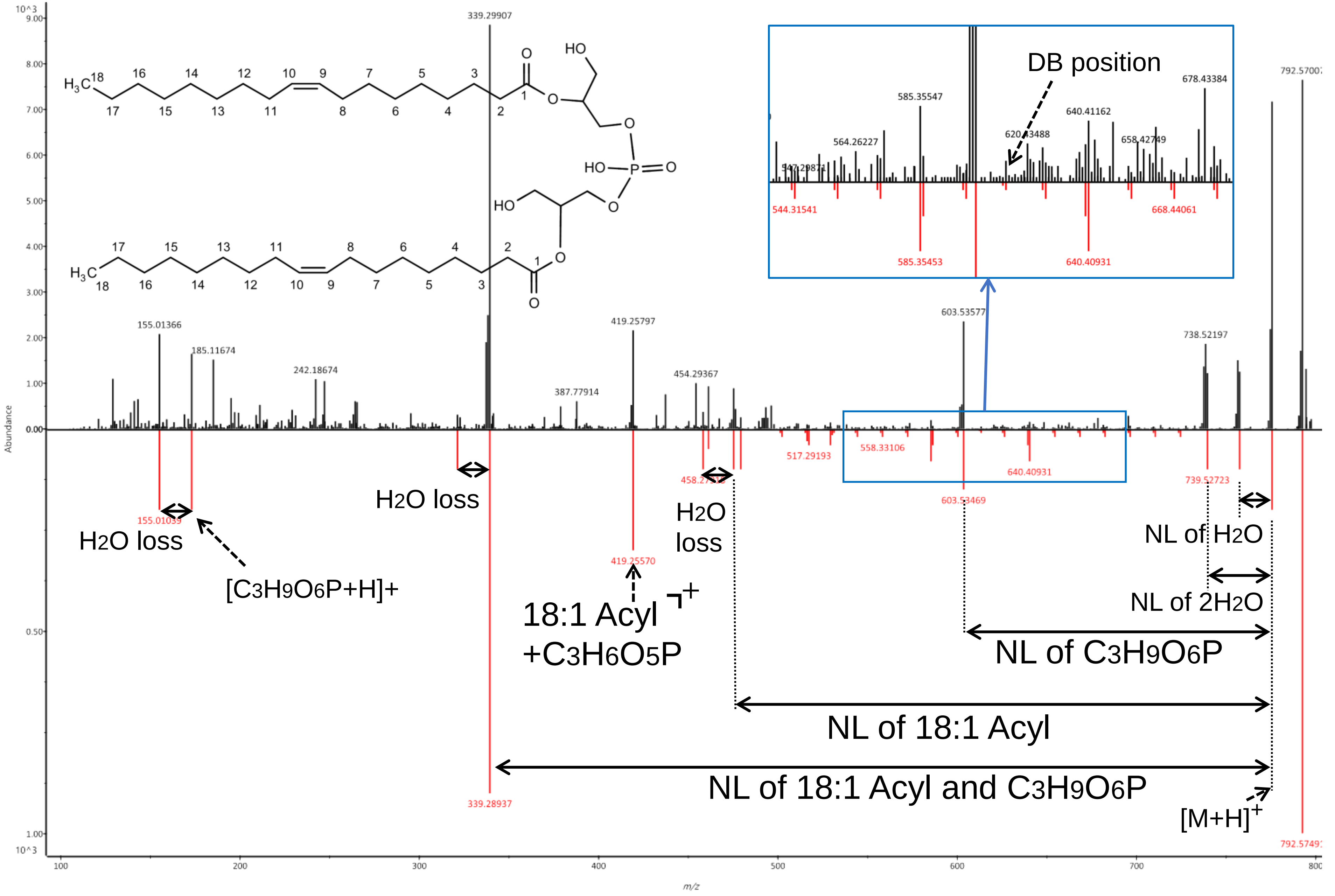

HBMP 18:1_18:1_18:1 as $[M+NH_4]^+$

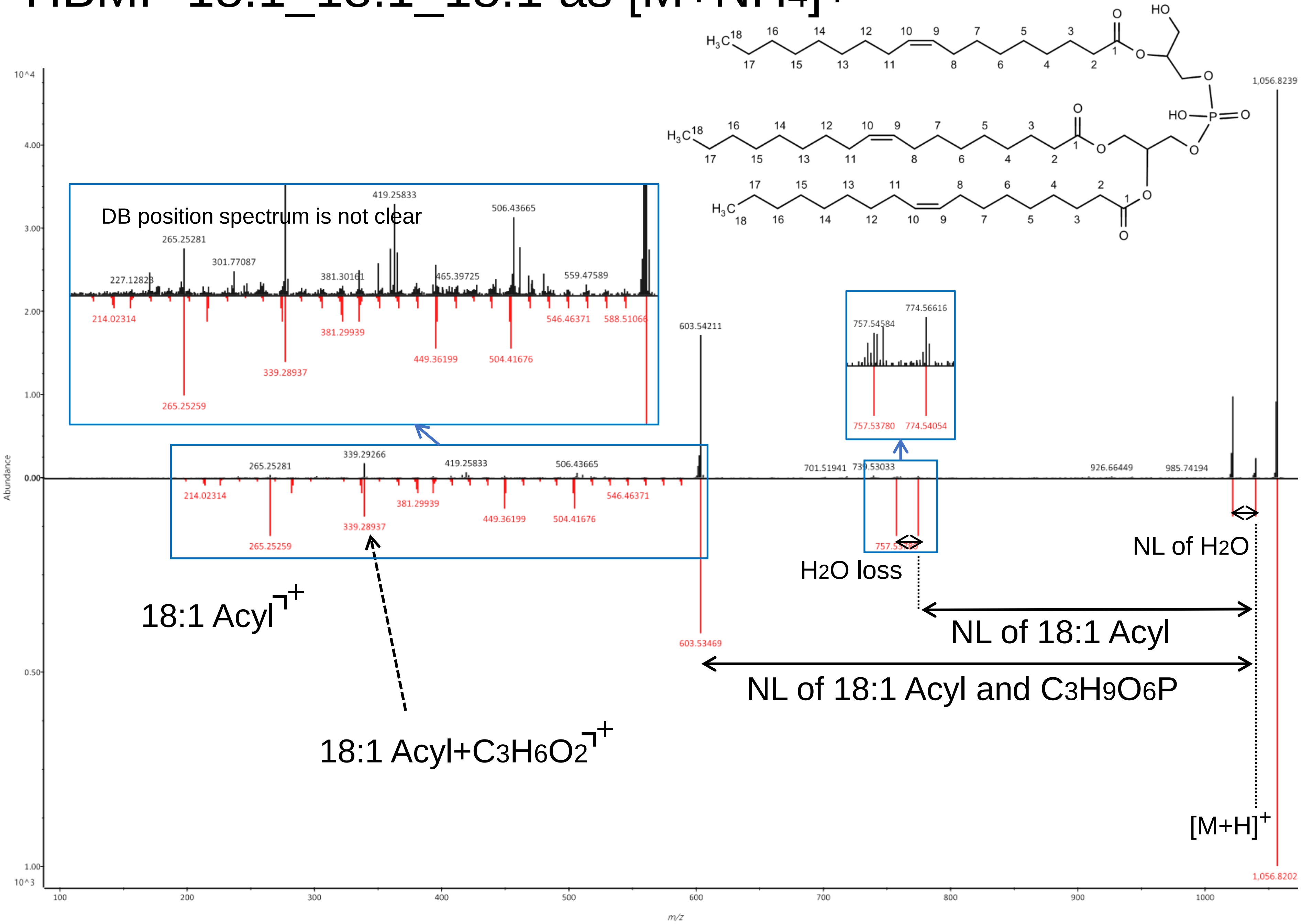

CL 16:0_18:1_16:0_18:1 as [M+NH4]+

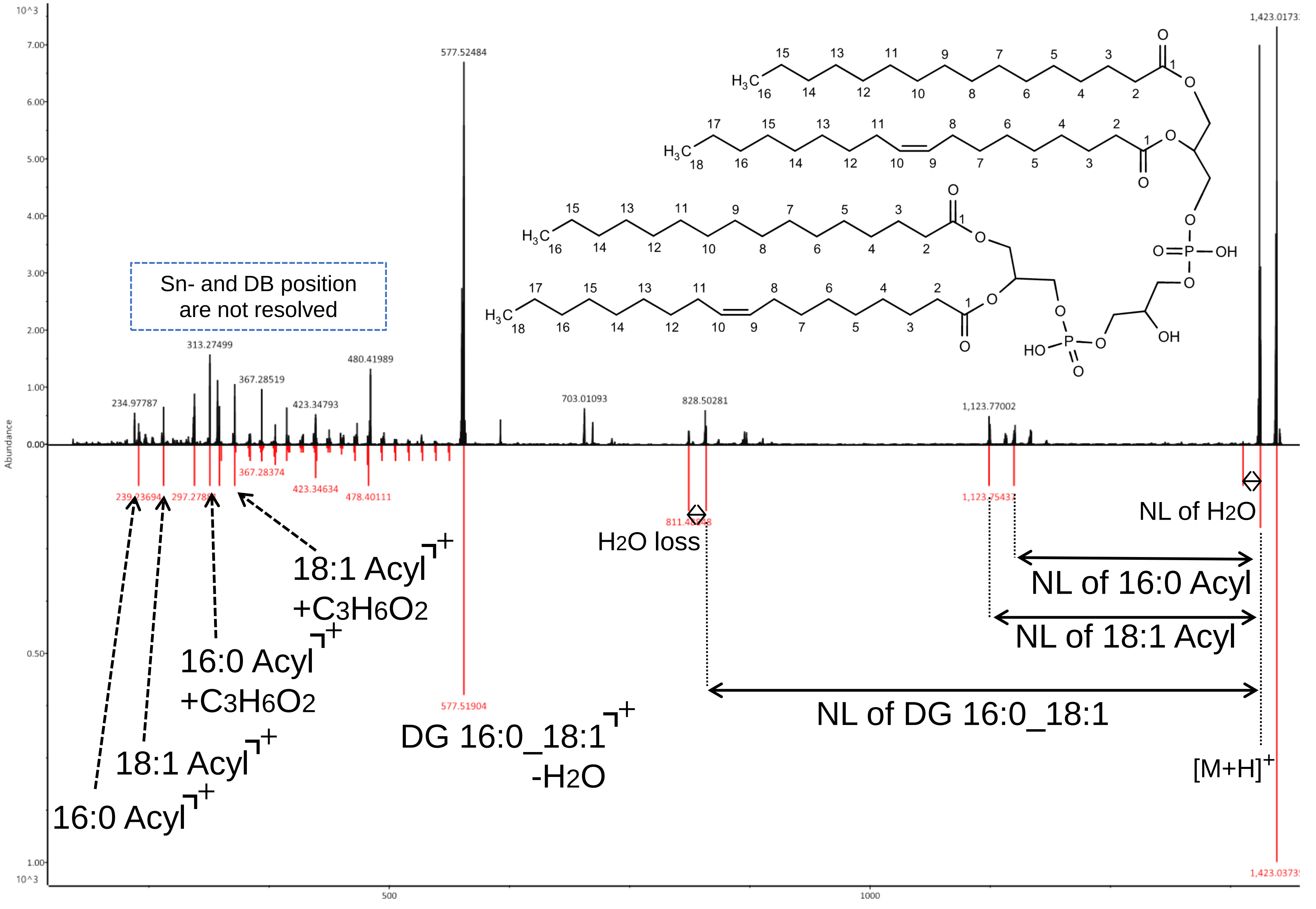

Supplementary Figure 3. EAD-MS/MS with kinetic energy at 10 eV, 14 eV, and 18 eV for phospholipids containing polyunsaturated fatty acids with more than three double bonds. EAD-MS/MS spectra of PC 18:3(9,12,15)/18:3(9,12,15), PC 22:6(4,7,10,13,16,19)/22:6(4,7,10,13,16,19), PC O-16:0/20:4(5,8,11,14), PE 18:0/20:4(5,8,11,14), PE 22:6(4,7,10,13,16,19)/22:6(4,7,10,13,16,19), and PG 22:6(4,7,10,13,16,19)/22:6(4,7,10,13,16,19) are shown with the same description method as described in **Figure 1d**.

PC 18:3(9,12,15)/18:3(9,12,15)

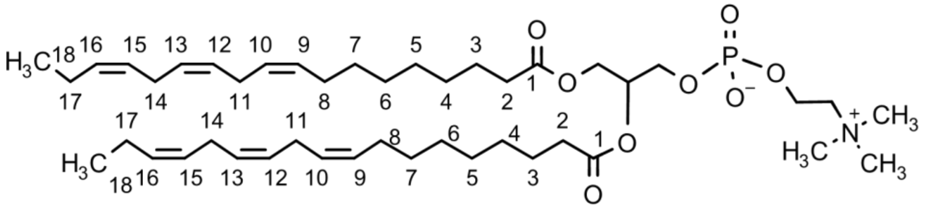

PC 18:3(9,12,15)/18:3(9,12,15) KE 10eV

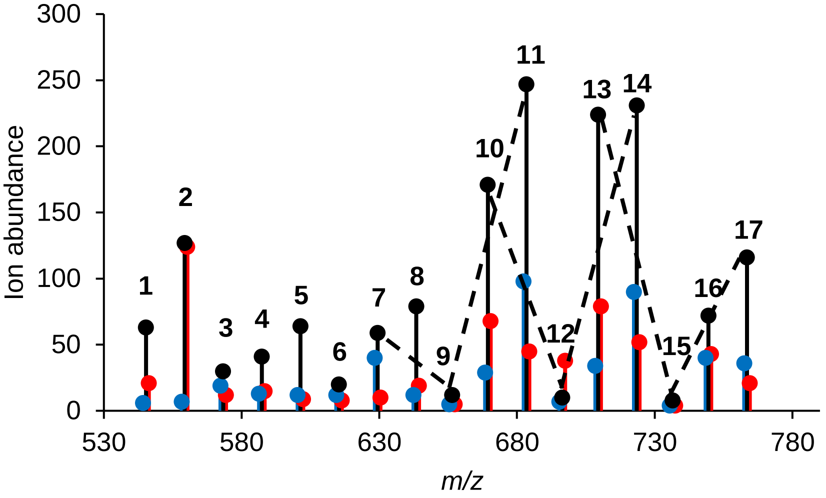

PC 18:3(9,12,15)/18:3(9,12,15) KE 14eV

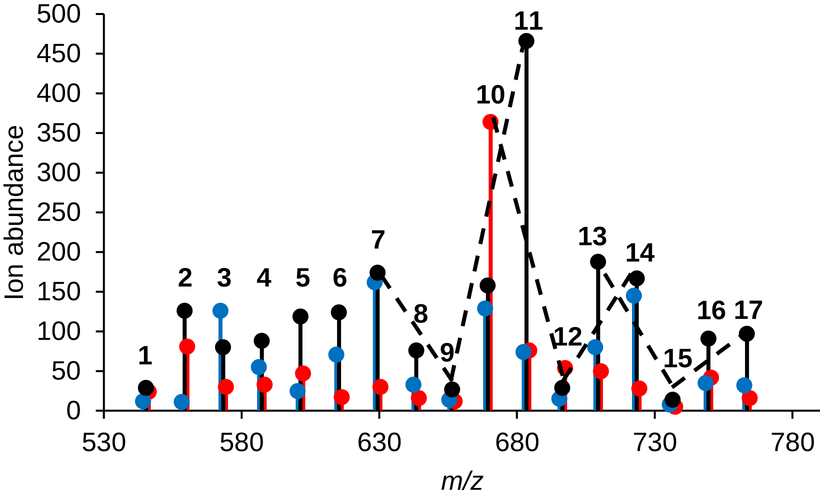

PC 18:3(9,12,15)/18:3(9,12,15) KE 18eV

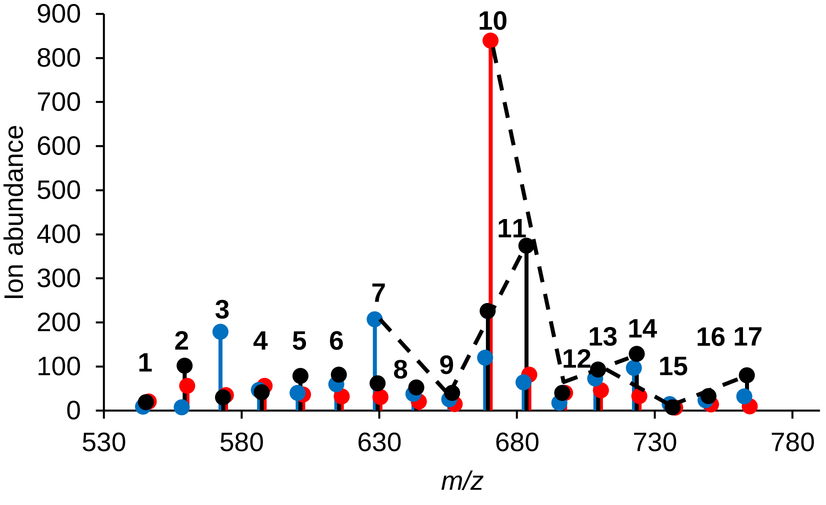

PC 22:6(4,7,10,13,16,19)/22:6(4,7,10,13,16,19)

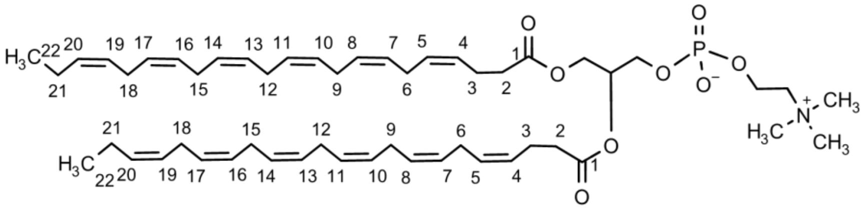

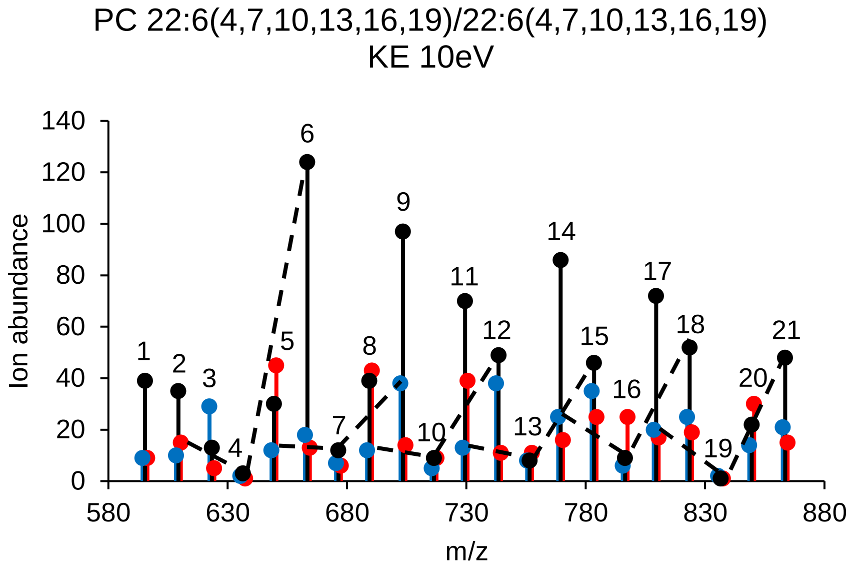

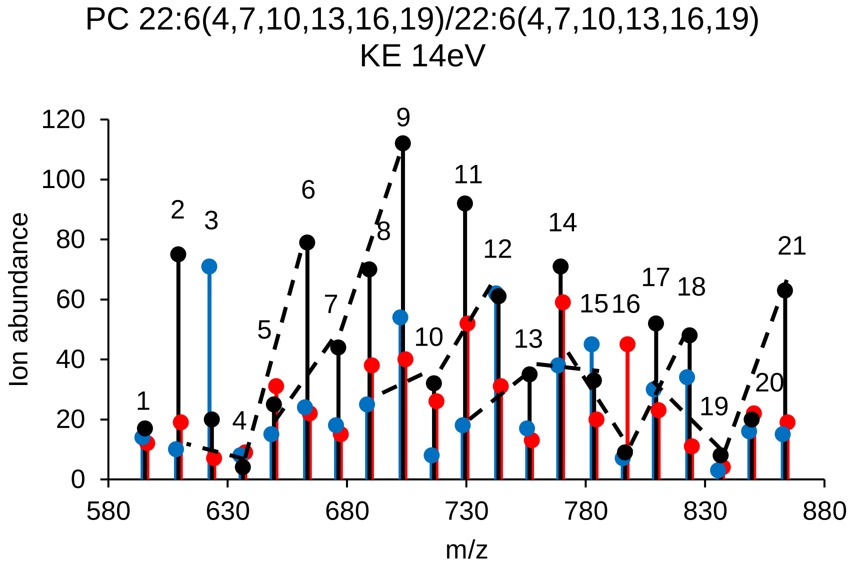

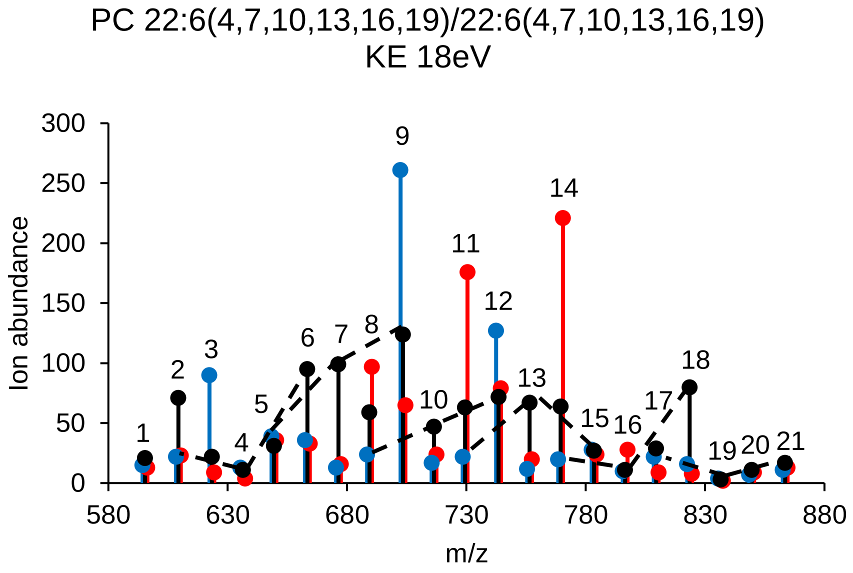

PC O-16:0/20:4(5,8,11,14)

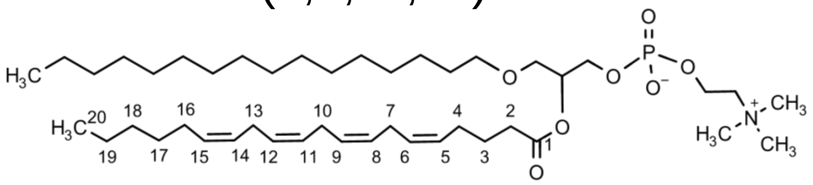

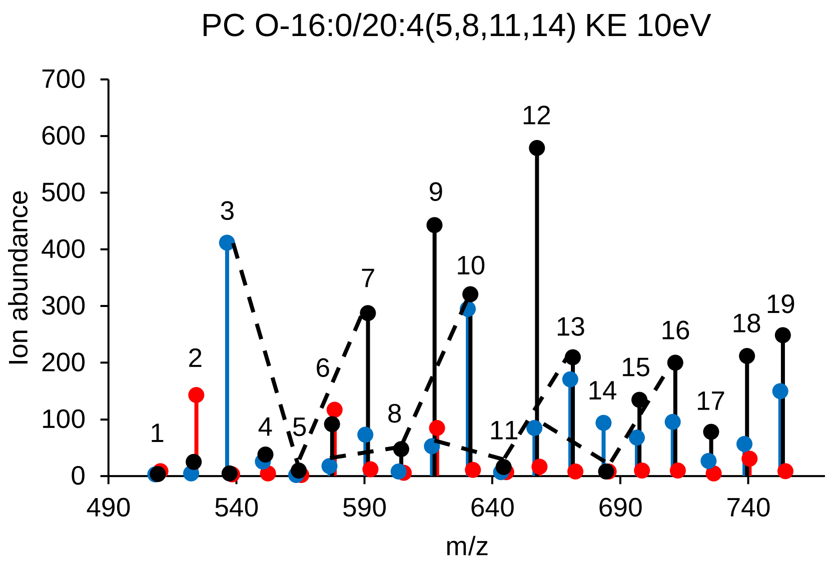

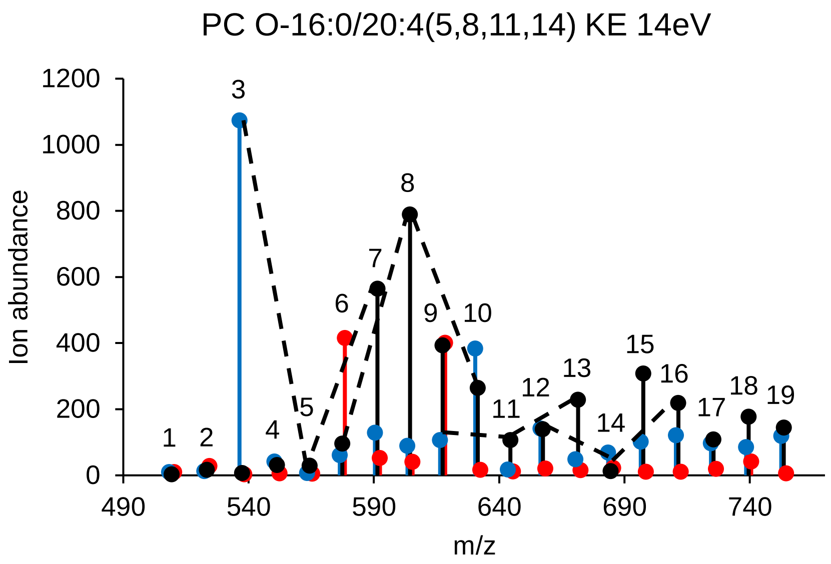

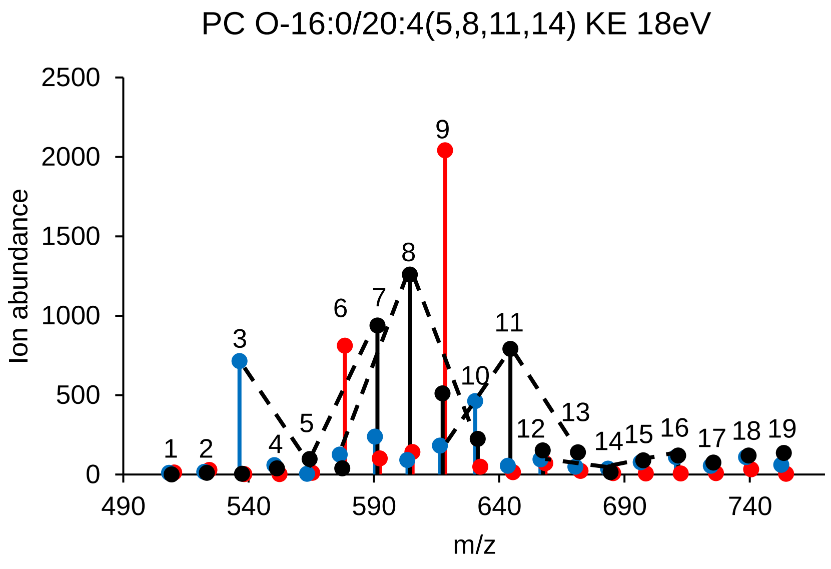

PE 18:0/20:4(5,8,11,14)

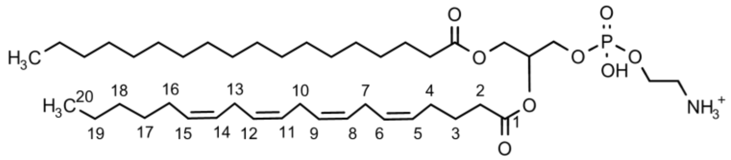

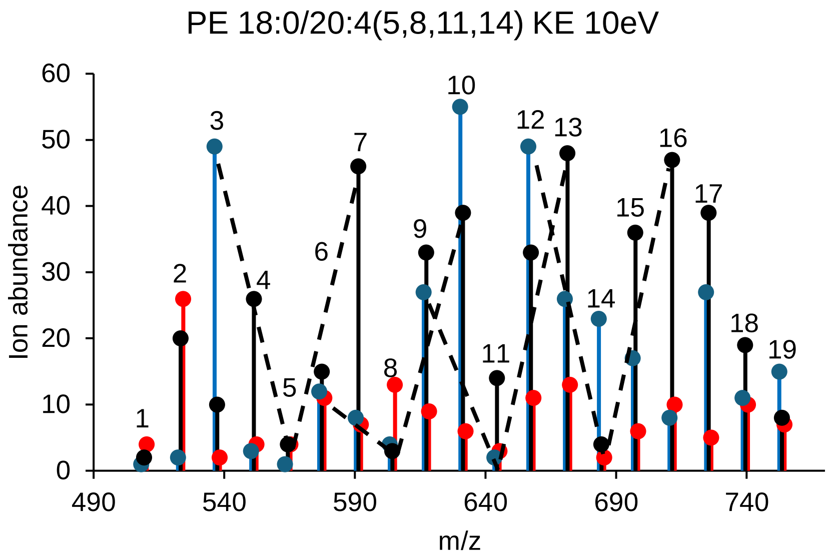

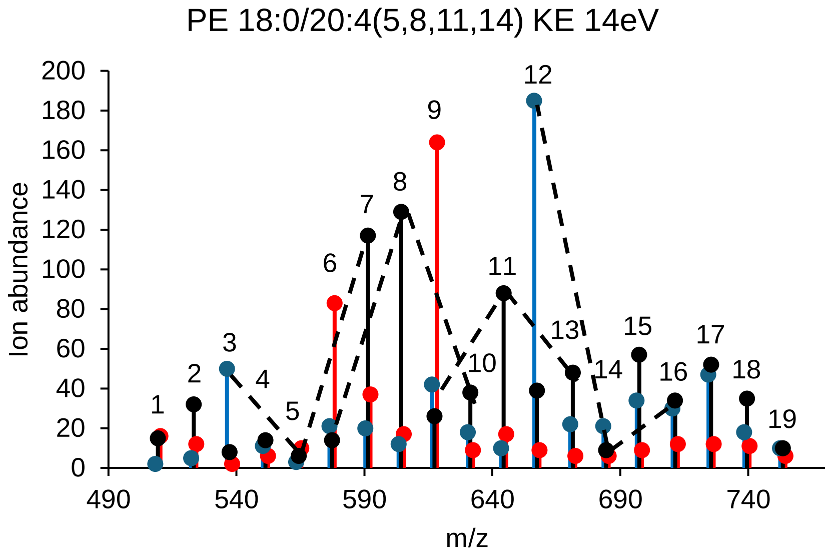

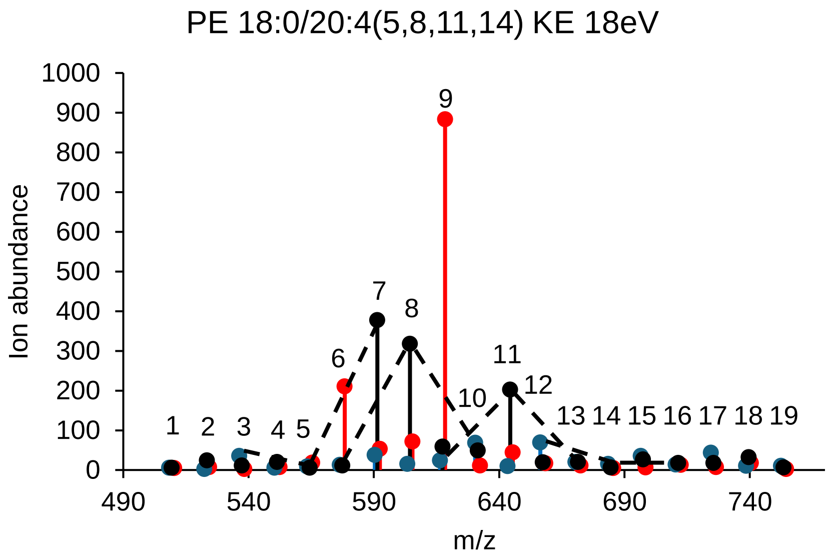

PE 22:6(4,7,10,13,16,19)/22:6(4,7,10,13,16,19)

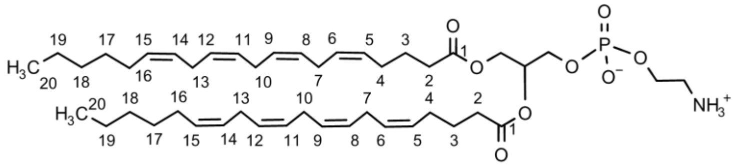

PE 22:6(4,7,10,13,16,19)/22:6(4,7,10,13,16,19)
KE 10eV

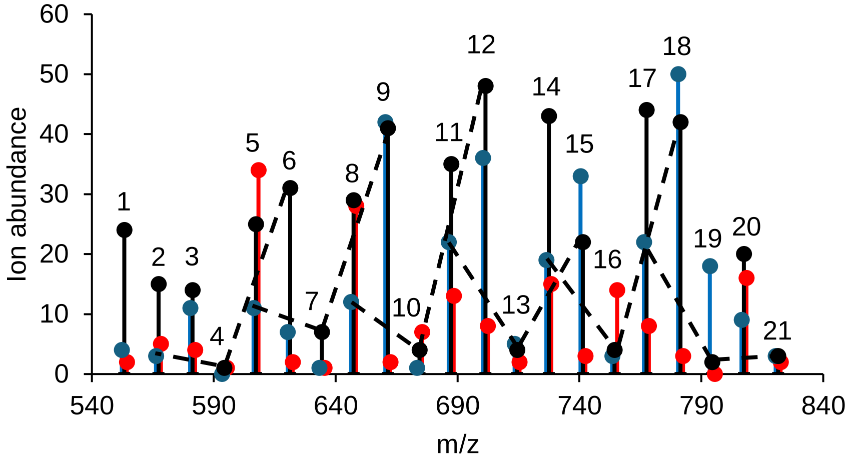

PE 22:6(4,7,10,13,16,19)/22:6(4,7,10,13,16,19)
KE 14eV

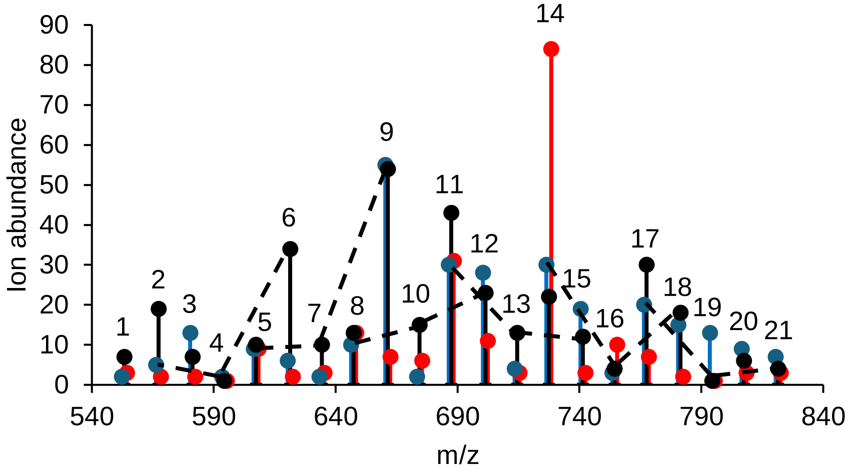

PE 22:6(4,7,10,13,16,19)/22:6(4,7,10,13,16,19)
KE 18eV

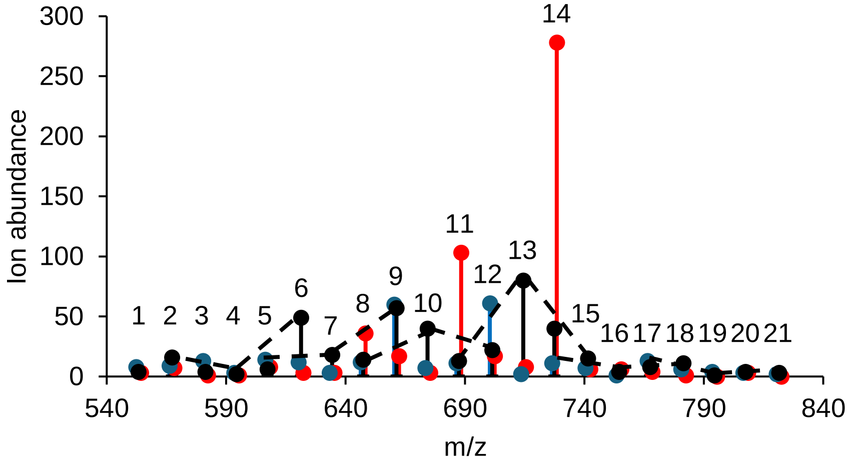

PG 22:6(4,7,10,13,16,19)/22:6(4,7,10,13,16,19)

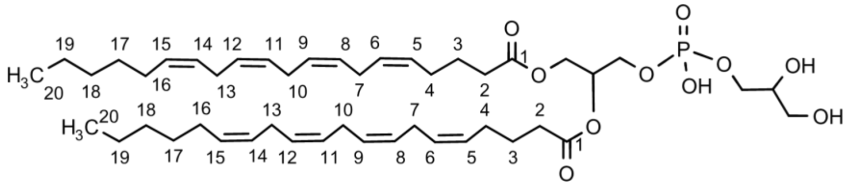

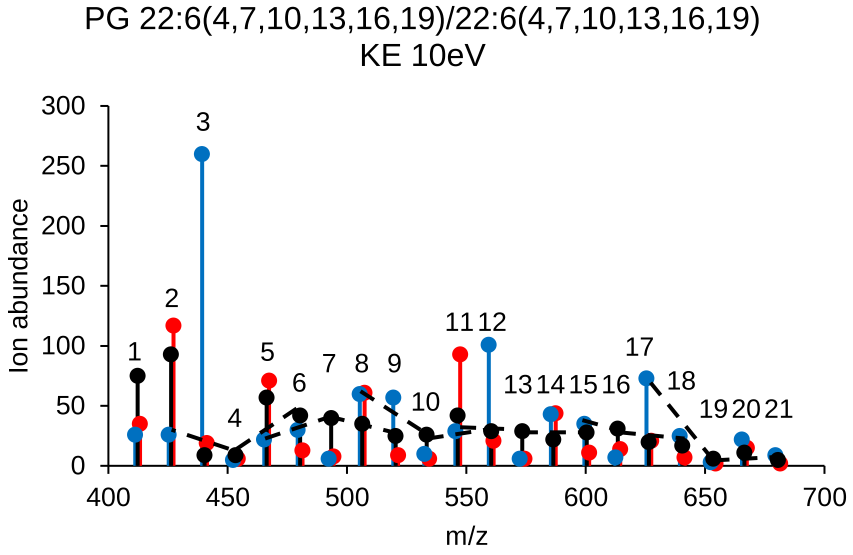

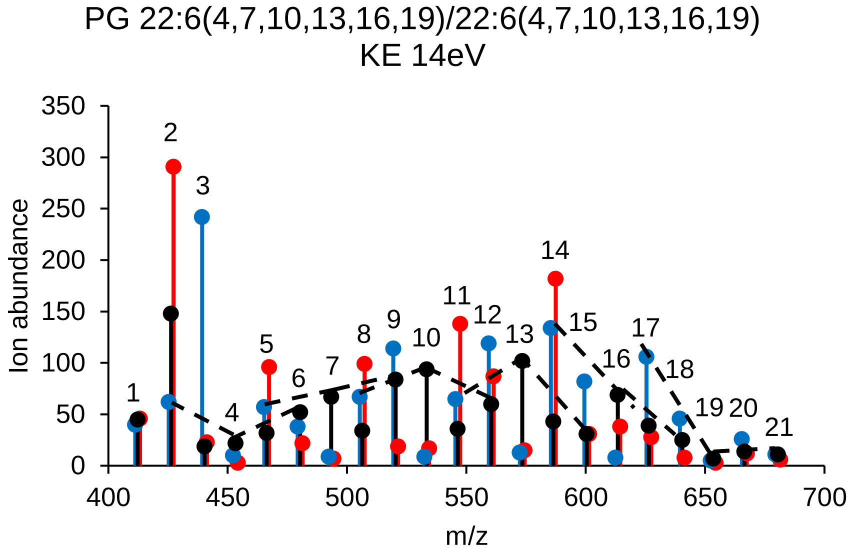

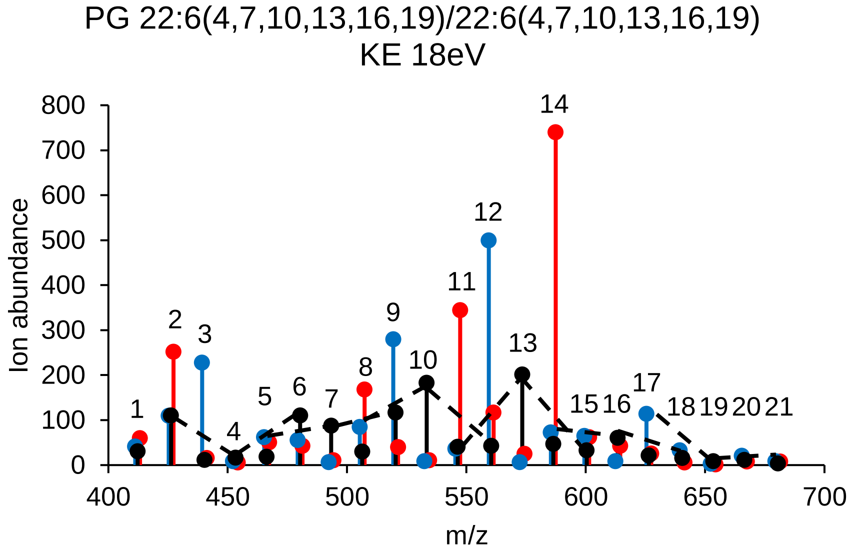

Supplementary Figure 4. Relationships between the kinetic energy 14 eV spectrum and the lipid structure in the glycerolipids and fatty acyls categories. The layout and the terms used are the same as those in Supplementary Figure 2. For free fatty acid (FA) and FAHFA, the spectra of the derived forms using 2-dimethylaminoethylamine (DMED) are described.

DG 18:1(9)_18:1(9) as [M+NH4]+

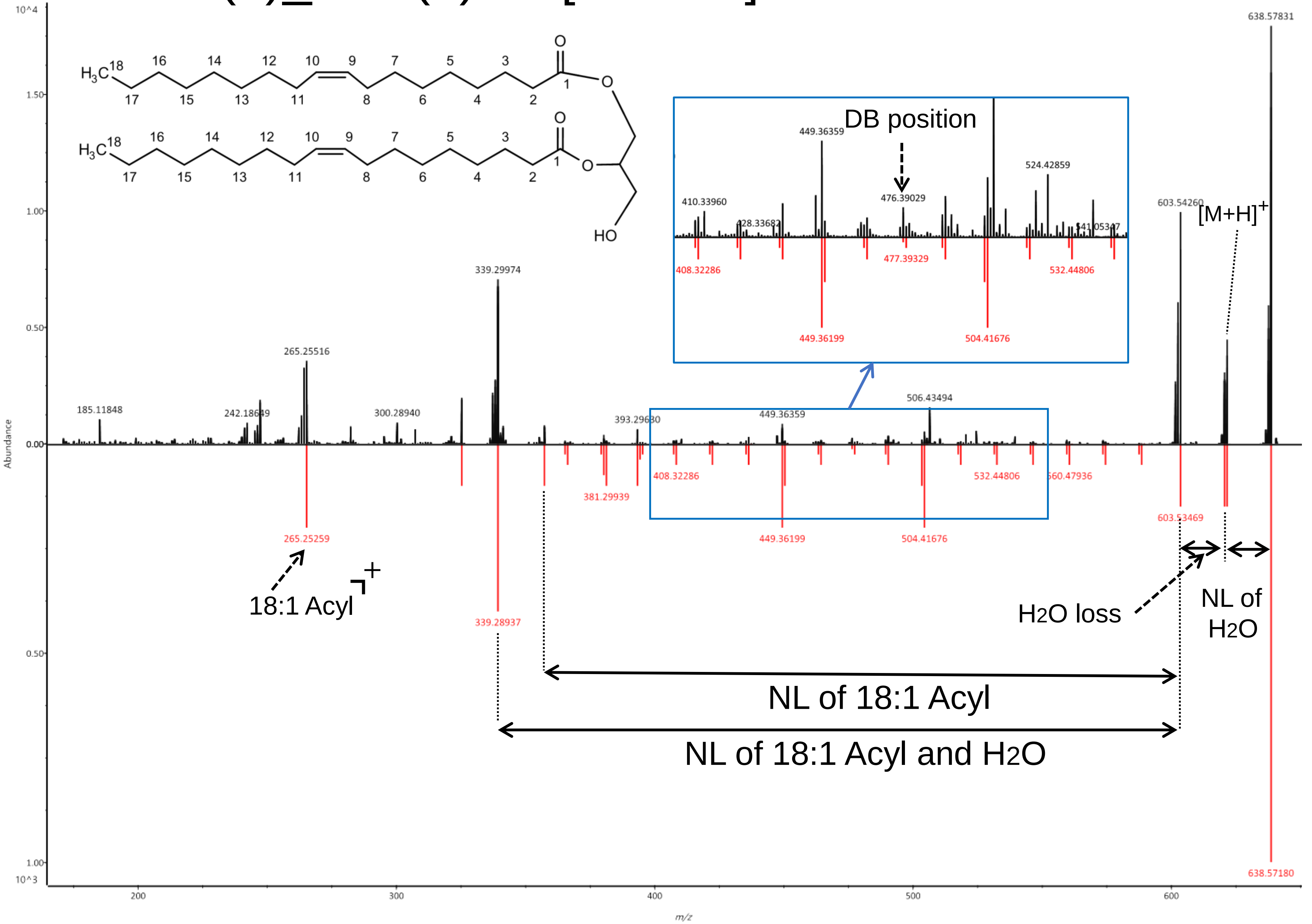

DG 18:1(9)/18:1(9) as [M+Na]+

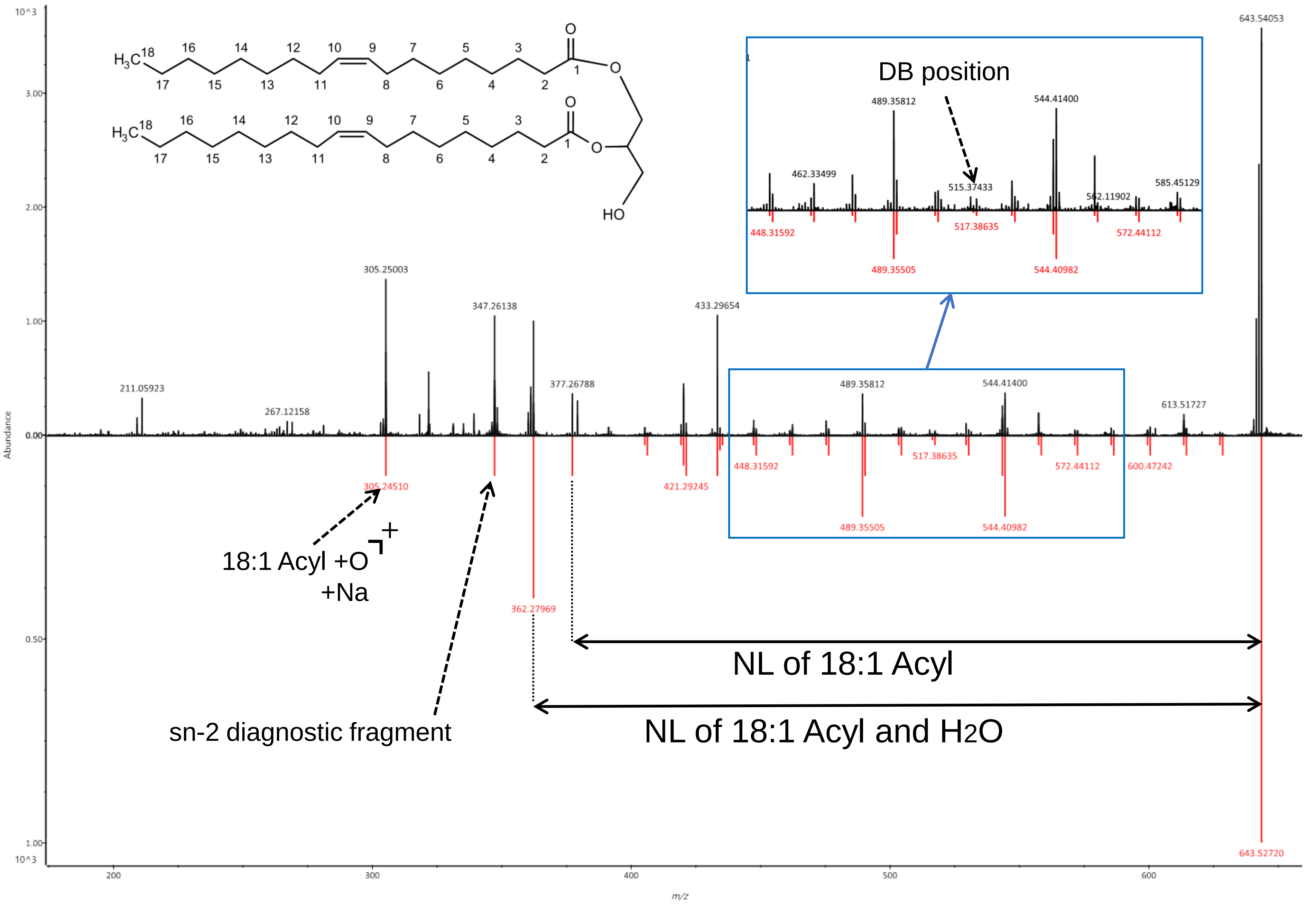

TG 18:1(9)_18:1(9)_18:1(9) as [M+NH4]+

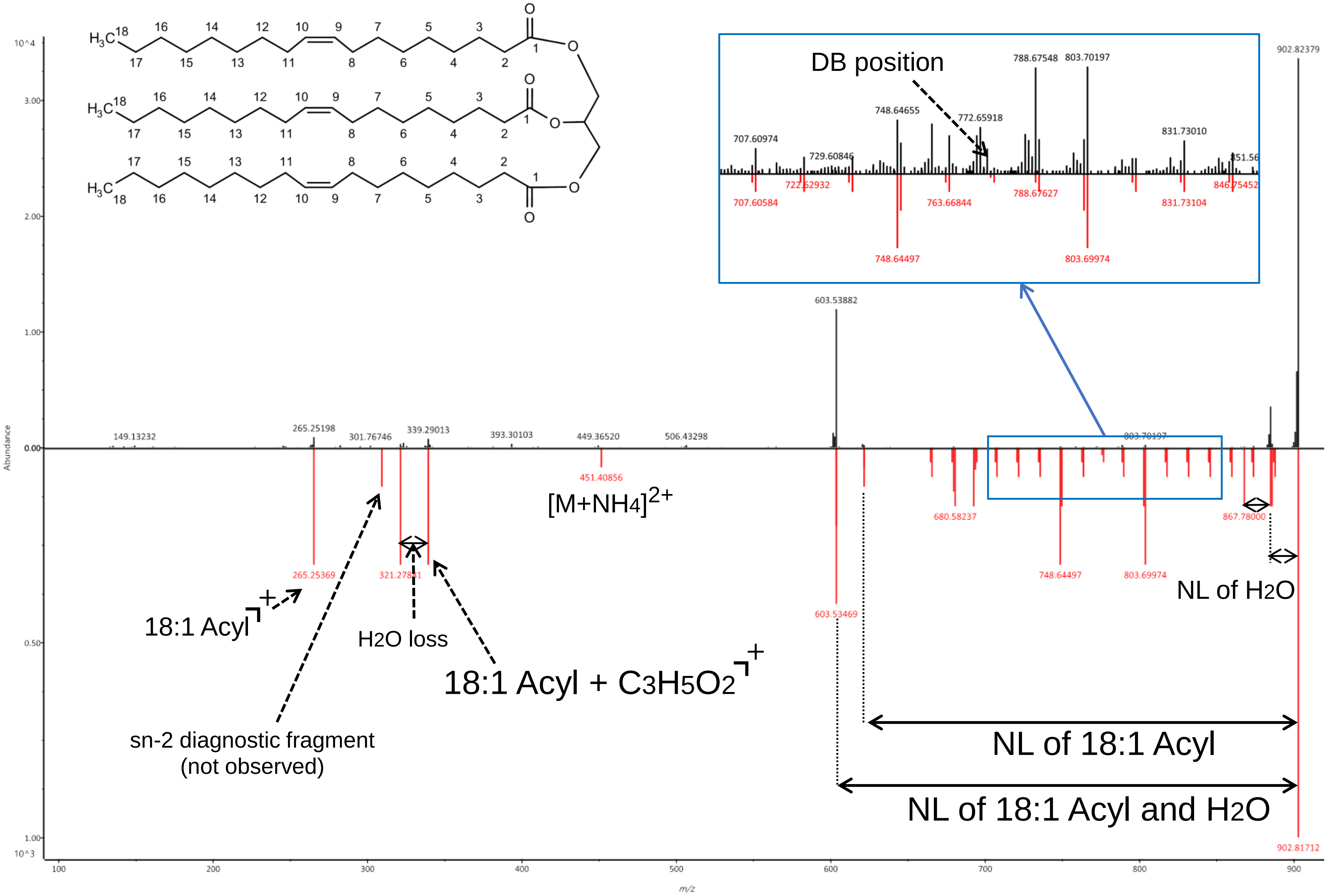

TG 18:1(9)/18:1(9)/18:1(9) as [M+Na]+

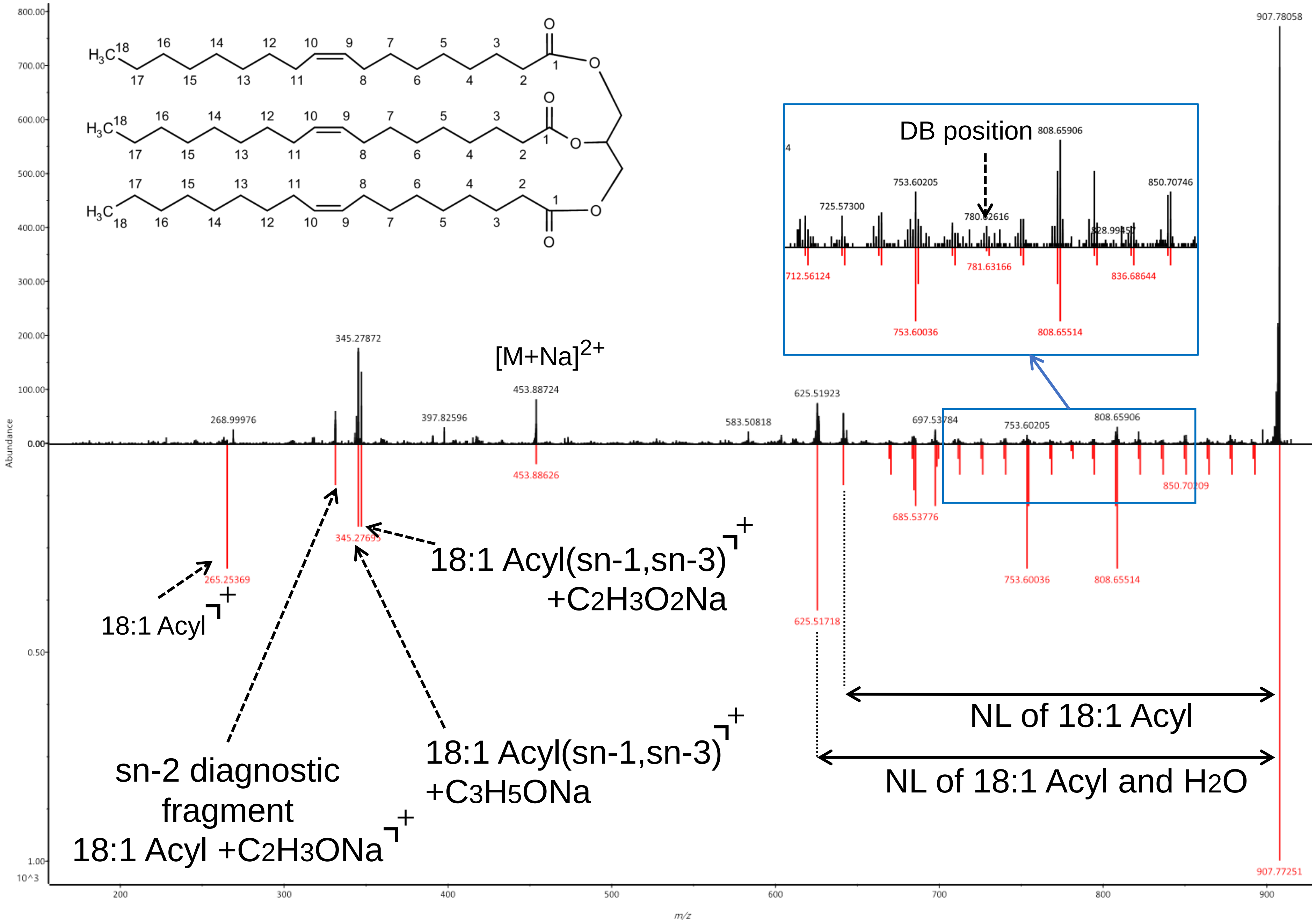

CAR 18:1(9) as [M+H]+

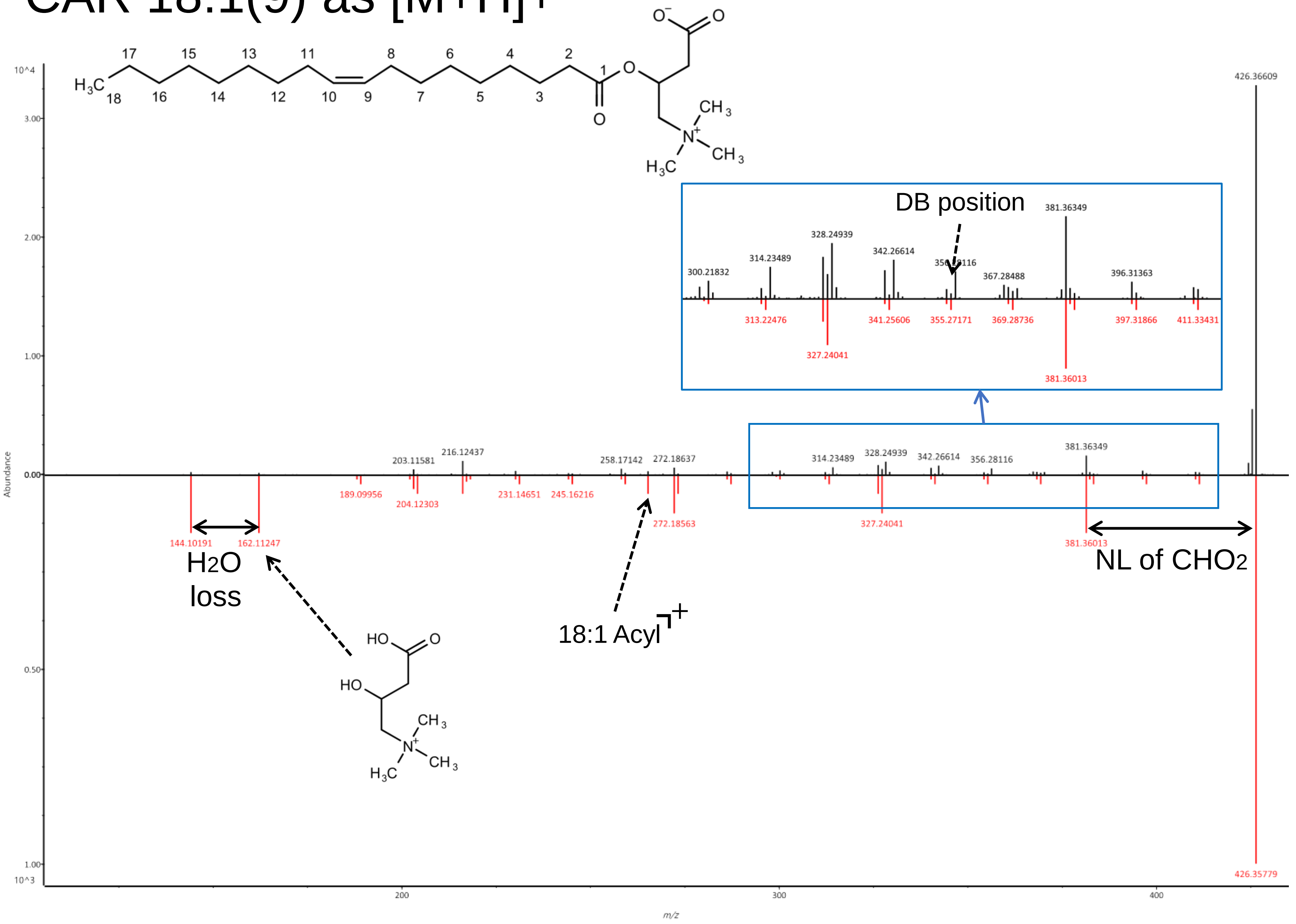

DGTS 18:2(9,12)/18:2(9,12) as [M+H]+

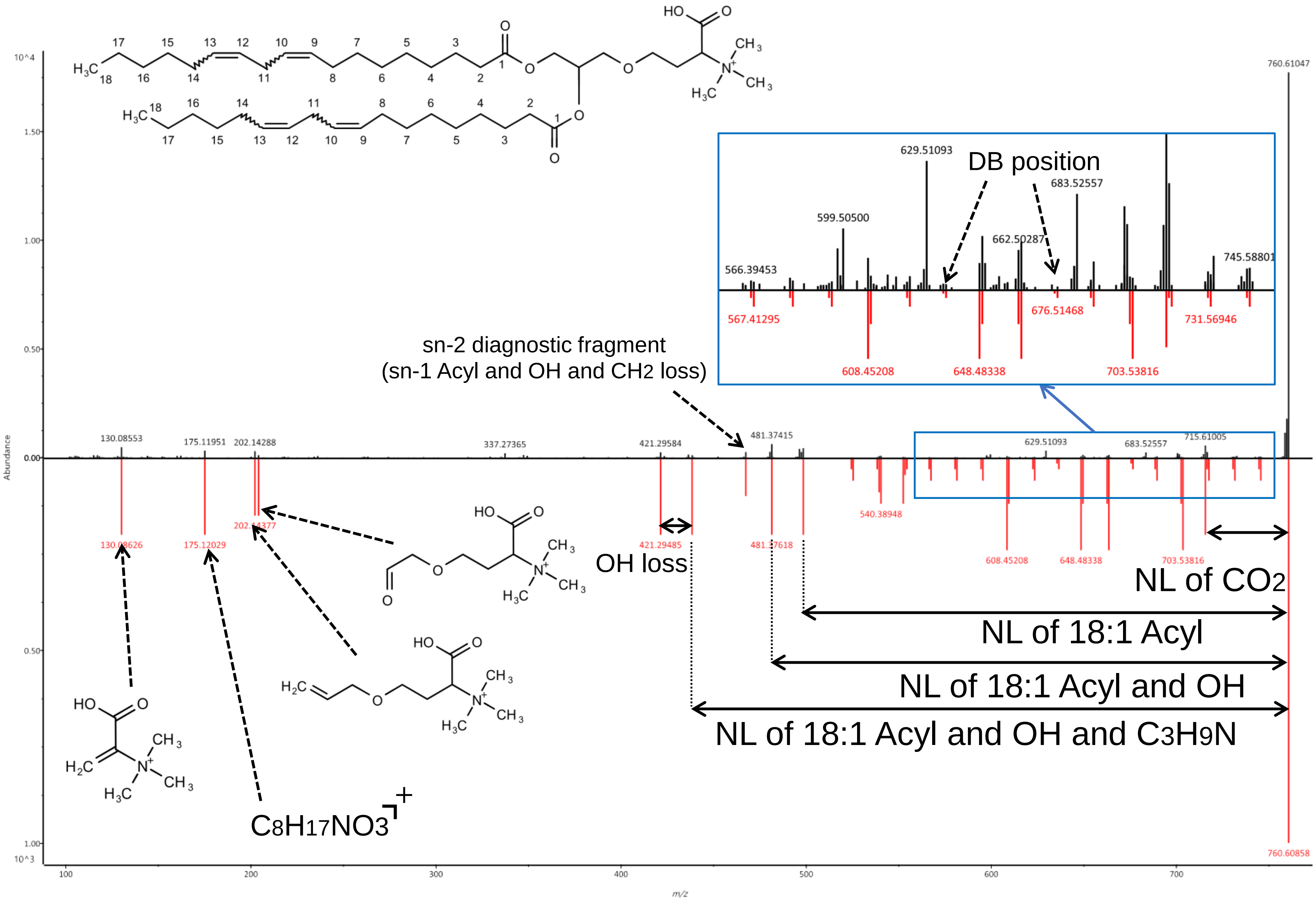

LDGTS 18:2(9,12) as [M+H]+

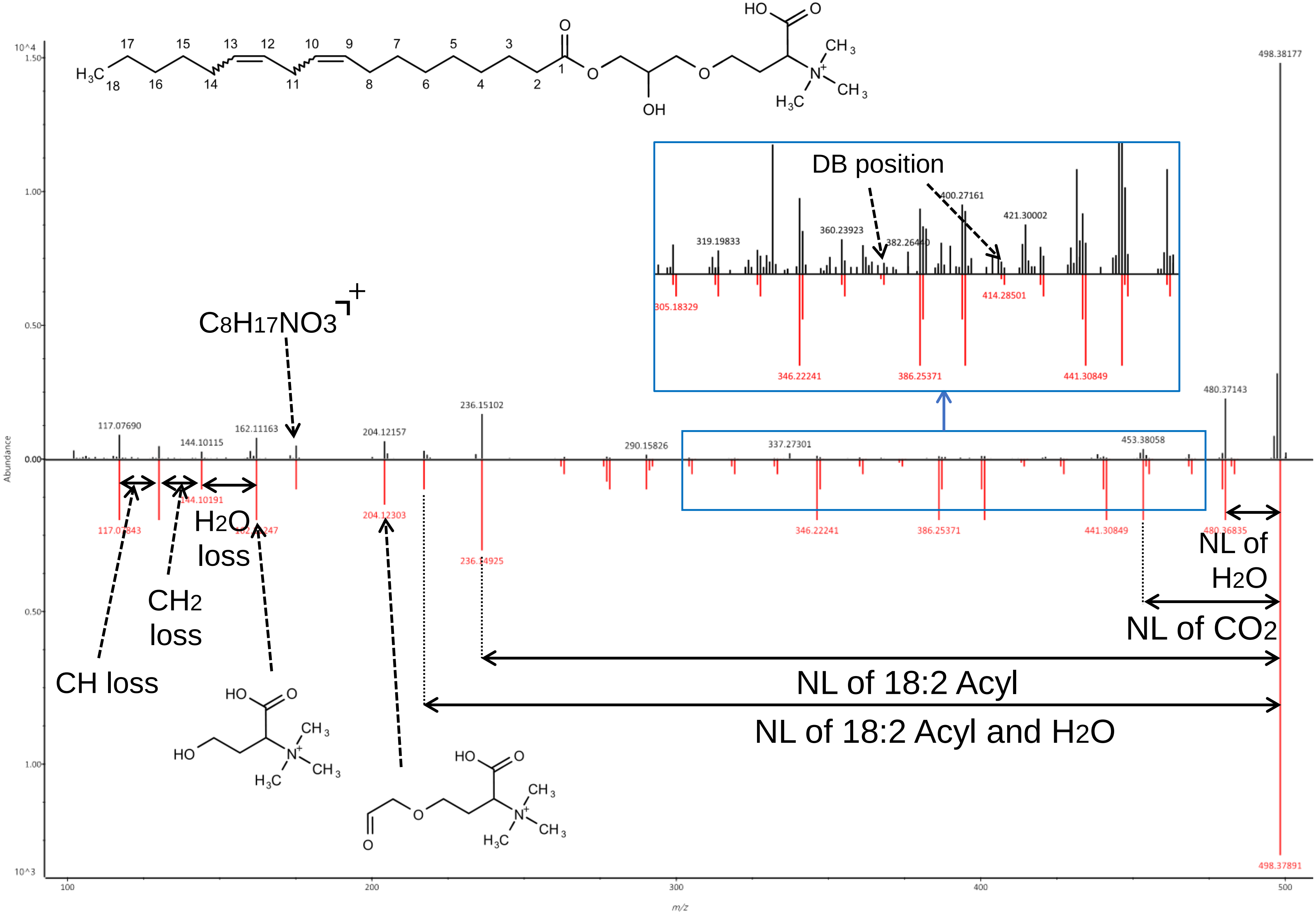

DGTA 16:0/20:5 as [M+H]+

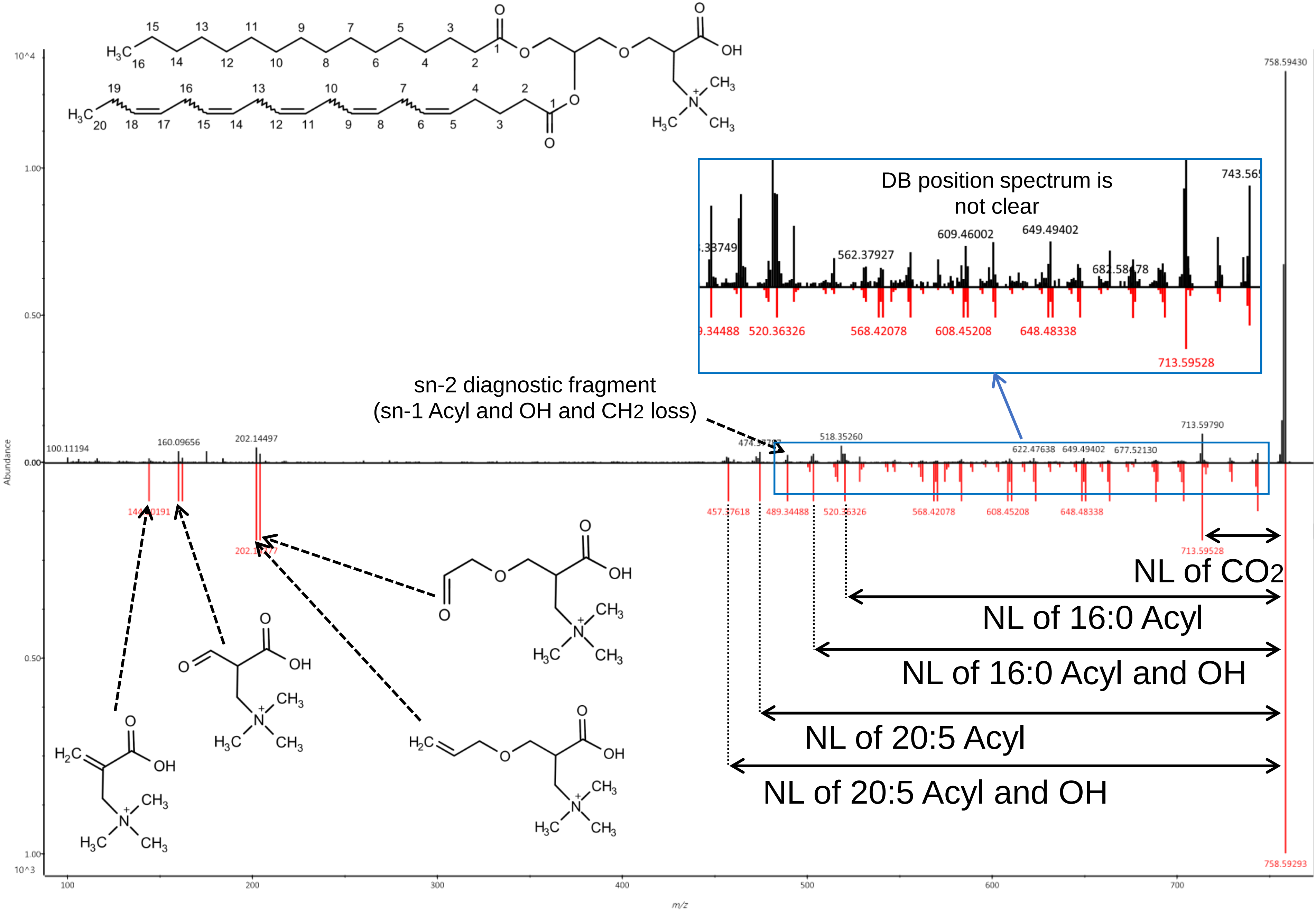

LDGTA 20:5 as [M+H]+

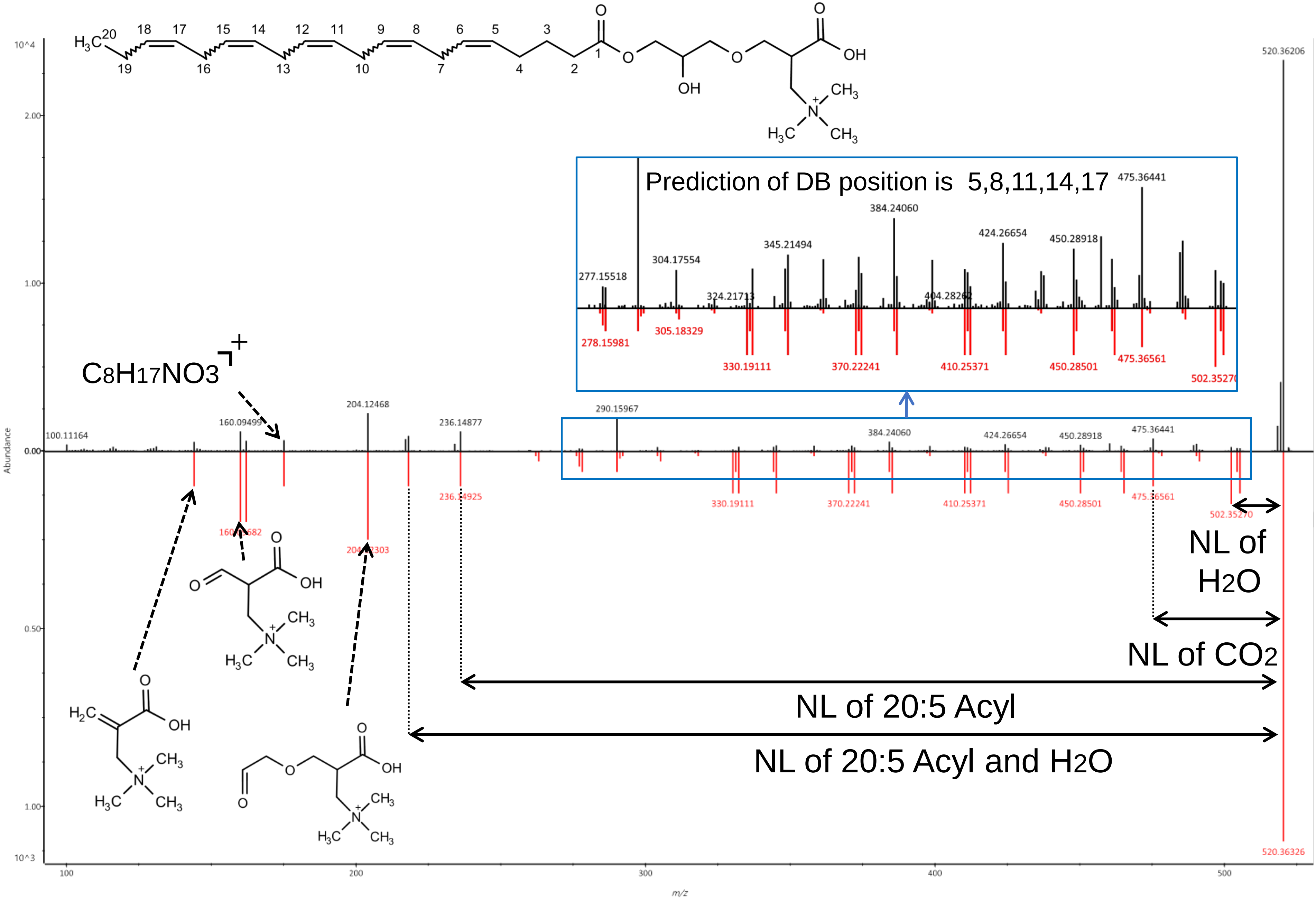

DMEDFAHFA 18:1(9)/18:0(9OH) as [M+H]+

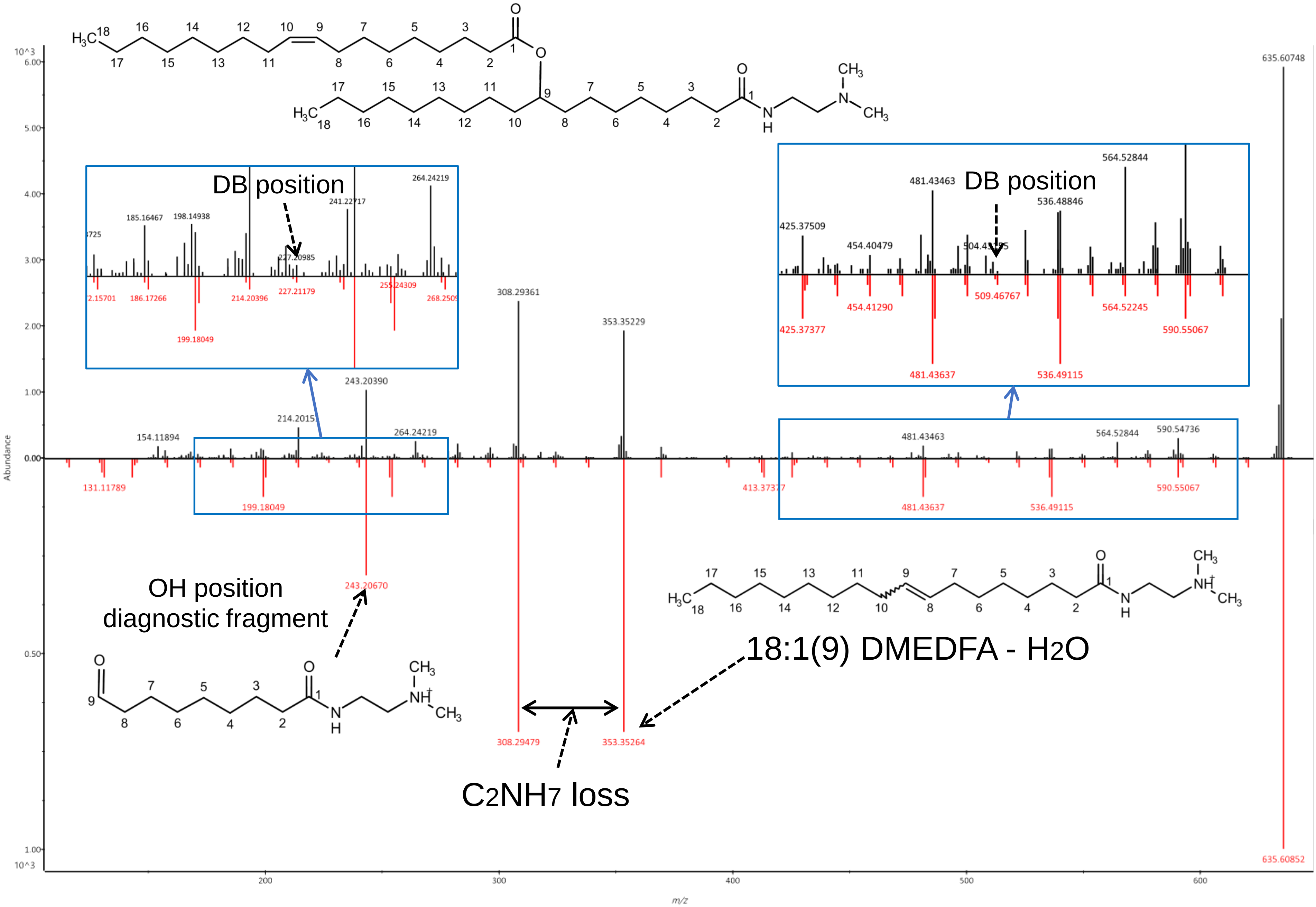

DMEDFA 18:1(9) as [M+H]+

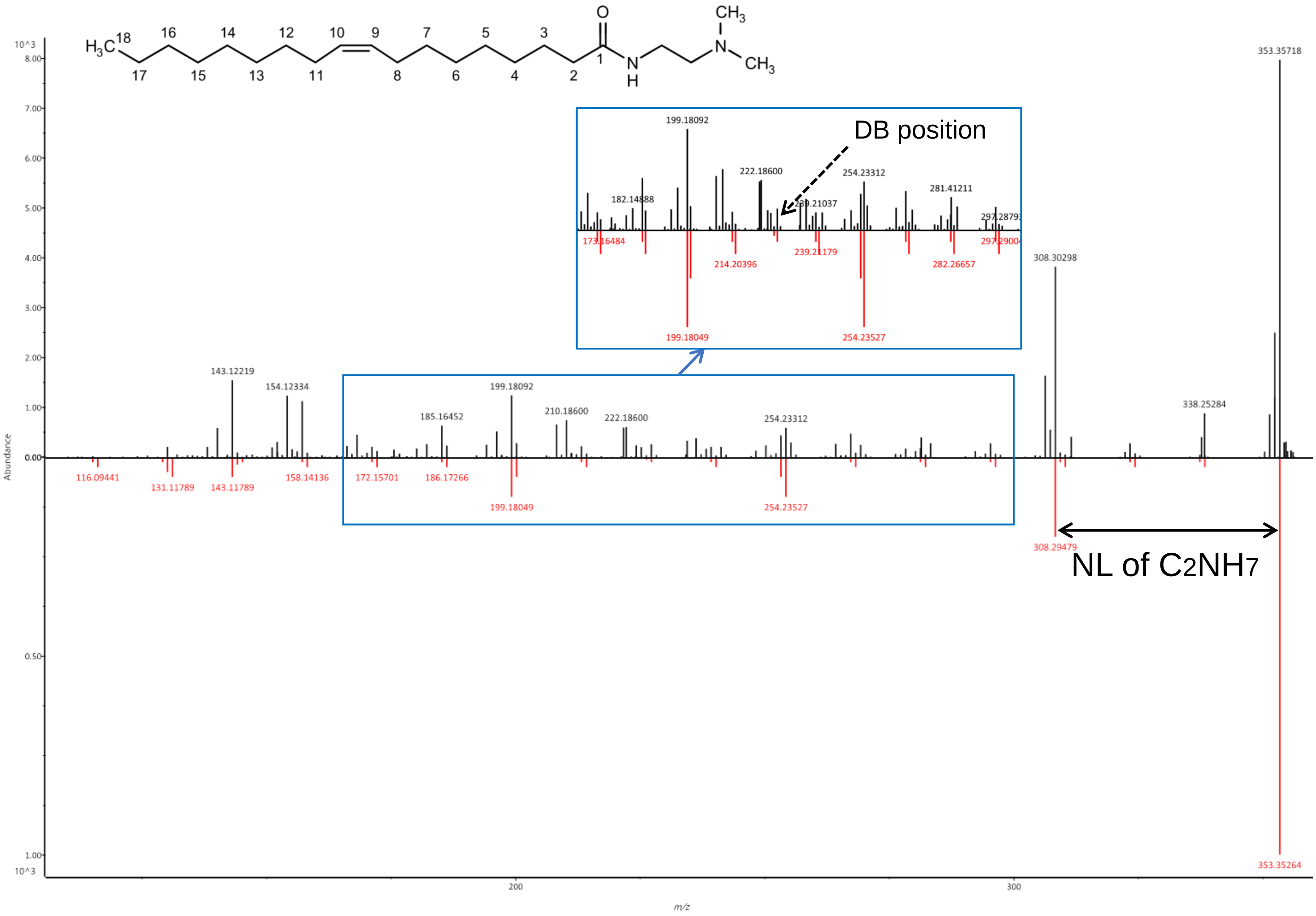

Supplementary Figure 5. Relationships between the kinetic energy 14 eV spectrum and the lipid structure in the sphingolipids category. The layout and the terms used are the same as those in Supplementary Figure 2. For the ceramide-AS type containing alpha-hydroxy fatty acid as the *N*-acyl chain, the OH position in the *N*-acyl chain was not characterized, while the OH positions in the sphingobase moiety were characterized.

SM 18:1(4)(1OH,3OH)/18:0 as [M+H]+

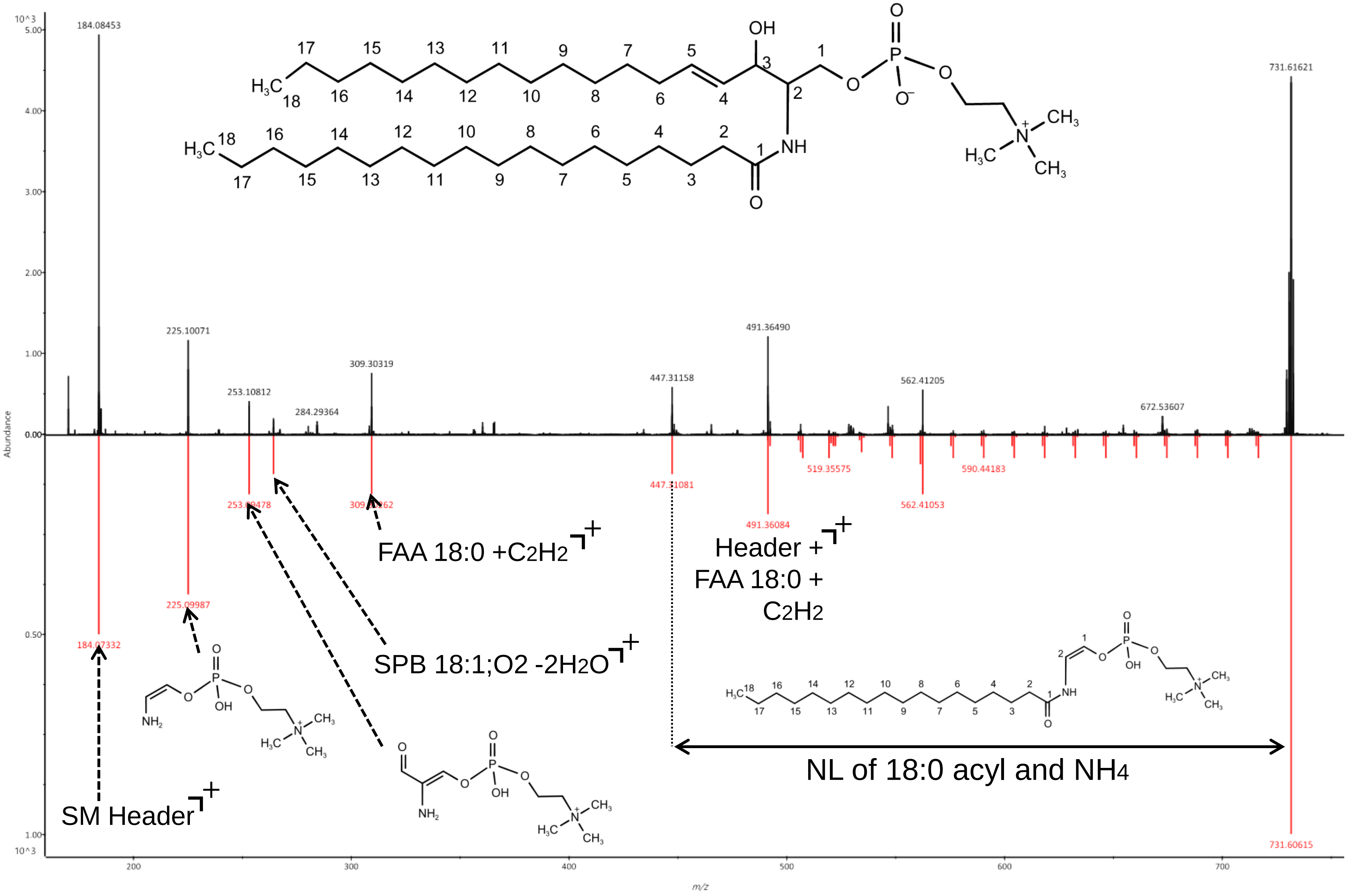

SM 18:1(4)(1OH,3OH)/18:0 as [M+Na]+

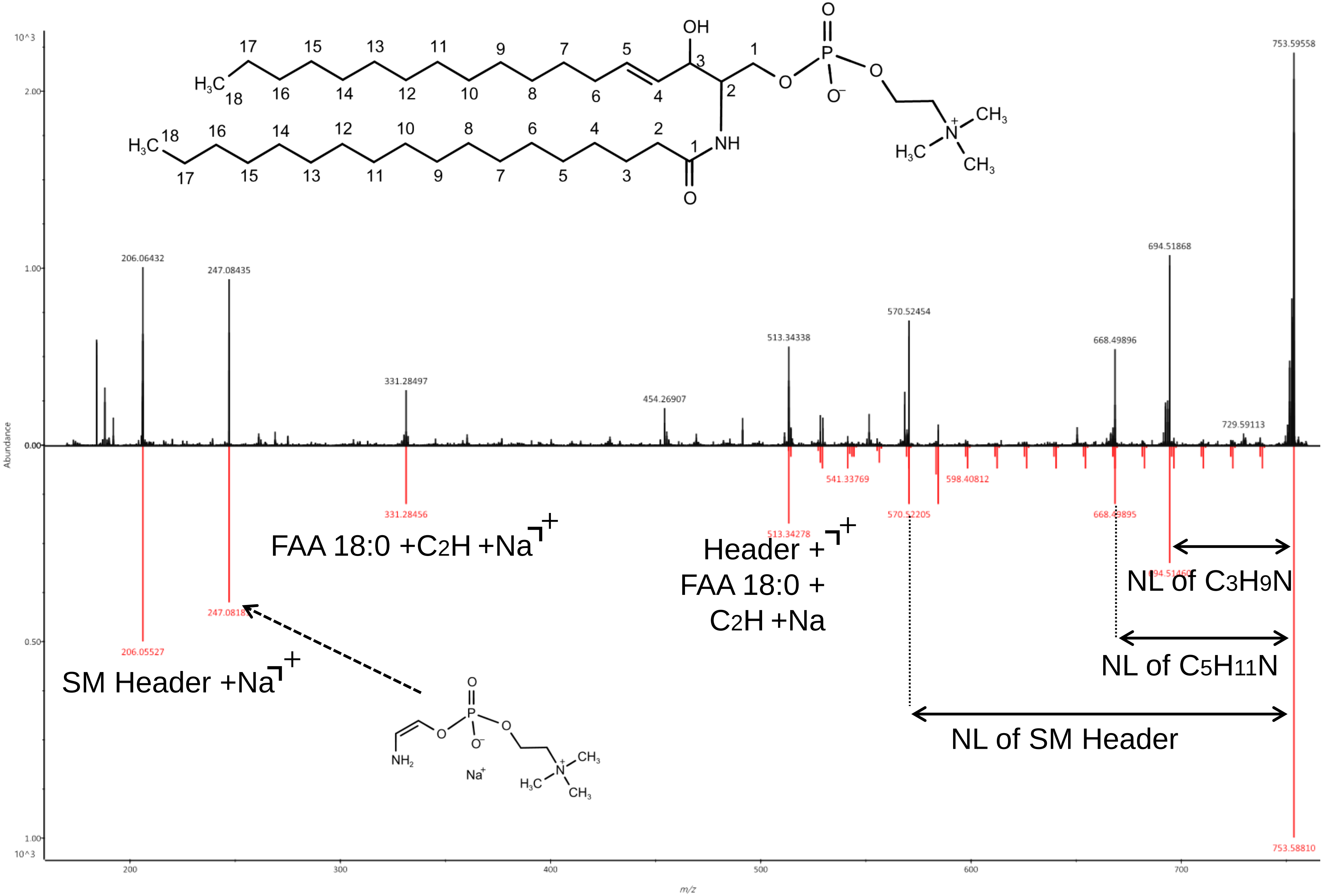

(Cer-NS) Cer 18:1(4)(1OH,3OH)/18:0 as [M+H]+

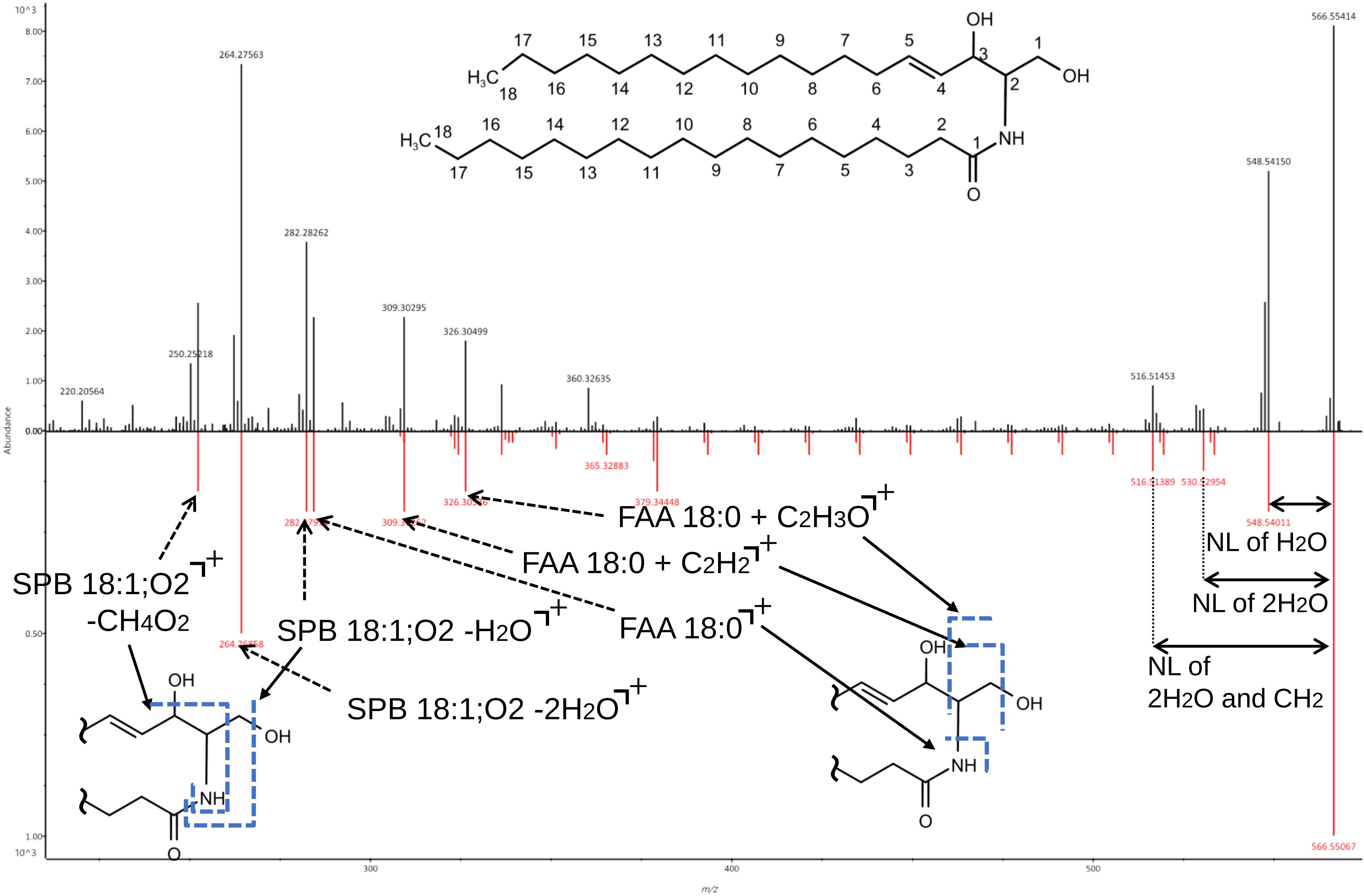

(Cer-NS) Cer 18:2(4,8)(1OH,3OH)/24:1(15) as [M+Na]+

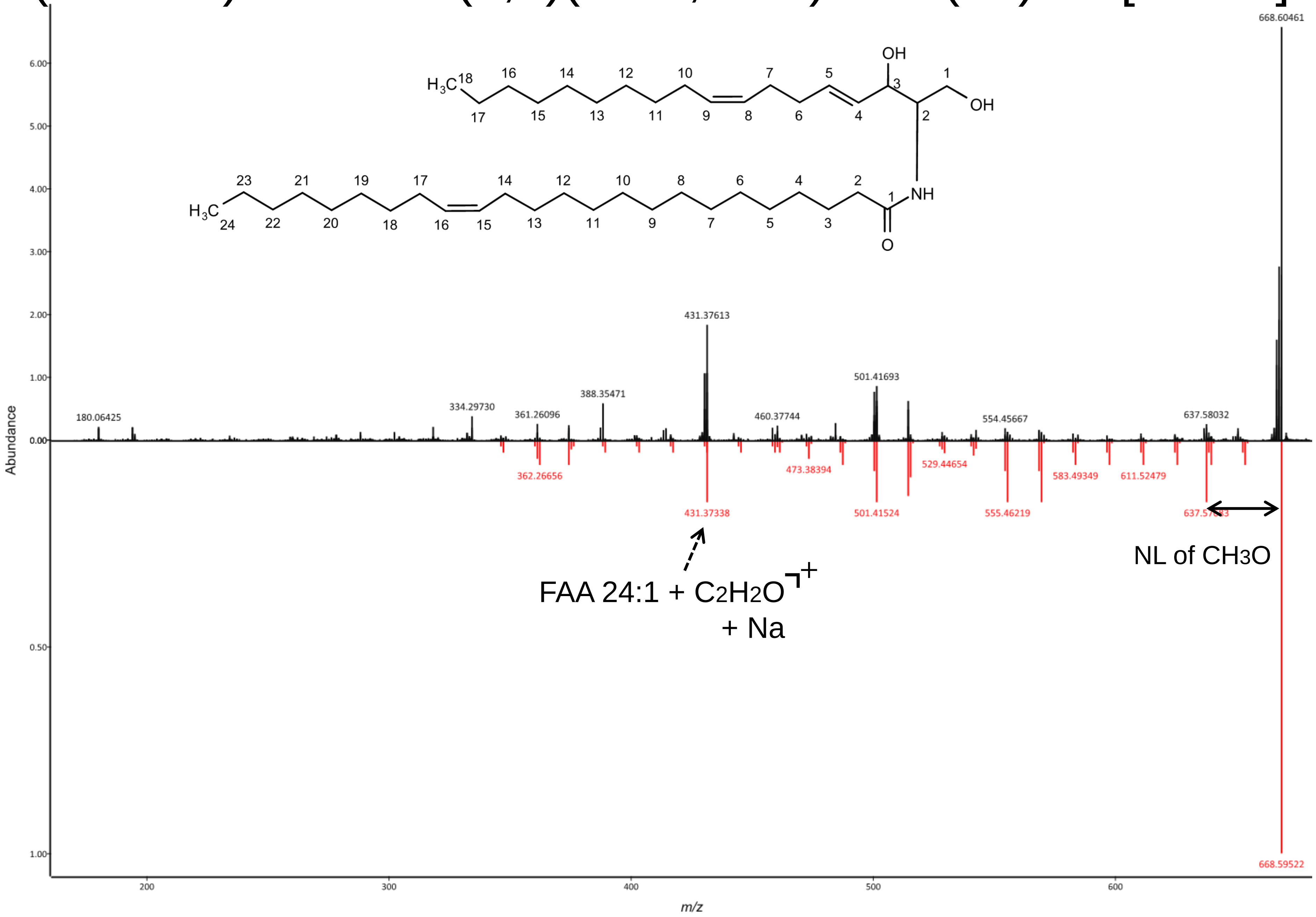

(Cer-NP) Cer 18:0(1OH,3OH,4OH)/24:0 as [M+H]+

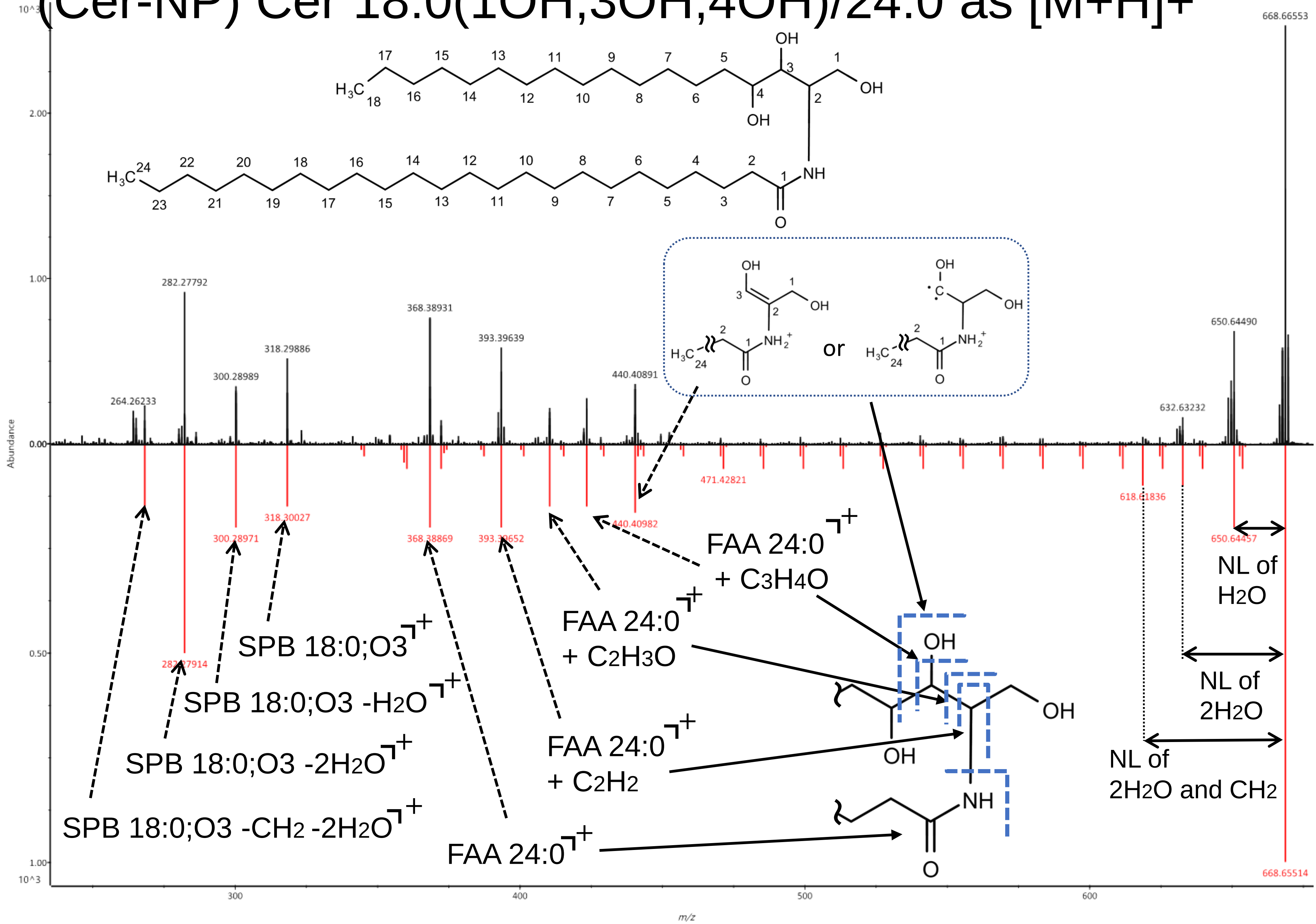

(Cer-AS) Cer 18:1(4)(1OH,3OH)/18:0(2OH) as [M+H]+

(Actual annotation name: Cer 18:1(4)(1OH,3OH)/18:0;O)

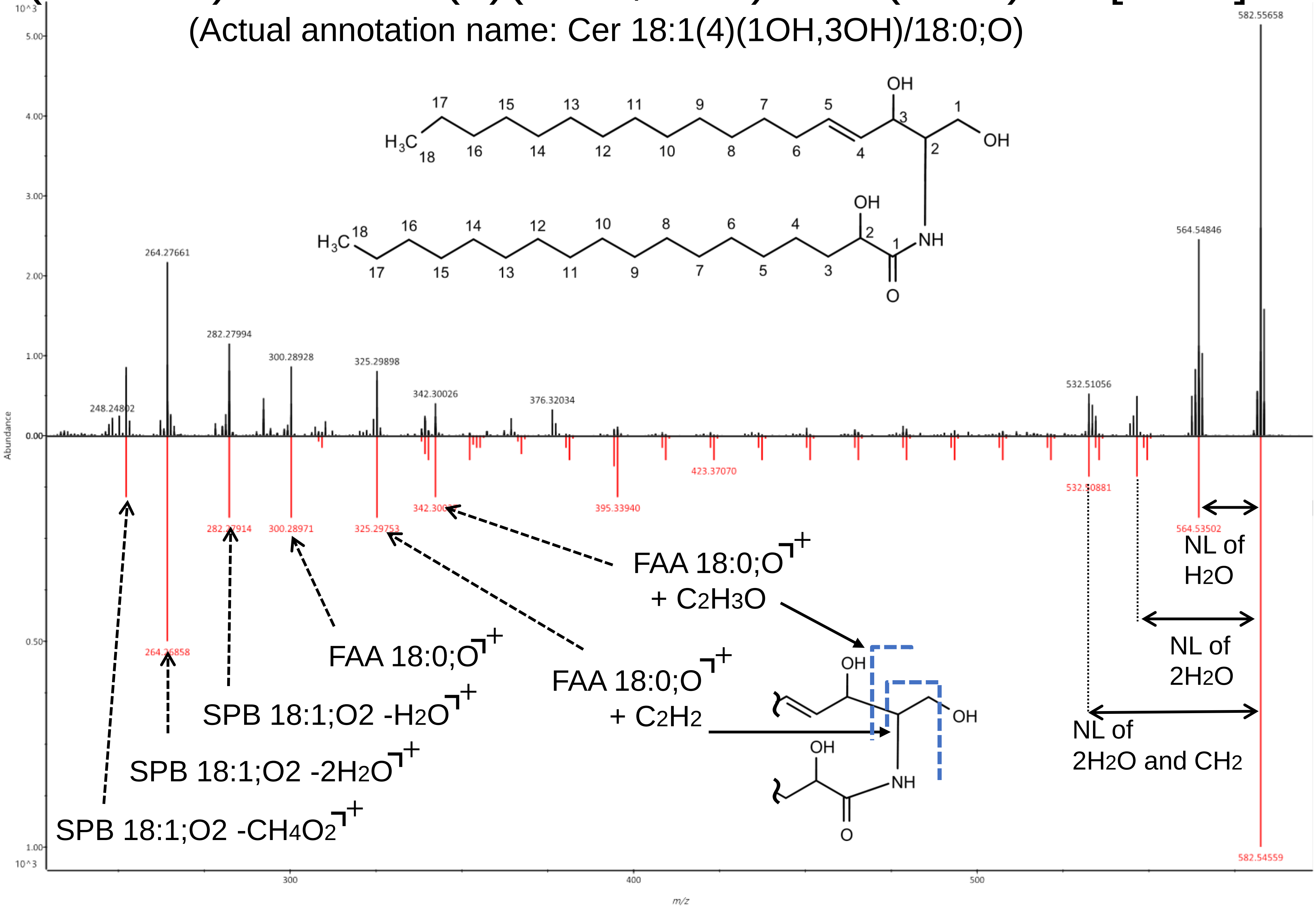

GalCer

(HexCer-NS) HexCer 18:1(4)(1OH,3OH)/18:0 as [M+H]+

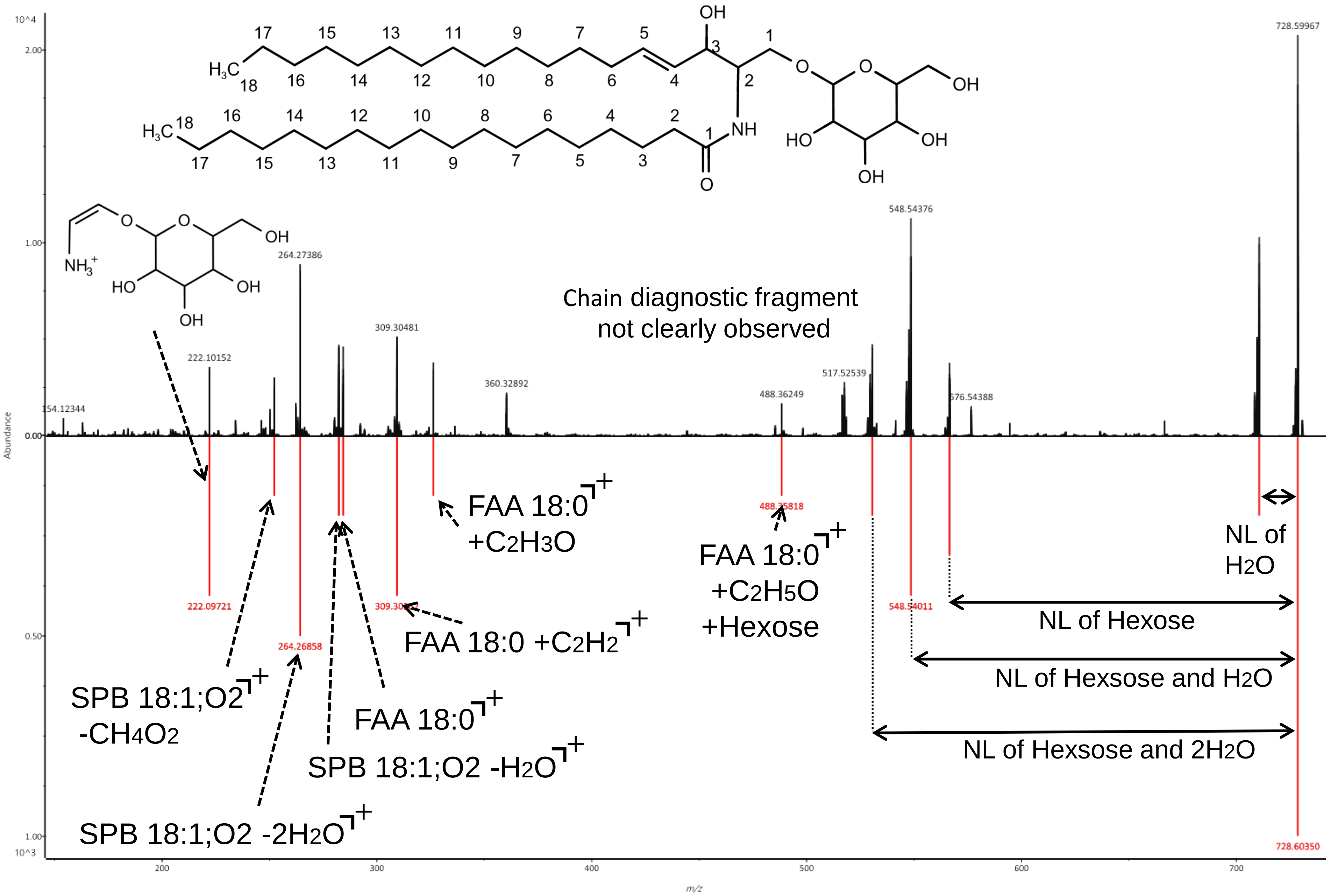

GalCer

(HexCer-NS) HexCer 18:1(4)(1OH,3OH)/18:0 as [M+Na]+

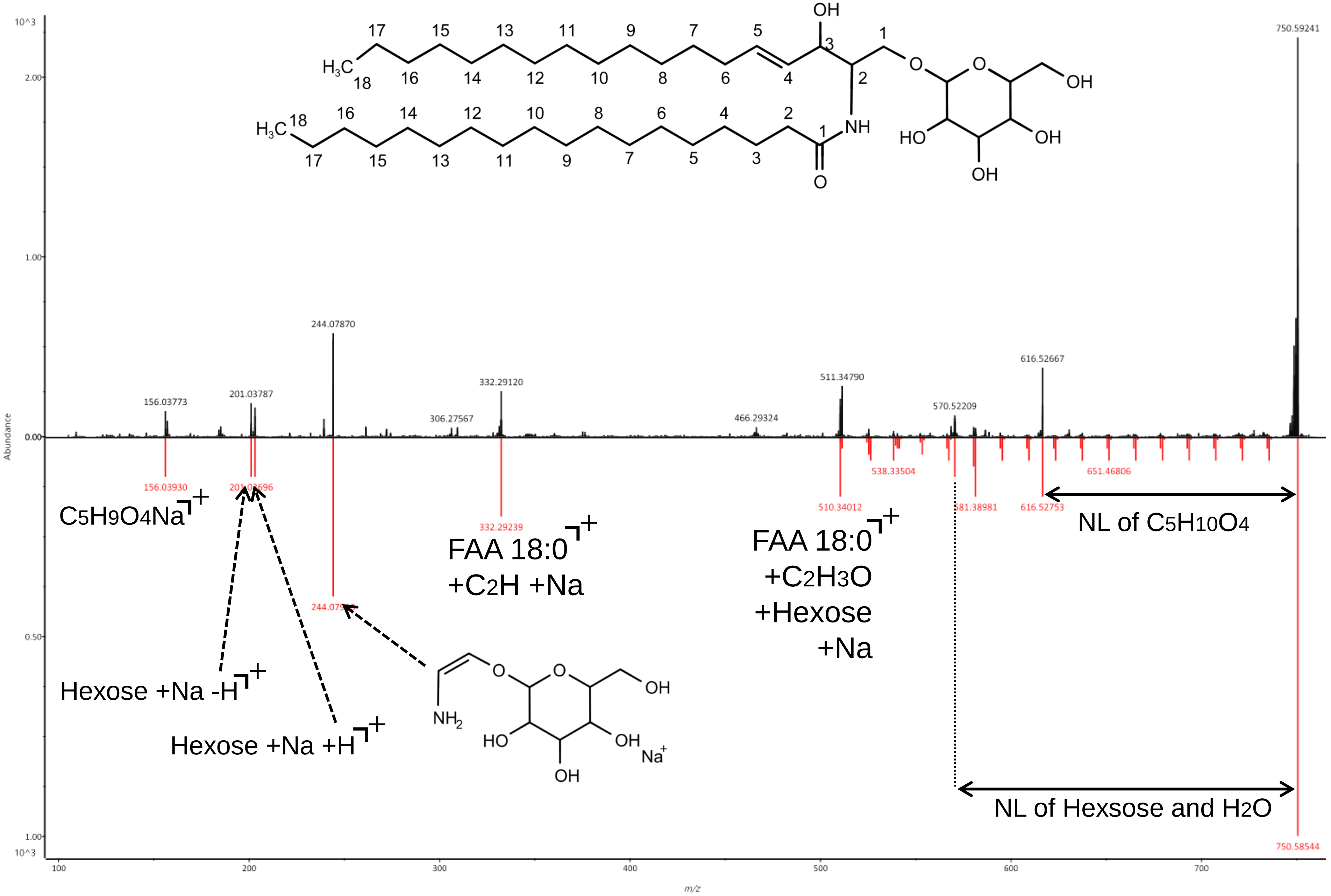

SHexCer 18:1(4)(1OH,3OH)/24:0 as [M+H]+

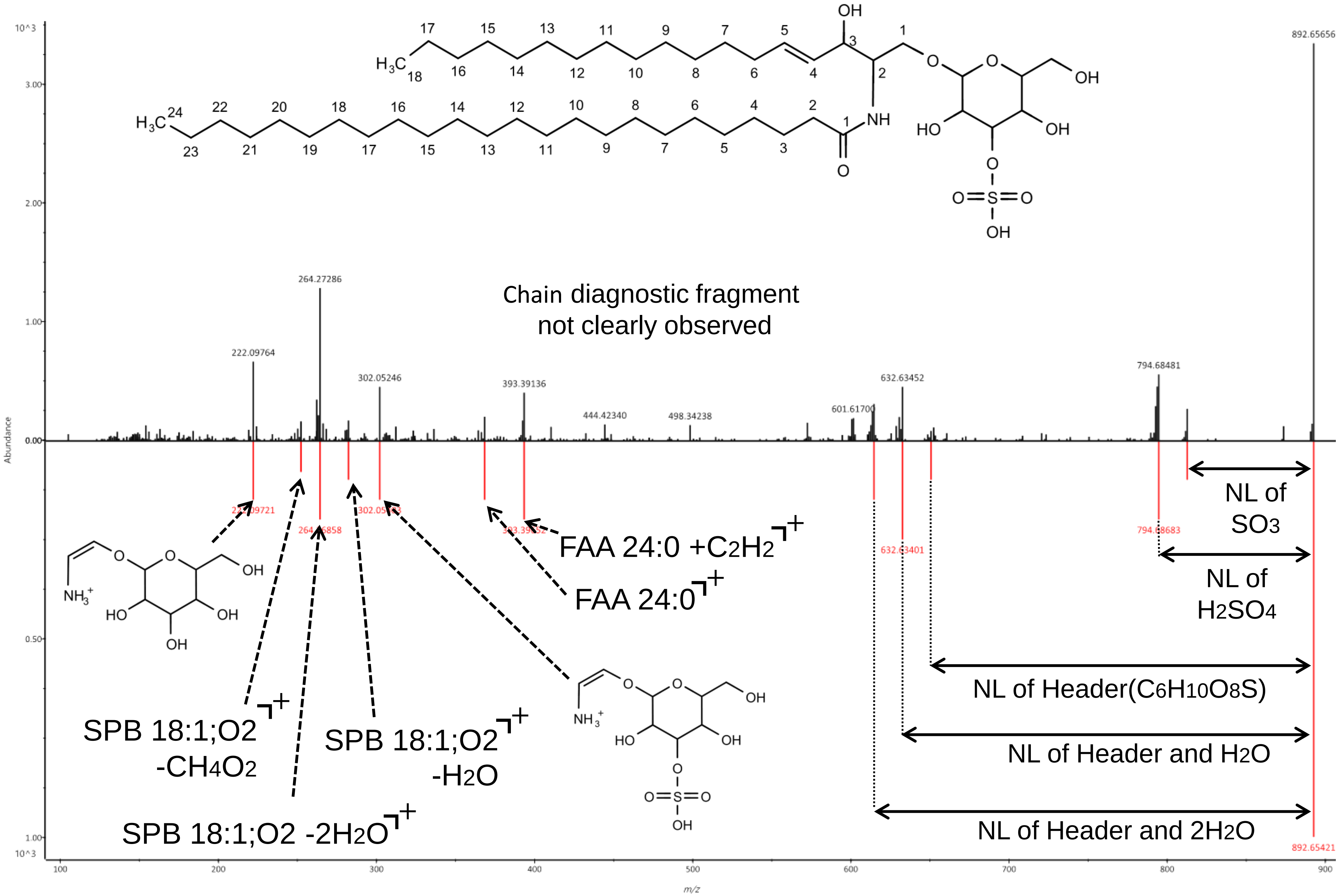

SHexCer 18:1(4)(1OH,3OH)/24:0 as [M+Na]+

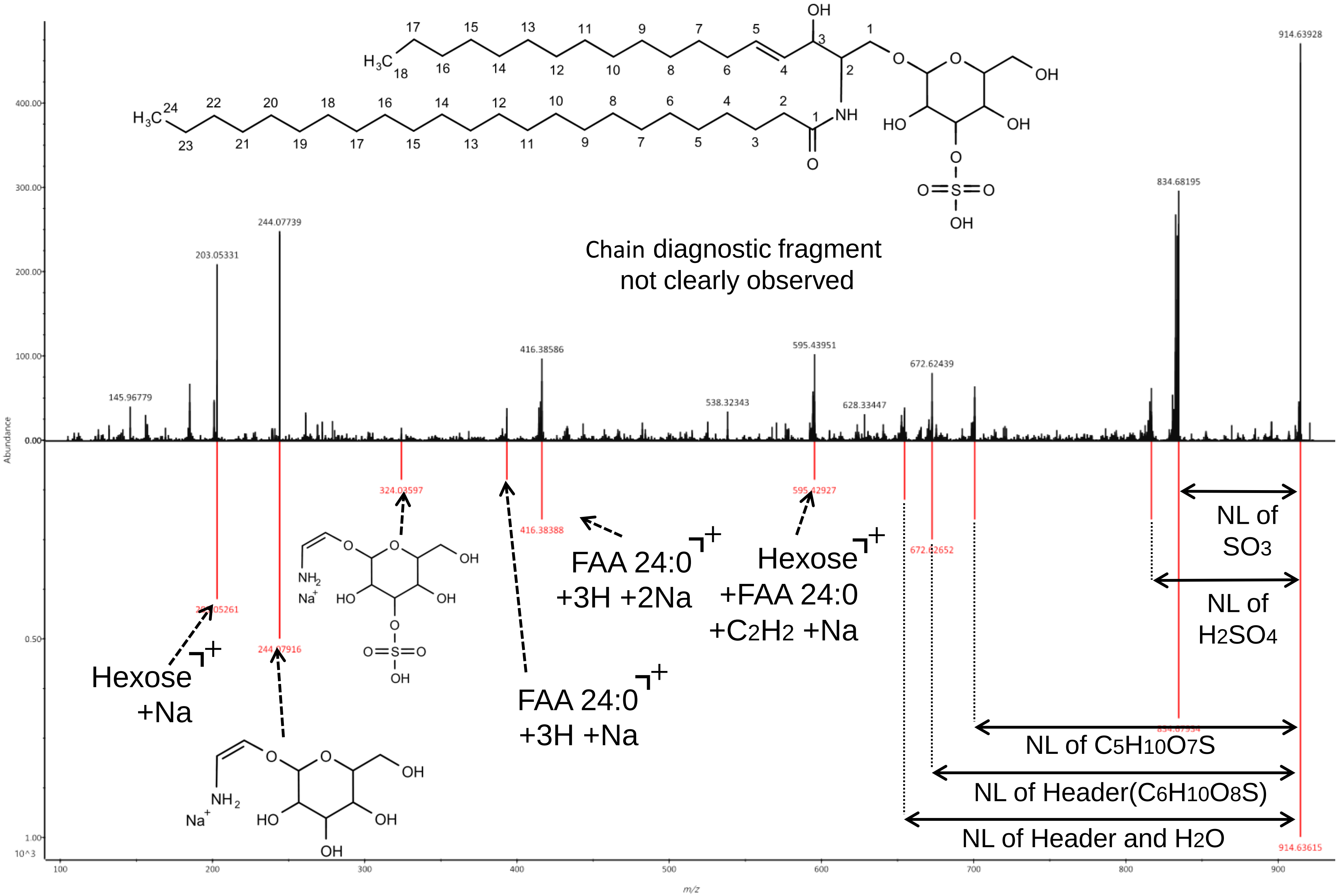

Supplementary Figure 6. Details of misannotations in the molecules of phosphatidylcholine (PC), phosphatidylinositol (PI), and triacylglycerol (TG). (a) The MS/MS spectrum of the protonated form of PC-d5 17:0/16:1(9). The spectra of the entire (bottom panel) and zoomed regions (top panel) are shown, where the diagnostic ions of *m/z* 466.3246 and *m/z* 482.3559 that determine the *sn*-position for *sn1*-17:0 and *sn1*-16:1, respectively, are described. The annotation was incorrect due to a lower abundance of the ion related to sn1-17:0 than that of sn1-16:1. (b) The MS/MS spectrum of the protonated form of PC-d5 17:0/ 22:4(7,10,13,16) was correctly annotated, although the contamination of sn1-22:4 related ion existed. The spectra of the entire (bottom panel) and zoomed regions (top panel) are shown, where the diagnostic ions of *m/z* 544.3715 and *m/z* 482.3559 that determine the *sn*-position for *sn1*-17:0 and *sn1*-22:4, respectively, are described. (c) MS/MS spectrum of the ammonium adduct form of PI 18:1(9)/18:1(9). The upper and lower panels show the experimental- and *in silico* MS/MS spectra. The V-shape pattern of the product ion spectrum determining the C=C-position as C9 is described. (d) MS/MS spectrum of the ammonium adduct form of PI-d5 17:0/16:1(9). The correct annotation is PI-d5 17:0/16:1(9), while the MS-DIAL program annotated the spectrum as PI-d5 17:0/16:1(7) due to the absence of a C=C high peak for 16:1(9). (e) MS/MS spectrum of the ammonium adduct form of TG 18:1(9)_18:1(9)_18:1(9), where the V-shape pattern for 18:1(9) is also described. (f) MS/MS spectrum of the ammonium adduct form of TG-d5 16:0_16:0_17:1(10). The spectrum was misannotated because the local correlation value for 17:1(5) was higher than that of 17:1(9). The V-shape patterns for 17:1(5) and 17:1(9) are described while the in silico MS/MS spectrum of TG-d5 16:0_16:0_17:1(10) is described in the lower panel.

a The original annotation was PC-d5 16:1(9)/17:0, while the correct annotation should be PC-d5 17:0/16:1(9)

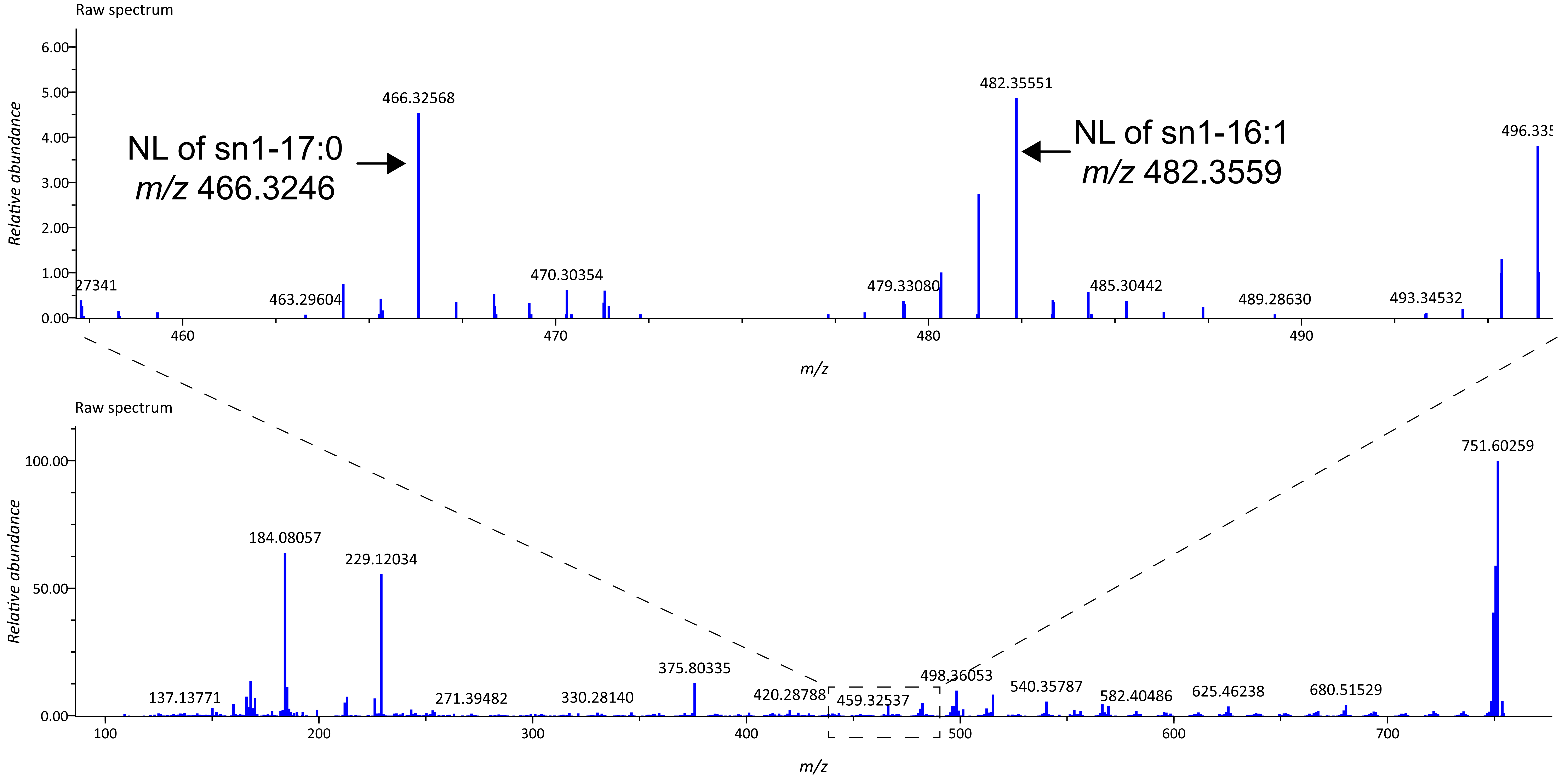

b The annotation of PC-d5 17:0/22:4(7,10,13,16) was correct, but sn1-16:1 exists in the spectrum.

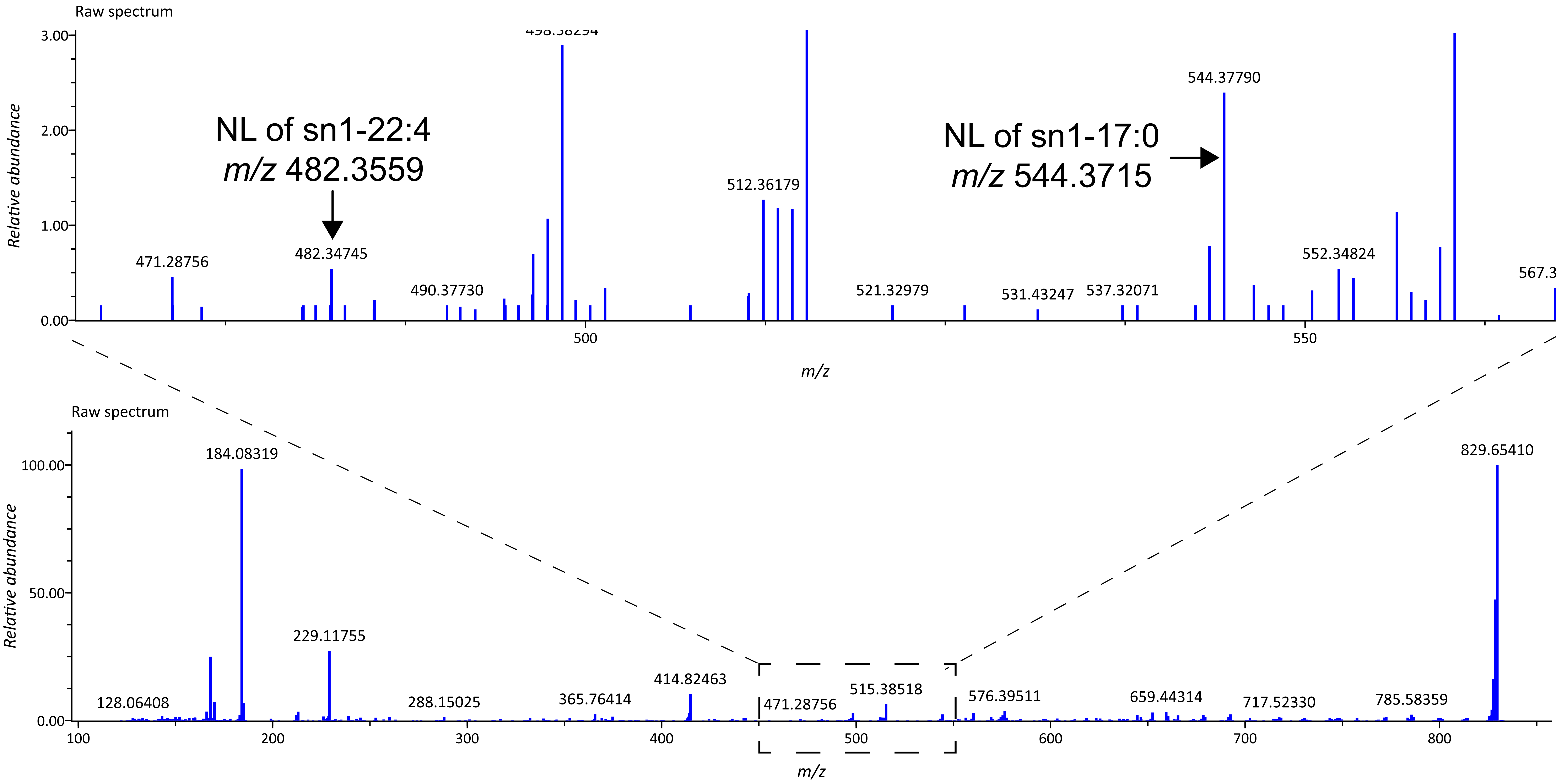

c PI 18:1(9)/18:1(9): the case of correct C=C position annotation in PI.

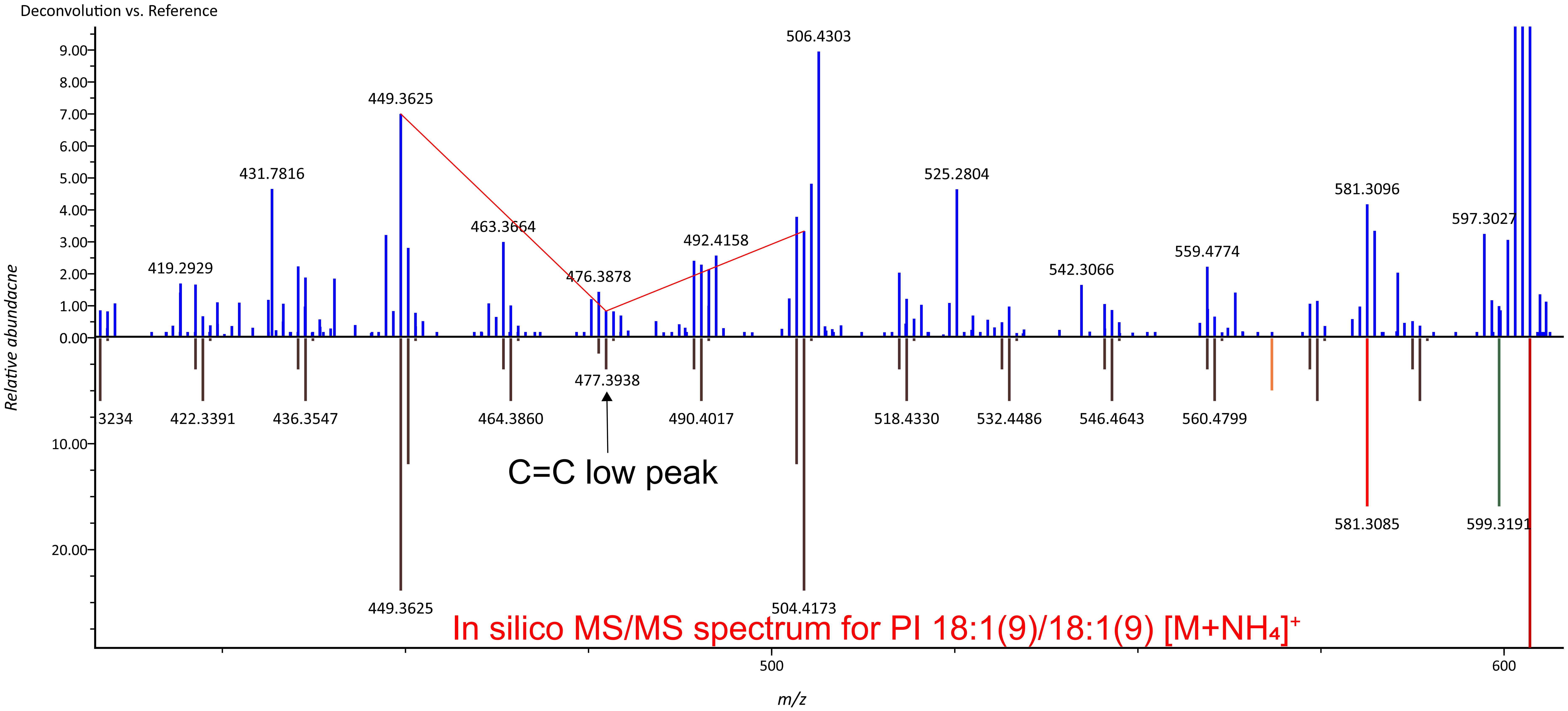

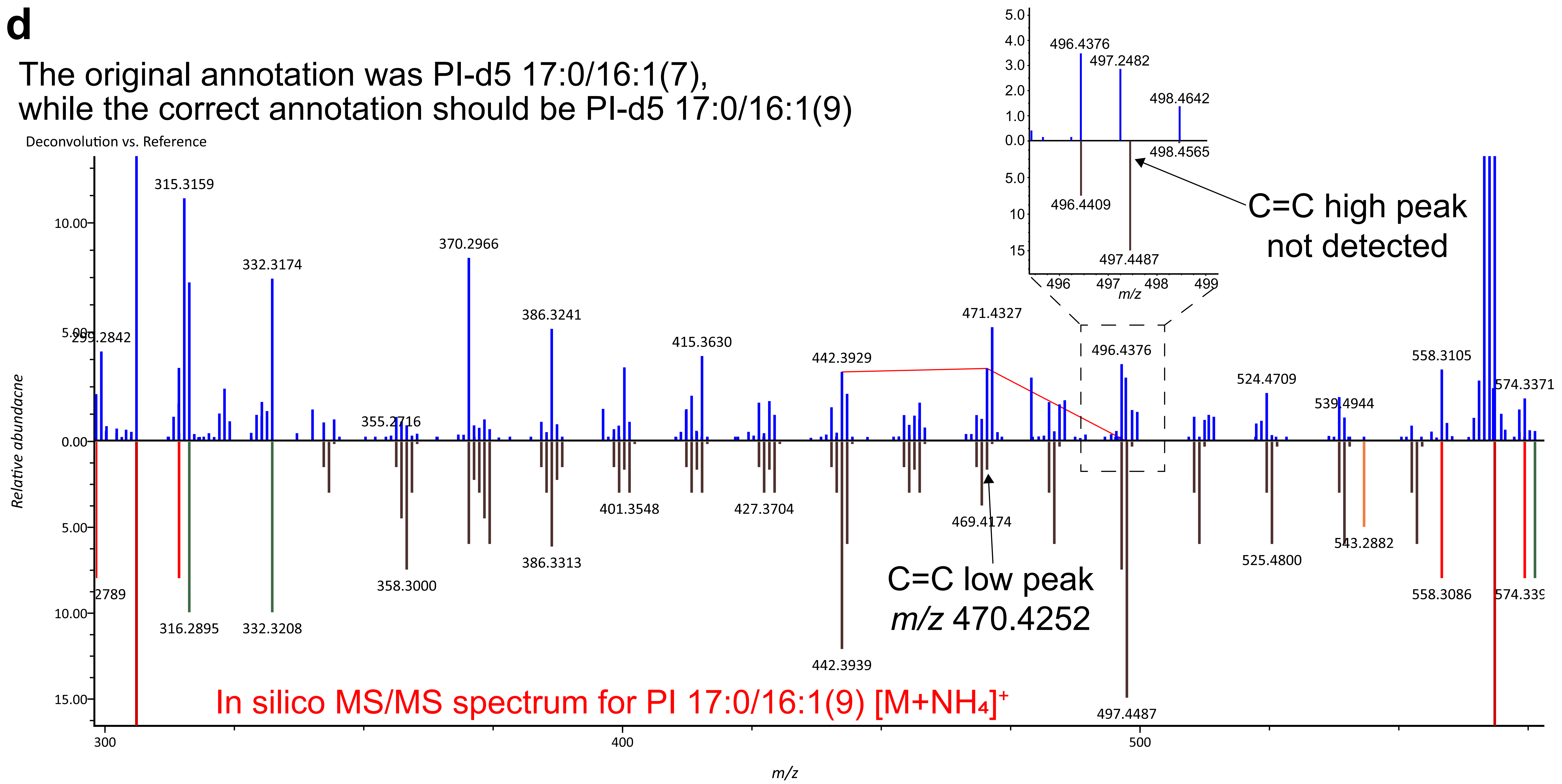
d
The original annotation was PI-d5 17:0/16:1(7),
while the correct annotation should be PI-d5 17:0/16:1(9)
Deconvolution vs. Reference
Relative abundacne
m/z
315.3159
332.3174
370.2966
386.3241
299.2842
415.3630
442.3929
471.4327
496.4376
524.4709
539.4944
558.3105
574.3371
355.2716
401.3548
427.3704
469.4174
386.3313
358.3000
316.2895
332.3208
442.3939
497.4487
525.4800
543.2882
558.3086
2789
574.339
C=C low peak
m/z 470.4252
496.4376
497.2482
498.4642
498.4565
496.4409
497.4487
C=C high peak
not detected
In silico MS/MS spectrum for PI 17:0/16:1(9) [M+NH4]+

e

TG 18:1(9)_18:1(9)_18:1(9): the case of correct C=C position annotation in TG.

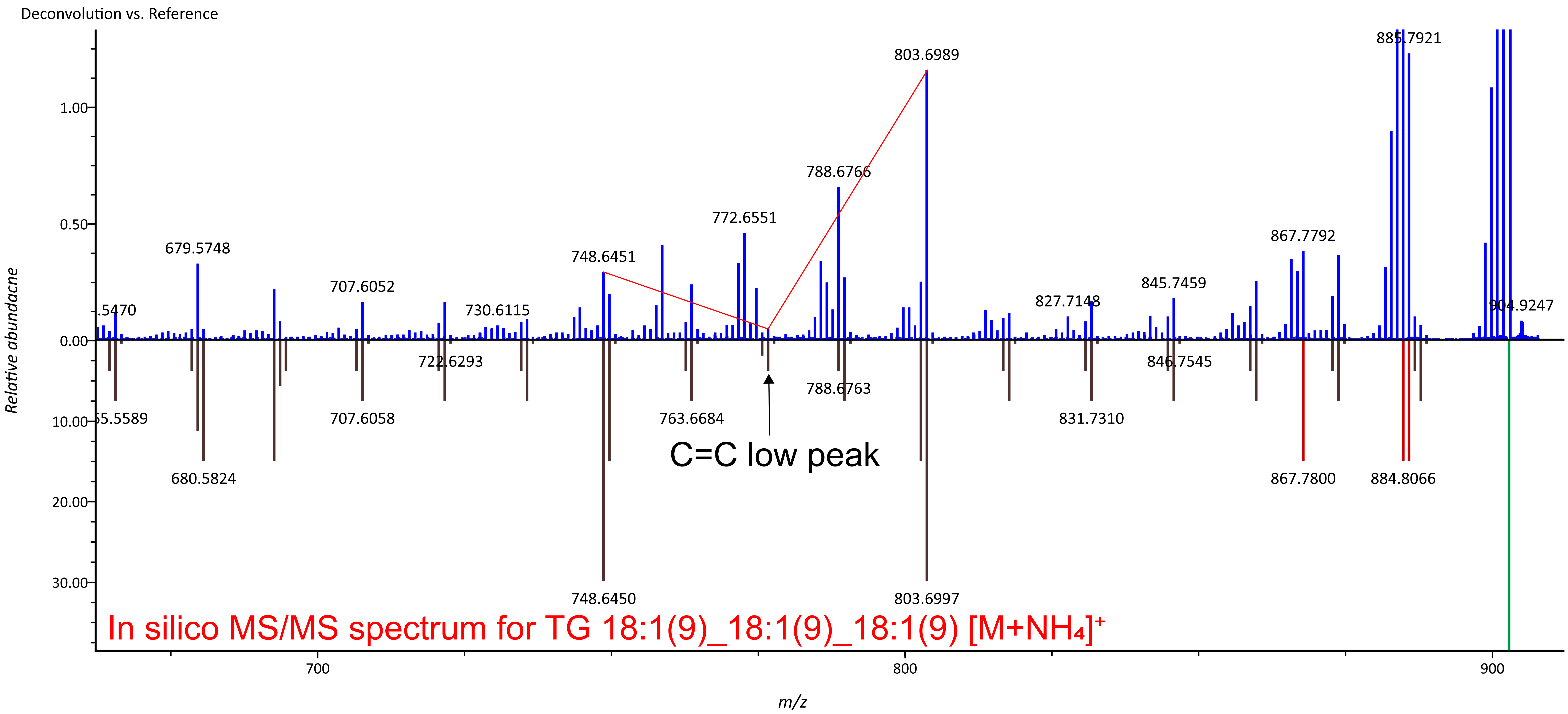

f The original annotation was TG-d5 16:0_16:0_17:1(5), while the correct annotation should be TG-d5 16:0_16:0_17:1(10)

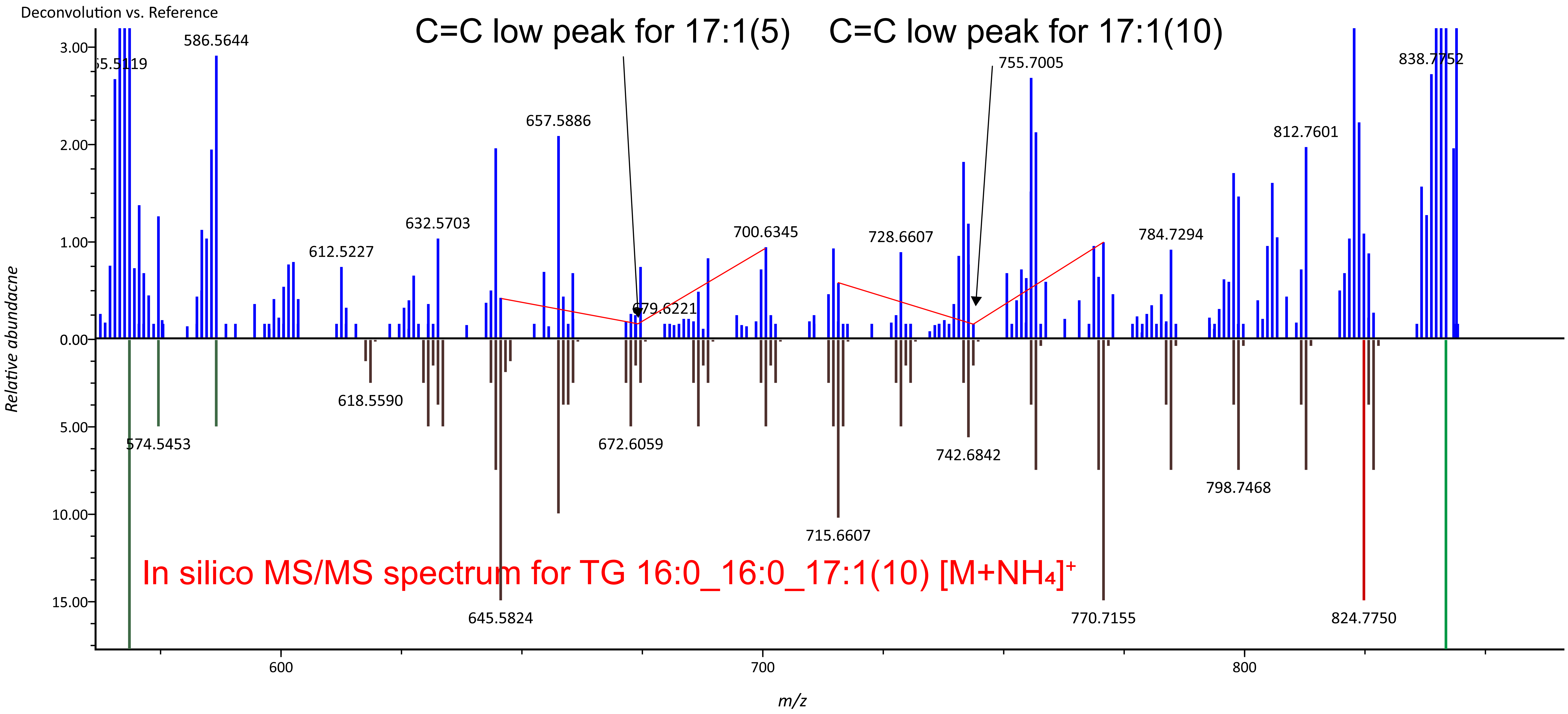

Supplementary Figure 7. Data set construction for MS-DIAL evaluation using lipid isomers, PC 16:0/18:1(9), PC 16:0/18:1(11), and PC 18:1(9)/16:0. (a) Extracted ion chromatograms (EICs) of PC 16:0/18:1(9Z), PC 16:0/18:1(11Z), and PC 18:1(9Z)/16:0, abbreviated as POPC, PVPC, and OPPC, respectively, in the LC-MS method used in this study. (b) MS/MS patterns of PC 16:0/18:1(9), PC 18:1(9)/16:0, and PC 16:0/18:1(11). (c) Calibration curves for PC 16:0/18:1(9), PC 18:1(9)/16:0, and PC 16:0/18:1(11). The calibration curves were constructed for each lipid isomer using MS1 peak height and normalized MS1 peak height by the internal standard of PC 15:0_18:1(d7). The calibration curves were also constructed using MS2 peak height of *sn*- and C=C position diagnostic ions. (d) Relationship between concentration ratio and MS2 peak height ratio in PC mixture. The mixture of POPC and PVPC and the mixture of POPC and OPPC were prepared. The calibration curves were constructed by the MS2 peak height ratios of H-loss diagnostic fragment ions and *sn1*-CH2 loss diagnostic fragment ions which are used to distinguish the C=C- and *sn*-positions, respectively. The calibration curve using the OAD2 fragment ions generated by OAD-MS/MS technique was also constructed. The definition of OAD2 is described in **Supplementary Figure 7f**. (e) Isomer ratio estimations for *Δ*9/*Δ*11 and *sn1*-16:0/*sn1*-18:1 in mouse brain and human plasma using EAD and OAD. The MS2 peak heights of MS/MS chromatograms were used to calculate the intensity ratios. The intensity ratio was then normalized by the calibration curve created in **Supplementary Figure 7d**. (f) OAD-MS/MS fragmentation patterns of PC 16:0/18:1(9) and PC 16:0/18:1(11). The fragment ions of *m/z* 622.4442 and *m/z* 650.4755 were defined as "OAD15" which is the specific fragment ion to determine the C=C position of PC 16:0/18:1(9) and PC 16:0/18:1(11), respectively. The fragment ions of *m/z* 664.4548 and *m/z* 692.4861 were defined as "OAD2" which is also the specific fragment ion to determine the C=C position of PC 16:0/18:1(9) and PC 16:0/18:1(11), respectively. The terminology of OAD2 and OAD15 follows the definition of the original publication using OAD-MS/MS technique for lipid profiling. (g) Calibration curves using MS2 peak height of C=C position diagnostic ions based on OAD-MS/MS for PC 16:0/18:1(9). (h) OAD-MS/MS in the mixture of PC isomers, plasma lipid extract, and brain lipid extract.

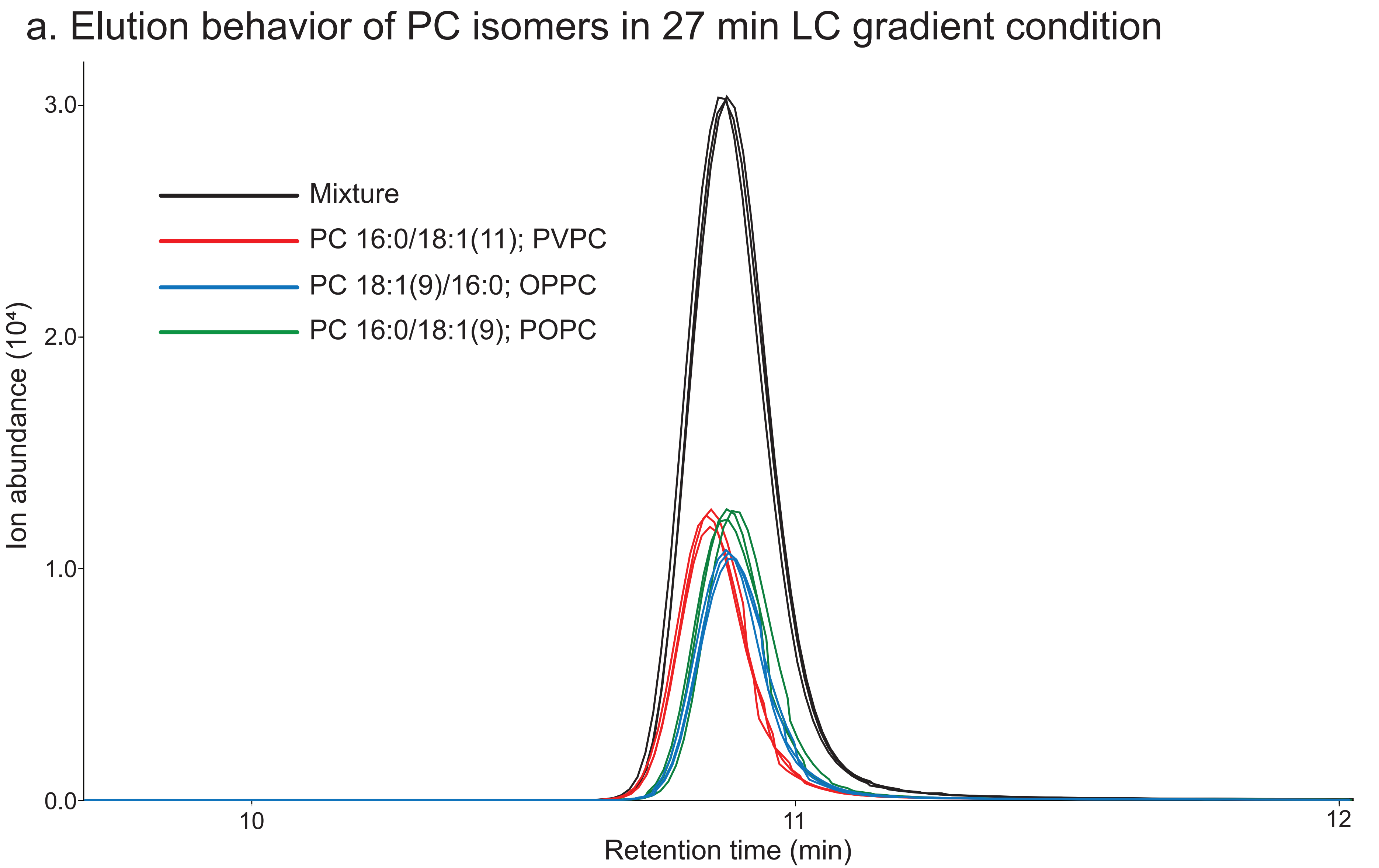
a. Elution behavior of PC isomers in 27 min LC gradient condition
Mixture
PC 16:0/18:1(11); PVPC
PC 18:1(9)/16:0; OPPC
PC 16:0/18:1(9); POPC
Ion abundance (10⁴)
3.0
2.0
1.0
0.0
10
11
12
Retention time (min)

b. Product ion spectral patterns of PC 16:0/18:1(9), PC 18:1(9)/16:0, and PC 16:0/18:1(11)

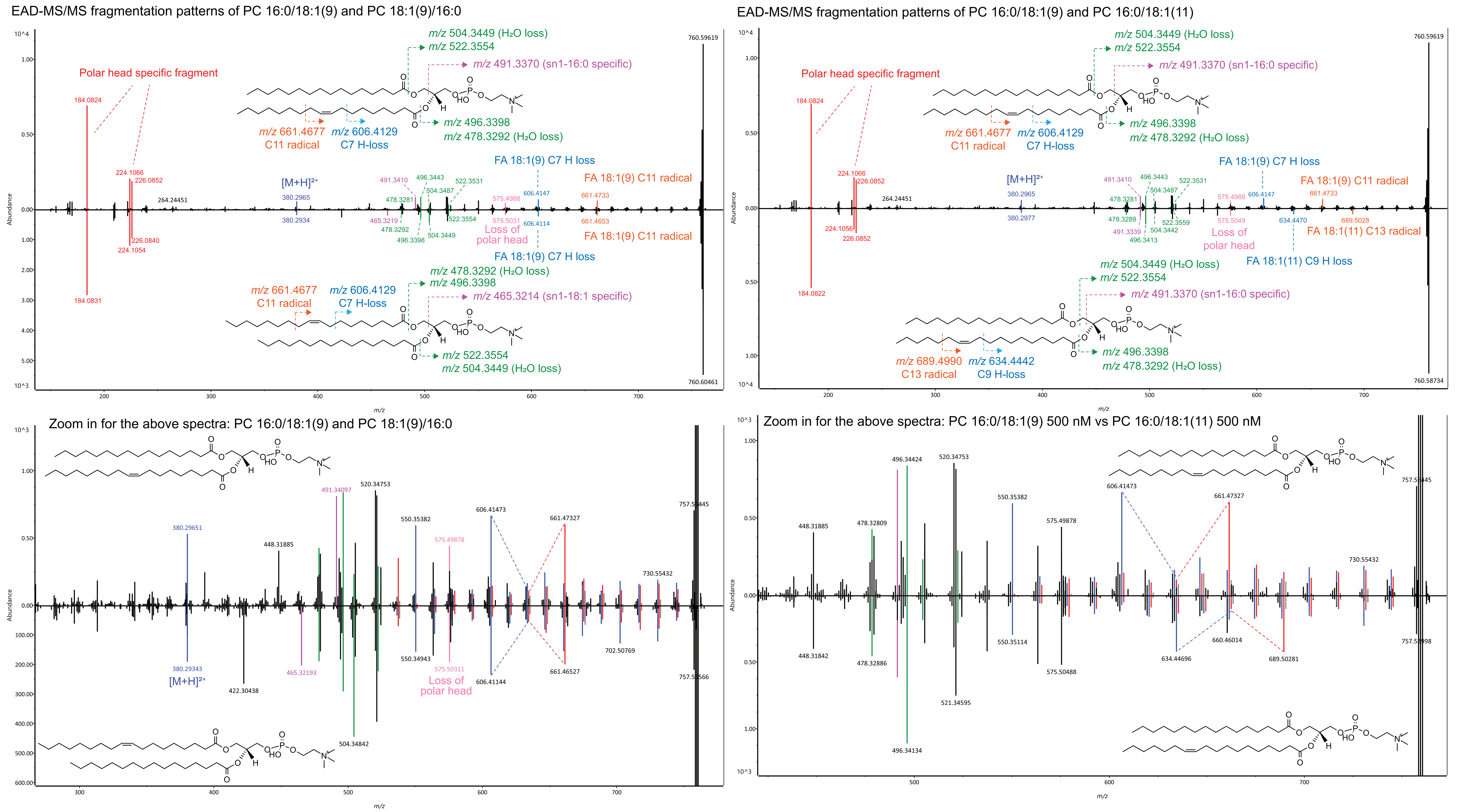

c. Calibration curves for PC 16:0/18:1(9), PC 18:1(9)/16:0, and PC 16:0/18:1(11)

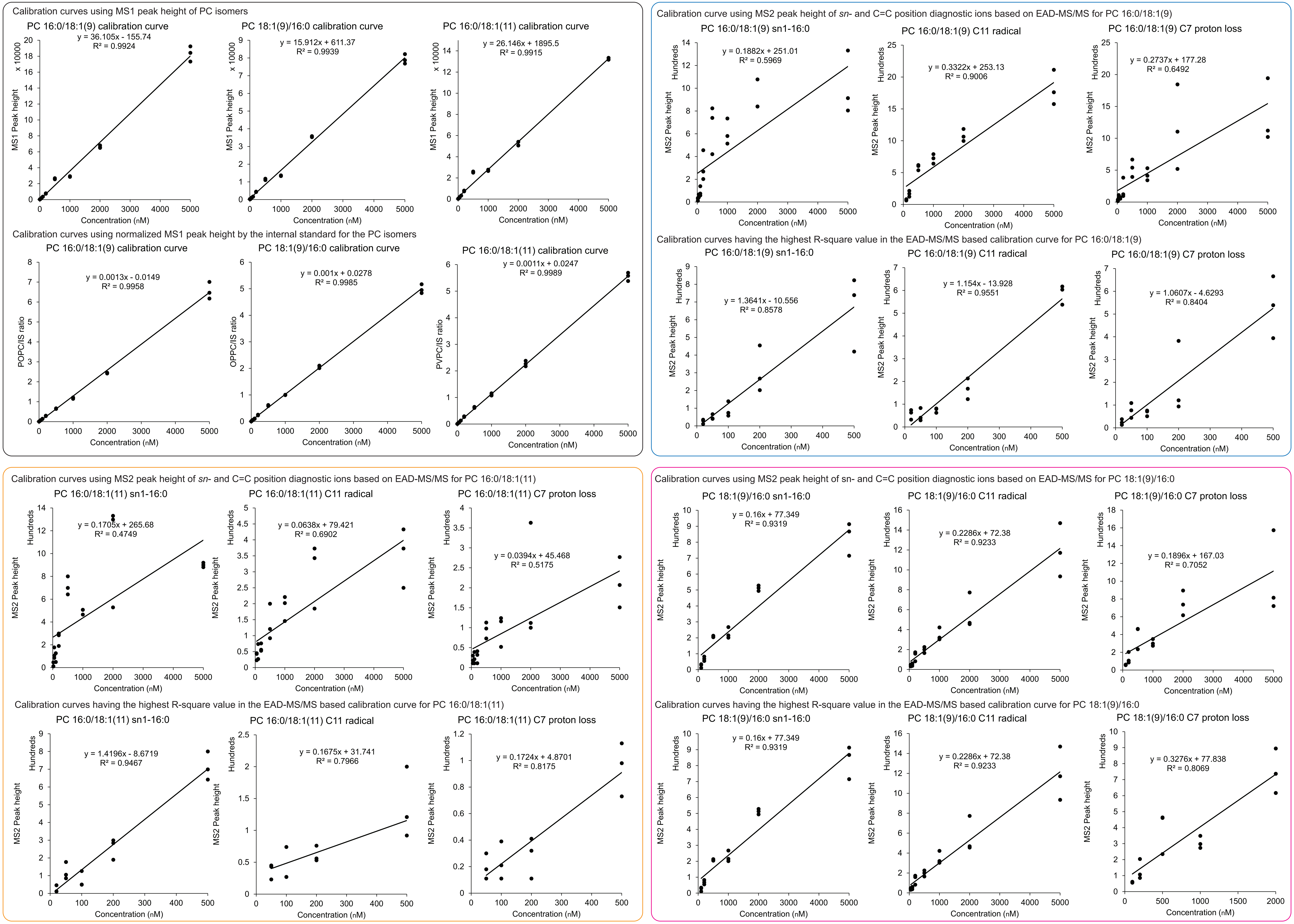

d. Relationship between concentration ratio and MS2 peak height ratio in PC mixture

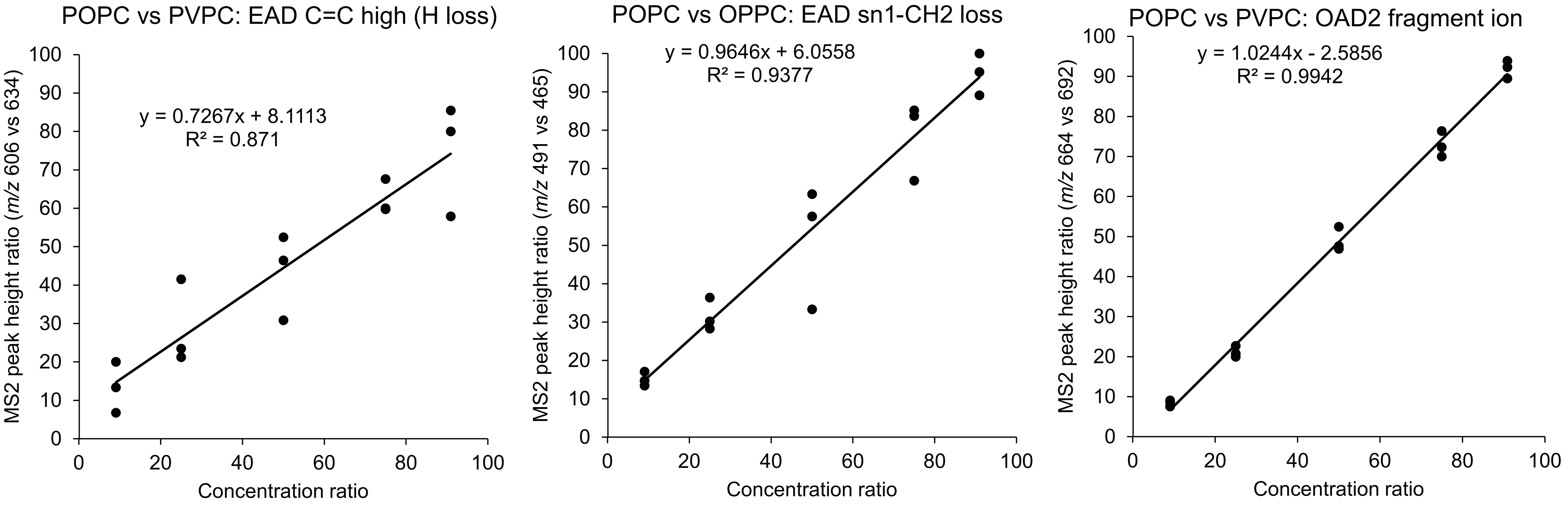

e. Isomer ratio estimations for *Δ9/Δ11* and *sn1*-16:0/*sn1*-18:1 in mouse brain and human plasma using EAD and OAD

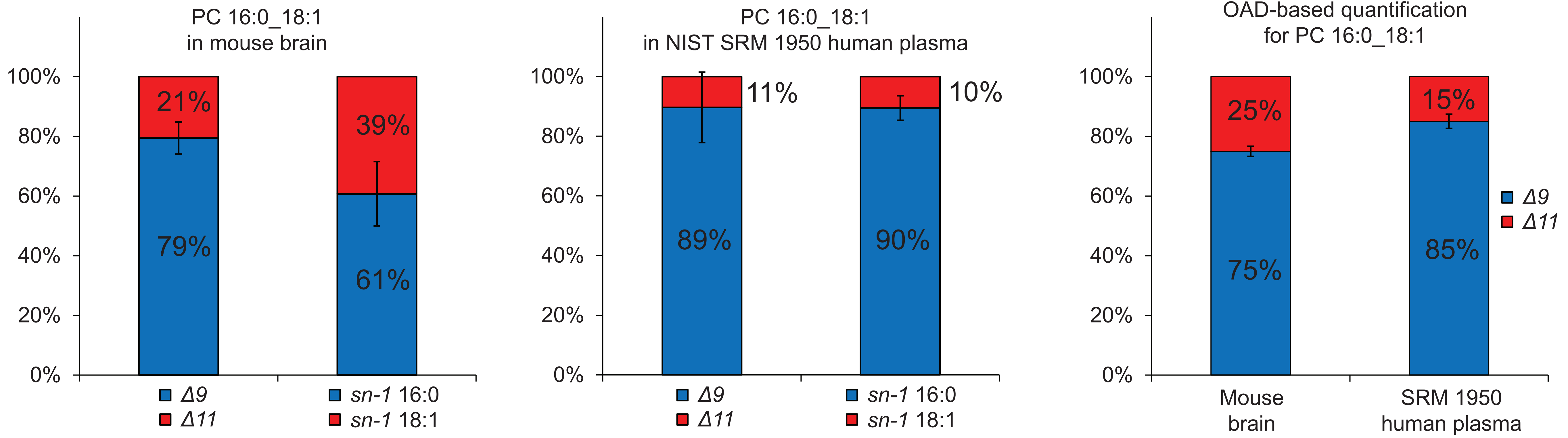

f. OAD-MS/MS fragmentation patterns of PC 16:0/18:1(9) and PC 16:0/18:1(11)

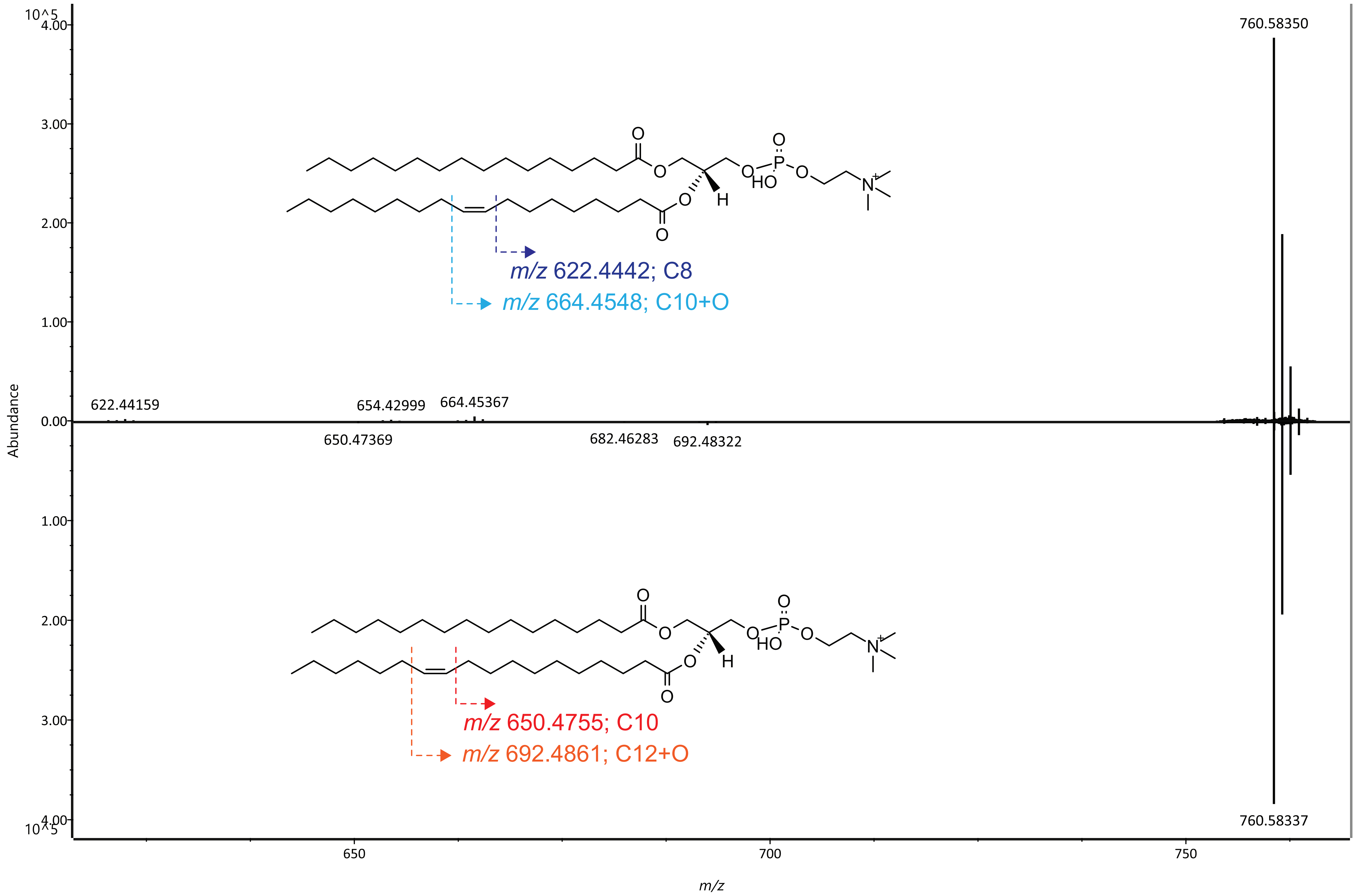

Zoom in for the above spectra: PC 16:0/18:1(9) 500 nM vs PC 16:0/18:1(11) 500 nM

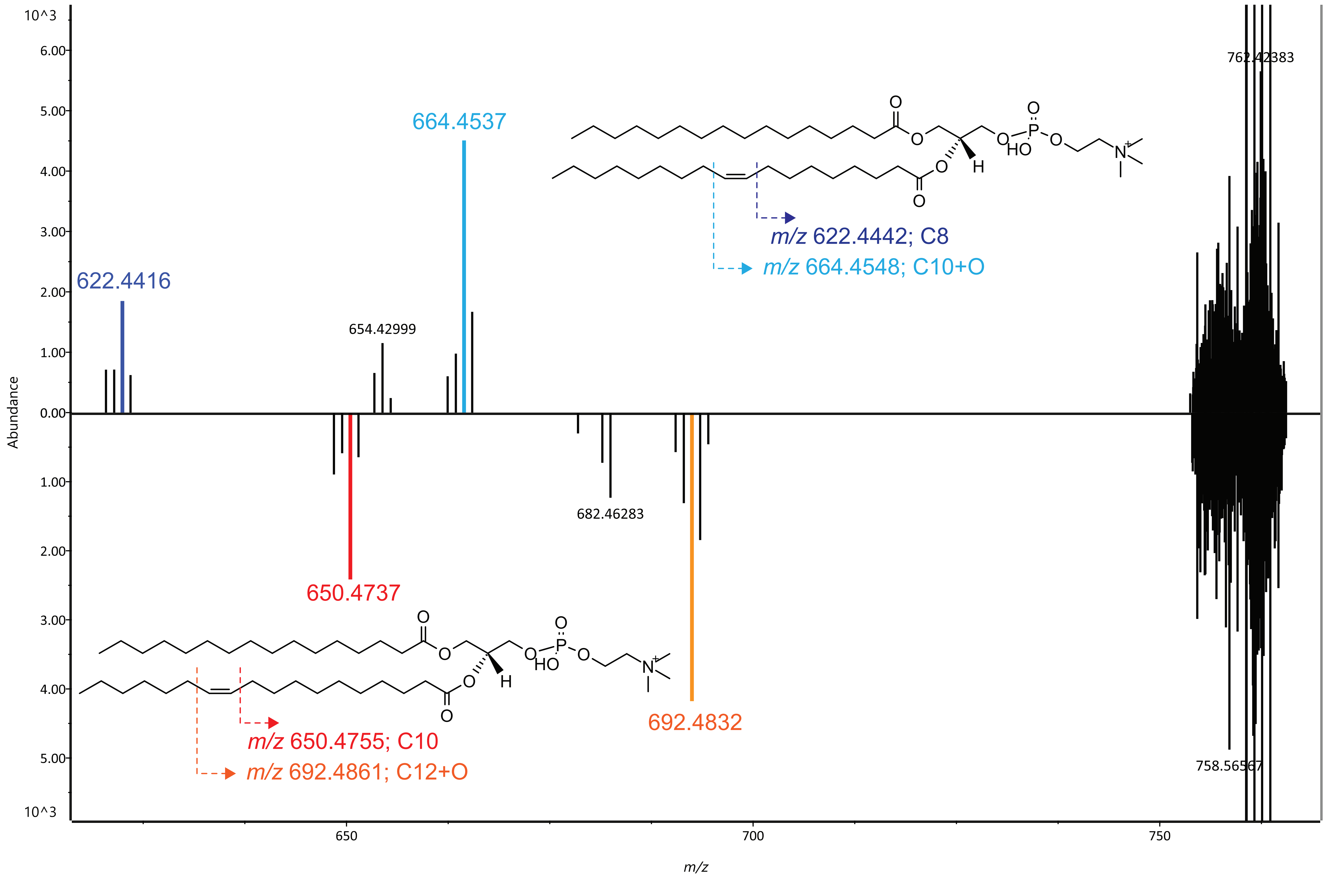

g. Calibration curve using MS2 peak height of *sn*- and C=C position diagnostic ions based on OAD-MS/MS for PC 16:0/18:1(9)

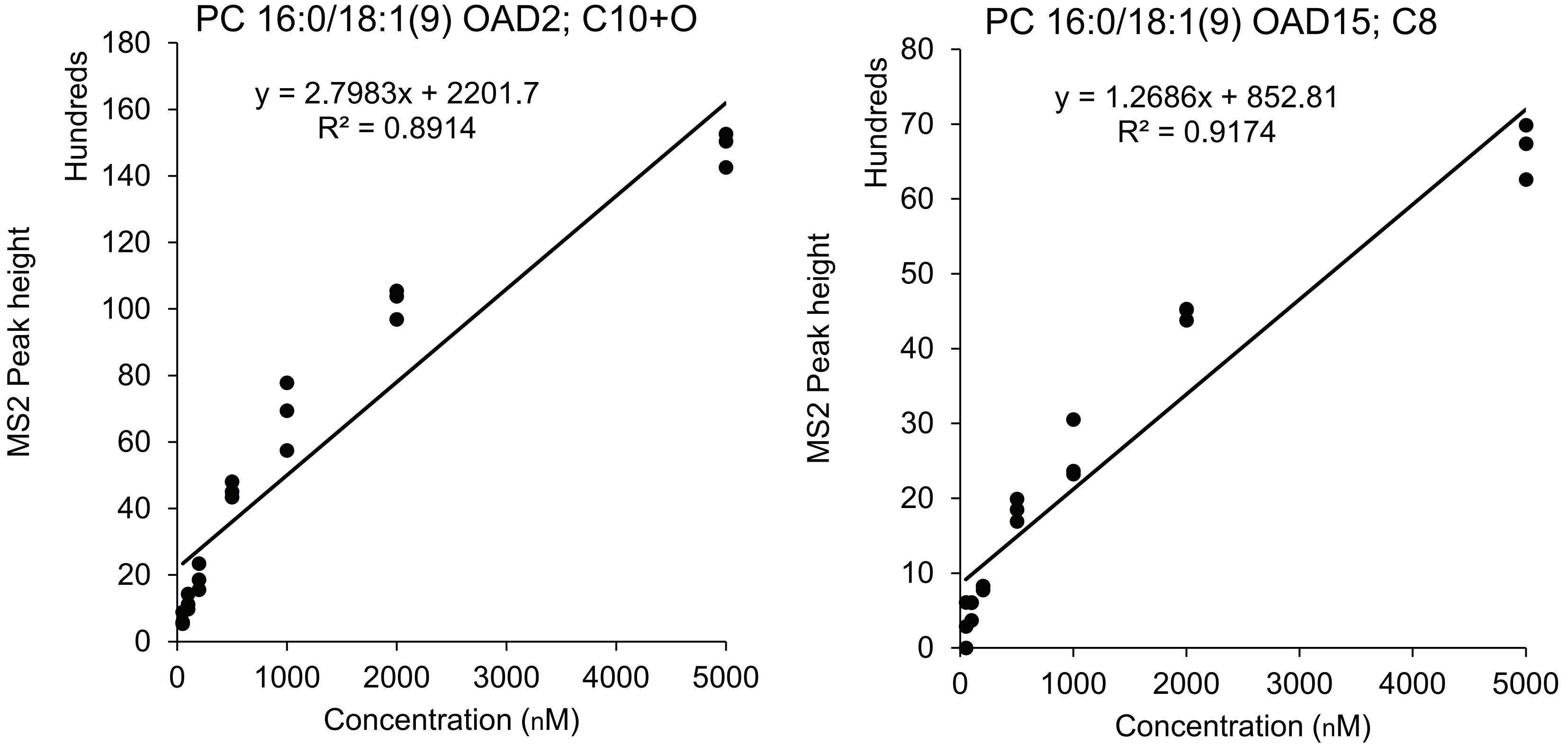

Calibration curve using MS2 peak height of sn- and C=C position diagnostic ions based on OAD-MS/MS for PC 16:0/18:1(11)

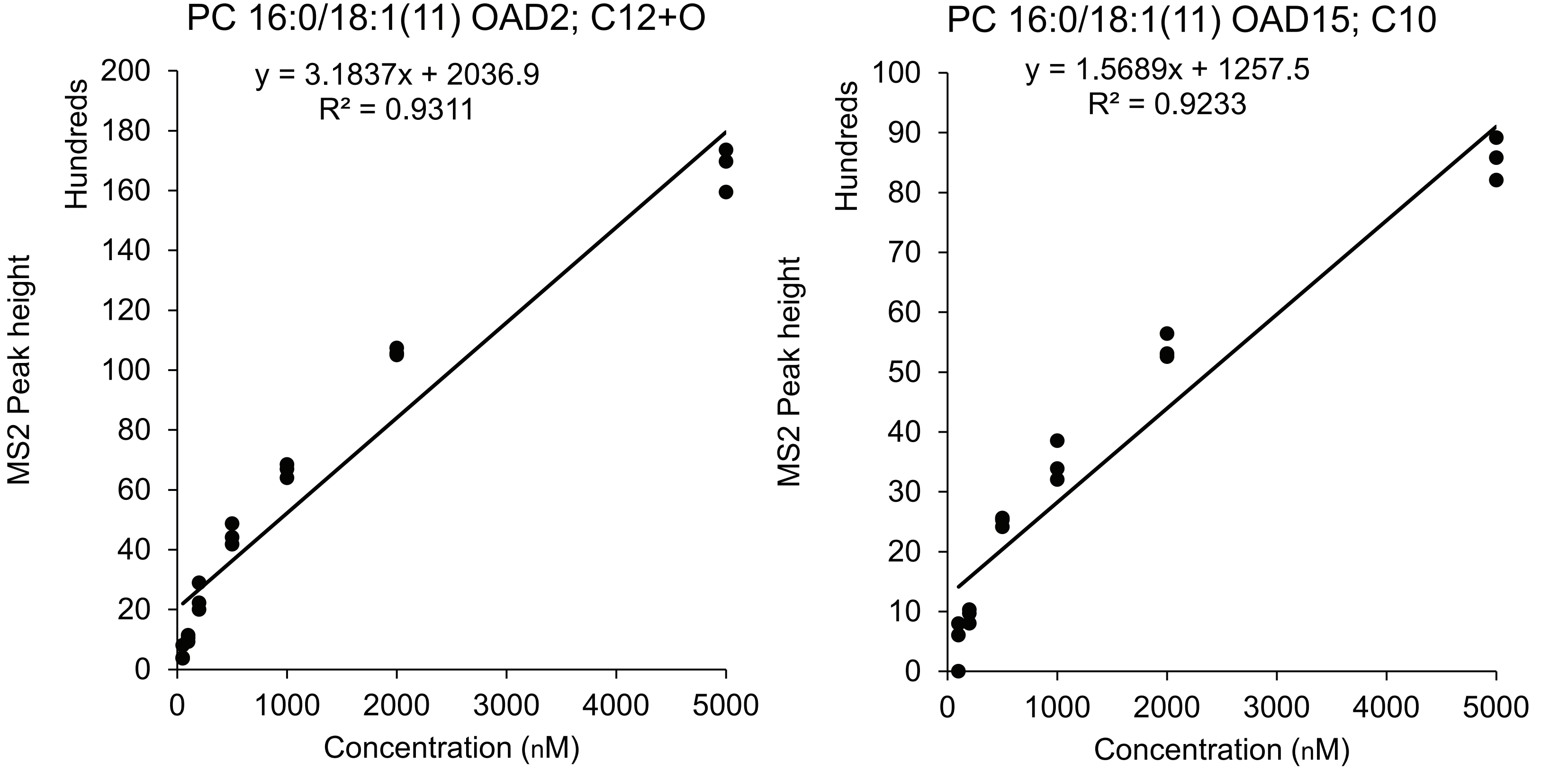

h. OAD-MS/MS in the mixture of PC isomers, plasma lipid extract, and brain lipid extract

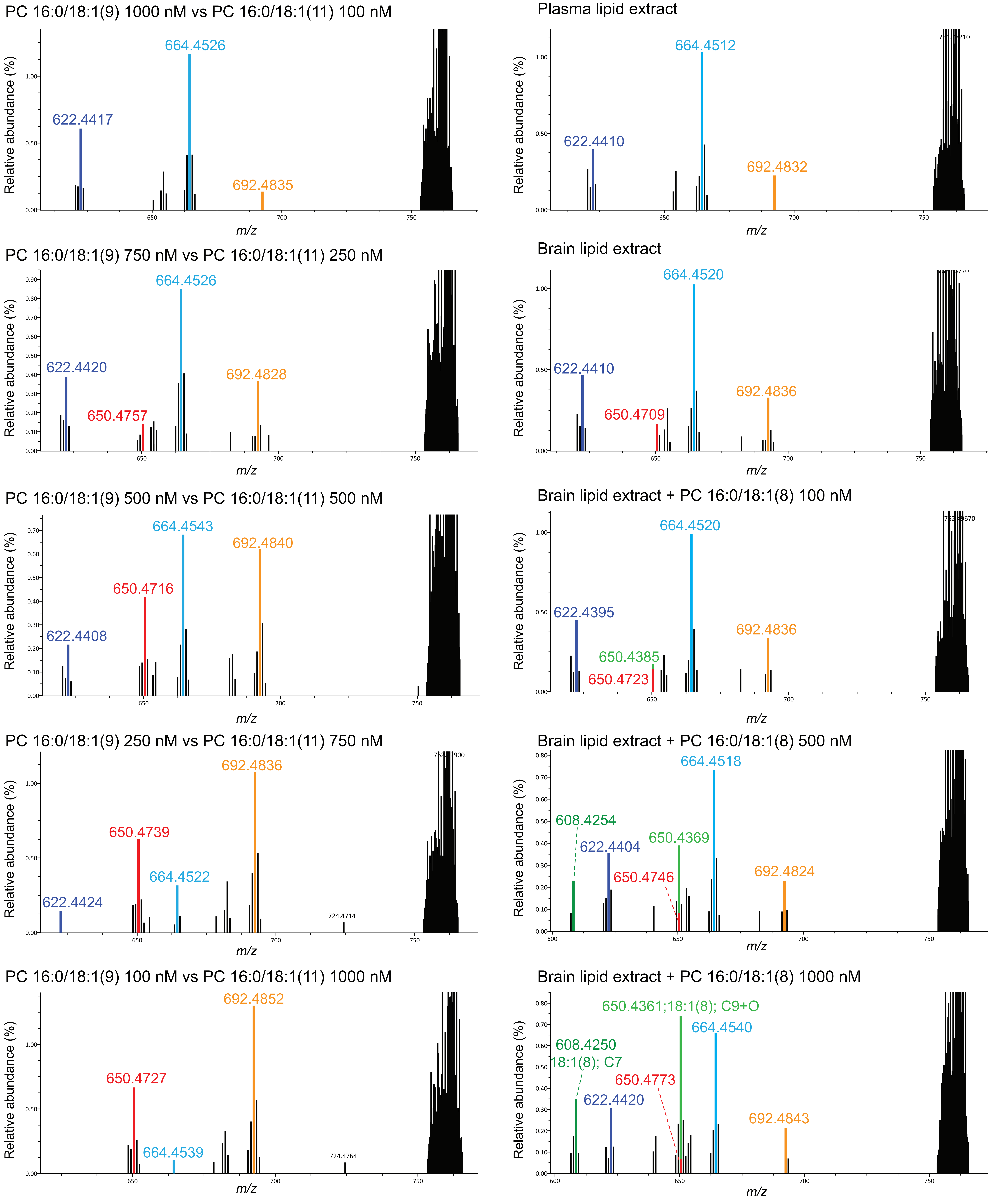

Supplementary Figure 8. Annotation results in the co-elution situation of two lipids having the same *m/z* value. The left panel shows the result of "DLPC-fixed," where PAPC concentrations varied at 0.1, 0.2, 0.5, 1.0, 2.0, 5.0, and 10 μM. The right panel shows the result of "PAPC" fixed where DLPC concentrations adjusted to 0.1, 0.2, 0.5, 1.0, 2.0, 5.0, and 10 μM. The term "Full description" means that both sn- and C=C-positions were characterized by the MS-DIAL program. The black and gray colors indicate the mis-annotation of not providing DLPC or PAPC.

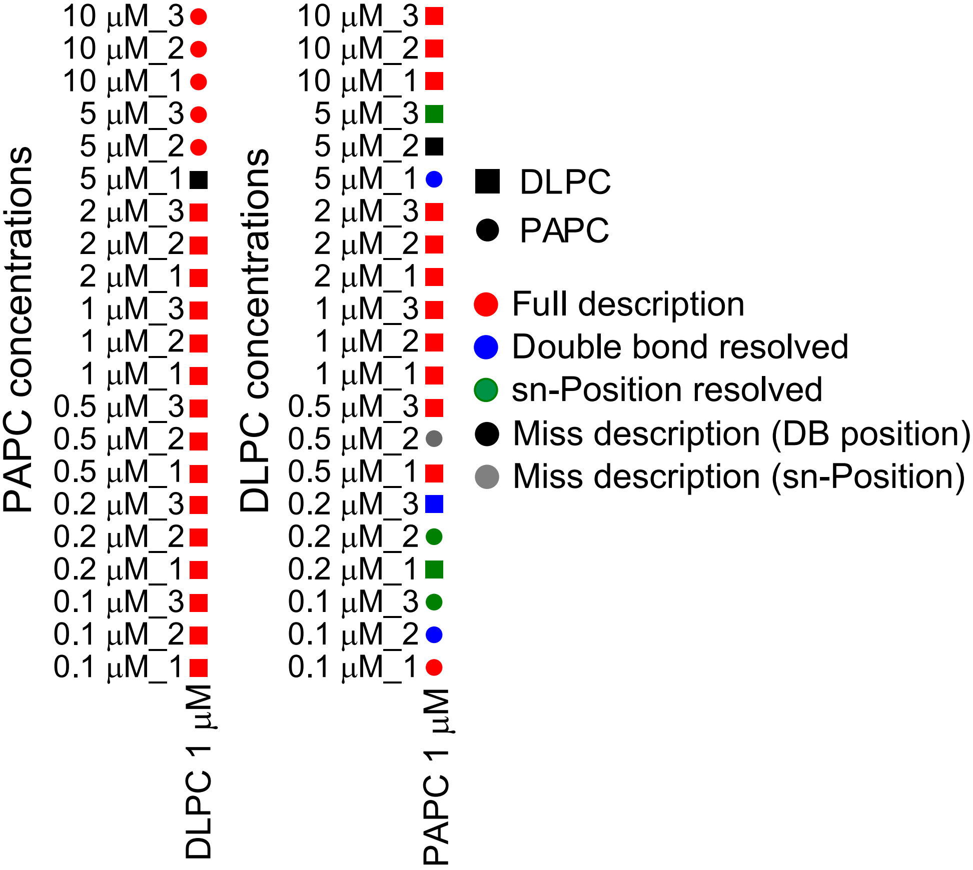
PAPC concentrations
10 μM_3
10 μM_2
10 μM_1
5 μM_3
5 μM_2
5 μM_1
2 μM_3
2 μM_2
2 μM_1
1 μM_3
1 μM_2
1 μM_1
0.5 μM_3
0.5 μM_2
0.5 μM_1
0.2 μM_3
0.2 μM_2
0.2 μM_1
0.1 μM_3
0.1 μM_2
0.1 μM_1
DLPC 1 μM
DLPC concentrations
10 μM_3
10 μM_2
10 μM_1
5 μM_3
5 μM_2
5 μM_1
2 μM_3
2 μM_2
2 μM_1
1 μM_3
1 μM_2
1 μM_1
0.5 μM_3
0.5 μM_2
0.5 μM_1
0.2 μM_3
0.2 μM_2
0.2 μM_1
0.1 μM_3
0.1 μM_2
0.1 μM_1
PAPC 1 μM
DLPC
PAPC
Full description
Double bond resolved
sn-Position resolved
Miss description (DB position)
Miss description (sn-Position)

Supplementary Figure 9. Fragment annotations for the top 5 abundant phosphatidylcholine (PC) molecules containing polyunsaturated fatty acid (PUFA) with more than three double bonds. One of the five molecules is described in **Figure 4**. EAD-MS/MS spectra for PC 16:0/22:6(4,7,10,13,16,19), PC 18:0/22:6(4,7,10,13,16,19), PC 16:0/20:4(5,8,11,14), and PC 34:5(19,22,25,28,31)/22:6(4,7,10,13,16,19) are described following the annotation in Figure 4.

a. Fragment annotations characterized as PC 16:0/22:6(4,7,10,13,16,19) in MS-DIAL 5

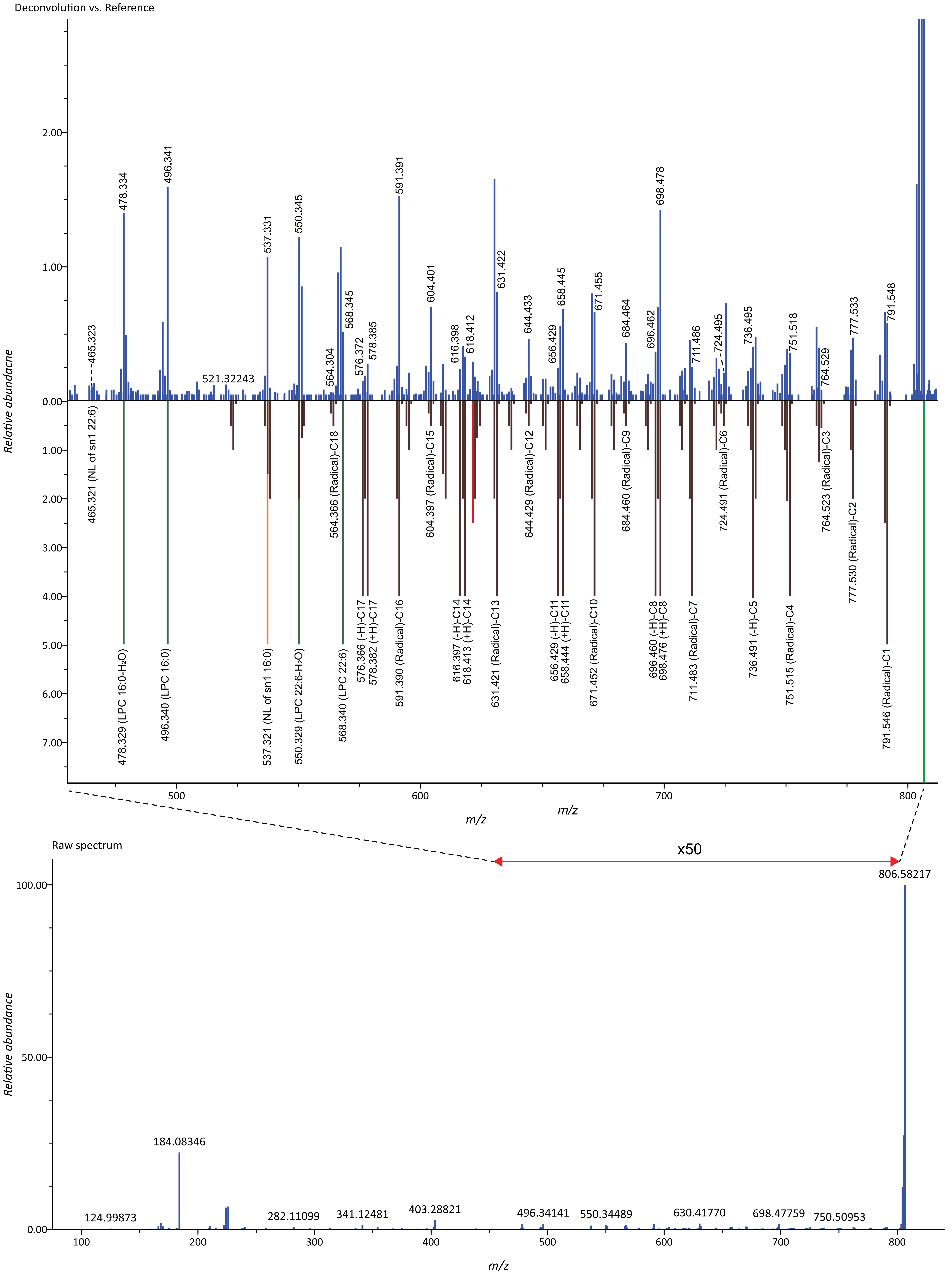

b. Fragment annotations characterized as PC 18:0/22:6(4,7,10,13,16,19) in MS-DIAL 5

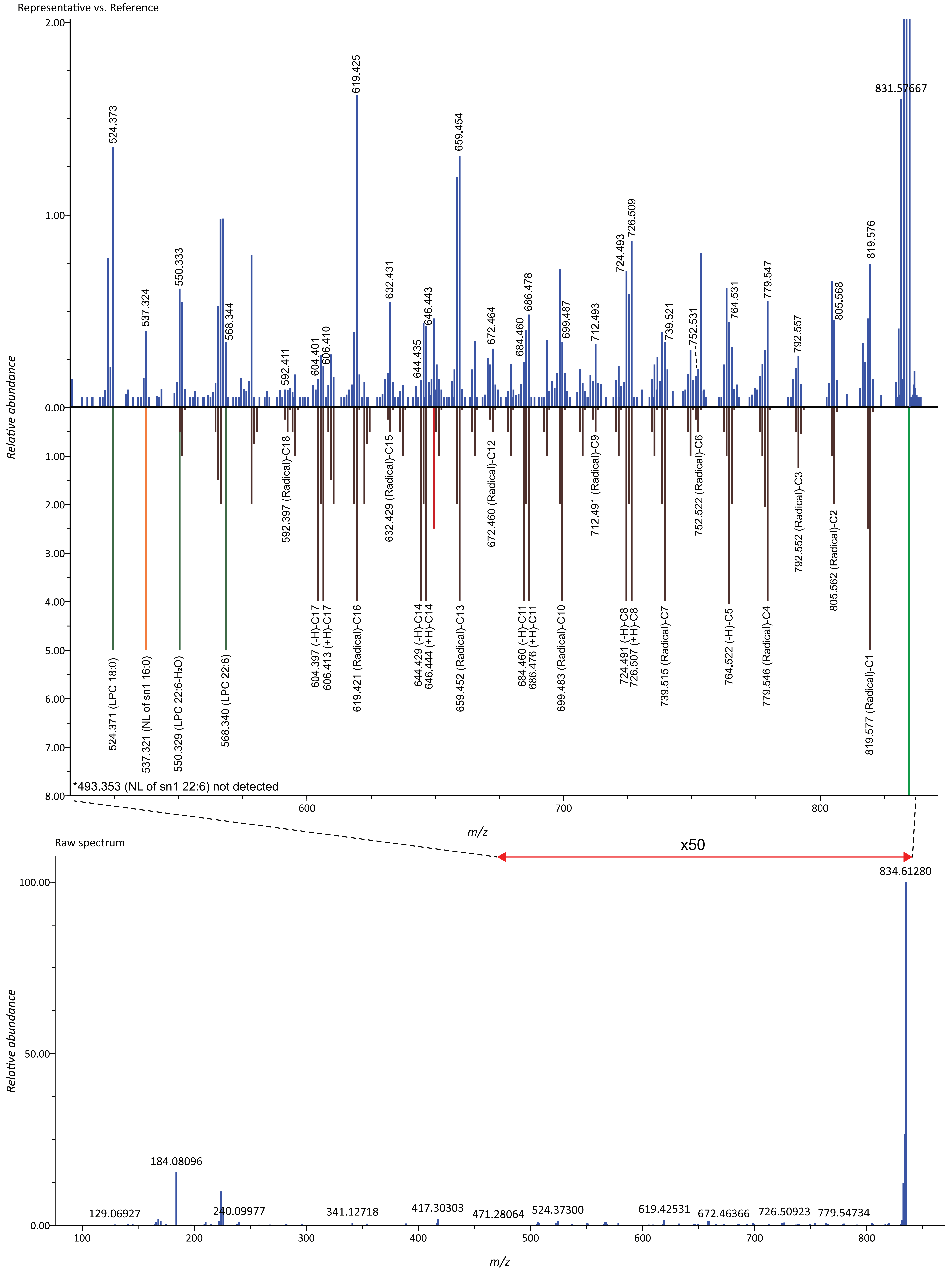

c. Fragment annotations characterized as PC 16:0/20:4(5,8,11,14) in MS-DIAL 5

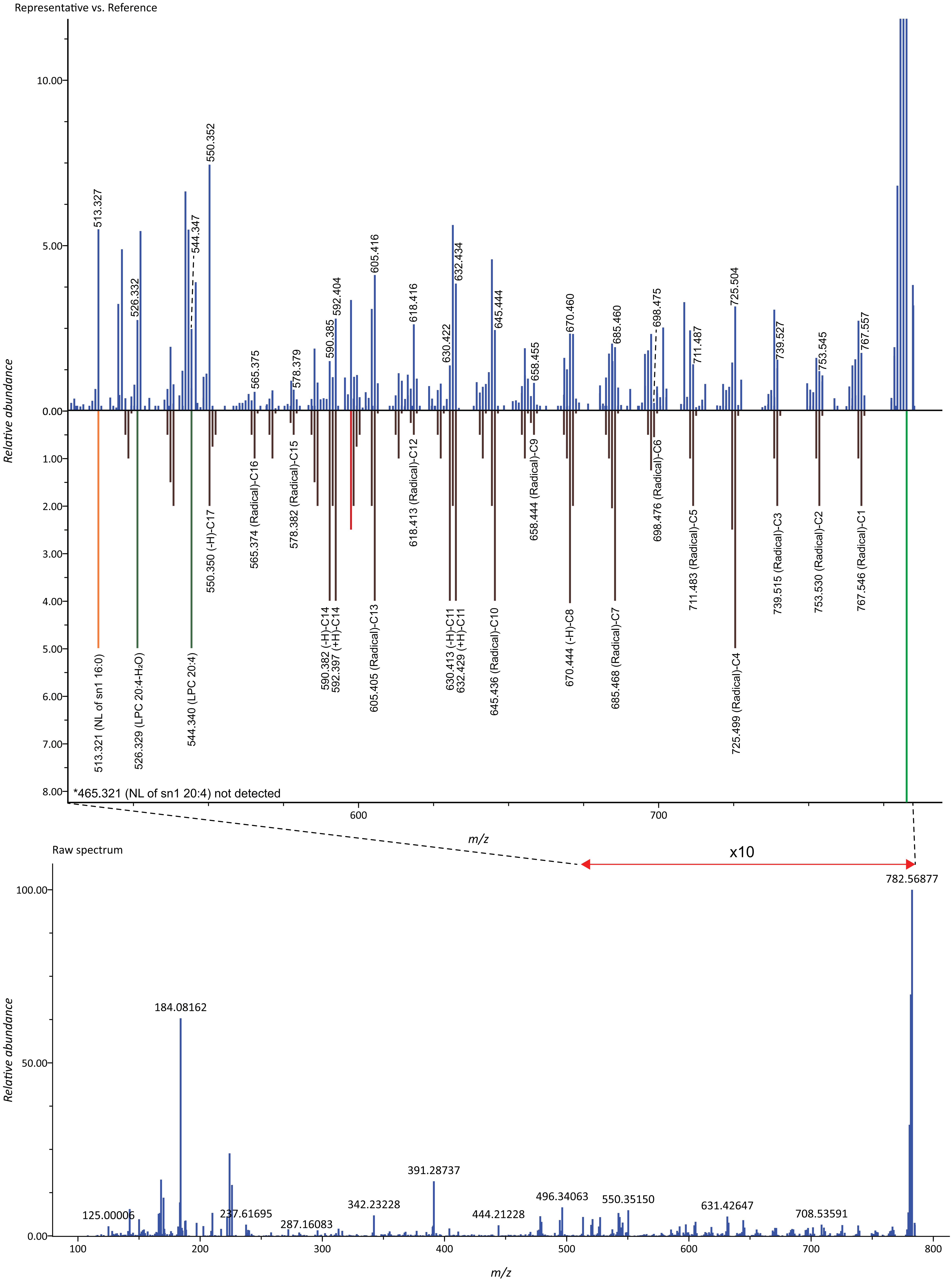

d. Fragment annotations characterized as PC 34:5(19,22,25,28,31)/22:6(4,7,10,13,16,19) in MS-DIAL 5

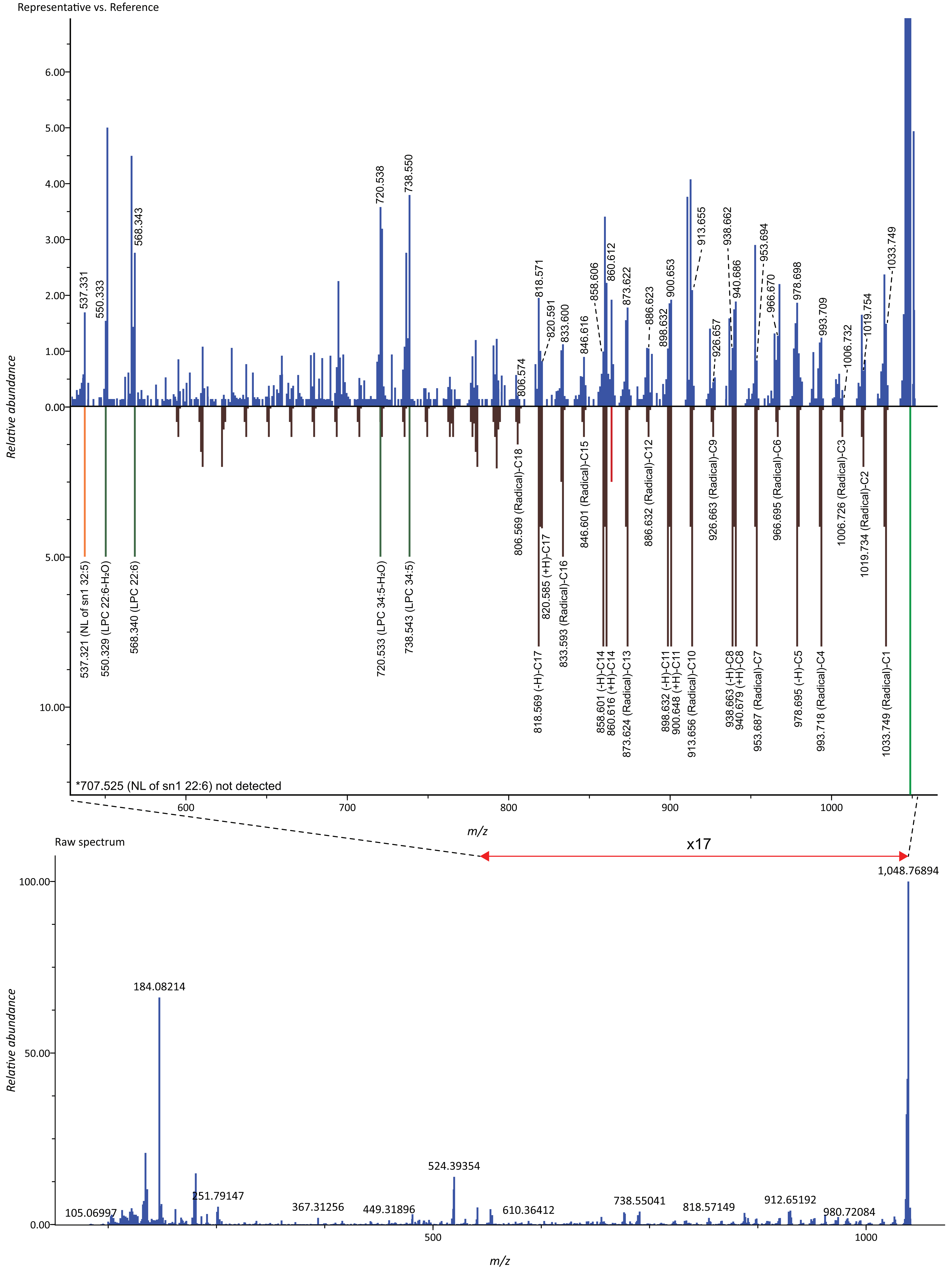

Supplementary Figure 10. EAD-MS/MS spectra for VLC-PUFA PC in HeLa cells with an abundance of sn1-VLC-PUFA higher than that of sn1-16:0 or sn1-18:1. (a) EAD-MS/MS spectrum annotated as PC 32:6/18:1 by MS-DIAL 5 as the major product. (b) EAD-MS/MS spectrum annotated as PC 32:6/16:0 by MS-DIAL 5 as the major product.

a. EAD-MS/MS spectrum annotated as PC 32:6(14,17,20,23,26,29)/16:0 in HeLa cells supplemented with FA 32:6(14,17,20,23,26,29)

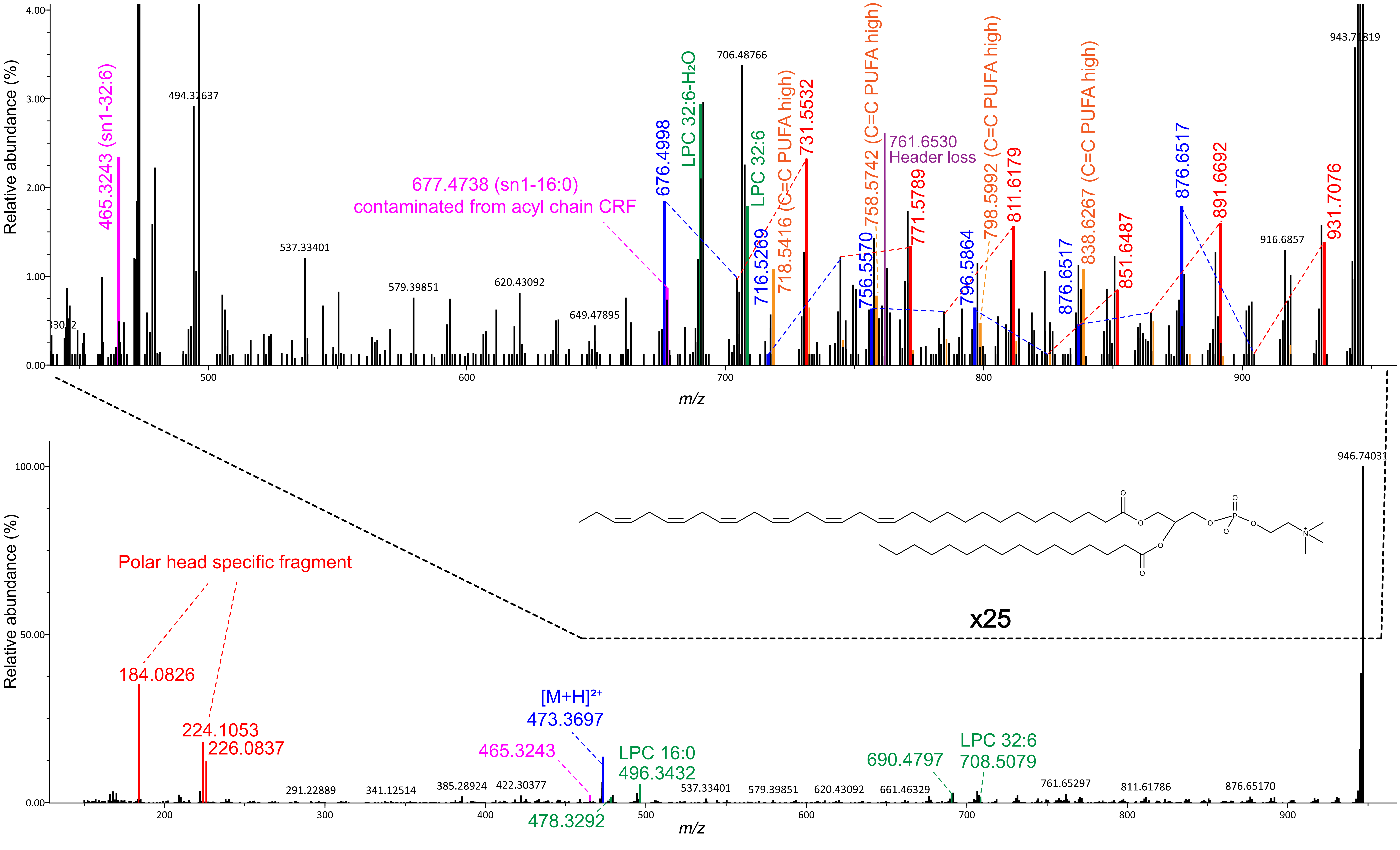

b. EAD-MS/MS spectrum annotated as PC 32:6(14,17,20,23,26,29)/18:1(9) in HeLa cells supplemented with FA 32:6(14,17,20,23,26,29)

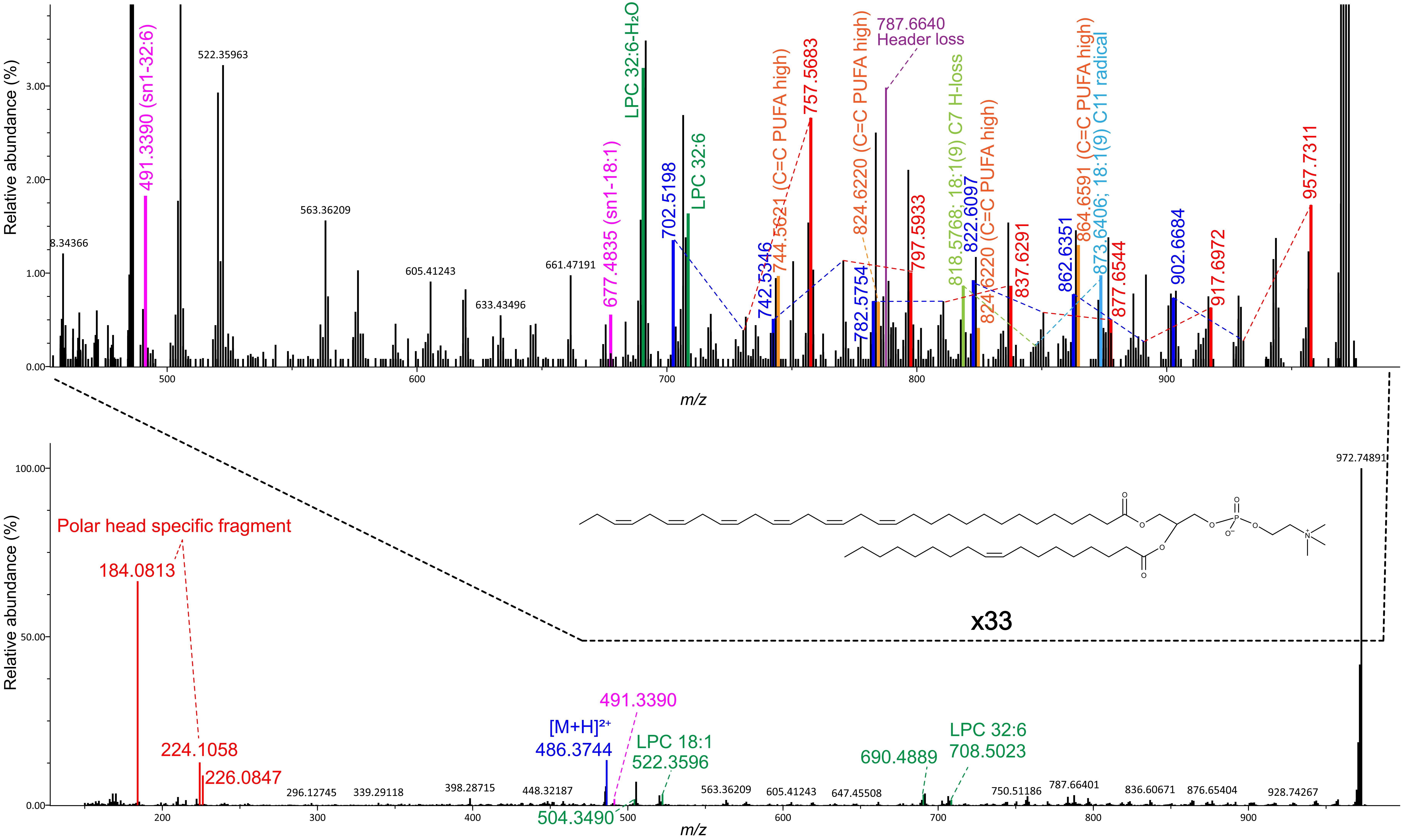

Supplementary Figure 11. Expression levels of GPAT genes. The GPAT expression levels were downloaded from http://biogps.org/?full#goto=welcome on January 21, 2024. When multiple data sets were available for the expression tables, the data set with the highest expression level for each GPAT enzyme was selected.

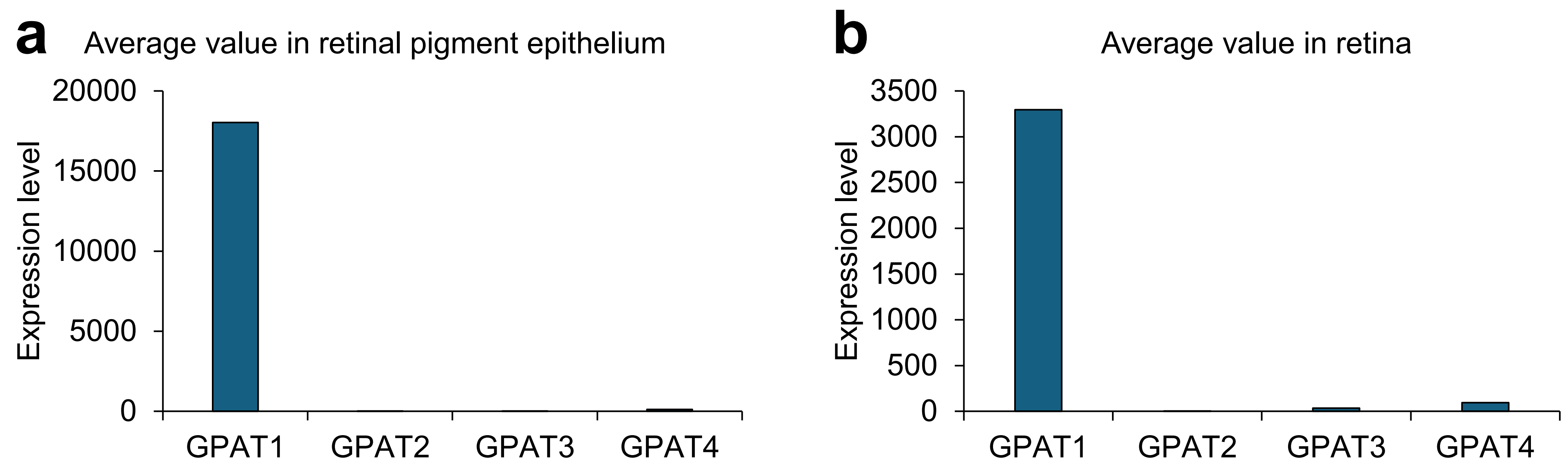
a
Average value in retinal pigment epithelium
Expression level
20000
15000
10000
5000
0
GPAT1
GPAT2
GPAT3
GPAT4
b
Average value in retina
Expression level
3500
3000
2500
2000
1500
1000
500
0
GPAT1
GPAT2
GPAT3
GPAT4

Supplementary Figure 12. Coomassie brilliant blue (CBB) staining for recombinant proteins.

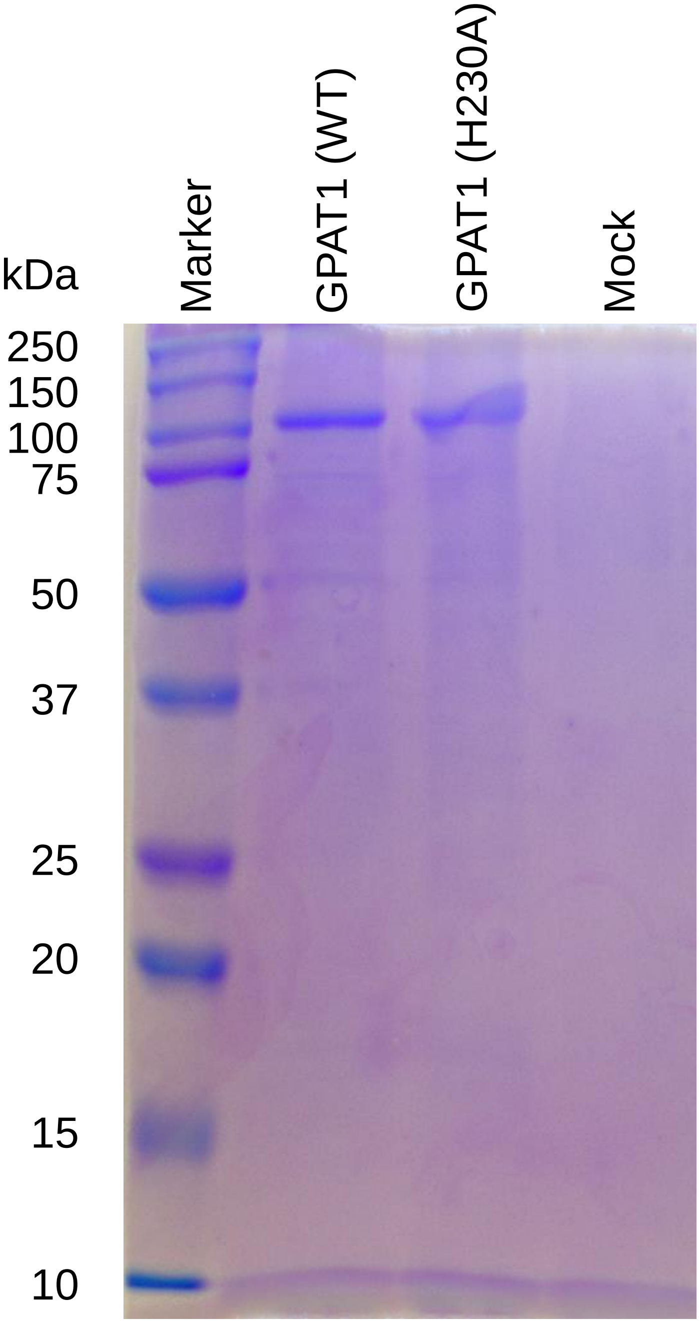
kDa
Marker
GPAT1 (WT)
GPAT1 (H230A)
Mock
250
150
100
75
50
37
25
20
15
10

Note 1. Lipidomics minimal reporting checklist for MS-DIAL EAD spectral annotation.

Separation Workflow

Overall study design

Title of the study	Overview of MS-DIAL lipid annotation for EAD-MS/MS		
Document creation date	02/05/2024	Corresponding Email	htsugawa@go.tuat.ac.jp
Principle investigator	Hiroshi Tsugawa	Is the workflow targeted or untargeted?	Untargeted
Institution	Tokyo University of Agriculture and Technology	Clinical	No

Lipid extraction

Extraction method	This reporting checklist is not for the description of sample analysis, but for the description of MS-DIAL 5 algorithm.	Were internal standards added prior extraction?	No
pH adjustment	None		

Analytical platform

Which solvents were used	NA	Mass resolution for detected ion at MS1	High resolution
Number of separation dimensions	One dimension	Resolution at m/z 200 at MS1	35000
Separation type 1	LC	Mass accuracy in ppm at MS1	2.5
Separation mode 1 (liquid)	RP	Mass window for precursor ion isolation (in Da total isolation window)	1
Detector	Mass spectrometer	Mass resolution for detected ion at MS2	High resolution
MS type	QTOF	Resolution at m/z 200 at MS2	25000
MS vendor	SCIEX	Mass accuracy in ppm at MS2	5
Ion source	ESI	Was/Were additional dimension/techniques used	Yes
MS Level	MS1, MS2		

Quality control

Blanks	No	Quality control	No

Method qualification and validation

Method validation	No

Reporting

Are reported raw data uploaded into repository?	Yes	Summary data	Identification data
Link to repository / ID to entry	http://prime.psc.riken.jp/menta.cgi/prime/drop_index, DM0054	Raw data upload	Yes
Are metadata available?	Yes	Additional comments	The uploaded files are msp files containing MS/MS spectra of lipid standards.

Lipid Class Descriptions

1) BMP[M+NH4]+ / Lipid identification

Lipid class	BMP	Did you presume assumptions for identification?	No
Derivatization	-	Check isomer overlap	No
MS Level for identification	MS1, MS2	RT verified by standard	Yes
Identification level	Double bond position	Separation of isobaric/isomeric interferece confirmed	Yes
Polarity mode	Positive	Model for separation prediction	Yes
Type of positive (precursor)ion	[M+NH4]+	Additional dimension/techniques	EAD
Fragments for identification	**Fragment name**<br>FA1(+C3H6O2)<br>FA2(+C3H6O2)<br>HG(GP,155)<br>-HG(GP,172)<br>FA1(+C3H5O4P)<br>FA2(+C3H5O4P)	How was/were the additional dimension(s) used?	To determine sn- positions
Isotope correction at MS1	No	Was a model used to predict lipid molecule separation?	No
Isotope correction at MS2	No	Lipid Identification Software	MS-DIAL
MS1 verified by standard	Yes	Data manipulation	Smoothing, Centroiding
MS2 verified by standard	Yes	Nomenclature for intact lipid molecule	Yes
Background check at MS1	Yes	Nomenclature for fragment ions	No
Background check at MS2	No	Further identification remarks	The reverse dot product similarity value, where the in silico spectrum is used for the library template, is used as the correlation coefficient value. The candidates are ranked by the reverse dot product score and the candidate with the highest similarity value is described as the representative C=C isomer candidate for the EAD-MS/MS spectrum.

1) BMP[M+NH4]+ / Lipid quantification

Quantitative	No	Batch correction	No
Normalization to reference	No	Further quantification remarks	-

2) Acylcarnitine (CAR)[M+H]+ / Lipid identification

Lipid class	Acylcarnitine (CAR)	Did you presume assumptions for identification?	No
Derivatization	-	Check isomer overlap	No
MS Level for identification	MS1, MS2	RT verified by standard	Yes
Identification level	Double bond position	Separation of isobaric/isomeric interferece confirmed	Yes
Polarity mode	Positive	Model for separation prediction	Yes
Type of positive (precursor)ion	[M+H]+	Additional dimension/techniques	EAD
Fragments for identification<br>**Fragment name**<br>Characteristic fragment (C4H5O2+)		How was/were the additional dimension(s) used?	To determine doublebond positions
Isotope correction at MS1	No	Was a model used to predict lipid molecule separation?	No
Isotope correction at MS2	No	Lipid Identification Software	MS-DIAL
MS1 verified by standard	Yes	Data manipulation	Smoothing, Centroiding
MS2 verified by standard	Yes	Nomenclature for intact lipid molecule	Yes
Background check at MS1	Yes	Nomenclature for fragment ions	No
Background check at MS2	No	Further identification remarks	The reverse dot product similarity value, where the in silico spectrum is used for the library template, is used as the correlation coefficient value. The candidates are ranked by the reverse dot product score and the candidate with the highest similarity value is described as the representative C=C isomer candidate for the EAD-MS/MS spectrum.

2) Acylcarnitine (CAR)[M+H]+ / Lipid quantification

Quantitative	No	Batch correction	No
Normalization to reference	No	Further quantification remarks	-

3) CL[M+NH4]+ / Lipid identification

Lipid class	CL	Did you presume assumptions for identification?	No
Derivatization	-	Check isomer overlap	No
MS Level for identification	MS1, MS2	RT verified by standard	Yes
Identification level	Molecular species level	Separation of isobaric/isomeric interferece confirmed	Yes
Polarity mode	Positive	Model for separation prediction	Yes
Type of positive (precursor)ion	[M+NH4]+	Additional dimension/techniques	EAD
Fragments for identification		How was/were the additional dimension(s) used?	To determine sn- positions
Isotope correction at MS1	No	Was a model used to predict lipid molecule separation?	No
Isotope correction at MS2	No	Lipid Identification Software	MS-DIAL
MS1 verified by standard	Yes	Data manipulation	Smoothing, Centroiding
MS2 verified by standard	Yes	Nomenclature for intact lipid molecule	Yes
Background check at MS1	Yes	Nomenclature for fragment ions	No
Background check at MS2	No	Further identification remarks	The reverse dot product similarity value, where the in silico spectrum is used for the library template, is used as the correlation coefficient value. The candidates are ranked by the reverse dot product score and the candidate with the highest similarity value is described as the representative C=C isomer candidate for the EAD-MS/MS spectrum.

Fragment name
Dehydro-monoacyl glycerols
Fatty acyl fragment
Dehydro-diacyl glycerols

3) CL[M+NH4]+ / Lipid quantification

Quantitative	No	Batch correction	No
Normalization to reference	No	Further quantification remarks	-

4) DG[M+NH4]+ / Lipid identification

Lipid class	DG	Did you presume assumptions for identification?	No
Derivatization	-	Check isomer overlap	No
MS Level for identification	MS1, MS2	RT verified by standard	Yes
Identification level	Double bond position	Separation of isobaric/isomeric interferece confirmed	Yes
Polarity mode	Positive	Model for separation prediction	Yes
Type of positive (precursor)ion	[M+NH4]+	Additional dimension/techniques	EAD
Fragments for identification	**Fragment name**<br>-(H2O+NH3,35)<br>The reverse dot product similarity value, where the in silico spectrum is used for the library template, is used as the correlation coefficient value. The candidates are ranked by the reverse dot product score and the candidate with the highest similarity value is described as the representative C=C isomer candidate for the EAD-MS/MS spectrum.<br>-FA1(-H)-(H2O+NH3)<br>-FA2(-H)-(H2O+NH3)	How was/were the additional dimension(s) used?	To determine doublebond positions
Isotope correction at MS1	No	Was a model used to predict lipid molecule separation?	No
Isotope correction at MS2	No	Lipid Identification Software	MS-DIAL
MS1 verified by standard	Yes	Data manipulation	Smoothing, Centroiding
MS2 verified by standard	Yes	Nomenclature for intact lipid molecule	Yes
Background check at MS1	Yes	Nomenclature for fragment ions	No
Background check at MS2	No	Further identification remarks	The reverse dot product similarity value, where the in silico spectrum is used for the library template, is used as the correlation coefficient value. The candidates are ranked by the reverse dot product score and the candidate with the highest similarity value is described as the representative C=C isomer candidate for the EAD-MS/MS spectrum.

4) DG[M+NH4]+ / Lipid quantification

Quantitative	No	Batch correction	No
Normalization to reference	No	Further quantification remarks	-

5) DG[M+Na]+ / Lipid identification

Lipid class	DG	Did you presume assumptions for identification?	No
Derivatization	-	Check isomer overlap	No
MS Level for identification	MS1, MS2	RT verified by standard	Yes
Identification level	Double bond position	Separation of isobaric/isomeric interferece confirmed	Yes
Polarity mode	Positive	Model for separation prediction	Yes
Type of positive (precursor)ion	[M+Na]+	Additional dimension/techniques	EAD
Fragments for identification	**Fragment name**<br>-FA1(-H)-(H2O+NH3)<br>-FA2(-H)-(H2O+NH3)	How was/were the additional dimension(s) used?	To determine the sn- and doublebond positions
Isotope correction at MS1	No	Was a model used to predict lipid molecule separation?	No
Isotope correction at MS2	No	Lipid Identification Software	MS-DIAL
MS1 verified by standard	Yes	Data manipulation	Smoothing, Centroiding
MS2 verified by standard	Yes	Nomenclature for intact lipid molecule	Yes
Background check at MS1	Yes	Nomenclature for fragment ions	No
Background check at MS2	No	Further identification remarks	The reverse dot product similarity value, where the in silico spectrum is used for the library template, is used as the correlation coefficient value. The candidates are ranked by the reverse dot product score and the candidate with the highest similarity value is described as the representative C=C isomer candidate for the EAD-MS/MS spectrum.

5) DG[M+Na]+ / Lipid quantification

Quantitative	No	Batch correction	No
Normalization to reference	No	Further quantification remarks	-

6) Diacylglyceryl hydroxymethyl-N,N,N-trimethyl-beta-alanine (DGTA)[M+H]+ / Lipid identification

Lipid class	Diacylglyceryl hydroxymethyl-N,N,N-trimethyl-beta-alanine (DGTA)	Did you presume assumptions for identification?	No
Derivatization	-	Check isomer overlap	No
MS Level for identification	MS1, MS2	RT verified by standard	Yes
Identification level	Double bond position	Separation of isobaric/isomeric interferece confirmed	Yes
Polarity mode	Positive	Model for separation prediction	Yes
Type of positive (precursor)ion	[M+H]+	Additional dimension/techniques	EAD
Fragments for identification		How was/were the additional dimension(s) used?	To determine sn- and doublebond positions
Isotope correction at MS1	No	Was a model used to predict lipid molecule separation?	No
Isotope correction at MS2	No	Lipid Identification Software	MS-DIAL
MS1 verified by standard	No	Data manipulation	Smoothing, Centroiding
MS2 verified by standard	No	Nomenclature for intact lipid molecule	Yes
Background check at MS1	Yes	Nomenclature for fragment ions	No
Background check at MS2	No	Further identification remarks	The reverse dot product similarity value, where the in silico spectrum is used for the library template, is used as the correlation coefficient value. The candidates are ranked by the reverse dot product score and the candidate with the highest similarity value is described as the representative C=C isomer candidate for the EAD-MS/MS spectrum.

Fragment name
Characteristic fragment (C7H14NO2+)
HG + C3H6+
HG + C2H4O+

6) Diacylglyceryl hydroxymethyl-N,N,N-trimethyl-beta-alanine (DGTA)[M+H]+ / Lipid quantification

Quantitative	No	Batch correction	No
Normalization to reference	No	Further quantification remarks	-

7) Diacylglyceryl trimethylhomoserine (DGTS)[M+H]+ / Lipid identification

Lipid class	Diacylglyceryl trimethylhomoserine (DGTS)	Did you presume assumptions for identification?	No
Derivatization	-	Check isomer overlap	No
MS Level for identification	MS1, MS2	RT verified by standard	Yes
Identification level	Double bond position	Separation of isobaric/isomeric interferece confirmed	Yes
Polarity mode	Positive	Model for separation prediction	Yes
Type of positive (precursor)ion	[M+H]+	Additional dimension/techniques	EAD
Fragments for identification		How was/were the additional dimension(s) used?	To determine sn- and doublebond positions
Isotope correction at MS1	No	Was a model used to predict lipid molecule separation?	No
Isotope correction at MS2	No	Lipid Identification Software	MS-DIAL
MS1 verified by standard	No	Data manipulation	Smoothing, Centroiding
MS2 verified by standard	No	Nomenclature for intact lipid molecule	Yes
Background check at MS1	Yes	Nomenclature for fragment ions	No
Background check at MS2	No	Further identification remarks	The reverse dot product similarity value, where the in silico spectrum is used for the library template, is used as the correlation coefficient value. The candidates are ranked by the reverse dot product score and the candidate with the highest similarity value is described as the representative C=C isomer candidate for the EAD-MS/MS spectrum.

Fragment name
Characteristic fragment (C6H12NO2+)
HG + C3H6+
HG + C2H4O+

7) Diacylglyceryl trimethylhomoserine (DGTS)[M+H]+ / Lipid quantification

Quantitative	No	Batch correction	No
Normalization to reference	No	Further quantification remarks	-

8) N, N-dimethylethylenediamine derivatized fatty acid (DMEDFA)[M+H]+ / Lipid identification

Lipid class	N, N-dimethylethylenediamine derivatized fatty acid (DMEDFA)	Did you presume assumptions for identification?	No
Derivatization	-	Check isomer overlap	No
MS Level for identification	MS1, MS2	RT verified by standard	Yes
Identification level	Double bond position	Separation of isobaric/isomeric interferece confirmed	Yes
Polarity mode	Positive	Model for separation prediction	Yes
Type of positive (precursor)ion	[M+H]+	Additional dimension/techniques	EAD
Fragments for identification		How was/were the additional dimension(s) used?	To determine doublebond positions
Isotope correction at MS1	No	Was a model used to predict lipid molecule separation?	No
Isotope correction at MS2	No	Lipid Identification Software	MS-DIAL
MS1 verified by standard	No	Data manipulation	Smoothing, Centroiding
MS2 verified by standard	No	Nomenclature for intact lipid molecule	Yes
Background check at MS1	Yes	Nomenclature for fragment ions	No
Background check at MS2	No	Further identification remarks	The reverse dot product similarity value, where the in silico spectrum is used for the library template, is used as the correlation coefficient value. The candidates are ranked by the reverse dot product score and the candidate with the highest similarity value is described as the representative C=C isomer candidate for the EAD-MS/MS spectrum.

Fragment name
NL of C2NH7

8) N, N-dimethylethylenediamine derivatized fatty acid (DMEDFA)[M+H]+ / Lipid quantification

Quantitative	No	Batch correction	No
Normalization to reference	No	Further quantification remarks	-

9) N, N-dimethylethylenediamine derivatized fatty acid ester of hydroxyl fatty acid (DMED-FAHFA)[M+H]+ / Lipid identification

Lipid class	N, N-dimethylethylenediamine derivatized fatty acid ester of hydroxyl fatty acid (DMEDFAHFA)	Did you presume assumptions for identification?	No
Derivatization	-	Check isomer overlap	No
MS Level for identification	MS1, MS2	RT verified by standard	Yes
Identification level	Double bond position	Separation of isobaric/isomeric interference confirmed	Yes
Polarity mode	Positive	Model for separation prediction	Yes
Type of positive (precursor)ion	[M+H]+	Additional dimension/techniques	EAD
Fragments for identification	(see below)	How was/were the additional dimension(s) used?	To determine hydroxyl moiety position and doublebond positions
Isotope correction at MS1	No	Was a model used to predict lipid molecule separation?	No
Isotope correction at MS2	No	Lipid Identification Software	MS-DIAL
MS1 verified by standard	No	Data manipulation	Smoothing, Centroiding
MS2 verified by standard	No	Nomenclature for intact lipid molecule	Yes
Background check at MS1	Yes	Nomenclature for fragment ions	No
Background check at MS2	No	Further identification remarks	The reverse dot product similarity value, where the in silico spectrum is used for the library template, is used as the correlation coefficient value. The candidates are ranked by the reverse dot product score and the candidate with the highest similarity value is described as the representative C=C isomer candidate for the EAD-MS/MS spectrum.

Fragments for identification:

Fragment name
The reverse dot product similarity value, where the in silico spectrum is used for the library template, is used as the correlation coefficient value. The candidates are ranked by the reverse dot product score and the candidate with the highest similarity value is described as the representative C=C isomer candidate for the EAD-MS/MS spectrum.
HFA -H2O +DMED
HFA -H2O +C2H5N
Specific fragments detached at OH position on HFA

9) N, N-dimethylethylenediamine derivatized fatty acid ester of hydroxyl fatty acid (DMED-FAHFA)[M+H]+ / Lipid quantification

Quantitative	No	Batch correction	No
Normalization to reference	No	Further quantification remarks	-

10) PC O[M+H]+ / Lipid identification

Lipid class	PC O	Did you presume assumptions for identification?	No
Derivatization	-	Check isomer overlap	No
MS Level for identification	MS1, MS2	RT verified by standard	Yes
Identification level	Double bond position	Separation of isobaric/isomeric interferece confirmed	Yes
Polarity mode	Positive	Model for separation prediction	Yes
Type of positive (precursor)ion	[M+H]+	Additional dimension/techniques	EAD
Fragments for identification	Fragment name: HG(PC,184); NL of FA; NL of Ether; HG + C3H5; HG + C2H3O	How was/were the additional dimension(s) used?	To determine sn- and doublebond positions
Isotope correction at MS1	No	Was a model used to predict lipid molecule separation?	No
Isotope correction at MS2	No	Lipid Identification Software	MS-DIAL
MS1 verified by standard	Yes	Data manipulation	Smoothing, Centroiding
MS2 verified by standard	Yes	Nomenclature for intact lipid molecule	Yes
Background check at MS1	Yes	Nomenclature for fragment ions	No
Background check at MS2	No	Further identification remarks	The reverse dot product similarity value, where the in silico spectrum is used for the library template, is used as the correlation coefficient value. The candidates are ranked by the reverse dot product score and the candidate with the highest similarity value is described as the representative C=C isomer candidate for the EAD-MS/MS spectrum.

10) PC O[M+H]+ / Lipid quantification

Quantitative	No	Batch correction	No
Normalization to reference	No	Further quantification remarks	-

11) PC P[M+H]+ / Lipid identification

Lipid class	PC P	Did you presume assumptions for identification?	No
Derivatization	-	Check isomer overlap	No
MS Level for identification	MS1, MS2	RT verified by standard	Yes
Identification level	Double bond position	Separation of isobaric/isomeric interferece confirmed	Yes
Polarity mode	Positive	Model for separation prediction	Yes
Type of positive (precursor)ion	[M+H]+	Additional dimension/techniques	EAD
Fragments for identification		How was/were the additional dimension(s) used?	To determine sn- and doublebond positions

Fragment name
HG(PC,184)
NL of FA
NL of Ether
HG + C3H5
HG + C2H3O

Isotope correction at MS1	No	Was a model used to predict lipid molecule separation?	No
Isotope correction at MS2	No	Lipid Identification Software	MS-DIAL
MS1 verified by standard	Yes	Data manipulation	Smoothing, Centroiding
MS2 verified by standard	Yes	Nomenclature for intact lipid molecule	Yes
Background check at MS1	Yes	Nomenclature for fragment ions	No
Background check at MS2	No	Further identification remarks	The reverse dot product similarity value, where the in silico spectrum is used for the library template, is used as the correlation coefficient value. The candidates are ranked by the reverse dot product score and the candidate with the highest similarity value is described as the representative C=C isomer candidate for the EAD-MS/MS spectrum.

11) PC P[M+H]+ / Lipid quantification

Quantitative	No	Batch correction	No
Normalization to reference	No	Further quantification remarks	-

12) PE O[M+H]+ / Lipid identification

Lipid class	PE O	Did you presume assumptions for identification?	No
Derivatization	-	Check isomer overlap	No
MS Level for identification	MS1, MS2	RT verified by standard	Yes
Identification level	Double bond position	Separation of isobaric/isomeric interferece confirmed	Yes
Polarity mode	Positive	Model for separation prediction	Yes
Type of positive (precursor)ion	[M+H]+	Additional dimension/techniques	EAD
Fragments for identification	Fragment name: -HG(PE,141); NL of FA; NL of Ether; HG + C3H6+; HG + C2H4O+	How was/were the additional dimension(s) used?	To determine doublebond positions
Isotope correction at MS1	No	Was a model used to predict lipid molecule separation?	No
Isotope correction at MS2	No	Lipid Identification Software	MS-DIAL
MS1 verified by standard	Yes	Data manipulation	Smoothing, Centroiding
MS2 verified by standard	Yes	Nomenclature for intact lipid molecule	Yes
Background check at MS1	Yes	Nomenclature for fragment ions	No
Background check at MS2	No	Further identification remarks	The reverse dot product similarity value, where the in silico spectrum is used for the library template, is used as the correlation coefficient value. The candidates are ranked by the reverse dot product score and the candidate with the highest similarity value is described as the representative C=C isomer candidate for the EAD-MS/MS spectrum.

12) PE O[M+H]+ / Lipid quantification

Quantitative	No	Batch correction	No
Normalization to reference	No	Further quantification remarks	-

13) PE P[M+H]+ / Lipid identification

Lipid class	PE P	Did you presume assumptions for identification?	No
Derivatization	-	Check isomer overlap	No
MS Level for identification	MS1, MS2	RT verified by standard	Yes
Identification level	Double bond position	Separation of isobaric/isomeric interferece confirmed	Yes
Polarity mode	Positive	Model for separation prediction	Yes
Type of positive (precursor)ion	[M+H]+	Additional dimension/techniques	EAD
Fragments for identification		How was/were the additional dimension(s) used?	To determine doublebond positions
Isotope correction at MS1	No	Was a model used to predict lipid molecule separation?	No
Isotope correction at MS2	No	Lipid Identification Software	MS-DIAL
MS1 verified by standard	Yes	Data manipulation	Smoothing, Centroiding
MS2 verified by standard	Yes	Nomenclature for intact lipid molecule	Yes
Background check at MS1	Yes	Nomenclature for fragment ions	No
Background check at MS2	No	Further identification remarks	The reverse dot product similarity value, where the in silico spectrum is used for the library template, is used as the correlation coefficient value. The candidates are ranked by the reverse dot product score and the candidate with the highest similarity value is described as the representative C=C isomer candidate for the EAD-MS/MS spectrum.

Fragment name
-HG(PE,141)
-FA1(-H)
-FA2+(C3H5O2)
HG+
HG + C3H6+
HG + C2H4O+

13) PE P[M+H]+ / Lipid quantification

Quantitative	No	Batch correction	No
Normalization to reference	No	Further quantification remarks	-

14) Hemibismonoacylglycerophosphate (HBMP)[M+NH4]+ / Lipid identification

Lipid class	Hemibismonoacylglycerophosphate (HBMP)	Did you presume assumptions for identification?	No
Derivatization	-	Check isomer overlap	No
MS Level for identification	MS1, MS2	RT verified by standard	Yes
Identification level	Molecular species level	Separation of isobaric/isomeric interferece confirmed	Yes
Polarity mode	Positive	Model for separation prediction	Yes
Type of positive (precursor)ion	[M+NH4]+	Additional dimension/techniques	EAD
Fragments for identification		How was/were the additional dimension(s) used?	To determine sn- positions

Fragment name
Dehydro-monoacyl glycerols
Dehydro-diacyl glycerol
NL of FA

Isotope correction at MS1	No	Was a model used to predict lipid molecule separation?	No
Isotope correction at MS2	No	Lipid Identification Software	MS-DIAL
MS1 verified by standard	No	Data manipulation	Smoothing, Centroiding
MS2 verified by standard	No	Nomenclature for intact lipid molecule	Yes
Background check at MS1	Yes	Nomenclature for fragment ions	No
Background check at MS2	No	Further identification remarks	The reverse dot product similarity value, where the in silico spectrum is used for the library template, is used as the correlation coefficient value. The candidates are ranked by the reverse dot product score and the candidate with the highest similarity value is described as the representative C=C isomer candidate for the EAD-MS/MS spectrum.

14) Hemibismonoacylglycerophosphate (HBMP)[M+NH4]+ / Lipid quantification

Quantitative	No	Batch correction	No
Normalization to reference	No	Further quantification remarks	-

15) Lysodiacylglyceryl hydroxymethyl-N,N,N-trimethyl-beta-alanine (LDGTA)[M+H]+ / Lipid identification

Lipid class	Lysodiacylglyceryl hydroxymethyl-N,N,N-trimethyl-beta-alanine (LDGTA)	Did you presume assumptions for identification?	No
Derivatization	-	Check isomer overlap	No
MS Level for identification	MS1, MS2	RT verified by standard	Yes
Identification level	Double bond position	Separation of isobaric/isomeric interferece confirmed	Yes
Polarity mode	Positive	Model for separation prediction	Yes
Type of positive (precursor)ion	[M+H]+	Additional dimension/techniques	EAD
Fragments for identification	Fragment name: Characteristic fragment (C7H14NO2+); HG + C2H4O+	How was/were the additional dimension(s) used?	To determine doublebond positions
Isotope correction at MS1	No	Was a model used to predict lipid molecule separation?	No
Isotope correction at MS2	No	Lipid Identification Software	MS-DIAL
MS1 verified by standard	No	Data manipulation	Smoothing, Centroiding
MS2 verified by standard	No	Nomenclature for intact lipid molecule	Yes
Background check at MS1	Yes	Nomenclature for fragment ions	No
Background check at MS2	No	Further identification remarks	The reverse dot product similarity value, where the in silico spectrum is used for the library template, is used as the correlation coefficient value. The candidates are ranked by the reverse dot product score and the candidate with the highest similarity value is described as the representative C=C isomer candidate for the EAD-MS/MS spectrum.

15) Lysodiacylglyceryl hydroxymethyl-N,N,N-trimethyl-beta-alanine (LDGTA)[M+H]+ / Lipid quantification

Quantitative	No	Batch correction	No
Normalization to reference	No	Further quantification remarks	-

16) Lysoiacylglyceryl trimethylhomoserine (LDGTS)[M+H]+ / Lipid identification

Lipid class	Lysoiacylglyceryl trimethylhomoserine (LDGTS)	Did you presume assumptions for identification?	No
Derivatization	-	Check isomer overlap	No
MS Level for identification	MS1, MS2	RT verified by standard	Yes
Identification level	Double bond position	Separation of isobaric/isomeric interferece confirmed	Yes
Polarity mode	Positive	Model for separation prediction	Yes
Type of positive (precursor)ion	[M+H]+	Additional dimension/techniques	EAD
Fragments for identification		How was/were the additional dimension(s) used?	To determine doublebond positions
Isotope correction at MS1	No	Was a model used to predict lipid molecule separation?	No
Isotope correction at MS2	No	Lipid Identification Software	MS-DIAL
MS1 verified by standard	No	Data manipulation	Smoothing, Centroiding
MS2 verified by standard	No	Nomenclature for intact lipid molecule	Yes
Background check at MS1	Yes	Nomenclature for fragment ions	No
Background check at MS2	No	Further identification remarks	The reverse dot product similarity value, where the in silico spectrum is used for the library template, is used as the correlation coefficient value. The candidates are ranked by the reverse dot product score and the candidate with the highest similarity value is described as the representative C=C isomer candidate for the EAD-MS/MS spectrum.

Fragments for identification:

Fragment name
Characteristic fragment ($C6H12NO2+$)
HG + $C2H4O+$

16) Lysoiacylglyceryl trimethylhomoserine (LDGTS)[M+H]+ / Lipid quantification

Quantitative	No	Batch correction	No
Normalization to reference	No	Further quantification remarks	-

17) LPC[M+H]+ / Lipid identification

Lipid class	LPC	Did you presume assumptions for identification?	No
Derivatization	-	Check isomer overlap	No
MS Level for identification	MS1, MS2	RT verified by standard	Yes
Identification level	Double bond position	Separation of isobaric/isomeric interferece confirmed	Yes
Polarity mode	Positive	Model for separation prediction	Yes
Type of positive (precursor)ion	[M+H]+	Additional dimension/techniques	EAD
Fragments for identification		How was/were the additional dimension(s) used?	To determine sn- and doublebond positions

Fragment name
HG(PC,184)
(C5H13NO,104)
HG + C3H5
HG + C2H3O
NL of FA

Isotope correction at MS1	No	Was a model used to predict lipid molecule separation?	No
Isotope correction at MS2	No	Lipid Identification Software	MS-DIAL
MS1 verified by standard	Yes	Data manipulation	Smoothing, Centroiding
MS2 verified by standard	Yes	Nomenclature for intact lipid molecule	Yes
Background check at MS1	Yes	Nomenclature for fragment ions	No
Background check at MS2	No	Further identification remarks	The reverse dot product similarity value, where the in silico spectrum is used for the library template, is used as the correlation coefficient value. The candidates are ranked by the reverse dot product score and the candidate with the highest similarity value is described as the representative C=C isomer candidate for the EAD-MS/MS spectrum.

17) LPC[M+H]+ / Lipid quantification

Quantitative	No	Batch correction	No
Normalization to reference	No	Further quantification remarks	-

18) LPE[M+H]+ / Lipid identification

Lipid class	LPE	Did you presume assumptions for identification?	No
Derivatization	-	Check isomer overlap	No
MS Level for identification	MS1, MS2	RT verified by standard	Yes
Identification level	Double bond position	Separation of isobaric/isomeric interferece confirmed	Yes
Polarity mode	Positive	Model for separation prediction	Yes
Type of positive (precursor)ion	[M+H]+	Additional dimension/techniques	EAD
Fragments for identification	Fragment name: -HG(PE,141); HG + C3H6+; HG + C2H4O+; HG+; NL of FA	How was/were the additional dimension(s) used?	To determine sn- and doublebond positions
Isotope correction at MS1	No	Was a model used to predict lipid molecule separation?	No
Isotope correction at MS2	No	Lipid Identification Software	MS-DIAL
MS1 verified by standard	Yes	Data manipulation	Smoothing, Centroiding
MS2 verified by standard	Yes	Nomenclature for intact lipid molecule	Yes
Background check at MS1	Yes	Nomenclature for fragment ions	No
Background check at MS2	No	Further identification remarks	The reverse dot product similarity value, where the in silico spectrum is used for the library template, is used as the correlation coefficient value. The candidates are ranked by the reverse dot product score and the candidate with the highest similarity value is described as the representative C=C isomer candidate for the EAD-MS/MS spectrum.

18) LPE[M+H]+ / Lipid quantification

Quantitative	No	Batch correction	No
Normalization to reference	No	Further quantification remarks	-

19) LPG[M+H]+ / Lipid identification

Lipid class	LPG	Did you presume assumptions for identification?	No
Derivatization	-	Check isomer overlap	No
MS Level for identification	MS1, MS2	RT verified by standard	Yes
Identification level	sn Position	Separation of isobaric/isomeric interferece confirmed	Yes
Polarity mode	Positive	Model for separation prediction	Yes
Type of positive (precursor)ion	[M+H]+	Additional dimension/techniques	EAD
Fragments for identification		How was/were the additional dimension(s) used?	To determine sn- position

Fragment name
-HG(PG,172)
HG + C3H6+
HG + C2H4O+
HG+
HG+ -H2O
NL of FA

Isotope correction at MS1	No	Was a model used to predict lipid molecule separation?	No
Isotope correction at MS2	No	Lipid Identification Software	MS-DIAL
MS1 verified by standard	Yes	Data manipulation	Smoothing, Centroiding
MS2 verified by standard	Yes	Nomenclature for intact lipid molecule	Yes
Background check at MS1	Yes	Nomenclature for fragment ions	No
Background check at MS2	No	Further identification remarks	The reverse dot product similarity value, where the in silico spectrum is used for the library template, is used as the correlation coefficient value. The candidates are ranked by the reverse dot product score and the candidate with the highest similarity value is described as the representative C=C isomer candidate for the EAD-MS/MS spectrum.

19) LPG[M+H]+ / Lipid quantification

Quantitative	No	Batch correction	No
Normalization to reference	No	Further quantification remarks	-

20) LPI[M+NH4]+ / Lipid identification

Lipid class	LPI	Did you presume assumptions for identification?	No
Derivatization	-	Check isomer overlap	No
MS Level for identification	MS1, MS2	RT verified by standard	Yes
Identification level	sn Position	Separation of isobaric/isomeric interferece confirmed	Yes
Polarity mode	Positive	Model for separation prediction	Yes
Type of positive (precursor)ion	[M+NH4]+	Additional dimension/techniques	EAD
Fragments for identification		How was/were the additional dimension(s) used?	To determine sn- position

Fragment name
-(H2O,18)
GP(155) + H2O
-HG(PI,260)
NL of FA
HG + C3H6+
HG + C2H4O+

Isotope correction at MS1	No	Was a model used to predict lipid molecule separation?	No
Isotope correction at MS2	No	Lipid Identification Software	MS-DIAL
MS1 verified by standard	Yes	Data manipulation	Smoothing, Centroiding
MS2 verified by standard	Yes	Nomenclature for intact lipid molecule	Yes
Background check at MS1	Yes	Nomenclature for fragment ions	No
Background check at MS2	No	Further identification remarks	The reverse dot product similarity value, where the in silico spectrum is used for the library template, is used as the correlation coefficient value. The candidates are ranked by the reverse dot product score and the candidate with the highest similarity value is described as the representative C=C isomer candidate for the EAD-MS/MS spectrum.

20) LPI[M+NH4]+ / Lipid quantification

Quantitative	No	Batch correction	No
Normalization to reference	No	Further quantification remarks	-

21) LPS[M+H]+ / Lipid identification

Lipid class	LPS	Did you presume assumptions for identification?	No
Derivatization	-	Check isomer overlap	No
MS Level for identification	MS1, MS2	RT verified by standard	Yes
Identification level	Double bond position	Separation of isobaric/isomeric interferece confirmed	Yes
Polarity mode	Positive	Model for separation prediction	Yes
Type of positive (precursor)ion	[M+H]+	Additional dimension/techniques	EAD
Fragments for identification	Fragment name: -(C3H7NO3,105); HG+; HG + C3H6+; HG + C2H4O+; NL of FA	How was/were the additional dimension(s) used?	To determine sn- and doublebond positions
Isotope correction at MS1	No	Was a model used to predict lipid molecule separation?	No
Isotope correction at MS2	No	Lipid Identification Software	MS-DIAL
MS1 verified by standard	Yes	Data manipulation	Smoothing, Centroiding
MS2 verified by standard	Yes	Nomenclature for intact lipid molecule	Yes
Background check at MS1	Yes	Nomenclature for fragment ions	No
Background check at MS2	No	Further identification remarks	The reverse dot product similarity value, where the in silico spectrum is used for the library template, is used as the correlation coefficient value. The candidates are ranked by the reverse dot product score and the candidate with the highest similarity value is described as the representative C=C isomer candidate for the EAD-MS/MS spectrum.

Fragment name
-(C3H7NO3,105)
HG+
HG + C3H6+
HG + C2H4O+
NL of FA

21) LPS[M+H]+ / Lipid quantification

Quantitative	No	Batch correction	No
Normalization to reference	No	Further quantification remarks	-

22) PC[M+H]+ / Lipid identification

Lipid class	PC	Did you presume assumptions for identification?	No
Derivatization	-	Check isomer overlap	No
MS Level for identification	MS1, MS2	RT verified by standard	Yes
Identification level	Double bond position	Separation of isobaric/isomeric interferece confirmed	Yes
Polarity mode	Positive	Model for separation prediction	Yes
Type of positive (precursor)ion	[M+H]+	Additional dimension/techniques	EAD
Fragments for identification		How was/were the additional dimension(s) used?	To determine sn- and doublebond positions
Isotope correction at MS1	No	Was a model used to predict lipid molecule separation?	No
Isotope correction at MS2	No	Lipid Identification Software	MS-DIAL
MS1 verified by standard	Yes	Data manipulation	Smoothing, Centroiding
MS2 verified by standard	Yes	Nomenclature for intact lipid molecule	Yes
Background check at MS1	Yes	Nomenclature for fragment ions	No
Background check at MS2	No	Further identification remarks	The reverse dot product similarity value, where the in silico spectrum is used for the library template, is used as the correlation coefficient value. The candidates are ranked by the reverse dot product score and the candidate with the highest similarity value is described as the representative C=C isomer candidate for the EAD-MS/MS spectrum.

Fragment name
HG(PC,184)
HG + C3H5
HG + C2H3O
NL of HG
NL of FA

22) PC[M+H]+ / Lipid quantification

Quantitative	No	Batch correction	No
Normalization to reference	No	Further quantification remarks	-

23) PC[M+Na]+ / Lipid identification

Lipid class	PC	Did you presume assumptions for identification?	No
Derivatization	-	Check isomer overlap	No
MS Level for identification	MS1, MS2	RT verified by standard	Yes
Identification level	Double bond position	Separation of isobaric/isomeric interferece confirmed	Yes
Polarity mode	Positive	Model for separation prediction	Yes
Type of positive (precursor)ion	[M+Na]+	Additional dimension/techniques	EAD
Fragments for identification		How was/were the additional dimension(s) used?	To determine sn- and doublebond positions

Fragment name
HG(PC,184) + Na+
HG + C3H5Na
HG + C2H3ONa
NL of HG
NL of FA
NL of C3H9N

Isotope correction at MS1	No	Was a model used to predict lipid molecule separation?	No
Isotope correction at MS2	No	Lipid Identification Software	MS-DIAL
MS1 verified by standard	Yes	Data manipulation	Smoothing, Centroiding
MS2 verified by standard	Yes	Nomenclature for intact lipid molecule	Yes
Background check at MS1	Yes	Nomenclature for fragment ions	No
Background check at MS2	No	Further identification remarks	The reverse dot product similarity value, where the in silico spectrum is used for the library template, is used as the correlation coefficient value. The candidates are ranked by the reverse dot product score and the candidate with the highest similarity value is described as the representative C=C isomer candidate for the EAD-MS/MS spectrum.

23) PC[M+Na]+ / Lipid quantification

Quantitative	No	Batch correction	No
Normalization to reference	No	Further quantification remarks	-

24) PE[M+H]+ / Lipid identification

Lipid class	PE	Did you presume assumptions for identification?	No
Derivatization	-	Check isomer overlap	No
MS Level for identification	MS1, MS2	RT verified by standard	Yes
Identification level	Double bond position	Separation of isobaric/isomeric interferece confirmed	Yes
Polarity mode	Positive	Model for separation prediction	Yes
Type of positive (precursor)ion	[M+H]+	Additional dimension/techniques	EAD
Fragments for identification		How was/were the additional dimension(s) used?	To determine sn- and doublebond positions
Isotope correction at MS1	No	Was a model used to predict lipid molecule separation?	No
Isotope correction at MS2	No	Lipid Identification Software	MS-DIAL
MS1 verified by standard	Yes	Data manipulation	Smoothing, Centroiding
MS2 verified by standard	Yes	Nomenclature for intact lipid molecule	Yes
Background check at MS1	Yes	Nomenclature for fragment ions	No
Background check at MS2	No	Further identification remarks	The reverse dot product similarity value, where the in silico spectrum is used for the library template, is used as the correlation coefficient value. The candidates are ranked by the reverse dot product score and the candidate with the highest similarity value is described as the representative C=C isomer candidate for the EAD-MS/MS spectrum.

Fragment name
-HG(PE,141)
HG+
HG + C3H6+
HG + C2H4O+
NL of FA
NL of HG and FA

24) PE[M+H]+ / Lipid quantification

Quantitative	No	Batch correction	No
Normalization to reference	No	Further quantification remarks	-

25) PE[M+Na]+ / Lipid identification

Lipid class	PE	Did you presume assumptions for identification?	No
Derivatization	-	Check isomer overlap	No
MS Level for identification	MS1, MS2	RT verified by standard	Yes
Identification level	Double bond position	Separation of isobaric/isomeric interferece confirmed	Yes
Polarity mode	Positive	Model for separation prediction	Yes
Type of positive (precursor)ion	[M+Na]+	Additional dimension/techniques	EAD
Fragments for identification		How was/were the additional dimension(s) used?	To determine sn- and doublebond positions

Fragment name
-HG(PE,141)
HG+Na+
HG + C3H5Na+
HG + C2H3ONa+
NL of FA

Isotope correction at MS1	No	Was a model used to predict lipid molecule separation?	No
Isotope correction at MS2	No	Lipid Identification Software	MS-DIAL
MS1 verified by standard	Yes	Data manipulation	Smoothing, Centroiding
MS2 verified by standard	Yes	Nomenclature for intact lipid molecule	Yes
Background check at MS1	Yes	Nomenclature for fragment ions	No
Background check at MS2	No	Further identification remarks	The reverse dot product similarity value, where the in silico spectrum is used for the library template, is used as the correlation coefficient value. The candidates are ranked by the reverse dot product score and the candidate with the highest similarity value is described as the representative C=C isomer candidate for the EAD-MS/MS spectrum.

25) PE[M+Na]+ / Lipid quantification

Quantitative	No	Batch correction	No
Normalization to reference	No	Further quantification remarks	-

26) PG[M+NH4]+ / Lipid identification

Lipid class	PG	Did you presume assumptions for identification?	No
Derivatization	-	Check isomer overlap	No
MS Level for identification	MS1, MS2	RT verified by standard	Yes
Identification level	Double bond position	Separation of isobaric/isomeric interferece confirmed	Yes
Polarity mode	Positive	Model for separation prediction	Yes
Type of positive (precursor)ion	[M+NH4]+	Additional dimension/techniques	EAD
Fragments for identification		How was/were the additional dimension(s) used?	To determine doublebond positions

Fragment name
-HG(PG,172)
HG+
NL of FA
NL of HG and FA
HG + C3H6+
HG + C2H4O+

Isotope correction at MS1	No	Was a model used to predict lipid molecule separation?	No
Isotope correction at MS2	No	Lipid Identification Software	MS-DIAL
MS1 verified by standard	Yes	Data manipulation	Smoothing, Centroiding
MS2 verified by standard	Yes	Nomenclature for intact lipid molecule	Yes
Background check at MS1	Yes	Nomenclature for fragment ions	No
Background check at MS2	No	Further identification remarks	The reverse dot product similarity value, where the in silico spectrum is used for the library template, is used as the correlation coefficient value. The candidates are ranked by the reverse dot product score and the candidate with the highest similarity value is described as the representative C=C isomer candidate for the EAD-MS/MS spectrum.

26) PG[M+NH4]+ / Lipid quantification

Quantitative	No	Batch correction	No
Normalization to reference	No	Further quantification remarks	-

27) PG[M+Na]+ / Lipid identification

Lipid class	PG	Did you presume assumptions for identification?	No
Derivatization	-	Check isomer overlap	No
MS Level for identification	MS1, MS2	RT verified by standard	Yes
Identification level	Double bond position	Separation of isobaric/isomeric interferece confirmed	Yes
Polarity mode	Positive	Model for separation prediction	Yes
Type of positive (precursor)ion	[M+Na]+	Additional dimension/techniques	EAD
Fragments for identification		How was/were the additional dimension(s) used?	To determine sn- and doublebond positions
Isotope correction at MS1	No	Was a model used to predict lipid molecule separation?	No
Isotope correction at MS2	No	Lipid Identification Software	MS-DIAL
MS1 verified by standard	Yes	Data manipulation	Smoothing, Centroiding
MS2 verified by standard	Yes	Nomenclature for intact lipid molecule	Yes
Background check at MS1	Yes	Nomenclature for fragment ions	No
Background check at MS2	No	Further identification remarks	The reverse dot product similarity value, where the in silico spectrum is used for the library template, is used as the correlation coefficient value. The candidates are ranked by the reverse dot product score and the candidate with the highest similarity value is described as the representative C=C isomer candidate for the EAD-MS/MS spectrum.

Fragment name
-HG(PG,172)
HG + Na+
NL of FA
HG + C3H5Na+
HG + C2H3ONa+

27) PG[M+Na]+ / Lipid quantification

Quantitative	No	Batch correction	No
Normalization to reference	No	Further quantification remarks	-

28) PI[M+NH4]+ / Lipid identification

Lipid class	PI	Did you presume assumptions for identification?	No
Derivatization	-	Check isomer overlap	No
MS Level for identification	MS1, MS2	RT verified by standard	Yes
Identification level	Double bond position	Separation of isobaric/isomeric interferece confirmed	Yes
Polarity mode	Positive	Model for separation prediction	Yes
Type of positive (precursor)ion	[M+NH4]+	Additional dimension/techniques	EAD
Fragments for identification		How was/were the additional dimension(s) used?	To determine doublebond positions

Fragment name
HG+
HG + C3H5
HG + C2H3O
NL of HG and FA
NL of FA
NL of inositol
-HG(PI,260)

Isotope correction at MS1	No	Was a model used to predict lipid molecule separation?	No
Isotope correction at MS2	No	Lipid Identification Software	MS-DIAL
MS1 verified by standard	Yes	Data manipulation	Smoothing, Centroiding
MS2 verified by standard	Yes	Nomenclature for intact lipid molecule	Yes
Background check at MS1	Yes	Nomenclature for fragment ions	No
Background check at MS2	No	Further identification remarks	The reverse dot product similarity value, where the in silico spectrum is used for the library template, is used as the correlation coefficient value. The candidates are ranked by the reverse dot product score and the candidate with the highest similarity value is described as the representative C=C isomer candidate for the EAD-MS/MS spectrum.

28) PI[M+NH4]+ / Lipid quantification

Quantitative	No	Batch correction	No
Normalization to reference	No	Further quantification remarks	-

29) PI[M+Na]+ / Lipid identification

Lipid class	PI	Did you presume assumptions for identification?	No
Derivatization	-	Check isomer overlap	No
MS Level for identification	MS1, MS2	RT verified by standard	Yes
Identification level	sn Position	Separation of isobaric/isomeric interferece confirmed	Yes
Polarity mode	Positive	Model for separation prediction	Yes
Type of positive (precursor)ion	[M+Na]+	Additional dimension/techniques	EAD
Fragments for identification		How was/were the additional dimension(s) used?	To determine sn- position

Fragment name
HG + Na+
HG + C3H5Na+
HG + C2H3ONa+
NL of FA
NL of inositol

Isotope correction at MS1	No	Was a model used to predict lipid molecule separation?	No
Isotope correction at MS2	No	Lipid Identification Software	MS-DIAL
MS1 verified by standard	Yes	Data manipulation	Smoothing, Centroiding
MS2 verified by standard	Yes	Nomenclature for intact lipid molecule	Yes
Background check at MS1	Yes	Nomenclature for fragment ions	No
Background check at MS2	No	Further identification remarks	The reverse dot product similarity value, where the in silico spectrum is used for the library template, is used as the correlation coefficient value. The candidates are ranked by the reverse dot product score and the candidate with the highest similarity value is described as the representative C=C isomer candidate for the EAD-MS/MS spectrum.

29) PI[M+Na]+ / Lipid quantification

Quantitative	No	Batch correction	No
Normalization to reference	No	Further quantification remarks	-

30) PS[M+H]+ / Lipid identification

Lipid class	PS	Did you presume assumptions for identification?	No
Derivatization	-	Check isomer overlap	No
MS Level for identification	MS1, MS2	RT verified by standard	Yes
Identification level	Double bond position	Separation of isobaric/isomeric interferece confirmed	Yes
Polarity mode	Positive	Model for separation prediction	Yes
Type of positive (precursor)ion	[M+H]+	Additional dimension/techniques	EAD
Fragments for identification		How was/were the additional dimension(s) used?	To determine doublebond positions
Isotope correction at MS1	No	Was a model used to predict lipid molecule separation?	No
Isotope correction at MS2	No	Lipid Identification Software	MS-DIAL
MS1 verified by standard	Yes	Data manipulation	Smoothing, Centroiding
MS2 verified by standard	Yes	Nomenclature for intact lipid molecule	Yes
Background check at MS1	Yes	Nomenclature for fragment ions	No
Background check at MS2	No	Further identification remarks	The reverse dot product similarity value, where the in silico spectrum is used for the library template, is used as the correlation coefficient value. The candidates are ranked by the reverse dot product score and the candidate with the highest similarity value is described as the representative C=C isomer candidate for the EAD-MS/MS spectrum.

Fragment name
-HG(PS,185)
HG+
NL of FA
NL of HG and FA
HG + C3H6+
HG + C2H4O+

30) PS[M+H]+ / Lipid quantification

Quantitative	No	Batch correction	No
Normalization to reference	No	Further quantification remarks	-

31) PS[M+Na]+ / Lipid identification

Lipid class	PS	Did you presume assumptions for identification?	No
Derivatization	-	Check isomer overlap	No
MS Level for identification	MS1, MS2	RT verified by standard	Yes
Identification level	sn Position	Separation of isobaric/isomeric interferece confirmed	Yes
Polarity mode	Positive	Model for separation prediction	Yes
Type of positive (precursor)ion	[M+Na]+	Additional dimension/techniques	EAD
Fragments for identification		How was/were the additional dimension(s) used?	To determine sn- position

Fragment name
-HG(PS,185)
HG + Na+
NL of FA
HG + C3H5Na+
HG + C2H3ONa+
NL of HG and FA

Isotope correction at MS1	No	Was a model used to predict lipid molecule separation?	No
Isotope correction at MS2	No	Lipid Identification Software	MS-DIAL
MS1 verified by standard	Yes	Data manipulation	Smoothing, Centroiding
MS2 verified by standard	Yes	Nomenclature for intact lipid molecule	Yes
Background check at MS1	Yes	Nomenclature for fragment ions	No
Background check at MS2	No	Further identification remarks	The reverse dot product similarity value, where the in silico spectrum is used for the library template, is used as the correlation coefficient value. The candidates are ranked by the reverse dot product score and the candidate with the highest similarity value is described as the representative C=C isomer candidate for the EAD-MS/MS spectrum.

31) PS[M+Na]+ / Lipid quantification

Quantitative	No	Batch correction	No
Normalization to reference	No	Further quantification remarks	-

32) TG[M+NH4]+ / Lipid identification

Lipid class	TG	Did you presume assumptions for identification?	No
Derivatization	-	Check isomer overlap	No
MS Level for identification	MS1, MS2	RT verified by standard	Yes
Identification level	Double bond position	Separation of isobaric/isomeric interferece confirmed	Yes
Polarity mode	Positive	Model for separation prediction	Yes
Type of positive (precursor)ion	[M+NH4]+	Additional dimension/techniques	EAD
Fragments for identification		How was/were the additional dimension(s) used?	To determine doublebond positions
Isotope correction at MS1	No	Was a model used to predict lipid molecule separation?	No
Isotope correction at MS2	No	Lipid Identification Software	MS-DIAL
MS1 verified by standard	Yes	Data manipulation	Smoothing, Centroiding
MS2 verified by standard	Yes	Nomenclature for intact lipid molecule	Yes
Background check at MS1	Yes	Nomenclature for fragment ions	No
Background check at MS2	No	Further identification remarks	The reverse dot product similarity value, where the in silico spectrum is used for the library template, is used as the correlation coefficient value. The candidates are ranked by the reverse dot product score and the candidate with the highest similarity value is described as the representative C=C isomer candidate for the EAD-MS/MS spectrum.

Fragment name
NL of FA
FA + C3H5O2+
Fatty Acyl

32) TG[M+NH4]+ / Lipid quantification

Quantitative	No	Batch correction	No
Normalization to reference	No	Further quantification remarks	-

33) TG[M+Na]+ / Lipid identification

Lipid class	TG	Did you presume assumptions for identification?	No
Derivatization	-	Check isomer overlap	No
MS Level for identification	MS1, MS2	RT verified by standard	Yes
Identification level	Double bond position	Separation of isobaric/isomeric interferece confirmed	Yes
Polarity mode	Positive	Model for separation prediction	Yes
Type of positive (precursor)ion	[M+Na]+	Additional dimension/techniques	EAD
Fragments for identification	**Fragment name**<br>NL of FA<br>Fatty Acyl<br>FA + C3H5ONa+<br>FA + C3H3O2Na+	How was/were the additional dimension(s) used?	To determine sn- and doublebond positions
Isotope correction at MS1	No	Was a model used to predict lipid molecule separation?	No
Isotope correction at MS2	No	Lipid Identification Software	MS-DIAL
MS1 verified by standard	Yes	Data manipulation	Smoothing, Centroiding
MS2 verified by standard	Yes	Nomenclature for intact lipid molecule	Yes
Background check at MS1	Yes	Nomenclature for fragment ions	No
Background check at MS2	No	Further identification remarks	The reverse dot product similarity value, where the in silico spectrum is used for the library template, is used as the correlation coefficient value. The candidates are ranked by the reverse dot product score and the candidate with the highest similarity value is described as the representative C=C isomer candidate for the EAD-MS/MS spectrum.

33) TG[M+Na]+ / Lipid quantification

Quantitative	No	Batch correction	No
Normalization to reference	No	Further quantification remarks	-

34) Ceramide non-hydroxyfatty acid-sphingosine (Cer_NS)[M+H]+ / Lipid identification

Lipid class	Ceramide non-hydroxyfatty acid-sphingosine (Cer_NS)	Did you presume assumptions for identification?	No
Derivatization	-	Check isomer overlap	No
MS Level for identification	MS1, MS2	RT verified by standard	Yes
Identification level	Double bond position	Separation of isobaric/isomeric interferece confirmed	Yes
Polarity mode	Positive	Model for separation prediction	Yes
Type of positive (precursor)ion	[M+H]+	Additional dimension/techniques	EAD
Fragments for identification		How was/were the additional dimension(s) used?	To determine doublebond positions

Fragment name
Neutral loss of H2O
Neutral loss of 2H2O
Neutral loss of CH4O2
Fatty acyl amide +C2H3O+ fragment
Fatty acyl amide +C2H2+ fragment
Fatty acyl amide+ fragment
Sphingosine -CH4O2 fragment
Sphingosine -2H2O fragment
Sphingosine -H2O fragment

Isotope correction at MS1	No	Was a model used to predict lipid molecule separation?	No
Isotope correction at MS2	No	Lipid Identification Software	MS-DIAL
MS1 verified by standard	Yes	Data manipulation	Smoothing, Centroiding
MS2 verified by standard	Yes	Nomenclature for intact lipid molecule	Yes
Background check at MS1	Yes	Nomenclature for fragment ions	No
Background check at MS2	No	Further identification remarks	The reverse dot product similarity value, where the in silico spectrum is used for the library template, is used as the correlation coefficient value. The candidates are ranked by the reverse dot product score and the candidate with the highest similarity value is described as the representative C=C isomer candidate for the EAD-MS/MS spectrum.

34) Ceramide non-hydroxyfatty acid-sphingosine (Cer_NS)[M+H]+ / Lipid quantification

Quantitative	No	Batch correction	No
Normalization to reference	No	Further quantification remarks	-

35) Ceramide non-hydroxyfatty acid-sphingosine (Cer_NS)[M+Na]+ / Lipid identification

Lipid class	Ceramide non-hydroxyfatty acid-sphingosine (Cer_NS)	Did you presume assumptions for identification?	No
Derivatization	-	Check isomer overlap	No
MS Level for identification	MS1, MS2	RT verified by standard	Yes
Identification level	Double bond position	Separation of isobaric/isomeric interferece confirmed	Yes
Polarity mode	Positive	Model for separation prediction	Yes
Type of positive (precursor)ion	[M+Na]+	Additional dimension/techniques	EAD
Fragments for identification		How was/were the additional dimension(s) used?	To determine doublebond positions

Fragment name
Neutral loss of CH3O
Fatty acyl amide +C2H2ONa fragment

The reverse dot product similarity value, where the in silico spectrum is used for the library template, is used as the correlation coefficient value. The candidates are ranked by the reverse dot product score and the candidate with the highest similarity value is described as the representative C=C isomer candidate for the EAD-MS/MS spectrum.

Isotope correction at MS1	No	Was a model used to predict lipid molecule separation?	No
Isotope correction at MS2	No	Lipid Identification Software	MS-DIAL
MS1 verified by standard	Yes	Data manipulation	Smoothing, Centroiding
MS2 verified by standard	Yes	Nomenclature for intact lipid molecule	Yes
Background check at MS1	Yes	Nomenclature for fragment ions	No
Background check at MS2	No	Further identification remarks	The reverse dot product similarity value, where the in silico spectrum is used for the library template, is used as the correlation coefficient value. The candidates are ranked by the reverse dot product score and the candidate with the highest similarity value is described as the representative C=C isomer candidate for the EAD-MS/MS spectrum.

35) Ceramide non-hydroxyfatty acid-sphingosine (Cer_NS)[M+Na]+ / Lipid quantification

Quantitative	No	Batch correction	No
Normalization to reference	No	Further quantification remarks	-

36) Ceramide non-hydroxyfatty acid-dihydrosphingosine (Cer_NDS)[M+H]+ / Lipid identification

Lipid class	Ceramide non-hydroxyfatty acid-dihydrosphingosine (Cer_NDS)	Did you presume assumptions for identification?	No
Derivatization	-	Check isomer overlap	No
MS Level for identification	MS1, MS2	RT verified by standard	Yes
Identification level	Double bond position	Separation of isobaric/isomeric interferece confirmed	Yes
Polarity mode	Positive	Model for separation prediction	Yes
Type of positive (precursor)ion	[M+H]+	Additional dimension/techniques	EAD
Fragments for identification		How was/were the additional dimension(s) used?	To determine doublebond positions
Isotope correction at MS1	No	Was a model used to predict lipid molecule separation?	No
Isotope correction at MS2	No	Lipid Identification Software	MS-DIAL
MS1 verified by standard	No	Data manipulation	Smoothing, Centroiding
MS2 verified by standard	No	Nomenclature for intact lipid molecule	Yes
Background check at MS1	Yes	Nomenclature for fragment ions	No
Background check at MS2	No	Further identification remarks	The reverse dot product similarity value, where the in silico spectrum is used for the library template, is used as the correlation coefficient value. The candidates are ranked by the reverse dot product score and the candidate with the highest similarity value is described as the representative C=C isomer candidate for the EAD-MS/MS spectrum.

Fragment name
Neutral loss of H2O
Neutral loss of 2H2O
Neutral loss of CH4O2
Fatty acyl amide +C2H3O+ fragment
Fatty acyl amide +C2H2+ fragment
Fatty acyl amide+ fragment
Sphingosine -CH4O2 fragment
Sphingosine -2H2O fragment
Sphingosine -H2O fragment

36) Ceramide non-hydroxyfatty acid-dihydrosphingosine (Cer_NDS)[M+H]+ / Lipid quantification

Quantitative	No	Batch correction	No
Normalization to reference	No	Further quantification remarks	-

37) Ceramide non-hydroxyfatty acid-dihydrosphingosine (Cer_NDS)[M+Na]+ / Lipid identification

Lipid class	Ceramide non-hydroxyfatty acid-dihydrosphingosine (Cer_NDS)	Did you presume assumptions for identification?	No
Derivatization	-	Check isomer overlap	No
MS Level for identification	MS1, MS2	RT verified by standard	Yes
Identification level	Double bond position	Separation of isobaric/isomeric interfernce confirmed	Yes
Polarity mode	Positive	Model for separation prediction	Yes
Type of positive (precursor)ion	[M+Na]+	Additional dimension/techniques	EAD
Fragments for identification		How was/were the additional dimension(s) used?	To determine doublebond positions

Fragment name
Neutral loss of CH3O
Fatty acyl amide +C2H2ONa fragment

The reverse dot product similarity value, where the in silico spectrum is used for the library template, is used as the correlation coefficient value. The candidates are ranked by the reverse dot product score and the candidate with the highest similarity value is described as the representative C=C isomer candidate for the EAD-MS/MS spectrum.

Isotope correction at MS1	No	Was a model used to predict lipid molecule separation?	No
Isotope correction at MS2	No	Lipid Identification Software	MS-DIAL
MS1 verified by standard	No	Data manipulation	Smoothing, Centroiding
MS2 verified by standard	No	Nomenclature for intact lipid molecule	Yes
Background check at MS1	Yes	Nomenclature for fragment ions	No
Background check at MS2	No	Further identification remarks	The reverse dot product similarity value, where the in silico spectrum is used for the library template, is used as the correlation coefficient value. The candidates are ranked by the reverse dot product score and the candidate with the highest similarity value is described as the representative C=C isomer candidate for the EAD-MS/MS spectrum.

37) Ceramide non-hydroxyfatty acid-dihydrosphingosine (Cer_NDS)[M+Na]+ / Lipid quantification

Quantitative	No	Batch correction	No
Normalization to reference	No	Further quantification remarks	-

38) Ceramide non-hydroxyfatty acid-phytospingosine(Cer_NP)[M+H]+ / Lipid identification

Lipid class	Ceramide non-hydroxyfatty acid-phytospingosine(Cer_NP)	Did you presume assumptions for identification?	No
Derivatization	-	Check isomer overlap	No
MS Level for identification	MS1, MS2	RT verified by standard	Yes
Identification level	Double bond position	Separation of isobaric/isomeric interferece confirmed	Yes
Polarity mode	Positive	Model for separation prediction	Yes
Type of positive (precursor)ion	[M+H]+	Additional dimension/techniques	EAD
Fragments for identification		How was/were the additional dimension(s) used?	To determine doublebond positions

Fragment name
Neutral loss of H2O
Neutral loss of 2H2O
Neutral loss of CH4O2
Fatty acyl amide +C2H3O+ fragment
Fatty acyl amide +C2H2+ fragment
Fatty acyl amide+ fragment
Phytosphingosine -CH4O2 fragment
Phytosphingosine -2H2O fragment
Phytosphingosine -H2O fragment
Fatty acyl amide +C3H4O+ fragment
Fatty acyl amide +C3H5O2+ fragment
Phytosphingosine -CH6O2 fragment

Isotope correction at MS1	No	Was a model used to predict lipid molecule separation?	No
Isotope correction at MS2	No	Lipid Identification Software	MS-DIAL
MS1 verified by standard	Yes	Data manipulation	Smoothing, Centroiding
MS2 verified by standard	Yes	Nomenclature for intact lipid molecule	Yes
Background check at MS1	Yes	Nomenclature for fragment ions	No
Background check at MS2	No	Further identification remarks	The reverse dot product similarity value, where the in silico spectrum is used for the library template, is used as the correlation coefficient value. The candidates are ranked by the reverse dot product score and the candidate with the highest similarity value is described as the representative C=C isomer candidate for the EAD-MS/MS spectrum.

38) Ceramide non-hydroxyfatty acid-phytospingosine(Cer_NP)[M+H]+ / Lipid quantification

Quantitative	No	Batch correction	No
Normalization to reference	No	Further quantification remarks	-

39) Ceramide alpha-hydroxy fatty acid-sphingosine (Cer_AS)[M+H]+ / Lipid identification

Lipid class	Ceramide alpha-hydroxy fatty acid-sphingosine (Cer_AS)	Did you presume assumptions for identification?	No
Derivatization	-	Check isomer overlap	No
MS Level for identification	MS1, MS2	RT verified by standard	Yes
Identification level	Double bond position	Separation of isobaric/isomeric interferece confirmed	Yes
Polarity mode	Positive	Model for separation prediction	Yes
Type of positive (precursor)ion	[M+H]+	Additional dimension/techniques	EAD
Fragments for identification		How was/were the additional dimension(s) used?	To determine doublebond positions

Fragment name
Neutral loss of H2O
Neutral loss of 2H2O
Neutral loss of CH4O2
Alpha-hydroxy fatty acyl amide +C2H3O+ fragment
Alpha-hydroxy fatty acyl amide +C2H2+ fragment
Alpha-hydroxy fatty acyl amide+ fragment
Sphingosine -CH4O2 fragment
Sphingosine -2H2O fragment
Sphingosine -H2O fragment

Isotope correction at MS1	No	Was a model used to predict lipid molecule separation?	No
Isotope correction at MS2	No	Lipid Identification Software	MS-DIAL
MS1 verified by standard	Yes	Data manipulation	Smoothing, Centroiding
MS2 verified by standard	Yes	Nomenclature for intact lipid molecule	Yes
Background check at MS1	Yes	Nomenclature for fragment ions	No
Background check at MS2	No	Further identification remarks	The reverse dot product similarity value, where the in silico spectrum is used for the library template, is used as the correlation coefficient value. The candidates are ranked by the reverse dot product score and the candidate with the highest similarity value is described as the representative C=C isomer candidate for the EAD-MS/MS spectrum.

39) Ceramide alpha-hydroxy fatty acid-sphingosine (Cer_AS)[M+H]+ / Lipid quantification

Quantitative	No	Batch correction	No
Normalization to reference	No	Further quantification remarks	-

40) Hexosylceramide non-hydroxyfatty acid-sphingosine (HexCer_NS)[M+H]+ / Lipid identification

Lipid class	Hexosylceramide non-hydroxyfatty acid-sphingosine (HexCer_NS)	Did you presume assumptions for identification?	No
Derivatization	-	Check isomer overlap	No
MS Level for identification	MS1, MS2	RT verified by standard	Yes
Identification level	Double bond position	Separation of isobaric/isomeric interferece confirmed	Yes
Polarity mode	Positive	Model for separation prediction	Yes
Type of positive (precursor)ion	[M+H]+	Additional dimension/techniques	EAD
Fragments for identification		How was/were the additional dimension(s) used?	To determine doublebond positions

Fragment name
Neutral loss of H2O
Neutral loss of hexose
Neutral loss of hexose and H2O
Neutral loss of hexose and 2H2O
Fatty acyl amide +C2H5O + hexose fragment
Fatty acyl amide +C2H3O+ fragment
Fatty acyl amide +C2H2+ fragment
Fatty acyl amide+ fragment
Sphingosine -CH4O2 fragment
Sphingosine -2H2O fragment
Sphingosine -H2O fragment
Hexose + C2H5N+

Isotope correction at MS1	No	Was a model used to predict lipid molecule separation?	No
Isotope correction at MS2	No	Lipid Identification Software	MS-DIAL
MS1 verified by standard	Yes	Data manipulation	Smoothing, Centroiding
MS2 verified by standard	Yes	Nomenclature for intact lipid molecule	Yes
Background check at MS1	Yes	Nomenclature for fragment ions	No
Background check at MS2	No	Further identification remarks	The reverse dot product similarity value, where the in silico spectrum is used for the library template, is used as the correlation coefficient value. The candidates are ranked by the reverse dot product score and the candidate with the highest similarity value is described as the representative C=C isomer candidate for the EAD-MS/MS spectrum.

40) Hexosylceramide non-hydroxyfatty acid-sphingosine (HexCer_NS)[M+H]+ / Lipid quantification

Quantitative	No	Batch correction	No
Normalization to reference	No	Further quantification remarks	-

41) Hexosylceramide non-hydroxyfatty acid-sphingosine (HexCer_NS)[M+Na]+ / Lipid identification

Lipid class	Hexosylceramide non-hydroxyfatty acid-sphingosine (HexCer_NS)	Did you presume assumptions for identification?	No
Derivatization	-	Check isomer overlap	No
MS Level for identification	MS1, MS2	RT verified by standard	Yes
Identification level	Double bond position	Separation of isobaric/isomeric interferece confirmed	Yes
Polarity mode	Positive	Model for separation prediction	Yes
Type of positive (precursor)ion	[M+Na]+	Additional dimension/techniques	EAD
Fragments for identification		How was/were the additional dimension(s) used?	To determine doublebond positions

Fragment name
Neutral loss of C5H10O4
Neutral loss of hexose and H2O
Fatty acyl amide +C2H3ONa+ fragment
Fatty acyl amide +C2HNa+ fragment
Fatty acyl amide+ fragment
Hexose + C2H5NNa+
C5H9O4Na+

Isotope correction at MS1	No	Was a model used to predict lipid molecule separation?	No
Isotope correction at MS2	No	Lipid Identification Software	MS-DIAL
MS1 verified by standard	Yes	Data manipulation	Smoothing, Centroiding
MS2 verified by standard	Yes	Nomenclature for intact lipid molecule	Yes
Background check at MS1	Yes	Nomenclature for fragment ions	No
Background check at MS2	No	Further identification remarks	The reverse dot product similarity value, where the in silico spectrum is used for the library template, is used as the correlation coefficient value. The candidates are ranked by the reverse dot product score and the candidate with the highest similarity value is described as the representative C=C isomer candidate for the EAD-MS/MS spectrum.

41) Hexosylceramide non-hydroxyfatty acid-sphingosine (HexCer_NS)[M+Na]+ / Lipid quantification

Quantitative	No	Batch correction	No
Normalization to reference	No	Further quantification remarks	-

42) Di-hexosylceramide hydroxyfatty acid-sphingosine (Hex2Cer_NS)[M+H]+ / Lipid identification

Lipid class	Di-hexosylceramide hydroxyfatty acid-sphingosine (Hex2Cer_NS)	Did you presume assumptions for identification?	No
Derivatization	-	Check isomer overlap	No
MS Level for identification	MS1, MS2	RT verified by standard	Yes
Identification level	Molecular species level	Separation of isobaric/isomeric interferece confirmed	Yes
Polarity mode	Positive	Model for separation prediction	Yes
Type of positive (precursor)ion	[M+H]+	Additional dimension/techniques	EAD
Fragments for identification		How was/were the additional dimension(s) used?	To determine OH positions

Fragment name
Neutral loss of H2O
Neutral loss of 2hexose
Neutral loss of 2hexose and H2O
Neutral loss of 2hexose and 2H2O
Fatty acyl amide +C2H5O + 2hexose fragment
Fatty acyl amide +C2H3O+ fragment
Fatty acyl amide +C2H2+ fragment
Fatty acyl amide+ fragment
Sphingosine -CH4O2 fragment
Sphingosine -2H2O fragment
Sphingosine -H2O fragment

Isotope correction at MS1	No	Was a model used to predict lipid molecule separation?	No
Isotope correction at MS2	No	Lipid Identification Software	MS-DIAL
MS1 verified by standard	Yes	Data manipulation	Smoothing, Centroiding
MS2 verified by standard	Yes	Nomenclature for intact lipid molecule	Yes
Background check at MS1	Yes	Nomenclature for fragment ions	No
Background check at MS2	No	Further identification remarks	The reverse dot product similarity value, where the in silico spectrum is used for the library template, is used as the correlation coefficient value. The candidates are ranked by the reverse dot product score and the candidate with the highest similarity value is described as the representative C=C isomer candidate for the EAD-MS/MS spectrum.

42) Di-hexosylceramide hydroxyfatty acid-sphingosine (Hex2Cer_NS)[M+H]+ / Lipid quantification

Quantitative	No	Batch correction	No
Normalization to reference	No	Further quantification remarks	-

43) SHexCer[M+H]+ / Lipid identification

Lipid class	SHexCer	Did you presume assumptions for identification?	No
Derivatization	-	Check isomer overlap	No
MS Level for identification	MS1, MS2	RT verified by standard	Yes
Identification level	Molecular species level	Separation of isobaric/isomeric interferece confirmed	Yes
Polarity mode	Positive	Model for separation prediction	Yes
Type of positive (precursor)ion	[M+H]+	Additional dimension/techniques	EAD
Fragments for identification		How was/were the additional dimension(s) used?	To determine OH positions

Fragment name
Neutral loss of SO3
-HG(SHex,98)
-HG(SHex,260)
-HG(SHex,278)
-HG(SHex,242)
Fatty acyl amide +C2H2+ fragment
Fatty acyl amide+ fragment
Sphingosine -2H2O fragment
Sphingosine -H2O fragment
Sphingosine -CH4O2 fragment
Hexose + C2H5N+

Isotope correction at MS1	No	Was a model used to predict lipid molecule separation?	No
Isotope correction at MS2	No	Lipid Identification Software	MS-DIAL
MS1 verified by standard	Yes	Data manipulation	Smoothing, Centroiding
MS2 verified by standard	Yes	Nomenclature for intact lipid molecule	Yes
Background check at MS1	Yes	Nomenclature for fragment ions	No
Background check at MS2	No	Further identification remarks	The reverse dot product similarity value, where the in silico spectrum is used for the library template, is used as the correlation coefficient value. The candidates are ranked by the reverse dot product score and the candidate with the highest similarity value is described as the representative C=C isomer candidate for the EAD-MS/MS spectrum.

43) SHexCer[M+H]+ / Lipid quantification

Quantitative	No	Batch correction	No
Normalization to reference	No	Further quantification remarks	-

44) SHexCer[M+Na]+ / Lipid identification

Lipid class	SHexCer	Did you presume assumptions for identification?	No
Derivatization	-	Check isomer overlap	No
MS Level for identification	MS1, MS2	RT verified by standard	Yes
Identification level	Molecular species level	Separation of isobaric/isomeric interferece confirmed	Yes
Polarity mode	Positive	Model for separation prediction	Yes
Type of positive (precursor)ion	[M+Na]+	Additional dimension/techniques	EAD
Fragments for identification		How was/were the additional dimension(s) used?	To determine OH positions

Fragment name
Fatty acyl amide +C2H2Na+ fragment
Fatty acyl amide +H3Na+ fragment
SHex + C2H4NNa+
Hexose + C2H4NNa+
Hexose + Na+
Neutral loss of SO3
-HG(SHex,98)
NL of C5H10O7S
NL of C6H10O8S
-HG(SHex,260)

Isotope correction at MS1	No	Was a model used to predict lipid molecule separation?	No
Isotope correction at MS2	No	Lipid Identification Software	MS-DIAL
MS1 verified by standard	Yes	Data manipulation	Smoothing, Centroiding
MS2 verified by standard	Yes	Nomenclature for intact lipid molecule	Yes
Background check at MS1	Yes	Nomenclature for fragment ions	No
Background check at MS2	No	Further identification remarks	The reverse dot product similarity value, where the in silico spectrum is used for the library template, is used as the correlation coefficient value. The candidates are ranked by the reverse dot product score and the candidate with the highest similarity value is described as the representative C=C isomer candidate for the EAD-MS/MS spectrum.

44) SHexCer[M+Na]+ / Lipid quantification

Quantitative	No	Batch correction	No
Normalization to reference	No	Further quantification remarks	-

45) SM[M+H]+ / Lipid identification

Lipid class	SM	Did you presume assumptions for identification?	No
Derivatization	-	Check isomer overlap	No
MS Level for identification	MS1, MS2	RT verified by standard	Yes
Identification level	Double bond position	Separation of isobaric/isomeric interferece confirmed	Yes
Polarity mode	Positive	Model for separation prediction	Yes
Type of positive (precursor)ion	[M+H]+	Additional dimension/techniques	EAD
Fragments for identification		How was/were the additional dimension(s) used?	To determine doublebond positions

Fragment name
NL of Fatty acyl and NH4
Fatty acyl amide +C2H2 + Header fragment
Fatty acyl amide +C2H2+ fragment
Sphingosine -2H2O fragment
Header + C3H4NO
Header + C2H4N
HG(PC,184)

Isotope correction at MS1	No	Was a model used to predict lipid molecule separation?	No
Isotope correction at MS2	No	Lipid Identification Software	MS-DIAL
MS1 verified by standard	Yes	Data manipulation	Smoothing, Centroiding
MS2 verified by standard	Yes	Nomenclature for intact lipid molecule	Yes
Background check at MS1	Yes	Nomenclature for fragment ions	No
Background check at MS2	No	Further identification remarks	The reverse dot product similarity value, where the in silico spectrum is used for the library template, is used as the correlation coefficient value. The candidates are ranked by the reverse dot product score and the candidate with the highest similarity value is described as the representative C=C isomer candidate for the EAD-MS/MS spectrum.

45) SM[M+H]+ / Lipid quantification

Quantitative	No	Batch correction	No
Normalization to reference	No	Further quantification remarks	-

46) SM[M+Na]+ / Lipid identification

Lipid class	SM	Did you presume assumptions for identification?	No
Derivatization	-	Check isomer overlap	No
MS Level for identification	MS1, MS2	RT verified by standard	Yes
Identification level	Double bond position	Separation of isobaric/isomeric interferece confirmed	Yes
Polarity mode	Positive	Model for separation prediction	Yes
Type of positive (precursor)ion	[M+Na]+	Additional dimension/techniques	EAD
Fragments for identification		How was/were the additional dimension(s) used?	To determine doublebond positions

Fragment name
NL of C3H9N
NL of C5H11N
NL of HG
Fatty acyl amide +C2H + header +Na fragment
Fatty acyl amide +C2H +Na fragment
Header + C2H4NNa
HG(PC,184) + Na

Isotope correction at MS1	No	Was a model used to predict lipid molecule separation?	No
Isotope correction at MS2	No	Lipid Identification Software	MS-DIAL
MS1 verified by standard	Yes	Data manipulation	Smoothing, Centroiding
MS2 verified by standard	Yes	Nomenclature for intact lipid molecule	Yes
Background check at MS1	Yes	Nomenclature for fragment ions	No
Background check at MS2	No	Further identification remarks	The reverse dot product similarity value, where the in silico spectrum is used for the library template, is used as the correlation coefficient value. The candidates are ranked by the reverse dot product score and the candidate with the highest similarity value is described as the representative C=C isomer candidate for the EAD-MS/MS spectrum.

46) SM[M+Na]+ / Lipid quantification

Quantitative	No	Batch correction	No
Normalization to reference	No	Further quantification remarks	-

Note 2. Lipidomics minimal reporting checklist for characterization of very long chain PUFA (VLC-PUFA) containing PC in the eye tissue of mice using EAD.

Separation Workflow

Created by https://lipidomicstandards.org, version v2.3.2

Overall study design

Title of the study	Characterization of very long chain PUFA (VLC-PUFA) containing PC in the eye tissue of mice using EAD		
Document creation date	02/07/2024	Corresponding Email	htsugawa@go.tuat.ac.jp
Principle investigator	Hiroshi Tsugawa	Is the workflow targeted or untargeted?	Untargeted
Institution	Tokyo University of Agriculture and Technology	Clinical	No

Lipid extraction

Extraction method	2-phase system	2-phase system	Bligh&Dyer
pH adjustment	None	Were internal standards added prior extraction?	No

Analytical platform

Which solvents were used	(A) acetonitrile (ACN):MeOH:H2O (1:1:3, v/v/v) and (B) ACN:IPA (1:9, v/v). Both the solvents contained 10 nM ethylenediaminetetraacetic acid and 5 mM ammonium acetate.	Mass resolution for detected ion at MS1	High resolution
Number of separation dimensions	One dimension	Resolution at m/z 200 at MS1	26316
Separation type 1	LC	Mass accuracy in ppm at MS1	1.13
Separation mode 1 (liquid)	RP	Mass window for precursor ion isolation (in Da total isolation window)	1
Detector	Mass spectrometer	Mass resolution for detected ion at MS2	High resolution
MS type	QTOF	Resolution at m/z 200 at MS2	28538
MS vendor	SCIEX	Mass accuracy in ppm at MS2	0.76
Ion source	ESI	Was/Were additional dimension/techniques used	No
MS Level	MS1, MS2		

Quality control

Blanks	Yes	Quality control	No
Type of Blanks	Extraction blank		

Method qualification and validation

Method validation	Yes	Precison	Yes
Lipid recovery	No	Accuracy	Yes
Dynamic quantification range	Yes	Guidelines followed	None
Limit of quantitation (LOQ)/Limit of detection (LOD)	Yes		

Reporting

Are reported raw data uploaded into repository?	Yes	Summary data	Identification data
Link to repository / ID to entry	http://prime.psc.riken.jp/menta.cgi/prime/drop_index, DM0054	Raw data upload	Yes
Are metadata available?	Yes	Additional comments	The resolution and accuracy (ppm) for MS1 are those of m/z 132.9049 which were determined by the mass calibration in SCIEX OS. In addition, the resolution and accuracy for MS2 are those of m/z 185.1284, which were also determined by the mass calibration in SCIEX OS.

Sample Descriptions

Mouse eye ball / Mouse / Tissues (e.g., liver, heart, brain)

Perfusion	No	Freeze-thaw cycles	0
Provided information	Time to separate plasma/serum (min), Time to freeze (min), Storage time (month), Freeze-thaw cycles	Additives	None
Temperature handling original sample	4-8 °C	Were samples stored under inert gas?	No
Instant sample preparation	No	Additional preservation methods	No
Time to freeze (min)	10	Biobank samples	No
Snap freezing in liquid N2	Yes	Sample homogenization	Yes
Storage temperature	-80 °C	Sample homogenization solvent	Methanol
Storage time (month)	1		

Lipid Class Descriptions

1) Acylcarnitine (CAR)[M+H]+ / Lipid identification

Lipid class	Acylcarnitine (CAR)	Did you presume assumptions for identification?	No
Derivatization	-	Check isomer overlap	No
MS Level for identification	MS1, MS2	RT verified by standard	No
Identification level	Double bond position	Separation of isobaric/isomeric interferece confirmed	No
Polarity mode	Positive	Model for separation prediction	No
Type of positive (precursor)ion	[M+H]+	Additional dimension/techniques	EAD
Fragments for identification<br>**Fragment name**<br>Characteristic fragment (C4H5O2+)		How was/were the additional dimension(s) used?	To determine doublebond positions
Isotope correction at MS1	No	Was a model used to predict lipid molecule separation?	No
Isotope correction at MS2	No	Lipid Identification Software	MS-DIAL
MS1 verified by standard	Yes	Data manipulation	Smoothing, Centroiding
MS2 verified by standard	Yes	Nomenclature for intact lipid molecule	Yes
Background check at MS1	Yes	Nomenclature for fragment ions	No
Background check at MS2	No	Further identification remarks	The reverse dot product similarity value, where the in silico spectrum is used for the library template, is used as the correlation coefficient value. The candidates are ranked by the reverse dot product score and the candidate with the highest similarity value is described as the representative C=C isomer candidate for the EAD-MS/MS spectrum.

1) Acylcarnitine (CAR)[M+H]+ / Lipid quantification

Quantitative	No	Batch correction	No
Normalization to reference	No	Further quantification remarks	-

2) DG[M+NH4]+ / Lipid identification

Lipid class	DG	Did you presume assumptions for identification?	No
Derivatization	-	Check isomer overlap	No
MS Level for identification	MS1, MS2	RT verified by standard	No
Identification level	Double bond position	Separation of isobaric/isomeric interferece confirmed	No
Polarity mode	Positive	Model for separation prediction	No
Type of positive (precursor)ion	[M+NH4]+	Additional dimension/techniques	EAD
Fragments for identification	**Fragment name**<br>-(H2O+NH3,35)<br>The reverse dot product similarity value, where the in silico spectrum is used for the library template, is used as the correlation coefficient value. The candidates are ranked by the reverse dot product score and the candidate with the highest similarity value is described as the representative C=C isomer candidate for the EAD-MS/MS spectrum.<br>-FA1(-H)-(H2O+NH3)<br>-FA2(-H)-(H2O+NH3)	How was/were the additional dimension(s) used?	To determine doublebond positions
Isotope correction at MS1	No	Was a model used to predict lipid molecule separation?	No
Isotope correction at MS2	No	Lipid Identification Software	MS-DIAL
MS1 verified by standard	Yes	Data manipulation	Smoothing, Centroiding
MS2 verified by standard	Yes	Nomenclature for intact lipid molecule	Yes
Background check at MS1	Yes	Nomenclature for fragment ions	No
Background check at MS2	No	Further identification remarks	The reverse dot product similarity value, where the in silico spectrum is used for the library template, is used as the correlation coefficient value. The candidates are ranked by the reverse dot product score and the candidate with the highest similarity value is described as the representative C=C isomer candidate for the EAD-MS/MS spectrum.

2) DG[M+NH4]+ / Lipid quantification

Quantitative	No	Batch correction	No
Normalization to reference	No	Further quantification remarks	-

3) PC O[M+H]+ / Lipid identification

Lipid class	PC O	Did you presume assumptions for identification?	No
Derivatization	-	Check isomer overlap	No
MS Level for identification	MS1, MS2	RT verified by standard	No
Identification level	Double bond position	Separation of isobaric/isomeric interferece confirmed	No
Polarity mode	Positive	Model for separation prediction	No
Type of positive (precursor)ion	[M+H]+	Additional dimension/techniques	EAD
Fragments for identification		How was/were the additional dimension(s) used?	To determine sn- and doublebond positions

Fragment name
HG(PC,184)
NL of FA
NL of Ether
HG + C3H5
HG + C2H3O

Isotope correction at MS1	No	Was a model used to predict lipid molecule separation?	No
Isotope correction at MS2	No	Lipid Identification Software	MS-DIAL
MS1 verified by standard	Yes	Data manipulation	Smoothing, Centroiding
MS2 verified by standard	Yes	Nomenclature for intact lipid molecule	Yes
Background check at MS1	Yes	Nomenclature for fragment ions	No
Background check at MS2	No	Further identification remarks	The reverse dot product similarity value, where the in silico spectrum is used for the library template, is used as the correlation coefficient value. The candidates are ranked by the reverse dot product score and the candidate with the highest similarity value is described as the representative C=C isomer candidate for the EAD-MS/MS spectrum.

3) PC O[M+H]+ / Lipid quantification

Quantitative	No	Batch correction	No
Normalization to reference	No	Further quantification remarks	-

4) PC P[M+H]+ / Lipid identification

Lipid class	PC P	Did you presume assumptions for identification?	No
Derivatization	-	Check isomer overlap	No
MS Level for identification	MS1, MS2	RT verified by standard	No
Identification level	Double bond position	Separation of isobaric/isomeric interferece confirmed	No
Polarity mode	Positive	Model for separation prediction	No
Type of positive (precursor)ion	[M+H]+	Additional dimension/techniques	EAD
Fragments for identification	Fragment name: HG(PC,184); NL of FA; NL of Ether; HG + C3H5; HG + C2H3O	How was/were the additional dimension(s) used?	To determine sn- and doublebond positions
Isotope correction at MS1	No	Was a model used to predict lipid molecule separation?	No
Isotope correction at MS2	No	Lipid Identification Software	MS-DIAL
MS1 verified by standard	Yes	Data manipulation	Smoothing, Centroiding
MS2 verified by standard	Yes	Nomenclature for intact lipid molecule	Yes
Background check at MS1	Yes	Nomenclature for fragment ions	No
Background check at MS2	No	Further identification remarks	The reverse dot product similarity value, where the in silico spectrum is used for the library template, is used as the correlation coefficient value. The candidates are ranked by the reverse dot product score and the candidate with the highest similarity value is described as the representative C=C isomer candidate for the EAD-MS/MS spectrum.

4) PC P[M+H]+ / Lipid quantification

Quantitative	No	Batch correction	No
Normalization to reference	No	Further quantification remarks	-

5) PE O[M+H]+ / Lipid identification

Lipid class	PE O	Did you presume assumptions for identification?	No
Derivatization	-	Check isomer overlap	No
MS Level for identification	MS1, MS2	RT verified by standard	No
Identification level	Double bond position	Separation of isobaric/isomeric interferece confirmed	No
Polarity mode	Positive	Model for separation prediction	No
Type of positive (precursor)ion	[M+H]+	Additional dimension/techniques	EAD
Fragments for identification		How was/were the additional dimension(s) used?	To determine doublebond positions

Fragment name
-HG(PE,141)
NL of FA
NL of Ether
HG + C3H6+
HG + C2H4O+

Isotope correction at MS1	No	Was a model used to predict lipid molecule separation?	No
Isotope correction at MS2	No	Lipid Identification Software	MS-DIAL
MS1 verified by standard	Yes	Data manipulation	Smoothing, Centroiding
MS2 verified by standard	Yes	Nomenclature for intact lipid molecule	Yes
Background check at MS1	Yes	Nomenclature for fragment ions	No
Background check at MS2	No	Further identification remarks	The reverse dot product similarity value, where the in silico spectrum is used for the library template, is used as the correlation coefficient value. The candidates are ranked by the reverse dot product score and the candidate with the highest similarity value is described as the representative C=C isomer candidate for the EAD-MS/MS spectrum.

5) PE O[M+H]+ / Lipid quantification

Quantitative	No	Batch correction	No
Normalization to reference	No	Further quantification remarks	-

6) PE P[M+H]+ / Lipid identification

Lipid class	PE P	Did you presume assumptions for identification?	No
Derivatization	-	Check isomer overlap	No
MS Level for identification	MS1, MS2	RT verified by standard	No
Identification level	Double bond position	Separation of isobaric/isomeric interferece confirmed	No
Polarity mode	Positive	Model for separation prediction	No
Type of positive (precursor)ion	[M+H]+	Additional dimension/techniques	EAD
Fragments for identification		How was/were the additional dimension(s) used?	To determine doublebond positions

Fragment name
-HG(PE,141)
-FA1(-H)
-FA2+(C3H5O2)
HG+
HG + C3H6+
HG + C2H4O+

Isotope correction at MS1	No	Was a model used to predict lipid molecule separation?	No
Isotope correction at MS2	No	Lipid Identification Software	MS-DIAL
MS1 verified by standard	Yes	Data manipulation	Smoothing, Centroiding
MS2 verified by standard	Yes	Nomenclature for intact lipid molecule	Yes
Background check at MS1	Yes	Nomenclature for fragment ions	No
Background check at MS2	No	Further identification remarks	The reverse dot product similarity value, where the in silico spectrum is used for the library template, is used as the correlation coefficient value. The candidates are ranked by the reverse dot product score and the candidate with the highest similarity value is described as the representative C=C isomer candidate for the EAD-MS/MS spectrum.

6) PE P[M+H]+ / Lipid quantification

Quantitative	No	Batch correction	No
Normalization to reference	No	Further quantification remarks	-

7) LPC[M+H]+ / Lipid identification

Lipid class	LPC	Did you presume assumptions for identification?	No
Derivatization	-	Check isomer overlap	No
MS Level for identification	MS1, MS2	RT verified by standard	No
Identification level	Double bond position	Separation of isobaric/isomeric interferece confirmed	No
Polarity mode	Positive	Model for separation prediction	No
Type of positive (precursor)ion	[M+H]+	Additional dimension/techniques	EAD
Fragments for identification		How was/were the additional dimension(s) used?	To determine sn- and doublebond positions

Fragment name
HG(PC,184)
(C5H13NO,104)
HG + C3H5
HG + C2H3O
NL of FA

Isotope correction at MS1	No	Was a model used to predict lipid molecule separation?	No
Isotope correction at MS2	No	Lipid Identification Software	MS-DIAL
MS1 verified by standard	Yes	Data manipulation	Smoothing, Centroiding
MS2 verified by standard	Yes	Nomenclature for intact lipid molecule	Yes
Background check at MS1	Yes	Nomenclature for fragment ions	No
Background check at MS2	No	Further identification remarks	The reverse dot product similarity value, where the in silico spectrum is used for the library template, is used as the correlation coefficient value. The candidates are ranked by the reverse dot product score and the candidate with the highest similarity value is described as the representative C=C isomer candidate for the EAD-MS/MS spectrum.

7) LPC[M+H]+ / Lipid quantification

Quantitative	No	Batch correction	No
Normalization to reference	No	Further quantification remarks	-

8) LPE[M+H]+ / Lipid identification

Lipid class	LPE	Did you presume assumptions for identification?	No
Derivatization	-	Check isomer overlap	No
MS Level for identification	MS1, MS2	RT verified by standard	No
Identification level	Double bond position	Separation of isobaric/isomeric interferece confirmed	No
Polarity mode	Positive	Model for separation prediction	No
Type of positive (precursor)ion	[M+H]+	Additional dimension/techniques	EAD
Fragments for identification	Fragment name: -HG(PE,141); HG + C3H6+; HG + C2H4O+; HG+; NL of FA	How was/were the additional dimension(s) used?	To determine sn- and doublebond positions
Isotope correction at MS1	No	Was a model used to predict lipid molecule separation?	No
Isotope correction at MS2	No	Lipid Identification Software	MS-DIAL
MS1 verified by standard	Yes	Data manipulation	Smoothing, Centroiding
MS2 verified by standard	Yes	Nomenclature for intact lipid molecule	Yes
Background check at MS1	Yes	Nomenclature for fragment ions	No
Background check at MS2	No	Further identification remarks	The reverse dot product similarity value, where the in silico spectrum is used for the library template, is used as the correlation coefficient value. The candidates are ranked by the reverse dot product score and the candidate with the highest similarity value is described as the representative C=C isomer candidate for the EAD-MS/MS spectrum.

8) LPE[M+H]+ / Lipid quantification

Quantitative	No	Batch correction	No
Normalization to reference	No	Further quantification remarks	-

9) PC[M+H]+ / Lipid identification

Lipid class	PC	Did you presume assumptions for identification?	No
Derivatization	-	Check isomer overlap	No
MS Level for identification	MS1, MS2	RT verified by standard	No
Identification level	Double bond position	Separation of isobaric/isomeric interferece confirmed	No
Polarity mode	Positive	Model for separation prediction	No
Type of positive (precursor)ion	[M+H]+	Additional dimension/techniques	EAD
Fragments for identification		How was/were the additional dimension(s) used?	To determine sn- and doublebond positions

Fragment name
HG(PC,184)
HG + C3H5
HG + C2H3O
NL of HG
NL of FA

Isotope correction at MS1	No	Was a model used to predict lipid molecule separation?	No
Isotope correction at MS2	No	Lipid Identification Software	MS-DIAL
MS1 verified by standard	Yes	Data manipulation	Smoothing, Centroiding
MS2 verified by standard	Yes	Nomenclature for intact lipid molecule	Yes
Background check at MS1	Yes	Nomenclature for fragment ions	No
Background check at MS2	No	Further identification remarks	The reverse dot product similarity value, where the in silico spectrum is used for the library template, is used as the correlation coefficient value. The candidates are ranked by the reverse dot product score and the candidate with the highest similarity value is described as the representative C=C isomer candidate for the EAD-MS/MS spectrum.

9) PC[M+H]+ / Lipid quantification

Quantitative	No	Batch correction	No
Normalization to reference	No	Further quantification remarks	-

10) PE[M+H]+ / Lipid identification

Lipid class	PE	Did you presume assumptions for identification?	No
Derivatization	-	Check isomer overlap	No
MS Level for identification	MS1, MS2	RT verified by standard	No
Identification level	Double bond position	Separation of isobaric/isomeric interferece confirmed	No
Polarity mode	Positive	Model for separation prediction	No
Type of positive (precursor)ion	[M+H]+	Additional dimension/techniques	EAD
Fragments for identification	Fragment name: -HG(PE,141); HG+; HG + C3H6+; HG + C2H4O+; NL of FA; NL of HG and FA	How was/were the additional dimension(s) used?	To determine sn- and doublebond positions
Isotope correction at MS1	No	Was a model used to predict lipid molecule separation?	No
Isotope correction at MS2	No	Lipid Identification Software	MS-DIAL
MS1 verified by standard	Yes	Data manipulation	Smoothing, Centroiding
MS2 verified by standard	Yes	Nomenclature for intact lipid molecule	Yes
Background check at MS1	Yes	Nomenclature for fragment ions	No
Background check at MS2	No	Further identification remarks	The reverse dot product similarity value, where the in silico spectrum is used for the library template, is used as the correlation coefficient value. The candidates are ranked by the reverse dot product score and the candidate with the highest similarity value is described as the representative C=C isomer candidate for the EAD-MS/MS spectrum.

10) PE[M+H]+ / Lipid quantification

Quantitative	No	Batch correction	No
Normalization to reference	No	Further quantification remarks	-

11) PG[M+NH4]+ / Lipid identification

Lipid class	PG	Did you presume assumptions for identification?	No
Derivatization	-	Check isomer overlap	No
MS Level for identification	MS1, MS2	RT verified by standard	No
Identification level	Double bond position	Separation of isobaric/isomeric interferece confirmed	No
Polarity mode	Positive	Model for separation prediction	No
Type of positive (precursor)ion	[M+NH4]+	Additional dimension/techniques	EAD
Fragments for identification		How was/were the additional dimension(s) used?	To determine doublebond positions

Fragment name
-HG(PG,172)
HG+
NL of FA
NL of HG and FA
HG + C3H6+
HG + C2H4O+

Isotope correction at MS1	No	Was a model used to predict lipid molecule separation?	No
Isotope correction at MS2	No	Lipid Identification Software	MS-DIAL
MS1 verified by standard	Yes	Data manipulation	Smoothing, Centroiding
MS2 verified by standard	Yes	Nomenclature for intact lipid molecule	Yes
Background check at MS1	Yes	Nomenclature for fragment ions	No
Background check at MS2	No	Further identification remarks	The reverse dot product similarity value, where the in silico spectrum is used for the library template, is used as the correlation coefficient value. The candidates are ranked by the reverse dot product score and the candidate with the highest similarity value is described as the representative C=C isomer candidate for the EAD-MS/MS spectrum.

11) PG[M+NH4]+ / Lipid quantification

Quantitative	No	Batch correction	No
Normalization to reference	No	Further quantification remarks	-

12) PI[M+NH4]+ / Lipid identification

Lipid class	PI	Did you presume assumptions for identification?	No
Derivatization	-	Check isomer overlap	No
MS Level for identification	MS1, MS2	RT verified by standard	No
Identification level	Double bond position	Separation of isobaric/isomeric interferece confirmed	No
Polarity mode	Positive	Model for separation prediction	No
Type of positive (precursor)ion	[M+NH4]+	Additional dimension/techniques	EAD
Fragments for identification		How was/were the additional dimension(s) used?	To determine doublebond positions

Fragment name
HG+
HG + C3H5
HG + C2H3O
NL of HG and FA
NL of FA
NL of inositol
-HG(PI,260)

Isotope correction at MS1	No	Was a model used to predict lipid molecule separation?	No
Isotope correction at MS2	No	Lipid Identification Software	MS-DIAL
MS1 verified by standard	Yes	Data manipulation	Smoothing, Centroiding
MS2 verified by standard	Yes	Nomenclature for intact lipid molecule	Yes
Background check at MS1	Yes	Nomenclature for fragment ions	No
Background check at MS2	No	Further identification remarks	The reverse dot product similarity value, where the in silico spectrum is used for the library template, is used as the correlation coefficient value. The candidates are ranked by the reverse dot product score and the candidate with the highest similarity value is described as the representative C=C isomer candidate for the EAD-MS/MS spectrum.

12) PI[M+NH4]+ / Lipid quantification

Quantitative	No	Batch correction	No
Normalization to reference	No	Further quantification remarks	-

13) PS[M+H]+ / Lipid identification

Lipid class	PS	Did you presume assumptions for identification?	No
Derivatization	-	Check isomer overlap	No
MS Level for identification	MS1, MS2	RT verified by standard	No
Identification level	Double bond position	Separation of isobaric/isomeric interferece confirmed	No
Polarity mode	Positive	Model for separation prediction	No
Type of positive (precursor)ion	[M+H]+	Additional dimension/techniques	EAD
Fragments for identification		How was/were the additional dimension(s) used?	To determine doublebond positions
Isotope correction at MS1	No	Was a model used to predict lipid molecule separation?	No
Isotope correction at MS2	No	Lipid Identification Software	MS-DIAL
MS1 verified by standard	Yes	Data manipulation	Smoothing, Centroiding
MS2 verified by standard	Yes	Nomenclature for intact lipid molecule	Yes
Background check at MS1	Yes	Nomenclature for fragment ions	No
Background check at MS2	No	Further identification remarks	The reverse dot product similarity value, where the in silico spectrum is used for the library template, is used as the correlation coefficient value. The candidates are ranked by the reverse dot product score and the candidate with the highest similarity value is described as the representative C=C isomer candidate for the EAD-MS/MS spectrum.

Fragment name
-HG(PS,185)
HG+
NL of FA
NL of HG and FA
HG + C3H6+
HG + C2H4O+

13) PS[M+H]+ / Lipid quantification

Quantitative	No	Batch correction	No
Normalization to reference	No	Further quantification remarks	-

14) TG[M+NH4]+ / Lipid identification

Lipid class	TG	Did you presume assumptions for identification?	No
Derivatization	-	Check isomer overlap	No
MS Level for identification	MS1, MS2	RT verified by standard	No
Identification level	Double bond position	Separation of isobaric/isomeric interferece confirmed	No
Polarity mode	Positive	Model for separation prediction	No
Type of positive (precursor)ion	[M+NH4]+	Additional dimension/techniques	EAD
Fragments for identification	Fragment name: NL of FA; FA + C3H5O2+; Fatty Acyl	How was/were the additional dimension(s) used?	To determine doublebond positions
Isotope correction at MS1	No	Was a model used to predict lipid molecule separation?	No
Isotope correction at MS2	No	Lipid Identification Software	MS-DIAL
MS1 verified by standard	Yes	Data manipulation	Smoothing, Centroiding
MS2 verified by standard	Yes	Nomenclature for intact lipid molecule	Yes
Background check at MS1	Yes	Nomenclature for fragment ions	No
Background check at MS2	No	Further identification remarks	The reverse dot product similarity value, where the in silico spectrum is used for the library template, is used as the correlation coefficient value. The candidates are ranked by the reverse dot product score and the candidate with the highest similarity value is described as the representative C=C isomer candidate for the EAD-MS/MS spectrum.

14) TG[M+NH4]+ / Lipid quantification

Quantitative	No	Batch correction	No
Normalization to reference	No	Further quantification remarks	-

15) Ceramide non-hydroxyfatty acid-sphingosine (Cer_NS)[M+H]+ / Lipid identification

Lipid class	Ceramide non-hydroxyfatty acid-sphingosine (Cer_NS)	Did you presume assumptions for identification?	No
Derivatization	-	Check isomer overlap	No
MS Level for identification	MS1, MS2	RT verified by standard	No
Identification level	Double bond position	Separation of isobaric/isomeric interferece confirmed	No
Polarity mode	Positive	Model for separation prediction	No
Type of positive (precursor)ion	[M+H]+	Additional dimension/techniques	EAD
Fragments for identification		How was/were the additional dimension(s) used?	To determine doublebond positions

Fragment name
Neutral loss of H2O
Neutral loss of 2H2O
Neutral loss of CH4O2
Fatty acyl amide +C2H3O+ fragment
Fatty acyl amide +C2H2+ fragment
Fatty acyl amide+ fragment
Sphingosine -CH4O2 fragment
Sphingosine -2H2O fragment
Sphingosine -H2O fragment

Isotope correction at MS1	No	Was a model used to predict lipid molecule separation?	No
Isotope correction at MS2	No	Lipid Identification Software	MS-DIAL
MS1 verified by standard	Yes	Data manipulation	Smoothing, Centroiding
MS2 verified by standard	Yes	Nomenclature for intact lipid molecule	Yes
Background check at MS1	Yes	Nomenclature for fragment ions	No
Background check at MS2	No	Further identification remarks	The reverse dot product similarity value, where the in silico spectrum is used for the library template, is used as the correlation coefficient value. The candidates are ranked by the reverse dot product score and the candidate with the highest similarity value is described as the representative C=C isomer candidate for the EAD-MS/MS spectrum.

15) Ceramide non-hydroxyfatty acid-sphingosine (Cer_NS)[M+H]+ / Lipid quantification

Quantitative	No	Batch correction	No
Normalization to reference	No	Further quantification remarks	-

16) Ceramide non-hydroxyfatty acid-dihydrosphingosine (Cer_NDS)[M+H]+ / Lipid identification

Lipid class	Ceramide non-hydroxyfatty acid-dihydrosphingosine (Cer_NDS)	Did you presume assumptions for identification?	No
Derivatization	-	Check isomer overlap	No
MS Level for identification	MS1, MS2	RT verified by standard	No
Identification level	Double bond position	Separation of isobaric/isomeric interferece confirmed	No
Polarity mode	Positive	Model for separation prediction	No
Type of positive (precursor)ion	[M+H]+	Additional dimension/techniques	EAD
Fragments for identification		How was/were the additional dimension(s) used?	To determine doublebond positions
Isotope correction at MS1	No	Was a model used to predict lipid molecule separation?	No
Isotope correction at MS2	No	Lipid Identification Software	MS-DIAL
MS1 verified by standard	No	Data manipulation	Smoothing, Centroiding
MS2 verified by standard	No	Nomenclature for intact lipid molecule	Yes
Background check at MS1	Yes	Nomenclature for fragment ions	No
Background check at MS2	No	Further identification remarks	The reverse dot product similarity value, where the in silico spectrum is used for the library template, is used as the correlation coefficient value. The candidates are ranked by the reverse dot product score and the candidate with the highest similarity value is described as the representative C=C isomer candidate for the EAD-MS/MS spectrum.

Fragment name
Neutral loss of H2O
Neutral loss of 2H2O
Neutral loss of CH4O2
Fatty acyl amide +C2H3O+ fragment
Fatty acyl amide +C2H2+ fragment
Fatty acyl amide+ fragment
Sphingosine -CH4O2 fragment
Sphingosine -2H2O fragment
Sphingosine -H2O fragment

16) Ceramide non-hydroxyfatty acid-dihydrosphingosine (Cer_NDS)[M+H]+ / Lipid quantification

Quantitative	No	Batch correction	No
Normalization to reference	No	Further quantification remarks	-

17) Hexosylceramide non-hydroxyfatty acid-sphingosine (HexCer_NS)[M+H]+ / Lipid identification

Lipid class	Hexosylceramide non-hydroxyfatty acid-sphingosine (HexCer_NS)	Did you presume assumptions for identification?	No
Derivatization	-	Check isomer overlap	No
MS Level for identification	MS1, MS2	RT verified by standard	No
Identification level	Double bond position	Separation of isobaric/isomeric interferece confirmed	No
Polarity mode	Positive	Model for separation prediction	No
Type of positive (precursor)ion	[M+H]+	Additional dimension/techniques	EAD
Fragments for identification		How was/were the additional dimension(s) used?	To determine doublebond positions

Fragment name
Neutral loss of H2O
Neutral loss of hexose
Neutral loss of hexose and H2O
Neutral loss of hexose and 2H2O
Fatty acyl amide +C2H5O + hexose fragment
Fatty acyl amide +C2H3O+ fragment
Fatty acyl amide +C2H2+ fragment
Fatty acyl amide+ fragment
Sphingosine -CH4O2 fragment
Sphingosine -2H2O fragment
Sphingosine -H2O fragment
Hexose + C2H5N+

Isotope correction at MS1	No	Was a model used to predict lipid molecule separation?	No
Isotope correction at MS2	No	Lipid Identification Software	MS-DIAL
MS1 verified by standard	Yes	Data manipulation	Smoothing, Centroiding
MS2 verified by standard	Yes	Nomenclature for intact lipid molecule	Yes
Background check at MS1	Yes	Nomenclature for fragment ions	No
Background check at MS2	No	Further identification remarks	The reverse dot product similarity value, where the in silico spectrum is used for the library template, is used as the correlation coefficient value. The candidates are ranked by the reverse dot product score and the candidate with the highest similarity value is described as the representative C=C isomer candidate for the EAD-MS/MS spectrum.

17) Hexosylceramide non-hydroxyfatty acid-sphingosine (HexCer_NS)[M+H]+ / Lipid quantification

Quantitative	No	Batch correction	No
Normalization to reference	No	Further quantification remarks	-

18) SHexCer[M+H]+ / Lipid identification

Lipid class	SHexCer	Did you presume assumptions for identification?	No
Derivatization	-	Check isomer overlap	No
MS Level for identification	MS1, MS2	RT verified by standard	No
Identification level	Molecular species level	Separation of isobaric/isomeric interferece confirmed	No
Polarity mode	Positive	Model for separation prediction	No
Type of positive (precursor)ion	[M+H]+	Additional dimension/techniques	EAD
Fragments for identification		How was/were the additional dimension(s) used?	To determine OH positions

Fragment name
Neutral loss of SO3
-HG(SHex,98)
-HG(SHex,260)
-HG(SHex,278)
-HG(SHex,242)
Fatty acyl amide +C2H2+ fragment
Fatty acyl amide+ fragment
Sphingosine -2H2O fragment
Sphingosine -H2O fragment
Sphingosine -CH4O2 fragment
Hexose + C2H5N+

Isotope correction at MS1	No	Was a model used to predict lipid molecule separation?	No
Isotope correction at MS2	No	Lipid Identification Software	MS-DIAL
MS1 verified by standard	Yes	Data manipulation	Smoothing, Centroiding
MS2 verified by standard	Yes	Nomenclature for intact lipid molecule	Yes
Background check at MS1	Yes	Nomenclature for fragment ions	No
Background check at MS2	No	Further identification remarks	The reverse dot product similarity value, where the in silico spectrum is used for the library template, is used as the correlation coefficient value. The candidates are ranked by the reverse dot product score and the candidate with the highest similarity value is described as the representative C=C isomer candidate for the EAD-MS/MS spectrum.

18) SHexCer[M+H]+ / Lipid quantification

Quantitative	No	Batch correction	No
Normalization to reference	No	Further quantification remarks	-

19) SM[M+H]+ / Lipid identification

Lipid class	SM	Did you presume assumptions for identification?	No
Derivatization	-	Check isomer overlap	No
MS Level for identification	MS1, MS2	RT verified by standard	No
Identification level	Double bond position	Separation of isobaric/isomeric interferece confirmed	No
Polarity mode	Positive	Model for separation prediction	No
Type of positive (precursor)ion	[M+H]+	Additional dimension/techniques	EAD
Fragments for identification		How was/were the additional dimension(s) used?	To determine doublebond positions

Fragment name
NL of Fatty acyl and NH4
Fatty acyl amide +C2H2 + Header fragment
Fatty acyl amide +C2H2+ fragment
Sphingosine -2H2O fragment
Header + C3H4NO
Header + C2H4N
HG(PC,184)

Isotope correction at MS1	No	Was a model used to predict lipid molecule separation?	No
Isotope correction at MS2	No	Lipid Identification Software	MS-DIAL
MS1 verified by standard	Yes	Data manipulation	Smoothing, Centroiding
MS2 verified by standard	Yes	Nomenclature for intact lipid molecule	Yes
Background check at MS1	Yes	Nomenclature for fragment ions	No
Background check at MS2	No	Further identification remarks	The reverse dot product similarity value, where the in silico spectrum is used for the library template, is used as the correlation coefficient value. The candidates are ranked by the reverse dot product score and the candidate with the highest similarity value is described as the representative C=C isomer candidate for the EAD-MS/MS spectrum.

19) SM[M+H]+ / Lipid quantification

Quantitative	No	Batch correction	No
Normalization to reference	No	Further quantification remarks	-

20) Hexosylceramide non-hydroxyfatty acid-dihydrosphingosine (HexCer_NDS)[M+H]+ / Lipid identification

Lipid class	Hexosylceramide non-hydroxyfatty acid-dihydrosphingosine (HexCer_NDS)	Did you presume assumptions for identification?	No
Derivatization	-	Check isomer overlap	No
MS Level for identification	MS1, MS2	RT verified by standard	No
Identification level	Double bond position	Separation of isobaric/isomeric interferece confirmed	No
Polarity mode	Positive	Model for separation prediction	No
Type of positive (precursor)ion	[M+H]+	Additional dimension/techniques	EAD
Fragments for identification	(see fragment table below)	How was/were the additional dimension(s) used?	To determine doublebond positions
Isotope correction at MS1	No	Was a model used to predict lipid molecule separation?	No
Isotope correction at MS2	No	Lipid Identification Software	MS-DIAL
MS1 verified by standard	Yes	Data manipulation	Smoothing, Centroiding
MS2 verified by standard	Yes	Nomenclature for intact lipid molecule	Yes
Background check at MS1	Yes	Nomenclature for fragment ions	No
Background check at MS2	No	Further identification remarks	The reverse dot product similarity value, where the in silico spectrum is used for the library template, is used as the correlation coefficient value. The candidates are ranked by the reverse dot product score and the candidate with the highest similarity value is described as the representative C=C isomer candidate for the EAD-MS/MS spectrum.

Fragment name
Neutral loss of H2O
Neutral loss of hexose
Neutral loss of hexose and H2O
Neutral loss of hexose and 2H2O
Fatty acyl amide +C2H5O + hexose fragment
Fatty acyl amide +C2H3O+ fragment
Fatty acyl amide +C2H2+ fragment
Fatty acyl amide+ fragment
Sphingosine -CH4O2 fragment
Sphingosine -2H2O fragment
Sphingosine -H2O fragment
Hexose + C2H5N+

20) Hexosylceramide non-hydroxyfatty acid-dihydrosphingosine (HexCer_NDS)[M+H]+ / Lipid quantification

Quantitative	No	Batch correction	No
Normalization to reference	No	Further quantification remarks	-

21) DG[M+NH4]+ / Lipid identification

Lipid class	DG	Background check at MS2	No
Derivatization	-	Did you presume assumptions for identification?	No
MS Level for identification	MS1, MS2	Check isomer overlap	No
Identification level	Molecular species level	RT verified by standard	No
Polarity mode	Positive	Separation of isobaric/isomeric interferece confirmed	No
Type of positive (precursor)ion	[M+NH4]+	Model for separation prediction	No
Fragments for identification		Additional dimension/techniques	-

Fragment name
Dehydro-monoacyl glycerols
Neutral loss of H2O

Isotope correction at MS1	No	Lipid Identification Software	MS-DIAL
Isotope correction at MS2	No	Data manipulation	Smoothing, Centroiding
MS1 verified by standard	Yes	Nomenclature for intact lipid molecule	Yes
MS2 verified by standard	Yes	Nomenclature for fragment ions	No
Background check at MS1	Yes	Further identification remarks	-

21) DG[M+NH4]+ / Lipid quantification

Quantitative	No	Batch correction	No
Normalization to reference	No	Further quantification remarks	-

22) Acylcarnitine (CAR)[M+H]+ / Lipid identification

Lipid class	Acylcarnitine (CAR)	Background check at MS2	No
Derivatization	-	Did you presume assumptions for identification?	No
MS Level for identification	MS1, MS2	Check isomer overlap	No
Identification level	Molecular species level	RT verified by standard	No
Polarity mode	Positive	Separation of isobaric/isomeric interferece confirmed	No
Type of positive (precursor)ion	[M+H]+	Model for separation prediction	No
Fragments for identification		Additional dimension/techniques	-

Fragment name
Characteristic fragment (C4H5O2+)

Isotope correction at MS1	No	Lipid Identification Software	MS-DIAL
Isotope correction at MS2	No	Data manipulation	Smoothing, Centroiding
MS1 verified by standard	No	Nomenclature for intact lipid molecule	Yes
MS2 verified by standard	No	Nomenclature for fragment ions	No
Background check at MS1	Yes	Further identification remarks	-

22) Acylcarnitine (CAR)[M+H]+ / Lipid quantification

Quantitative	No	Batch correction	No
Normalization to reference	No	Further quantification remarks	-

23) BMP[M+NH4]+ / Lipid identification

Lipid class	BMP	Background check at MS2	No
Derivatization	-	Did you presume assumptions for identification?	No
MS Level for identification	MS1, MS2	Check isomer overlap	No
Identification level	Molecular species level	RT verified by standard	No
Polarity mode	Positive	Separation of isobaric/isomeric interferece confirmed	No
Type of positive (precursor)ion	[M+NH4]+	Model for separation prediction	No
Fragments for identification		Additional dimension/techniques	-

Fragment name
Dehydro-monoacyl glycerols
Neutral loss of glycerophosphate

Isotope correction at MS1	No	Lipid Identification Software	MS-DIAL
Isotope correction at MS2	No	Data manipulation	Smoothing, Centroiding
MS1 verified by standard	No	Nomenclature for intact lipid molecule	Yes
MS2 verified by standard	No	Nomenclature for fragment ions	No
Background check at MS1	Yes	Further identification remarks	-

23) BMP[M+NH4]+ / Lipid quantification

Quantitative	No	Batch correction	No
Normalization to reference	No	Further quantification remarks	-

24) CE[M+NH4]+ / Lipid identification

Lipid class	CE	Background check at MS2	No
Derivatization	-	Did you presume assumptions for identification?	No
MS Level for identification	MS1, MS2	Check isomer overlap	No
Identification level	Molecular species level	RT verified by standard	No
Polarity mode	Positive	Separation of isobaric/isomeric interferece confirmed	No
Type of positive (precursor)ion	[M+NH4]+	Model for separation prediction	No
Fragments for identification		Additional dimension/techniques	-

Fragment name
Neutral loss of fatty acyl

Isotope correction at MS1	No	Lipid Identification Software	MS-DIAL
Isotope correction at MS2	No	Data manipulation	Smoothing, Centroiding
MS1 verified by standard	Yes	Nomenclature for intact lipid molecule	Yes
MS2 verified by standard	Yes	Nomenclature for fragment ions	No
Background check at MS1	Yes	Further identification remarks	-

24) CE[M+NH4]+ / Lipid quantification

Quantitative	No	Batch correction	No
Normalization to reference	No	Further quantification remarks	-

25) Coenzyme Q (CoQ)[M+H]+ / Lipid identification

Lipid class	Coenzyme Q (CoQ)	Background check at MS2	No
Derivatization	-	Did you presume assumptions for identification?	No
MS Level for identification	MS1, MS2	Check isomer overlap	No
Identification level	Molecular species level	RT verified by standard	No
Polarity mode	Positive	Separation of isobaric/isomeric interferece confirmed	No
Type of positive (precursor)ion	[M+H]+	Model for separation prediction	No
Fragments for identification		Additional dimension/techniques	-

Fragment name
Characteristic fragment (C10H13O4+)

Isotope correction at MS1	No	Lipid Identification Software	MS-DIAL
Isotope correction at MS2	No	Data manipulation	Smoothing, Centroiding
MS1 verified by standard	No	Nomenclature for intact lipid molecule	Yes
MS2 verified by standard	No	Nomenclature for fragment ions	No
Background check at MS1	Yes	Further identification remarks	-

25) Coenzyme Q (CoQ)[M+H]+ / Lipid quantification

Quantitative	No	Batch correction	No
Normalization to reference	No	Further quantification remarks	-

26) Hex2Cer[M+H]+ / Lipid identification

Lipid class	Hex2Cer	Background check at MS2	No
Derivatization	-	Did you presume assumptions for identification?	No
MS Level for identification	MS1, MS2	Check isomer overlap	No
Identification level	Molecular species level	RT verified by standard	No
Polarity mode	Positive	Separation of isobaric/isomeric interferece confirmed	No
Type of positive (precursor)ion	[M+H]+	Model for separation prediction	No
Fragments for identification		Additional dimension/techniques	-

Fragment name
Neutral loss of hexose
Neutral loss of 2hexose
Sphingosine -H2O fragment
Sphingosine -2H2O fragment
Sphingosine -CH4O2 fragment

Isotope correction at MS1	No	Lipid Identification Software	MS-DIAL
Isotope correction at MS2	No	Data manipulation	Smoothing, Centroiding
MS1 verified by standard	No	Nomenclature for intact lipid molecule	Yes
MS2 verified by standard	No	Nomenclature for fragment ions	No
Background check at MS1	Yes	Further identification remarks	-

26) Hex2Cer[M+H]+ / Lipid quantification

Quantitative	No	Batch correction	No
Normalization to reference	No	Further quantification remarks	-

27) LPE O[M+H]+ / Lipid identification

Lipid class	LPE O	Background check at MS2	No
Derivatization	-	Did you presume assumptions for identification?	No
MS Level for identification	MS1, MS2	Check isomer overlap	No
Identification level	Molecular species level	RT verified by standard	No
Polarity mode	Positive	Separation of isobaric/isomeric interferece confirmed	No
Type of positive (precursor)ion	[M+H]+	Model for separation prediction	No
Fragments for identification		Additional dimension/techniques	-

Fragment name
Neutral loss of C3H8NO4P
Neutral loss of C3H10NO5P

Isotope correction at MS1	No	Lipid Identification Software	MS-DIAL
Isotope correction at MS2	No	Data manipulation	Smoothing, Centroiding
MS1 verified by standard	No	Nomenclature for intact lipid molecule	Yes
MS2 verified by standard	No	Nomenclature for fragment ions	No
Background check at MS1	Yes	Further identification remarks	-

27) LPE O[M+H]+ / Lipid quantification

Quantitative	No	Batch correction	No
Normalization to reference	No	Further quantification remarks	-

28) Ether-linked triacylglycerol (EtherTG)[M+NH4]+ / Lipid identification

Lipid class	Ether-linked triacylglycerol (EtherTG)	Background check at MS2	No
Derivatization	-	Did you presume assumptions for identification?	No
MS Level for identification	MS1, MS2	Check isomer overlap	No
Identification level	Molecular species level	RT verified by standard	No
Polarity mode	Positive	Separation of isobaric/isomeric interferece confirmed	No
Type of positive (precursor)ion	[M+NH4]+	Model for separation prediction	No
Fragments for identification		Additional dimension/techniques	-

Fragment name
Neutral loss of fatty acyl and H2O
Neutral loss of alkyl ether

Isotope correction at MS1	No	Lipid Identification Software	MS-DIAL
Isotope correction at MS2	No	Data manipulation	Smoothing, Centroiding
MS1 verified by standard	No	Nomenclature for intact lipid molecule	Yes
MS2 verified by standard	No	Nomenclature for fragment ions	No
Background check at MS1	Yes	Further identification remarks	-

28) Ether-linked triacylglycerol (EtherTG)[M+NH4]+ / Lipid quantification

Quantitative	No	Batch correction	No
Normalization to reference	No	Further quantification remarks	-

29) Hexosylceramide hydroxyfatty acid-sphingosine (HexCer_HS)[M+H]+ / Lipid identification

Lipid class	Hexosylceramide hydroxyfatty acid-sphingosine (HexCer_HS)	Background check at MS2	No
Derivatization	-	Did you presume assumptions for identification?	No
MS Level for identification	MS1, MS2	Check isomer overlap	No
Identification level	Molecular species level	RT verified by standard	No
Polarity mode	Positive	Separation of isobaric/isomeric interferece confirmed	No
Type of positive (precursor)ion	[M+H]+	Model for separation prediction	No
Fragments for identification		Additional dimension/techniques	-

Fragment name
Neutral loss of hexose
Neutral loss of hexose and H2O
Sphingosine -H2O fragment
Sphingosine -2H2O fragment
Sphingosine -CH4O2 fragment

Isotope correction at MS1	No	Lipid Identification Software	MS-DIAL
Isotope correction at MS2	No	Data manipulation	Smoothing, Centroiding
MS1 verified by standard	No	Nomenclature for intact lipid molecule	Yes
MS2 verified by standard	No	Nomenclature for fragment ions	No
Background check at MS1	Yes	Further identification remarks	-

29) Hexosylceramide hydroxyfatty acid-sphingosine (HexCer_HS)[M+H]+ / Lipid quantification

Quantitative	No	Batch correction	No
Normalization to reference	No	Further quantification remarks	-

30) LPC[M+H]+ / Lipid identification

Lipid class	LPC	Background check at MS2	No
Derivatization	-	Did you presume assumptions for identification?	No
MS Level for identification	MS1, MS2	Check isomer overlap	No
Identification level	Molecular species level	RT verified by standard	No
Polarity mode	Positive	Separation of isobaric/isomeric interferece confirmed	No
Type of positive (precursor)ion	[M+H]+	Model for separation prediction	No
Fragments for identification		Additional dimension/techniques	-

Fragment name
Characteristic fragment (C5H15NO4P+)

Isotope correction at MS1	No	Lipid Identification Software	MS-DIAL
Isotope correction at MS2	No	Data manipulation	Smoothing, Centroiding
MS1 verified by standard	Yes	Nomenclature for intact lipid molecule	Yes
MS2 verified by standard	Yes	Nomenclature for fragment ions	No
Background check at MS1	Yes	Further identification remarks	-

30) LPC[M+H]+ / Lipid quantification

Quantitative	No	Batch correction	No
Normalization to reference	No	Further quantification remarks	-

31) LPE[M+H]+ / Lipid identification

Lipid class	LPE	Background check at MS2	No
Derivatization	-	Did you presume assumptions for identification?	No
MS Level for identification	MS1, MS2	Check isomer overlap	No
Identification level	Molecular species level	RT verified by standard	No
Polarity mode	Positive	Separation of isobaric/isomeric interferece confirmed	No
Type of positive (precursor)ion	[M+H]+	Model for separation prediction	No
Fragments for identification	Fragment name: Neutral loss of C2H8NO4P	Additional dimension/techniques	-
Isotope correction at MS1	No	Lipid Identification Software	MS-DIAL
Isotope correction at MS2	No	Data manipulation	Smoothing, Centroiding
MS1 verified by standard	Yes	Nomenclature for intact lipid molecule	Yes
MS2 verified by standard	Yes	Nomenclature for fragment ions	No
Background check at MS1	Yes	Further identification remarks	-

31) LPE[M+H]+ / Lipid quantification

Quantitative	No	Batch correction	No
Normalization to reference	No	Further quantification remarks	-

32) MG[M+NH4]+ / Lipid identification

Lipid class	MG	Background check at MS2	No
Derivatization	-	Did you presume assumptions for identification?	No
MS Level for identification	MS1, MS2	Check isomer overlap	No
Identification level	Molecular species level	RT verified by standard	No
Polarity mode	Positive	Separation of isobaric/isomeric interferece confirmed	No
Type of positive (precursor)ion	[M+NH4]+	Model for separation prediction	No
Fragments for identification	Fragment name: Neutral loss of H2O	Additional dimension/techniques	-
Isotope correction at MS1	No	Lipid Identification Software	MS-DIAL
Isotope correction at MS2	No	Data manipulation	Smoothing, Centroiding
MS1 verified by standard	Yes	Nomenclature for intact lipid molecule	Yes
MS2 verified by standard	Yes	Nomenclature for fragment ions	No
Background check at MS1	Yes	Further identification remarks	-

32) MG[M+NH4]+ / Lipid quantification

Quantitative	No	Batch correction	No
Normalization to reference	No	Further quantification remarks	-

33) MGDG[M+NH4]+ / Lipid identification

Lipid class	MGDG	Background check at MS2	No
Derivatization	-	Did you presume assumptions for identification?	No
MS Level for identification	MS1, MS2	Check isomer overlap	No
Identification level	Molecular species level	RT verified by standard	No
Polarity mode	Positive	Separation of isobaric/isomeric interferece confirmed	No
Type of positive (precursor)ion	[M+NH4]+	Model for separation prediction	No
Fragments for identification		Additional dimension/techniques	-

Fragment name
-HG(Hex,180)
-HG(Hex,180) -H2O and -SN1 acyl chain
-HG(Hex,180) -H2O and -SN2 acyl chain

Isotope correction at MS1	No	Lipid Identification Software	MS-DIAL
Isotope correction at MS2	No	Data manipulation	Smoothing, Centroiding
MS1 verified by standard	No	Nomenclature for intact lipid molecule	Yes
MS2 verified by standard	No	Nomenclature for fragment ions	No
Background check at MS1	Yes	Further identification remarks	-

33) MGDG[M+NH4]+ / Lipid quantification

Quantitative	No	Batch correction	No
Normalization to reference	No	Further quantification remarks	-

34) PC[M+H]+ / Lipid identification

Lipid class	PC	Background check at MS2	No
Derivatization	-	Did you presume assumptions for identification?	No
MS Level for identification	MS1, MS2	Check isomer overlap	No
Identification level	Molecular species level	RT verified by standard	No
Polarity mode	Positive	Separation of isobaric/isomeric interferece confirmed	No
Type of positive (precursor)ion	[M+H]+	Model for separation prediction	No
Fragments for identification		Additional dimension/techniques	-

Fragment name
HG(PC,184)
NL of SN1 fatty acyl
NL of SN2 fatty acyl
NL of SN1 fatty acyl and H2O
NL of SN2 fatty acyl and H2O

Isotope correction at MS1	No	Lipid Identification Software	MS-DIAL
Isotope correction at MS2	No	Data manipulation	Smoothing, Centroiding
MS1 verified by standard	Yes	Nomenclature for intact lipid molecule	Yes
MS2 verified by standard	Yes	Nomenclature for fragment ions	No
Background check at MS1	Yes	Further identification remarks	-

34) PC[M+H]+ / Lipid quantification

Quantitative	No	Batch correction	No
Normalization to reference	No	Further quantification remarks	-

35) PE[M+H]+ / Lipid identification

Lipid class	PE	Background check at MS2	No
Derivatization	-	Did you presume assumptions for identification?	No
MS Level for identification	MS1, MS2	Check isomer overlap	No
Identification level	Molecular species level	RT verified by standard	No
Polarity mode	Positive	Separation of isobaric/isomeric interferece confirmed	No
Type of positive (precursor)ion	[M+H]+	Model for separation prediction	No
Fragments for identification		Additional dimension/techniques	-

Fragment name
-HG(PE,141)
Fatty acyl fragment

Isotope correction at MS1	No	Lipid Identification Software	MS-DIAL
Isotope correction at MS2	No	Data manipulation	Smoothing, Centroiding
MS1 verified by standard	Yes	Nomenclature for intact lipid molecule	Yes
MS2 verified by standard	Yes	Nomenclature for fragment ions	No
Background check at MS1	Yes	Further identification remarks	-

35) PE[M+H]+ / Lipid quantification

Quantitative	No	Batch correction	No
Normalization to reference	No	Further quantification remarks	-

36) PG[M+NH4]+ / Lipid identification

Lipid class	PG	Background check at MS2	No
Derivatization	-	Did you presume assumptions for identification?	No
MS Level for identification	MS1, MS2	Check isomer overlap	No
Identification level	Molecular species level	RT verified by standard	No
Polarity mode	Positive	Separation of isobaric/isomeric interferece confirmed	No
Type of positive (precursor)ion	[M+NH4]+	Model for separation prediction	No
Fragments for identification		Additional dimension/techniques	-

Fragment name
-HG(PG,172)
Fatty acyl fragment

Isotope correction at MS1	No	Lipid Identification Software	MS-DIAL
Isotope correction at MS2	No	Data manipulation	Smoothing, Centroiding
MS1 verified by standard	Yes	Nomenclature for intact lipid molecule	Yes
MS2 verified by standard	Yes	Nomenclature for fragment ions	No
Background check at MS1	Yes	Further identification remarks	-

36) PG[M+NH4]+ / Lipid quantification

Quantitative	No	Batch correction	No
Normalization to reference	No	Further quantification remarks	-

37) PI[M+NH4]+ / Lipid identification

Lipid class	PI	Background check at MS2	No
Derivatization	-	Did you presume assumptions for identification?	No
MS Level for identification	MS1, MS2	Check isomer overlap	No
Identification level	Molecular species level	RT verified by standard	No
Polarity mode	Positive	Separation of isobaric/isomeric interferece confirmed	No
Type of positive (precursor)ion	[M+NH4]+	Model for separation prediction	No
Fragments for identification		Additional dimension/techniques	-

Fragment name
-HG(PI,260)
NL of SN1 acyl chain
NL of SN1 acyl chain and H2O
NL of SN2 acyl chain
NL of SN2 acyl chain and H2O

Isotope correction at MS1	No	Lipid Identification Software	MS-DIAL
Isotope correction at MS2	No	Data manipulation	Smoothing, Centroiding
MS1 verified by standard	Yes	Nomenclature for intact lipid molecule	Yes
MS2 verified by standard	Yes	Nomenclature for fragment ions	No
Background check at MS1	Yes	Further identification remarks	-

37) PI[M+NH4]+ / Lipid quantification

Quantitative	No	Batch correction	No
Normalization to reference	No	Further quantification remarks	-

38) PS[M+H]+ / Lipid identification

Lipid class	PS	Background check at MS2	No
Derivatization	-	Did you presume assumptions for identification?	No
MS Level for identification	MS1, MS2	Check isomer overlap	No
Identification level	Molecular species level	RT verified by standard	No
Polarity mode	Positive	Separation of isobaric/isomeric interferece confirmed	No
Type of positive (precursor)ion	[M+H]+	Model for separation prediction	No
Fragments for identification		Additional dimension/techniques	-

Fragment name
-HG(PS,185)
NL of fatty acyl chain
NL of fatty acyl chain and H2O

Isotope correction at MS1	No	Lipid Identification Software	MS-DIAL
Isotope correction at MS2	No	Data manipulation	Smoothing, Centroiding
MS1 verified by standard	Yes	Nomenclature for intact lipid molecule	Yes
MS2 verified by standard	Yes	Nomenclature for fragment ions	No
Background check at MS1	Yes	Further identification remarks	-

38) PS[M+H]+ / Lipid quantification

Quantitative	No	Batch correction	No
Normalization to reference	No	Further quantification remarks	-

39) SPB[M+H]+ / Lipid identification

Lipid class	SPB	Background check at MS2	No
Derivatization	-	Did you presume assumptions for identification?	No
MS Level for identification	MS1, MS2	Check isomer overlap	No
Identification level	Molecular species level	RT verified by standard	No
Polarity mode	Positive	Separation of isobaric/isomeric interferece confirmed	No
Type of positive (precursor)ion	[M+H]+	Model for separation prediction	No
Fragments for identification		Additional dimension/techniques	-

Fragment name
Neutral loss of H2O
Neutral loss of 2H2O
Neutral loss of CH4O2

Isotope correction at MS1	No	Lipid Identification Software	MS-DIAL
Isotope correction at MS2	No	Data manipulation	Smoothing, Centroiding
MS1 verified by standard	No	Nomenclature for intact lipid molecule	Yes
MS2 verified by standard	No	Nomenclature for fragment ions	No
Background check at MS1	Yes	Further identification remarks	-

39) SPB[M+H]+ / Lipid quantification

Quantitative	No	Batch correction	No
Normalization to reference	No	Further quantification remarks	-

40) TG[M+NH4]+ / Lipid identification

Lipid class	TG	Background check at MS2	No
Derivatization	-	Did you presume assumptions for identification?	No
MS Level for identification	MS1, MS2	Check isomer overlap	No
Identification level	Molecular species level	RT verified by standard	No
Polarity mode	Positive	Separation of isobaric/isomeric interferece confirmed	No
Type of positive (precursor)ion	[M+NH4]+	Model for separation prediction	No
Fragments for identification		Additional dimension/techniques	-
Isotope correction at MS1	No	Lipid Identification Software	MS-DIAL
Isotope correction at MS2	No	Data manipulation	Smoothing, Centroiding
MS1 verified by standard	Yes	Nomenclature for intact lipid molecule	Yes
MS2 verified by standard	Yes	Nomenclature for fragment ions	No
Background check at MS1	Yes	Further identification remarks	-

Fragment name
Neutral loss of acyl and H2O

40) TG[M+NH4]+ / Lipid quantification

Quantitative	No	Batch correction	No
Normalization to reference	No	Further quantification remarks	-

41) Triacylglycerol estolides (TG_EST)[M+NH4]+ / Lipid identification

Lipid class	Triacylglycerol estolides (TG_EST)	Background check at MS2	No
Derivatization	-	Did you presume assumptions for identification?	No
MS Level for identification	MS1, MS2	Check isomer overlap	No
Identification level	Molecular species level	RT verified by standard	No
Polarity mode	Positive	Separation of isobaric/isomeric interferece confirmed	No
Type of positive (precursor)ion	[M+NH4]+	Model for separation prediction	No
Fragments for identification		Additional dimension/techniques	-
Isotope correction at MS1	No	Lipid Identification Software	MS-DIAL
Isotope correction at MS2	No	Data manipulation	Smoothing, Centroiding
MS1 verified by standard	No	Nomenclature for intact lipid molecule	Yes
MS2 verified by standard	No	Nomenclature for fragment ions	No
Background check at MS1	Yes	Further identification remarks	-

Fragment name
Neutral loss of acyl and H2O
Neutral loss of oxidized acyl and H2O
Neutral loss of FAHFA

41) Triacylglycerol estolides (TG_EST)[M+NH4]+ / Lipid quantification

Quantitative	No	Batch correction	No
Normalization to reference	No	Further quantification remarks	-

42) PE P[M+H]+ / Lipid identification

Lipid class	PE P	Background check at MS2	No
Derivatization	-	Did you presume assumptions for identification?	No
MS Level for identification	MS1, MS2	Check isomer overlap	No
Identification level	Molecular species level	RT verified by standard	No
Polarity mode	Positive	Separation of isobaric/isomeric interferece confirmed	No
Type of positive (precursor)ion	[M+H]+	Model for separation prediction	No
Fragments for identification		Additional dimension/techniques	-

Fragment name
Neutral loss of C2H8NO4P
Alkyl ether +C2H8NO3P fragment
Dehydro-monoacyl glycerols

Isotope correction at MS1	No	Lipid Identification Software	MS-DIAL
Isotope correction at MS2	No	Data manipulation	Smoothing, Centroiding
MS1 verified by standard	No	Nomenclature for intact lipid molecule	Yes
MS2 verified by standard	No	Nomenclature for fragment ions	No
Background check at MS1	Yes	Further identification remarks	-

42) PE P[M+H]+ / Lipid quantification

Quantitative	No	Batch correction	No
Normalization to reference	No	Further quantification remarks	-

43) N-acyl ethanolamines (NAE)[M+H]+ / Lipid identification

Lipid class	N-acyl ethanolamines (NAE)	Background check at MS2	No
Derivatization	-	Did you presume assumptions for identification?	No
MS Level for identification	MS1, MS2	Check isomer overlap	No
Identification level	Molecular species level	RT verified by standard	No
Polarity mode	Positive	Separation of isobaric/isomeric interferece confirmed	No
Type of positive (precursor)ion	[M+H]+	Model for separation prediction	No
Fragments for identification	Fragment name: Neutral loss of 2H2O	Additional dimension/techniques	-
Isotope correction at MS1	No	Lipid Identification Software	MS-DIAL
Isotope correction at MS2	No	Data manipulation	Smoothing, Centroiding
MS1 verified by standard	No	Nomenclature for intact lipid molecule	Yes
MS2 verified by standard	No	Nomenclature for fragment ions	No
Background check at MS1	Yes	Further identification remarks	-

43) N-acyl ethanolamines (NAE)[M+H]+ / Lipid quantification

Quantitative	No	Batch correction	No
Normalization to reference	No	Further quantification remarks	-

44) ST[M+NH4]+ / Lipid identification

Lipid class	ST	Background check at MS2	No
Derivatization	-	Did you presume assumptions for identification?	No
MS Level for identification	MS1, MS2	Check isomer overlap	No
Identification level	Species level	RT verified by standard	No
Polarity mode	Positive	Separation of isobaric/isomeric interferece confirmed	No
Type of positive (precursor)ion	[M+NH4]+	Model for separation prediction	No
Fragments for identification	Fragment name: precursor m/z	Additional dimension/techniques	-
Isotope correction at MS1	No	Lipid Identification Software	MS-DIAL
Isotope correction at MS2	No	Data manipulation	Smoothing, Centroiding
MS1 verified by standard	No	Nomenclature for intact lipid molecule	Yes
MS2 verified by standard	No	Nomenclature for fragment ions	No
Background check at MS1	Yes	Further identification remarks	-

44) ST[M+NH4]+ / Lipid quantification

Quantitative	No	Batch correction	No
Normalization to reference	No	Further quantification remarks	-

45) PC O[M+H]+ / Lipid identification

Lipid class	PC O	Background check at MS2	No
Derivatization	-	Did you presume assumptions for identification?	No
MS Level for identification	MS1, MS2	Check isomer overlap	No
Identification level	Molecular species level	RT verified by standard	No
Polarity mode	Positive	Separation of isobaric/isomeric interferece confirmed	No
Type of positive (precursor)ion	[M+H]+	Model for separation prediction	No
Fragments for identification		Additional dimension/techniques	-

Fragment name
HG(PC,184)
NL of fatty acyl chain

Isotope correction at MS1	No	Lipid Identification Software	MS-DIAL
Isotope correction at MS2	No	Data manipulation	Smoothing, Centroiding
MS1 verified by standard	No	Nomenclature for intact lipid molecule	Yes
MS2 verified by standard	No	Nomenclature for fragment ions	No
Background check at MS1	Yes	Further identification remarks	-

45) PC O[M+H]+ / Lipid quantification

Quantitative	No	Batch correction	No
Normalization to reference	No	Further quantification remarks	-

46) SM[M+H]+ / Lipid identification

Lipid class	SM	Background check at MS2	No
Derivatization	-	Did you presume assumptions for identification?	No
MS Level for identification	MS1, MS2	Check isomer overlap	No
Identification level	Molecular species level	RT verified by standard	No
Polarity mode	Positive	Separation of isobaric/isomeric interferece confirmed	No
Type of positive (precursor)ion	[M+H]+	Model for separation prediction	No
Fragments for identification		Additional dimension/techniques	-

Fragment name
HG(PC,184)
Sphingosine fragment

Isotope correction at MS1	No	Lipid Identification Software	MS-DIAL
Isotope correction at MS2	No	Data manipulation	Smoothing, Centroiding
MS1 verified by standard	No	Nomenclature for intact lipid molecule	Yes
MS2 verified by standard	No	Nomenclature for fragment ions	No
Background check at MS1	Yes	Further identification remarks	-

46) SM[M+H]+ / Lipid quantification

Quantitative	No	Batch correction	No
Normalization to reference	No	Further quantification remarks	-

47) Ether-linked diacylglycerol (EtherDG)[M+NH4]+ / Lipid identification

Lipid class	Ether-linked diacylglycerol (EtherDG)	Background check at MS2	No
Derivatization	-	Did you presume assumptions for identification?	No
MS Level for identification	MS1, MS2	Check isomer overlap	No
Identification level	Molecular species level	RT verified by standard	No
Polarity mode	Positive	Separation of isobaric/isomeric interferece confirmed	No
Type of positive (precursor)ion	[M+NH4]+	Model for separation prediction	No
Fragments for identification	Fragment name: NL of fatty acyl chain	Additional dimension/techniques	-
Isotope correction at MS1	No	Lipid Identification Software	MS-DIAL
Isotope correction at MS2	No	Data manipulation	Smoothing, Centroiding
MS1 verified by standard	No	Nomenclature for intact lipid molecule	Yes
MS2 verified by standard	No	Nomenclature for fragment ions	No
Background check at MS1	Yes	Further identification remarks	-

47) Ether-linked diacylglycerol (EtherDG)[M+NH4]+ / Lipid quantification

Quantitative	No	Batch correction	No
Normalization to reference	No	Further quantification remarks	-

48) Ceramide hydroxy fatty acid-sphingosine (Cer_HS)[M+H]+ / Lipid identification

Lipid class	Ceramide hydroxy fatty acid-sphingosine (Cer_HS)	Background check at MS2	No
Derivatization	-	Did you presume assumptions for identification?	No
MS Level for identification	MS1, MS2	Check isomer overlap	No
Identification level	Molecular species level	RT verified by standard	No
Polarity mode	Positive	Separation of isobaric/isomeric interferece confirmed	No
Type of positive (precursor)ion	[M+H]+	Model for separation prediction	No
Fragments for identification		Additional dimension/techniques	-

Fragment name
NL of H2O
NL of hydroxy fatty acyl chain
NL of hydroxy fatty acyl chain and H2O
NL of hydroxy fatty acyl chain and CH2O

Isotope correction at MS1	No	Lipid Identification Software	MS-DIAL
Isotope correction at MS2	No	Data manipulation	-
MS1 verified by standard	No	Nomenclature for intact lipid molecule	Yes
MS2 verified by standard	No	Nomenclature for fragment ions	No
Background check at MS1	Yes	Further identification remarks	-

48) Ceramide hydroxy fatty acid-sphingosine (Cer_HS)[M+H]+ / Lipid quantification

Quantitative	No	Batch correction	No
Normalization to reference	No	Further quantification remarks	-

Note 3. Lipidomics minimal reporting checklist for HeLa lipid profiling for HeLa cells with the supplementation of VLC-PUFA (FA 32:6).

Separation Workflow

Created by https://lipidomicstandards.org, version v2.0.0

Overall study design

Title of the study	HeLa lipid profiling for HeLa cells with the supplementation of VLC-PUFA (FA 32:6)		
Document creation date	02/07/2024	Corresponding Email	htsugawa@go.tuat.ac.jp
Principle investigator	Hiroshi Tsugawa	Is the workflow targeted or untargeted?	Untargeted
Institution	Tokyo University of Agriculture and Technology	Clinical	No

Lipid extraction

Extraction method	2-phase system	2-phase system	Bligh&Dyer
pH adjustment	None	Were internal standards added prior extraction?	No

Analytical platform

Number of separation dimensions	One dimension	Ion source	ESI
Separation Type 1	LC	MS Level	MS1, MS2
Separation Mode 1	RP	Mass resolution for detected ion at MS1	High resolution
Separation window (1) for lipid analyte selection (±) in minutes	1.5	Resolution at m/z 200 at MS1	25071
RT verified by standard	Yes	Mass accuracy in ppm at MS1	0.00549
CCS verified by standard	No	Mass window for precursor ion isolation (in Da total isolation window)	1
Separation of isobaric/isomeric interferece confirmed	No	Mass resolution for detected ion at MS2	High resolution
Model for separation prediction	No	Resolution at m/z 200 at MS2	27858
MS type	QTOF	Mass accuracy in ppm at MS2	0.09463
MS vendor	SCIEX	Was/Were additional dimension/techniques used	No

Quality control

Blanks	Yes	Quality control	No
Type of Blanks	Extraction blank, Injection blank		

Method qualification and validation

Method validation	Yes	Precison	Yes
Lipid recovery	Yes	Accuracy	Yes
Dynamic quantification range	Yes	Guidelines followed	None
Limit of quantitation (LOQ)/Limit of detection (LOD)	Yes		

Reporting

Are reported raw data uploaded into repository?	Yes	Raw data upload	Yes
Are metadata available?	Yes	Additional comments	The raw data is available under the index of DM0054 at http://prime.psc.riken.jp/menta.cgi/prime/drop dex. The resolution and accuracy (ppm) for MS1 are those of m/z 132.9049 which were determined by the mass calibration in SCIEX OS. In addition, the resolution and accuracy for MS2 are those of m/z 185.1284, which were also determined by the mass calibration in SCIEX OS.
Summary data	Quantification and identification data		

Sample Descriptions

HeLa cells / Human / Cells

Provided information	Time to freeze (min), Storage time (month), Freeze-thaw cycles	Storage time (month)	1
Temperature handling original sample	4-8 °C	Freeze-thaw cycles	0
Instant sample preparation	Yes	Additives	None
Time to freeze (min)	10	Were samples stored under inert gas?	No
Snap freezing in liquid N2	No	Additional preservation methods	No
Storage temperature	-80 °C	Biobank samples	No

Lipid Class Descriptions

1) FA[M-H]- / Lipid identification

Lipid class	FA	Check isomer overlap	No
Derivatization	-	RT verified by standard	Yes
MS Level for identification	MS1	Separation of isobaric/isomeric interferece confirmed	Yes
Identification level	Species level	Model for separation prediction	Yes
Polarity mode	Negative	Additional dimension/techniques	-
Type of negative (precursor)ion	[M-H]-	Lipid Identification Software	MS-DIAL
Isotope correction at MS1	No	Data manipulation	Smoothing, Centroiding
MS1 verified by standard	Yes	Nomenclature for intact lipid molecule	Yes
Background check at MS1	Yes	Further identification remarks	-
Did you presume assumptions for identification?	No		

1) FA[M-H]- / Lipid quantification

Quantitative	Yes	Limit of quantification	No
MS Level for quantification	MS1	Normalization to reference	No
Internal lipid standard(s) MS1		Lipid Quantification Software	MS-DIAL

Internal standard	Endogenous subclass
FA 18:0(d3)	FA subclass

Type of quantification	Internal standard amount	Batch correction	No
Response correction	No	Further quantification remarks	-
Type I isotope correction	No		

2) DG[M+NH4]+ / Lipid identification

Lipid class	DG	Background check at MS2	No
Derivatization	-	Did you presume assumptions for identification?	No
MS Level for identification	MS1, MS2	Check isomer overlap	No
Identification level	Molecular species level	RT verified by standard	Yes
Polarity mode	Positive	Separation of isobaric/isomeric interferece confirmed	Yes
Type of positive (precursor)ion	[M+NH4]+	Model for separation prediction	Yes
Fragments for identification		Additional dimension/techniques	-

Fragment name
Dehydro-monoacyl glycerols
Neutral loss of H2O

Isotope correction at MS1	No	Lipid Identification Software	MS-DIAL
Isotope correction at MS2	No	Data manipulation	Smoothing, Centroiding
MS1 verified by standard	Yes	Nomenclature for intact lipid molecule	Yes
MS2 verified by standard	Yes	Nomenclature for fragment ions	No
Background check at MS1	Yes	Further identification remarks	-

2) DG[M+NH4]+ / Lipid quantification

Quantitative	Yes	Limit of quantification	No
MS Level for quantification	MS1	Normalization to reference	No
Internal lipid standard(s) MS1		Lipid Quantification Software	MS-DIAL

Internal standard	Endogenous subclass
DG 15:0_18:1(d7)	DG subclass

Type of quantification	Internal standard amount	Batch correction	No
Response correction	No	Further quantification remarks	-
Type I isotope correction	No		

3) DGDG[M+CH3COO]- / Lipid identification

Lipid class	DGDG	Background check at MS2	No
Derivatization	-	Did you presume assumptions for identification?	No
MS Level for identification	MS1, MS2	Check isomer overlap	No
Identification level	Molecular species level	RT verified by standard	Yes
Polarity mode	Negative	Separation of isobaric/isomeric interferece confirmed	Yes
Type of negative (precursor)ion	[M+CH3COO]-	Model for separation prediction	Yes
Fragments for identification		Additional dimension/techniques	-

Fragment name
Fatty acid fragment
Neutral loss of fatty acids

Isotope correction at MS1	No	Lipid Identification Software	MS-DIAL
Isotope correction at MS2	No	Data manipulation	Smoothing, Centroiding
MS1 verified by standard	No	Nomenclature for intact lipid molecule	Yes
MS2 verified by standard	No	Nomenclature for fragment ions	No
Background check at MS1	Yes	Further identification remarks	-

3) DGDG[M+CH3COO]- / Lipid quantification

Quantitative	Yes	Limit of quantification	No
MS Level for quantification	MS1	Normalization to reference	No
Internal lipid standard(s) MS1		Lipid Quantification Software	MS-DIAL

Internal standard	Endogenous subclass
LPC 18:1(d7)	DGDG subclass

Type of quantification	Internal standard amount	Batch correction	No
Response correction	No	Further quantification remarks	-
Type I isotope correction	No		

4) Sulfonolipid (SL)[M-H]- / Lipid identification

Lipid class	Sulfonolipid (SL)	Background check at MS2	No
Derivatization	-	Did you presume assumptions for identification?	No
MS Level for identification	MS1, MS2	Check isomer overlap	No
Identification level	Molecular species level	RT verified by standard	Yes
Polarity mode	Negative	Separation of isobaric/isomeric interferece confirmed	Yes
Type of negative (precursor)ion	[M-H]-	Model for separation prediction	Yes
Fragments for identification		Additional dimension/techniques	-

Fragment name
Sulfite
Neutral loss of N-acyl chain

Isotope correction at MS1	No	Lipid Identification Software	MS-DIAL
Isotope correction at MS2	No	Data manipulation	Smoothing, Centroiding
MS1 verified by standard	No	Nomenclature for intact lipid molecule	Yes
MS2 verified by standard	No	Nomenclature for fragment ions	No
Background check at MS1	Yes	Further identification remarks	-

4) Sulfonolipid (SL)[M-H]- / Lipid quantification

Quantitative	Yes	Limit of quantification	No
MS Level for quantification	MS1	Normalization to reference	No
Internal lipid standard(s) MS1		Lipid Quantification Software	MS-DIAL

Internal standard	Endogenous subclass
Cer 18:1;2O/15:0(d7)	SL subclass

Type of quantification	Internal standard amount	Batch correction	No
Response correction	No	Further quantification remarks	-
Type I isotope correction	No		

5) Acyl diacylglyceryl glucuronide(ADGGA)[M-H]- / Lipid identification

Lipid class	Acyl diacylglyceryl glucuronide(ADGGA)	Background check at MS2	No
Derivatization	-	Did you presume assumptions for identification?	No
MS Level for identification	MS1, MS2	Check isomer overlap	No
Identification level	Molecular species level	RT verified by standard	Yes
Polarity mode	Negative	Separation of isobaric/isomeric interferece confirmed	Yes
Type of negative (precursor)ion	[M-H]-	Model for separation prediction	Yes
Fragments for identification		Additional dimension/techniques	-

Fragment name
Fatty acid fragment

Isotope correction at MS1	No	Lipid Identification Software	MS-DIAL
Isotope correction at MS2	No	Data manipulation	Smoothing, Centroiding
MS1 verified by standard	No	Nomenclature for intact lipid molecule	Yes
MS2 verified by standard	No	Nomenclature for fragment ions	No
Background check at MS1	Yes	Further identification remarks	-

5) Acyl diacylglyceryl glucuronide(ADGGA)[M-H]- / Lipid quantification

Quantitative	Yes	Limit of quantification	No
MS Level for quantification	MS1	Normalization to reference	No
Internal lipid standard(s) MS1		Lipid Quantification Software	MS-DIAL

Internal standard	Endogenous subclass
LPC 18:1(d7)	ADGGA subclass

Type of quantification	Internal standard amount	Batch correction	No
Response correction	No	Further quantification remarks	-
Type I isotope correction	No		

6) Acylcarnitine (CAR)[M+H]+ / Lipid identification

Lipid class	Acylcarnitine (CAR)	Background check at MS2	No
Derivatization	-	Did you presume assumptions for identification?	No
MS Level for identification	MS1, MS2	Check isomer overlap	No
Identification level	Molecular species level	RT verified by standard	Yes
Polarity mode	Positive	Separation of isobaric/isomeric interferece confirmed	Yes
Type of positive (precursor)ion	[M+H]+	Model for separation prediction	Yes
Fragments for identification		Additional dimension/techniques	-

Fragment name
Characteristic fragment (C4H5O2+)

Isotope correction at MS1	No	Lipid Identification Software	MS-DIAL
Isotope correction at MS2	No	Data manipulation	Smoothing, Centroiding
MS1 verified by standard	No	Nomenclature for intact lipid molecule	Yes
MS2 verified by standard	No	Nomenclature for fragment ions	No
Background check at MS1	Yes	Further identification remarks	-

6) Acylcarnitine (CAR)[M+H]+ / Lipid quantification

Quantitative	Yes	Limit of quantification	No
MS Level for quantification	MS1	Normalization to reference	No
Internal lipid standard(s) MS1		Lipid Quantification Software	MS-DIAL

Internal standard	Endogenous subclass
LPC 18:1(d7)	CAR subclass

Type of quantification	Internal standard amount	Batch correction	No
Response correction	No	Further quantification remarks	-
Type I isotope correction	No		

7) Acylhexosyl brassicasterol (AHexBRS)[M+CH3COO]- / Lipid identification

Lipid class	Acylhexosyl brassicasterol (AHexBRS)	Background check at MS2	No
Derivatization	-	Did you presume assumptions for identification?	No
MS Level for identification	MS1, MS2	Check isomer overlap	No
Identification level	Molecular species level	RT verified by standard	Yes
Polarity mode	Negative	Separation of isobaric/isomeric interferece confirmed	Yes
Type of negative (precursor)ion	[M+CH3COO]-	Model for separation prediction	Yes
Fragments for identification		Additional dimension/techniques	-

Fragment name
Fatty acid fragment

Isotope correction at MS1	No	Lipid Identification Software	MS-DIAL
Isotope correction at MS2	No	Data manipulation	Smoothing, Centroiding
MS1 verified by standard	No	Nomenclature for intact lipid molecule	Yes
MS2 verified by standard	No	Nomenclature for fragment ions	No
Background check at MS1	Yes	Further identification remarks	-

7) Acylhexosyl brassicasterol (AHexBRS)[M+CH3COO]- / Lipid quantification

Quantitative	Yes	Limit of quantification	No
MS Level for quantification	MS1	Normalization to reference	No
Internal lipid standard(s) MS1		Lipid Quantification Software	MS-DIAL

Internal standard	Endogenous subclass
CE 18:1(d7)	AHexBRS subclass

Type of quantification	Internal standard amount	Batch correction	No
Response correction	No	Further quantification remarks	-
Type I isotope correction	No		

8) Acylhexosyl campesterol (AHexCAS)[M+CH3COO]- / Lipid identification

Lipid class	Acylhexosyl campesterol (AHexCAS)	Background check at MS2	No
Derivatization	-	Did you presume assumptions for identification?	No
MS Level for identification	MS1, MS2	Check isomer overlap	No
Identification level	Molecular species level	RT verified by standard	Yes
Polarity mode	Negative	Separation of isobaric/isomeric interferece confirmed	Yes
Type of negative (precursor)ion	[M+CH3COO]-	Model for separation prediction	Yes
Fragments for identification		Additional dimension/techniques	-

Fragment name
Fatty acid fragment

Isotope correction at MS1	No	Lipid Identification Software	MS-DIAL
Isotope correction at MS2	No	Data manipulation	Smoothing, Centroiding
MS1 verified by standard	No	Nomenclature for intact lipid molecule	Yes
MS2 verified by standard	No	Nomenclature for fragment ions	No
Background check at MS1	Yes	Further identification remarks	-

8) Acylhexosyl campesterol (AHexCAS)[M+CH3COO]- / Lipid quantification

Quantitative	Yes	Limit of quantification	No
MS Level for quantification	MS1	Normalization to reference	No
Internal lipid standard(s) MS1		Lipid Quantification Software	MS-DIAL

Internal standard	Endogenous subclass
CE 18:1(d7)	AHexCAS subclass

Type of quantification	Internal standard amount	Batch correction	No
Response correction	No	Further quantification remarks	-
Type I isotope correction	No		

9) Acylhexosyl cholesterol (AHexCS)[M+CH3COO]- / Lipid identification

Lipid class	Acylhexosyl cholesterol (AHexCS)	Background check at MS2	No
Derivatization	-	Did you presume assumptions for identification?	No
MS Level for identification	MS1, MS2	Check isomer overlap	No
Identification level	Molecular species level	RT verified by standard	Yes
Polarity mode	Negative	Separation of isobaric/isomeric interferece confirmed	Yes
Type of negative (precursor)ion	[M+CH3COO]-	Model for separation prediction	Yes
Fragments for identification		Additional dimension/techniques	-

Fragment name
Fatty acid fragment

Isotope correction at MS1	No	Lipid Identification Software	MS-DIAL
Isotope correction at MS2	No	Data manipulation	Smoothing, Centroiding
MS1 verified by standard	No	Nomenclature for intact lipid molecule	Yes
MS2 verified by standard	No	Nomenclature for fragment ions	No
Background check at MS1	Yes	Further identification remarks	-

9) Acylhexosyl cholesterol (AHexCS)[M+CH3COO]- / Lipid quantification

Quantitative	Yes	Limit of quantification	No
MS Level for quantification	MS1	Normalization to reference	No
Internal lipid standard(s) MS1		Lipid Quantification Software	MS-DIAL

Internal standard	Endogenous subclass
CE 18:1(d7)	AHexCS subclass

Type of quantification	Internal standard amount	Batch correction	No
Response correction	No	Further quantification remarks	-
Type I isotope correction	No		

10) Acylhexosyl sitosterol (AHexSIS)[M+CH3COO]- / Lipid identification

Lipid class	Acylhexosyl sitosterol (AHexSIS)	Background check at MS2	No
Derivatization	-	Did you presume assumptions for identification?	No
MS Level for identification	MS1, MS2	Check isomer overlap	No
Identification level	Molecular species level	RT verified by standard	Yes
Polarity mode	Negative	Separation of isobaric/isomeric interferece confirmed	Yes
Type of negative (precursor)ion	[M+CH3COO]-	Model for separation prediction	Yes
Fragments for identification		Additional dimension/techniques	-

Fragment name
Fatty acid fragment

Isotope correction at MS1	No	Lipid Identification Software	MS-DIAL
Isotope correction at MS2	No	Data manipulation	Smoothing, Centroiding
MS1 verified by standard	No	Nomenclature for intact lipid molecule	Yes
MS2 verified by standard	No	Nomenclature for fragment ions	No
Background check at MS1	Yes	Further identification remarks	-

10) Acylhexosyl sitosterol (AHexSIS)[M+CH3COO]- / Lipid quantification

Quantitative	Yes	Limit of quantification	No
MS Level for quantification	MS1	Normalization to reference	No
Internal lipid standard(s) MS1		Lipid Quantification Software	MS-DIAL

Internal standard	Endogenous subclass
CE 18:1(d7)	AHexSIS subclass

Type of quantification	Internal standard amount	Batch correction	No
Response correction	No	Further quantification remarks	-
Type I isotope correction	No		

11) Acylhexosyl stigmasterol (AHexSTS)[M+CH3COO]- / Lipid identification

Lipid class	Acylhexosyl stigmasterol (AHexSTS)	Background check at MS2	No
Derivatization	-	Did you presume assumptions for identification?	No
MS Level for identification	MS1, MS2	Check isomer overlap	No
Identification level	Molecular species level	RT verified by standard	Yes
Polarity mode	Negative	Separation of isobaric/isomeric interferece confirmed	Yes
Type of negative (precursor)ion	[M+CH3COO]-	Model for separation prediction	Yes
Fragments for identification		Additional dimension/techniques	-

Fragment name
Fatty acid fragment

Isotope correction at MS1	No	Lipid Identification Software	MS-DIAL
Isotope correction at MS2	No	Data manipulation	Smoothing, Centroiding
MS1 verified by standard	No	Nomenclature for intact lipid molecule	Yes
MS2 verified by standard	No	Nomenclature for fragment ions	No
Background check at MS1	Yes	Further identification remarks	-

11) Acylhexosyl stigmasterol (AHexSTS)[M+CH3COO]- / Lipid quantification

Quantitative	Yes	Limit of quantification	No
MS Level for quantification	MS1	Normalization to reference	No
Internal lipid standard(s) MS1		Lipid Quantification Software	MS-DIAL

Internal standard	Endogenous subclass
CE 18:1(d7)	AHexSTS subclass

Type of quantification	Internal standard amount	Batch correction	No
Response correction	No	Further quantification remarks	-
Type I isotope correction	No		

12) Acylhexosylceramide (AHexCer)[M+CH3COO]- / Lipid identification

Lipid class	Acylhexosylceramide (AHexCer)	Background check at MS2	No
Derivatization	-	Did you presume assumptions for identification?	No
MS Level for identification	MS1, MS2	Check isomer overlap	No
Identification level	Molecular species level	RT verified by standard	Yes
Polarity mode	Negative	Separation of isobaric/isomeric interferece confirmed	Yes
Type of negative (precursor)ion	[M+CH3COO]-	Model for separation prediction	Yes
Fragments for identification		Additional dimension/techniques	-

Fragment name
Neutral loss of fatty acid
Neutral loss of fatty acid and hexose
Fatty acid fragment
Neutral loss of N- acyl
Neutral loss of fatty acid and H2O
Neutral loss of N-acyl and H2O

Isotope correction at MS1	No	Lipid Identification Software	MS-DIAL
Isotope correction at MS2	No	Data manipulation	Smoothing, Centroiding
MS1 verified by standard	No	Nomenclature for intact lipid molecule	Yes
MS2 verified by standard	No	Nomenclature for fragment ions	No
Background check at MS1	Yes	Further identification remarks	-

12) Acylhexosylceramide (AHexCer)[M+CH3COO]- / Lipid quantification

Quantitative	Yes	Limit of quantification	No
MS Level for quantification	MS1	Normalization to reference	No
Internal lipid standard(s) MS1		Lipid Quantification Software	MS-DIAL

Internal standard	Endogenous subclass
Cer 18:1;2O/15:0(d7)	AHexCer subclass

Type of quantification	Internal standard amount	Batch correction	No
Response correction	No	Further quantification remarks	-
Type I isotope correction	No		

13) Acylsphingomyelin (ASM)[M+CH3COO]- / Lipid identification

Lipid class	Acylsphingomyelin (ASM)	Background check at MS2	No
Derivatization	-	Did you presume assumptions for identification?	No
MS Level for identification	MS1, MS2	Check isomer overlap	No
Identification level	Molecular species level	RT verified by standard	Yes
Polarity mode	Negative	Separation of isobaric/isomeric interferece confirmed	Yes
Type of negative (precursor)ion	[M+CH3COO]-	Model for separation prediction	Yes
Fragments for identification		Additional dimension/techniques	-

Fragment name
Neutral loss of methyl
Neutral loss of methyl and fatty acid and H2O
Fatty acid fragment
Acyl amide

Isotope correction at MS1	No	Lipid Identification Software	MS-DIAL
Isotope correction at MS2	No	Data manipulation	Smoothing, Centroiding
MS1 verified by standard	No	Nomenclature for intact lipid molecule	Yes
MS2 verified by standard	No	Nomenclature for fragment ions	No
Background check at MS1	Yes	Further identification remarks	-

13) Acylsphingomyelin (ASM)[M+CH3COO]- / Lipid quantification

Quantitative	Yes	Limit of quantification	No
MS Level for quantification	MS1	Normalization to reference	No
Internal lipid standard(s) MS1		Lipid Quantification Software	MS-DIAL

Internal standard	Endogenous subclass
SM 18:1;2O/18:1(d9)	ASM subclass

Type of quantification	Internal standard amount	Batch correction	No
Response correction	No	Further quantification remarks	-
Type I isotope correction	No		

14) BMP[M+NH4]+ / Lipid identification

Lipid class	BMP	Background check at MS2	No
Derivatization	-	Did you presume assumptions for identification?	No
MS Level for identification	MS1, MS2	Check isomer overlap	No
Identification level	Molecular species level	RT verified by standard	Yes
Polarity mode	Positive	Separation of isobaric/isomeric interferece confirmed	Yes
Type of positive (precursor)ion	[M+NH4]+	Model for separation prediction	Yes
Fragments for identification		Additional dimension/techniques	-

Fragment name
Dehydro-monoacyl glycerols
Neutral loss of glycerophosphate

Isotope correction at MS1	No	Lipid Identification Software	MS-DIAL
Isotope correction at MS2	No	Data manipulation	Smoothing, Centroiding
MS1 verified by standard	No	Nomenclature for intact lipid molecule	Yes
MS2 verified by standard	No	Nomenclature for fragment ions	No
Background check at MS1	Yes	Further identification remarks	-

14) BMP[M+NH4]+ / Lipid quantification

Quantitative	Yes	Limit of quantification	No
MS Level for quantification	MS1	Normalization to reference	No
Internal lipid standard(s) MS1		Lipid Quantification Software	MS-DIAL

Internal standard	Endogenous subclass
PG 15:0_18:1(d7)	BMP subclass

Type of quantification	Internal standard amount	Batch correction	No
Response correction	No	Further quantification remarks	-
Type I isotope correction	No		

15) Brassicasterol[M+NH4]+ / Lipid identification

Lipid class	Brassicasterol	Check isomer overlap	No
Derivatization	-	RT verified by standard	Yes
MS Level for identification	MS1	Separation of isobaric/isomeric interferece confirmed	Yes
Identification level	Species level	Model for separation prediction	Yes
Polarity mode	Positive	Additional dimension/techniques	-
Type of positive (precursor)ion	[M+NH4]+	Lipid Identification Software	MS-DIAL
Isotope correction at MS1	No	Data manipulation	Smoothing, Centroiding
MS1 verified by standard	No	Nomenclature for intact lipid molecule	Yes
Background check at MS1	Yes	Further identification remarks	-
Did you presume assumptions for identification?	No		

15) Brassicasterol[M+NH4]+ / Lipid quantification

Quantitative	Yes	Limit of quantification	No
MS Level for quantification	MS1	Normalization to reference	No
Internal lipid standard(s) MS1		Lipid Quantification Software	MS-DIAL

Internal standard	Endogenous subclass
CE 18:1(d7)	Brassicasterol

Type of quantification	Internal standard amount	Batch correction	No
Response correction	No	Further quantification remarks	-
Type I isotope correction	No		

16) Brassicasterol ester (BRSE)[M+NH4]+ / Lipid identification

Lipid class	Brassicasterol ester (BRSE)	Background check at MS2	No
Derivatization	-	Did you presume assumptions for identification?	No
MS Level for identification	MS1, MS2	Check isomer overlap	No
Identification level	Molecular species level	RT verified by standard	Yes
Polarity mode	Positive	Separation of isobaric/isomeric interferece confirmed	Yes
Type of positive (precursor)ion	[M+NH4]+	Model for separation prediction	Yes
Fragments for identification		Additional dimension/techniques	-

Fragment name
Neutral loss of fatty acid

Isotope correction at MS1	No	Lipid Identification Software	MS-DIAL
Isotope correction at MS2	No	Data manipulation	Smoothing, Centroiding
MS1 verified by standard	No	Nomenclature for intact lipid molecule	Yes
MS2 verified by standard	No	Nomenclature for fragment ions	No
Background check at MS1	Yes	Further identification remarks	-

16) Brassicasterol ester (BRSE)[M+NH4]+ / Lipid quantification

Quantitative	Yes	Limit of quantification	No
MS Level for quantification	MS1	Normalization to reference	No
Internal lipid standard(s) MS1		Lipid Quantification Software	MS-DIAL

Internal standard	Endogenous subclass
CE 18:1(d7)	BRSE subclass

Type of quantification	Internal standard amount	Batch correction	No
Response correction	No	Further quantification remarks	-
Type I isotope correction	No		

17) Campesterol ester (CASE)[M+NH4]+ / Lipid identification

Lipid class	Campesterol ester (CASE)	Background check at MS2	No
Derivatization	-	Did you presume assumptions for identification?	No
MS Level for identification	MS1, MS2	Check isomer overlap	No
Identification level	Molecular species level	RT verified by standard	Yes
Polarity mode	Positive	Separation of isobaric/isomeric interferece confirmed	Yes
Type of positive (precursor)ion	[M+NH4]+	Model for separation prediction	Yes
Fragments for identification		Additional dimension/techniques	-

Fragment name
Neutral loss of fatty acid

Isotope correction at MS1	No	Lipid Identification Software	MS-DIAL
Isotope correction at MS2	No	Data manipulation	Smoothing, Centroiding
MS1 verified by standard	No	Nomenclature for intact lipid molecule	Yes
MS2 verified by standard	No	Nomenclature for fragment ions	No
Background check at MS1	Yes	Further identification remarks	-

17) Campesterol ester (CASE)[M+NH4]+ / Lipid quantification

Quantitative	Yes	Limit of quantification	No
MS Level for quantification	MS1	Normalization to reference	No
Internal lipid standard(s) MS1		Lipid Quantification Software	MS-DIAL

Internal standard	Endogenous subclass
CE 18:1(d7)	CASE subclass

Type of quantification	Internal standard amount	Batch correction	No
Response correction	No	Further quantification remarks	-
Type I isotope correction	No		

18) CL[M-H]- / Lipid identification

Lipid class	CL	Background check at MS2	No
Derivatization	-	Did you presume assumptions for identification?	No
MS Level for identification	MS1, MS2	Check isomer overlap	No
Identification level	Molecular species level	RT verified by standard	Yes
Polarity mode	Negative	Separation of isobaric/isomeric interferece confirmed	Yes
Type of negative (precursor)ion	[M-H]-	Model for separation prediction	Yes
Fragments for identification		Additional dimension/techniques	-

Fragment name
Phosphoglycerol - H2O
Fatty acid fragment
Phosphatidic acid

Isotope correction at MS1	No	Lipid Identification Software	MS-DIAL
Isotope correction at MS2	No	Data manipulation	Smoothing, Centroiding
MS1 verified by standard	No	Nomenclature for intact lipid molecule	Yes
MS2 verified by standard	No	Nomenclature for fragment ions	No
Background check at MS1	Yes	Further identification remarks	-

18) CL[M-H]- / Lipid quantification

Quantitative	Yes	Limit of quantification	No
MS Level for quantification	MS1	Normalization to reference	No
Internal lipid standard(s) MS1		Lipid Quantification Software	MS-DIAL

Internal standard	Endogenous subclass
PG 15:0_18:1(d7)	CL subclass

Type of quantification	Internal standard amount	Batch correction	No
Response correction	No	Further quantification remarks	-
Type I isotope correction	No		

19) Ceramide alpha-hydroxy fatty acid-dihydrosphingosine (Cer_ADS)[M+CH3COO]- / Lipid identification

Lipid class	Ceramide alpha-hydroxy fatty acid-dihydrosphingosine (Cer_ADS)	Background check at MS2	No
Derivatization	-	Did you presume assumptions for identification?	No
MS Level for identification	MS1, MS2	Check isomer overlap	No
Identification level	Molecular species level	RT verified by standard	Yes
Polarity mode	Negative	Separation of isobaric/isomeric interferece confirmed	Yes
Type of negative (precursor)ion	[M+CH3COO]-	Model for separation prediction	Yes
Fragments for identification		Additional dimension/techniques	-

Fragment name
Neutral loss of CH4O2
Neutral loss of H2O
Sphinganine
Sphinganine -H2O fragment
Oxidized acyl -2H fragment
Oxidized acyl -CH2O fragment
Sphinganine -C2H7NO fragment

Isotope correction at MS1	No	Lipid Identification Software	MS-DIAL
Isotope correction at MS2	No	Data manipulation	Smoothing, Centroiding
MS1 verified by standard	No	Nomenclature for intact lipid molecule	Yes
MS2 verified by standard	No	Nomenclature for fragment ions	No
Background check at MS1	Yes	Further identification remarks	-

19) Ceramide alpha-hydroxy fatty acid-dihydrosphingosine (Cer_ADS)[M+CH3COO]- / Lipid quantification

Quantitative	Yes	Limit of quantification	No
MS Level for quantification	MS1	Normalization to reference	No
Internal lipid standard(s) MS1		Lipid Quantification Software	MS-DIAL

Internal standard	Endogenous subclass
Cer 18:1;2O/15:0(d7)	Cer_ADS subclass

Type of quantification	Internal standard amount	Batch correction	No
Response correction	No	Further quantification remarks	-
Type I isotope correction	No		

20) Ceramide alpha-hydroxy fatty acid-phytospingosine (Cer_AP)[M+CH3COO]- / Lipid identification

Lipid class	Ceramide alpha-hydroxy fatty acid-phytospingosine (Cer_AP)	Background check at MS2	No
Derivatization	-	Did you presume assumptions for identification?	No
MS Level for identification	MS1, MS2	Check isomer overlap	No
Identification level	Molecular species level	RT verified by standard	Yes
Polarity mode	Negative	Separation of isobaric/isomeric interferece confirmed	Yes
Type of negative (precursor)ion	[M+CH3COO]-	Model for separation prediction	Yes
Fragments for identification		Additional dimension/techniques	-

Fragment name
Oxidized fatty acyl -CH2O fragment
Oxidized fatty acyl +O fragment
Oxidized fatty acyl +C3H5NO fragment

Isotope correction at MS1	No	Lipid Identification Software	MS-DIAL
Isotope correction at MS2	No	Data manipulation	Smoothing, Centroiding
MS1 verified by standard	No	Nomenclature for intact lipid molecule	Yes
MS2 verified by standard	No	Nomenclature for fragment ions	No
Background check at MS1	Yes	Further identification remarks	-

20) Ceramide alpha-hydroxy fatty acid-phytospingosine (Cer_AP)[M+CH3COO]- / Lipid quantification

Quantitative	Yes	Limit of quantification	No
MS Level for quantification	MS1	Normalization to reference	No
Internal lipid standard(s) MS1		Lipid Quantification Software	MS-DIAL

Internal standard	Endogenous subclass
Cer 18:1;2O/15:0(d7)	Cer_AP subclass

Type of quantification	Internal standard amount	Batch correction	No
Response correction	No	Further quantification remarks	-
Type I isotope correction	No		

21) Ceramide alpha-hydroxy fatty acid-sphingosine (Cer_AS)[M+CH3COO]- / Lipid identification

Lipid class	Ceramide alpha-hydroxy fatty acid-sphingosine (Cer_AS)	Background check at MS2	No
Derivatization	-	Did you presume assumptions for identification?	No
MS Level for identification	MS1, MS2	Check isomer overlap	No
Identification level	Molecular species level	RT verified by standard	Yes
Polarity mode	Negative	Separation of isobaric/isomeric interferece confirmed	Yes
Type of negative (precursor)ion	[M+CH3COO]-	Model for separation prediction	Yes
Fragments for identification		Additional dimension/techniques	-

Fragment name
Neutral loss of CH4O2
Neutral loss of H2O
Sphingosine -H2O fragment
Sphingosine -C2H7NO fragment
Oxidized fatty acyl -CH2O fragment
Oxidized fatty acyl -2H fragment

Isotope correction at MS1	No	Lipid Identification Software	MS-DIAL
Isotope correction at MS2	No	Data manipulation	Smoothing, Centroiding
MS1 verified by standard	No	Nomenclature for intact lipid molecule	Yes
MS2 verified by standard	No	Nomenclature for fragment ions	No
Background check at MS1	Yes	Further identification remarks	-

21) Ceramide alpha-hydroxy fatty acid-sphingosine (Cer_AS)[M+CH3COO]- / Lipid quantification

Quantitative	Yes	Limit of quantification	No
MS Level for quantification	MS1	Normalization to reference	No
Internal lipid standard(s) MS1		Lipid Quantification Software	MS-DIAL

Internal standard	Endogenous subclass
Cer 18:1;2O/15:0(d7)	Cer_AS subclass

Type of quantification	Internal standard amount	Batch correction	No
Response correction	No	Further quantification remarks	-
Type I isotope correction	No		

22) Ceramide beta-hydroxy fatty acid-dihydrosphingosine (Cer_BDS)[M-H]- / Lipid identification

Lipid class	Ceramide beta-hydroxy fatty acid-dihydrosphingosine (Cer_BDS)	Background check at MS2	No
Derivatization	-	Did you presume assumptions for identification?	No
MS Level for identification	MS1, MS2	Check isomer overlap	No
Identification level	Molecular species level	RT verified by standard	Yes
Polarity mode	Negative	Separation of isobaric/isomeric interferece confirmed	Yes
Type of negative (precursor)ion	[M-H]-	Model for separation prediction	Yes
Fragments for identification		Additional dimension/techniques	-

Fragment name
Sphinganine
Sphinganine +C2H2O fragment
Sphinganine +C2H2O -CH4O fragment
Sphinganine -C2H7NO fragment

Isotope correction at MS1	No	Lipid Identification Software	MS-DIAL
Isotope correction at MS2	No	Data manipulation	Smoothing, Centroiding
MS1 verified by standard	No	Nomenclature for intact lipid molecule	Yes
MS2 verified by standard	No	Nomenclature for fragment ions	No
Background check at MS1	Yes	Further identification remarks	-

22) Ceramide beta-hydroxy fatty acid-dihydrosphingosine (Cer_BDS)[M-H]- / Lipid quantification

Quantitative	Yes	Limit of quantification	No
MS Level for quantification	MS1	Normalization to reference	No
Internal lipid standard(s) MS1		Lipid Quantification Software	MS-DIAL

Internal standard	Endogenous subclass
Cer 18:1;2O/15:0(d7)	Cer_BDS subclass

Type of quantification	Internal standard amount	Batch correction	No
Response correction	No	Further quantification remarks	-
Type I isotope correction	No		

23) Ceramide beta-hydroxy fatty acid-sphingosine (Cer_BS)[M-H]- / Lipid identification

Lipid class	Ceramide beta-hydroxy fatty acid-sphingosine (Cer_BS)	Background check at MS2	No
Derivatization	-	Did you presume assumptions for identification?	No
MS Level for identification	MS1, MS2	Check isomer overlap	No
Identification level	Molecular species level	RT verified by standard	Yes
Polarity mode	Negative	Separation of isobaric/isomeric interferece confirmed	Yes
Type of negative (precursor)ion	[M-H]-	Model for separation prediction	Yes
Fragments for identification		Additional dimension/techniques	-

Fragment name
Sphingosine -C2H7NO fragment
Sphingosine +C2H2O fragment
Sphinganine +C2H2O -CH2O fragment

Isotope correction at MS1	No	Lipid Identification Software	MS-DIAL
Isotope correction at MS2	No	Data manipulation	Smoothing, Centroiding
MS1 verified by standard	No	Nomenclature for intact lipid molecule	Yes
MS2 verified by standard	No	Nomenclature for fragment ions	No
Background check at MS1	Yes	Further identification remarks	-

23) Ceramide beta-hydroxy fatty acid-sphingosine (Cer_BS)[M-H]- / Lipid quantification

Quantitative	Yes	Limit of quantification	No
MS Level for quantification	MS1	Normalization to reference	No
Internal lipid standard(s) MS1		Lipid Quantification Software	MS-DIAL

Internal standard	Endogenous subclass
Cer 18:1;2O/15:0(d7)	Cer_BS subclass

Type of quantification	Internal standard amount	Batch correction	No
Response correction	No	Further quantification remarks	-
Type I isotope correction	No		

24) Ceramide Esterified beta-hydroxy fatty acid-dihydrosphingosine (Cer_EBDS)[M+CH3COO]- / Lipid identification

Lipid class	Ceramide Esterified beta-hydroxy fatty acid-dihydrosphingosine (Cer_EBDS)	Background check at MS2	No
Derivatization	-	Did you presume assumptions for identification?	No
MS Level for identification	MS1, MS2	Check isomer overlap	No
Identification level	Molecular species level	RT verified by standard	Yes
Polarity mode	Negative	Separation of isobaric/isomeric interferece confirmed	Yes
Type of negative (precursor)ion	[M+CH3COO]-	Model for separation prediction	Yes
Fragments for identification		Additional dimension/techniques	-

Fragment name
Sphingosine +C2H2O fragment
Fatty acid fragment
Neutral loss of fatty acyl and H2O

Isotope correction at MS1	No	Lipid Identification Software	MS-DIAL
Isotope correction at MS2	No	Data manipulation	Smoothing, Centroiding
MS1 verified by standard	No	Nomenclature for intact lipid molecule	Yes
MS2 verified by standard	No	Nomenclature for fragment ions	No
Background check at MS1	Yes	Further identification remarks	-

24) Ceramide Esterified beta-hydroxy fatty acid-dihydrosphingosine (Cer_EBDS)[M+CH3COO]- / Lipid quantification

Quantitative	Yes	Limit of quantification	No
MS Level for quantification	MS1	Normalization to reference	No
Internal lipid standard(s) MS1		Lipid Quantification Software	MS-DIAL

Internal standard	Endogenous subclass
Cer 18:1;2O/15:0(d7)	Cer_EBDS subclass

Type of quantification	Internal standard amount	Batch correction	No
Response correction	No	Further quantification remarks	-
Type I isotope correction	No		

25) Ceramide Esterified omega-hydroxy fatty acid-dihydrosphingosine (Cer_EODS)[M-H]- / Lipid identification

Lipid class	Ceramide Esterified omega-hydroxy fatty acid-dihydrosphingosine (Cer_EODS)	Background check at MS2	No
Derivatization	-	Did you presume assumptions for identification?	No
MS Level for identification	MS1, MS2	Check isomer overlap	No
Identification level	Molecular species level	RT verified by standard	Yes
Polarity mode	Negative	Separation of isobaric/isomeric interferece confirmed	Yes
Type of negative (precursor)ion	[M-H]-	Model for separation prediction	Yes
Fragments for identification		Additional dimension/techniques	-

Fragment name
Fatty acid fragment
Neutral loss of fatty acyl
Acyl amide

Isotope correction at MS1	No	Lipid Identification Software	MS-DIAL
Isotope correction at MS2	No	Data manipulation	Smoothing, Centroiding
MS1 verified by standard	No	Nomenclature for intact lipid molecule	Yes
MS2 verified by standard	No	Nomenclature for fragment ions	No
Background check at MS1	Yes	Further identification remarks	-

25) Ceramide Esterified omega-hydroxy fatty acid-dihydrosphingosine (Cer_EODS)[M-H]- / Lipid quantification

Quantitative	Yes	Limit of quantification	No
MS Level for quantification	MS1	Normalization to reference	No
Internal lipid standard(s) MS1		Lipid Quantification Software	MS-DIAL

Internal standard	Endogenous subclass
Cer 18:1;2O/15:0(d7)	Cer_EODS subclass

Type of quantification	Internal standard amount	Batch correction	No
Response correction	No	Further quantification remarks	-
Type I isotope correction	No		

26) Ceramide Esterified omega-hydroxy fatty acid-sphingosine (Cer_EOS)[M+CH3COO]- / Lipid identification

Lipid class	Ceramide Esterified omega-hydroxy fatty acid-sphingosine (Cer_EOS)	Background check at MS2	No
Derivatization	-	Did you presume assumptions for identification?	No
MS Level for identification	MS1, MS2	Check isomer overlap	No
Identification level	Molecular species level	RT verified by standard	Yes
Polarity mode	Negative	Separation of isobaric/isomeric interferece confirmed	Yes
Type of negative (precursor)ion	[M+CH3COO]-	Model for separation prediction	Yes
Fragments for identification		Additional dimension/techniques	-

Fragment name
Acyl amide
Fatty acid fragment
Neutral loss of fatty acyl

Isotope correction at MS1	No	Lipid Identification Software	MS-DIAL
Isotope correction at MS2	No	Data manipulation	Smoothing, Centroiding
MS1 verified by standard	No	Nomenclature for intact lipid molecule	Yes
MS2 verified by standard	No	Nomenclature for fragment ions	No
Background check at MS1	Yes	Further identification remarks	-

26) Ceramide Esterified omega-hydroxy fatty acid-sphingosine (Cer_EOS)[M+CH3COO]- / Lipid quantification

Quantitative	Yes	Limit of quantification	No
MS Level for quantification	MS1	Normalization to reference	No
Internal lipid standard(s) MS1		Lipid Quantification Software	MS-DIAL

Internal standard	Endogenous subclass
Cer 18:1;2O/15:0(d7)	Cer_EOS subclass

Type of quantification	Internal standard amount	Batch correction	No
Response correction	No	Further quantification remarks	-
Type I isotope correction	No		

27) Ceramide non-hydroxyfatty acid-dihydrosphingosine (Cer_NDS)[M+CH3COO]- / Lipid identification

Lipid class	Ceramide non-hydroxyfatty acid-dihydrosphingosine (Cer_NDS)	Background check at MS2	No
Derivatization	-	Did you presume assumptions for identification?	No
MS Level for identification	MS1, MS2	Check isomer overlap	No
Identification level	Molecular species level	RT verified by standard	Yes
Polarity mode	Negative	Separation of isobaric/isomeric interferece confirmed	Yes
Type of negative (precursor)ion	[M+CH3COO]-	Model for separation prediction	Yes
Fragments for identification		Additional dimension/techniques	-

Fragment name
Neutral loss of CH4O
Neutral loss of CH4O2
Sphinganine -C2H7NO fragment
Fatty acyl +C2H3N fragment
Fatty acyl -2H fragment

Isotope correction at MS1	No	Lipid Identification Software	MS-DIAL
Isotope correction at MS2	No	Data manipulation	Smoothing, Centroiding
MS1 verified by standard	No	Nomenclature for intact lipid molecule	Yes
MS2 verified by standard	No	Nomenclature for fragment ions	No
Background check at MS1	Yes	Further identification remarks	-

27) Ceramide non-hydroxyfatty acid-dihydrosphingosine (Cer_NDS)[M+CH3COO]- / Lipid quantification

Quantitative	Yes	Limit of quantification	No
MS Level for quantification	MS1	Normalization to reference	No
Internal lipid standard(s) MS1		Lipid Quantification Software	MS-DIAL

Internal standard	Endogenous subclass
Cer 18:1;2O/15:0(d7)	Cer_NDS subclass

Type of quantification	Internal standard amount	Batch correction	No
Response correction	No	Further quantification remarks	-
Type I isotope correction	No		

28) Ceramide non-hydroxyfatty acid-phytospingosine (Cer_NP)[M+CH3COO]- / Lipid identification

Lipid class	Ceramide non-hydroxyfatty acid-phytospingosine (Cer_NP)	Background check at MS2	No
Derivatization	-	Did you presume assumptions for identification?	No
MS Level for identification	MS1, MS2	Check isomer overlap	No
Identification level	Molecular species level	RT verified by standard	Yes
Polarity mode	Negative	Separation of isobaric/isomeric interferece confirmed	Yes
Type of negative (precursor)ion	[M+CH3COO]-	Model for separation prediction	Yes
Fragments for identification		Additional dimension/techniques	-

Fragment name
Neutral loss of H2O
Neutral loss of 2H2O
Sphinganine -CH7NO fragment
Fatty acyl +C3H5NO fragment
Acyl amide

Isotope correction at MS1	No	Lipid Identification Software	MS-DIAL
Isotope correction at MS2	No	Data manipulation	Smoothing, Centroiding
MS1 verified by standard	No	Nomenclature for intact lipid molecule	Yes
MS2 verified by standard	No	Nomenclature for fragment ions	No
Background check at MS1	Yes	Further identification remarks	-

28) Ceramide non-hydroxyfatty acid-phytospingosine (Cer_NP)[M+CH3COO]- / Lipid quantification

Quantitative	Yes	Limit of quantification	No
MS Level for quantification	MS1	Normalization to reference	No
Internal lipid standard(s) MS1		Lipid Quantification Software	MS-DIAL

Internal standard	Endogenous subclass
Cer 18:1;2O/15:0(d7)	Cer_NP subclass

Type of quantification	Internal standard amount	Batch correction	No
Response correction	No	Further quantification remarks	-
Type I isotope correction	No		

29) Ceramide non-hydroxyfatty acid-sphingosine (Cer_NS)[M+CH3COO]- / Lipid identification

Lipid class	Ceramide non-hydroxyfatty acid-sphingosine (Cer_NS)	Background check at MS2	No
Derivatization	-	Did you presume assumptions for identification?	No
MS Level for identification	MS1, MS2	Check isomer overlap	No
Identification level	Molecular species level	RT verified by standard	Yes
Polarity mode	Negative	Separation of isobaric/isomeric interferece confirmed	Yes
Type of negative (precursor)ion	[M+CH3COO]-	Model for separation prediction	Yes
Fragments for identification		Additional dimension/techniques	-

Fragment name
Neutral loss of H2O
Neutral loss of CH2O
Sphingosine -C2H7NO fragment
Fatty acyl +C2H3N fragment
Fatty acyl -2H fragment

Isotope correction at MS1	No	Lipid Identification Software	MS-DIAL
Isotope correction at MS2	No	Data manipulation	Smoothing, Centroiding
MS1 verified by standard	Yes	Nomenclature for intact lipid molecule	Yes
MS2 verified by standard	Yes	Nomenclature for fragment ions	No
Background check at MS1	Yes	Further identification remarks	-

29) Ceramide non-hydroxyfatty acid-sphingosine (Cer_NS)[M+CH3COO]- / Lipid quantification

Quantitative	Yes	Limit of quantification	No
MS Level for quantification	MS1	Normalization to reference	No
Internal lipid standard(s) MS1		Lipid Quantification Software	MS-DIAL

Internal standard	Endogenous subclass
Cer 18:1;2O/15:0(d7)	Cer_NS subclass

Type of quantification	Internal standard amount	Batch correction	No
Response correction	No	Further quantification remarks	-
Type I isotope correction	No		

30) Ceramide phosphoethanolamine (PE_Cer)[M-H]- / Lipid identification

Lipid class	Ceramide phosphoethanolamine (PE_Cer)	Background check at MS2	No
Derivatization	-	Did you presume assumptions for identification?	No
MS Level for identification	MS1, MS2	Check isomer overlap	No
Identification level	Molecular species level	RT verified by standard	Yes
Polarity mode	Negative	Separation of isobaric/isomeric interferece confirmed	Yes
Type of negative (precursor)ion	[M-H]-	Model for separation prediction	Yes
Fragments for identification		Additional dimension/techniques	-

Fragment name
Characteristic fragment (C2H7NO4P-)
Neutral loss of fatty acyl

Isotope correction at MS1	No	Lipid Identification Software	MS-DIAL
Isotope correction at MS2	No	Data manipulation	Smoothing, Centroiding
MS1 verified by standard	No	Nomenclature for intact lipid molecule	Yes
MS2 verified by standard	No	Nomenclature for fragment ions	No
Background check at MS1	Yes	Further identification remarks	-

30) Ceramide phosphoethanolamine (PE_Cer)[M-H]- / Lipid quantification

Quantitative	Yes	Limit of quantification	No
MS Level for quantification	MS1	Normalization to reference	No
Internal lipid standard(s) MS1		Lipid Quantification Software	MS-DIAL

Internal standard	Endogenous subclass
Cer 18:1;2O/15:0(d7)	PE_Cer subclass

Type of quantification	Internal standard amount	Batch correction	No
Response correction	No	Further quantification remarks	-
Type I isotope correction	No		

31) Ceramide phosphoinositol (PI_Cer)[M-H]- / Lipid identification

Lipid class	Ceramide phosphoinositol (PI_Cer)	Background check at MS2	No
Derivatization	-	Did you presume assumptions for identification?	No
MS Level for identification	MS1, MS2	Check isomer overlap	No
Identification level	Molecular species level	RT verified by standard	Yes
Polarity mode	Negative	Separation of isobaric/isomeric interferece confirmed	Yes
Type of negative (precursor)ion	[M-H]-	Model for separation prediction	Yes
Fragments for identification		Additional dimension/techniques	-

Fragment name
Phosphoinositol - H2O
Neutral loss of inositol
Neutral loss of fatty acyl

Isotope correction at MS1	No	Lipid Identification Software	MS-DIAL
Isotope correction at MS2	No	Data manipulation	Smoothing, Centroiding
MS1 verified by standard	No	Nomenclature for intact lipid molecule	Yes
MS2 verified by standard	No	Nomenclature for fragment ions	No
Background check at MS1	Yes	Further identification remarks	-

31) Ceramide phosphoinositol (PI_Cer)[M-H]- / Lipid quantification

Quantitative	Yes	Limit of quantification	No
MS Level for quantification	MS1	Normalization to reference	No
Internal lipid standard(s) MS1		Lipid Quantification Software	MS-DIAL

Internal standard	Endogenous subclass
Cer 18:1;2O/15:0(d7)	PI_Cer subclass

Type of quantification	Internal standard amount	Batch correction	No
Response correction	No	Further quantification remarks	-
Type I isotope correction	No		

32) FA[M+NH4]+ / Lipid identification

Lipid class	FA	Background check at MS2	No
Derivatization	-	Did you presume assumptions for identification?	No
MS Level for identification	MS1, MS2	Check isomer overlap	No
Identification level	Molecular species level	RT verified by standard	Yes
Polarity mode	Positive	Separation of isobaric/isomeric interferece confirmed	Yes
Type of positive (precursor)ion	[M+NH4]+	Model for separation prediction	Yes
Fragments for identification		Additional dimension/techniques	-

Fragment name
Neutral loss of fatty acyl

Isotope correction at MS1	No	Lipid Identification Software	MS-DIAL
Isotope correction at MS2	No	Data manipulation	Smoothing, Centroiding
MS1 verified by standard	Yes	Nomenclature for intact lipid molecule	Yes
MS2 verified by standard	Yes	Nomenclature for fragment ions	No
Background check at MS1	Yes	Further identification remarks	-

32) FA[M+NH4]+ / Lipid quantification

Quantitative	Yes	Limit of quantification	No
MS Level for quantification	MS1	Normalization to reference	No
Internal lipid standard(s) MS1		Lipid Quantification Software	MS-DIAL

Internal standard	Endogenous subclass
CE 18:1(d7)	CE subclass

Type of quantification	Internal standard amount	Batch correction	No
Response correction	No	Further quantification remarks	-
Type I isotope correction	No		

33) Cholic acid (BileAcid)[M-H]- / Lipid identification

Lipid class	Cholic acid (BileAcid)	Check isomer overlap	No
Derivatization	-	RT verified by standard	Yes
MS Level for identification	MS1	Separation of isobaric/isomeric interferece confirmed	Yes
Identification level	Species level	Model for separation prediction	Yes
Polarity mode	Negative	Additional dimension/techniques	-
Type of negative (precursor)ion	[M-H]-	Lipid Identification Software	MS-DIAL
Isotope correction at MS1	No	Data manipulation	Smoothing, Centroiding
MS1 verified by standard	No	Nomenclature for intact lipid molecule	Yes
Background check at MS1	Yes	Further identification remarks	-
Did you presume assumptions for identification?	No		

33) Cholic acid (BileAcid)[M-H]- / Lipid quantification

Quantitative	Yes	Limit of quantification	No
MS Level for quantification	MS1	Normalization to reference	No
Internal lipid standard(s) MS1		Lipid Quantification Software	MS-DIAL

Internal standard	Endogenous subclass
LPC 18:1(d7)	BileAcid subclass

Type of quantification	Internal standard amount	Batch correction	No
Response correction	No	Further quantification remarks	-
Type I isotope correction	No		

34) Cholic acid sulfate (BASulfate)[M-H]- / Lipid identification

Lipid class	Cholic acid sulfate (BASulfate)	Background check at MS2	No
Derivatization	-	Did you presume assumptions for identification?	No
MS Level for identification	MS1, MS2	Check isomer overlap	No
Identification level	Molecular species level	RT verified by standard	Yes
Polarity mode	Negative	Separation of isobaric/isomeric interferece confirmed	Yes
Type of negative (precursor)ion	[M-H]-	Model for separation prediction	Yes
Fragments for identification		Additional dimension/techniques	-

Fragment name
Hydrogensulfate

Isotope correction at MS1	No	Lipid Identification Software	MS-DIAL
Isotope correction at MS2	No	Data manipulation	Smoothing, Centroiding
MS1 verified by standard	No	Nomenclature for intact lipid molecule	Yes
MS2 verified by standard	No	Nomenclature for fragment ions	No
Background check at MS1	Yes	Further identification remarks	-

34) Cholic acid sulfate (BASulfate)[M-H]- / Lipid quantification

Quantitative	Yes	Limit of quantification	No
MS Level for quantification	MS1	Normalization to reference	No
Internal lipid standard(s) MS1		Lipid Quantification Software	MS-DIAL

Internal standard	Endogenous subclass
LPC 18:1(d7)	BASulfate subclass

Type of quantification	Internal standard amount	Batch correction	No
Response correction	No	Further quantification remarks	-
Type I isotope correction	No		

35) Coenzyme Q (CoQ)[M+H]+ / Lipid identification

Lipid class	Coenzyme Q (CoQ)	Background check at MS2	No
Derivatization	-	Did you presume assumptions for identification?	No
MS Level for identification	MS1, MS2	Check isomer overlap	No
Identification level	Molecular species level	RT verified by standard	Yes
Polarity mode	Positive	Separation of isobaric/isomeric interferece confirmed	Yes
Type of positive (precursor)ion	[M+H]+	Model for separation prediction	Yes
Fragments for identification		Additional dimension/techniques	-

Fragment name
Characteristic fragment (C10H13O4+)

Isotope correction at MS1	No	Lipid Identification Software	MS-DIAL
Isotope correction at MS2	No	Data manipulation	Smoothing, Centroiding
MS1 verified by standard	No	Nomenclature for intact lipid molecule	Yes
MS2 verified by standard	No	Nomenclature for fragment ions	No
Background check at MS1	Yes	Further identification remarks	-

35) Coenzyme Q (CoQ)[M+H]+ / Lipid quantification

Quantitative	Yes	Limit of quantification	No
MS Level for quantification	MS1	Normalization to reference	No
Internal lipid standard(s) MS1		Lipid Quantification Software	MS-DIAL

Internal standard	Endogenous subclass
LPC 18:1(d7)	CoQ subclass

Type of quantification	Internal standard amount	Batch correction	No
Response correction	No	Further quantification remarks	-
Type I isotope correction	No		

36) Dehydroergosterol ester (DEGSE)[M+NH4]+ / Lipid identification

Lipid class	Dehydroergosterol ester (DEGSE)	Background check at MS2	No
Derivatization	-	Did you presume assumptions for identification?	No
MS Level for identification	MS1, MS2	Check isomer overlap	No
Identification level	Molecular species level	RT verified by standard	Yes
Polarity mode	Positive	Separation of isobaric/isomeric interferece confirmed	Yes
Type of positive (precursor)ion	[M+NH4]+	Model for separation prediction	Yes
Fragments for identification		Additional dimension/techniques	-

Fragment name
Neutral loss of fatty acyl

Isotope correction at MS1	No	Lipid Identification Software	MS-DIAL
Isotope correction at MS2	No	Data manipulation	Smoothing, Centroiding
MS1 verified by standard	No	Nomenclature for intact lipid molecule	Yes
MS2 verified by standard	No	Nomenclature for fragment ions	No
Background check at MS1	Yes	Further identification remarks	-

36) Dehydroergosterol ester (DEGSE)[M+NH4]+ / Lipid quantification

Quantitative	Yes	Limit of quantification	No
MS Level for quantification	MS1	Normalization to reference	No
Internal lipid standard(s) MS1		Lipid Quantification Software	MS-DIAL

Internal standard	Endogenous subclass
CE 18:1(d7)	DEGSE subclass

Type of quantification	Internal standard amount	Batch correction	No
Response correction	No	Further quantification remarks	-
Type I isotope correction	No		

37) Desmosterol ester (DSMSE)[M+NH4]+ / Lipid identification

Lipid class	Desmosterol ester (DSMSE)	Background check at MS2	No
Derivatization	-	Did you presume assumptions for identification?	No
MS Level for identification	MS1, MS2	Check isomer overlap	No
Identification level	Molecular species level	RT verified by standard	Yes
Polarity mode	Positive	Separation of isobaric/isomeric interferece confirmed	Yes
Type of positive (precursor)ion	[M+NH4]+	Model for separation prediction	Yes
Fragments for identification		Additional dimension/techniques	-

Fragment name
Neutral loss of fatty acyl

Isotope correction at MS1	No	Lipid Identification Software	MS-DIAL
Isotope correction at MS2	No	Data manipulation	Smoothing, Centroiding
MS1 verified by standard	No	Nomenclature for intact lipid molecule	Yes
MS2 verified by standard	No	Nomenclature for fragment ions	No
Background check at MS1	Yes	Further identification remarks	-

37) Desmosterol ester (DSMSE)[M+NH4]+ / Lipid quantification

Quantitative	Yes	Limit of quantification	No
MS Level for quantification	MS1	Normalization to reference	No
Internal lipid standard(s) MS1		Lipid Quantification Software	MS-DIAL

Internal standard	Endogenous subclass
CE 18:1(d7)	DSMSE subclass

Type of quantification	Internal standard amount	Batch correction	No
Response correction	No	Further quantification remarks	-
Type I isotope correction	No		

38) Diacylglyceryl glucuronide (DGGA)[M-H]- / Lipid identification

Lipid class	Diacylglyceryl glucuronide (DGGA)	Background check at MS2	No
Derivatization	-	Did you presume assumptions for identification?	No
MS Level for identification	MS1, MS2	Check isomer overlap	No
Identification level	Molecular species level	RT verified by standard	Yes
Polarity mode	Negative	Separation of isobaric/isomeric interferece confirmed	Yes
Type of negative (precursor)ion	[M-H]-	Model for separation prediction	Yes
Fragments for identification		Additional dimension/techniques	-

Fragment name
Fatty acid fragment

Isotope correction at MS1	No	Lipid Identification Software	MS-DIAL
Isotope correction at MS2	No	Data manipulation	Smoothing, Centroiding
MS1 verified by standard	No	Nomenclature for intact lipid molecule	Yes
MS2 verified by standard	No	Nomenclature for fragment ions	No
Background check at MS1	Yes	Further identification remarks	-

38) Diacylglyceryl glucuronide (DGGA)[M-H]- / Lipid quantification

Quantitative	Yes	Limit of quantification	No
MS Level for quantification	MS1	Normalization to reference	No
Internal lipid standard(s) MS1		Lipid Quantification Software	MS-DIAL

Internal standard	Endogenous subclass
LPC 18:1(d7)	DGGA subclass

Type of quantification	Internal standard amount	Batch correction	No
Response correction	No	Further quantification remarks	-
Type I isotope correction	No		

39) Diacylglyceryl trimethylhomoserine (DGTS)[M+H]+ / Lipid identification

Lipid class	Diacylglyceryl trimethylhomoserine (DGTS)	Background check at MS2	No
Derivatization	-	Did you presume assumptions for identification?	No
MS Level for identification	MS1, MS2	Check isomer overlap	No
Identification level	Molecular species level	RT verified by standard	Yes
Polarity mode	Positive	Separation of isobaric/isomeric interferece confirmed	Yes
Type of positive (precursor)ion	[M+H]+	Model for separation prediction	Yes
Fragments for identification		Additional dimension/techniques	-

Fragment name
Characteristic fragment (C10H22NO5+)
Characteristic fragment (C7H14NO2+)
Neutral loss of fatty acyl
Neutral loss of fatty acyl and H2O

Isotope correction at MS1	No	Lipid Identification Software	MS-DIAL
Isotope correction at MS2	No	Data manipulation	Smoothing, Centroiding
MS1 verified by standard	No	Nomenclature for intact lipid molecule	Yes
MS2 verified by standard	No	Nomenclature for fragment ions	No
Background check at MS1	Yes	Further identification remarks	-

39) Diacylglyceryl trimethylhomoserine (DGTS)[M+H]+ / Lipid quantification

Quantitative	Yes	Limit of quantification	No
MS Level for quantification	MS1	Normalization to reference	No
Internal lipid standard(s) MS1		Lipid Quantification Software	MS-DIAL

Internal standard	Endogenous subclass
LPC 18:1(d7)	DGTS subclass

Type of quantification	Internal standard amount	Batch correction	No
Response correction	No	Further quantification remarks	-
Type I isotope correction	No		

40) Diacylglyceryl-3-O-carboxyhydroxymethylcholine (DGCC)[M+H]+ / Lipid identification

Lipid class	Diacylglyceryl-3-O-carboxyhydroxymethylcholine (DGCC)	Background check at MS2	No
Derivatization	-	Did you presume assumptions for identification?	No
MS Level for identification	MS1, MS2	Check isomer overlap	No
Identification level	Molecular species level	RT verified by standard	Yes
Polarity mode	Positive	Separation of isobaric/isomeric interferece confirmed	Yes
Type of positive (precursor)ion	[M+H]+	Model for separation prediction	Yes
Fragments for identification		Additional dimension/techniques	-

Fragment name
Characteristic fragment (C6H14NO2+)
Neutral loss of fatty acyl
Neutral loss of fatty acyl and H2O

Isotope correction at MS1	No	Lipid Identification Software	MS-DIAL
Isotope correction at MS2	No	Data manipulation	Smoothing, Centroiding
MS1 verified by standard	No	Nomenclature for intact lipid molecule	Yes
MS2 verified by standard	No	Nomenclature for fragment ions	No
Background check at MS1	Yes	Further identification remarks	-

40) Diacylglyceryl-3-O-carboxyhydroxymethylcholine (DGCC)[M+H]+ / Lipid quantification

Quantitative	Yes	Limit of quantification	No
MS Level for quantification	MS1	Normalization to reference	No
Internal lipid standard(s) MS1		Lipid Quantification Software	MS-DIAL

Internal standard	Endogenous subclass
LPC 18:1(d7)	DGCC subclass

Type of quantification	Internal standard amount	Batch correction	No
Response correction	No	Further quantification remarks	-
Type I isotope correction	No		

41) Digalactosylmonoacylglycerol (DGMG)[M+CH3COO]- / Lipid identification

Lipid class	Digalactosylmonoacylglycerol (DGMG)	Background check at MS2	No
Derivatization	-	Did you presume assumptions for identification?	No
MS Level for identification	MS1, MS2	Check isomer overlap	No
Identification level	Molecular species level	RT verified by standard	Yes
Polarity mode	Negative	Separation of isobaric/isomeric interferece confirmed	Yes
Type of negative (precursor)ion	[M+CH3COO]-	Model for separation prediction	Yes
Fragments for identification		Additional dimension/techniques	-

Fragment name
Fatty acid fragment

Isotope correction at MS1	No	Lipid Identification Software	MS-DIAL
Isotope correction at MS2	No	Data manipulation	Smoothing, Centroiding
MS1 verified by standard	No	Nomenclature for intact lipid molecule	Yes
MS2 verified by standard	No	Nomenclature for fragment ions	No
Background check at MS1	Yes	Further identification remarks	-

41) Digalactosylmonoacylglycerol (DGMG)[M+CH3COO]- / Lipid quantification

Quantitative	Yes	Limit of quantification	No
MS Level for quantification	MS1	Normalization to reference	No
Internal lipid standard(s) MS1		Lipid Quantification Software	MS-DIAL

Internal standard	Endogenous subclass
LPC 18:1(d7)	DGMG subclass

Type of quantification	Internal standard amount	Batch correction	No
Response correction	No	Further quantification remarks	-
Type I isotope correction	No		

42) Hex2Cer[M+H]+ / Lipid identification

Lipid class	Hex2Cer	Background check at MS2	No
Derivatization	-	Did you presume assumptions for identification?	No
MS Level for identification	MS1, MS2	Check isomer overlap	No
Identification level	Molecular species level	RT verified by standard	Yes
Polarity mode	Positive	Separation of isobaric/isomeric interferece confirmed	Yes
Type of positive (precursor)ion	[M+H]+	Model for separation prediction	Yes
Fragments for identification		Additional dimension/techniques	-

Fragment name
Neutral loss of hexose
Neutral loss of 2hexose
Sphingosine -H2O fragment
Sphingosine -2H2O fragment
Sphingosine -CH4O2 fragment

Isotope correction at MS1	No	Lipid Identification Software	MS-DIAL
Isotope correction at MS2	No	Data manipulation	Smoothing, Centroiding
MS1 verified by standard	No	Nomenclature for intact lipid molecule	Yes
MS2 verified by standard	No	Nomenclature for fragment ions	No
Background check at MS1	Yes	Further identification remarks	-

42) Hex2Cer[M+H]+ / Lipid quantification

Quantitative	Yes	Limit of quantification	No
MS Level for quantification	MS1	Normalization to reference	No
Internal lipid standard(s) MS1		Lipid Quantification Software	MS-DIAL

Internal standard	Endogenous subclass
Cer 18:1;2O/15:0(d7)	Hex2Cer subclass

Type of quantification	Internal standard amount	Batch correction	No
Response correction	No	Further quantification remarks	-
Type I isotope correction	No		

43) Dilysocardiolipin (DLCL)[M-H]- / Lipid identification

Lipid class	Dilysocardiolipin (DLCL)	Background check at MS2	No
Derivatization	-	Did you presume assumptions for identification?	No
MS Level for identification	MS1, MS2	Check isomer overlap	No
Identification level	Molecular species level	RT verified by standard	Yes
Polarity mode	Negative	Separation of isobaric/isomeric interferece confirmed	Yes
Type of negative (precursor)ion	[M-H]-	Model for separation prediction	Yes
Fragments for identification		Additional dimension/techniques	-

Fragment name
Phosphoglycerol -H2O fragment
lysophosphatidic acid

Isotope correction at MS1	No	Lipid Identification Software	MS-DIAL
Isotope correction at MS2	No	Data manipulation	Smoothing, Centroiding
MS1 verified by standard	No	Nomenclature for intact lipid molecule	Yes
MS2 verified by standard	No	Nomenclature for fragment ions	No
Background check at MS1	Yes	Further identification remarks	-

43) Dilysocardiolipin (DLCL)[M-H]- / Lipid quantification

Quantitative	Yes	Limit of quantification	No
MS Level for quantification	MS1	Normalization to reference	No
Internal lipid standard(s) MS1		Lipid Quantification Software	MS-DIAL

Internal standard	Endogenous subclass
PG 15:0_18:1(d7)	DLCL subclass

Type of quantification	Internal standard amount	Batch correction	No
Response correction	No	Further quantification remarks	-
Type I isotope correction	No		

44) Ergosterol ester (EGSE)[M+NH4]+ / Lipid identification

Lipid class	Ergosterol ester (EGSE)	Background check at MS2	No
Derivatization	-	Did you presume assumptions for identification?	No
MS Level for identification	MS1, MS2	Check isomer overlap	No
Identification level	Molecular species level	RT verified by standard	Yes
Polarity mode	Positive	Separation of isobaric/isomeric interferece confirmed	Yes
Type of positive (precursor)ion	[M+NH4]+	Model for separation prediction	Yes
Fragments for identification		Additional dimension/techniques	-

Fragment name
Neutral loss of fatty acyl

Isotope correction at MS1	No	Lipid Identification Software	MS-DIAL
Isotope correction at MS2	No	Data manipulation	Smoothing, Centroiding
MS1 verified by standard	No	Nomenclature for intact lipid molecule	Yes
MS2 verified by standard	No	Nomenclature for fragment ions	No
Background check at MS1	Yes	Further identification remarks	-

44) Ergosterol ester (EGSE)[M+NH4]+ / Lipid quantification

Quantitative	Yes	Limit of quantification	No
MS Level for quantification	MS1	Normalization to reference	No
Internal lipid standard(s) MS1		Lipid Quantification Software	MS-DIAL

Internal standard	Endogenous subclass
CE 18:1(d7)	EGSE subclass

Type of quantification	Internal standard amount	Batch correction	No
Response correction	No	Further quantification remarks	-
Type I isotope correction	No		

45) Esterified ketodeoxycholic acid (KDCAE)[M+NH4]+ / Lipid identification

Lipid class	Esterified ketodeoxycholic acid (KDCAE)	Background check at MS2	No
Derivatization	-	Did you presume assumptions for identification?	No
MS Level for identification	MS1, MS2	Check isomer overlap	No
Identification level	Molecular species level	RT verified by standard	Yes
Polarity mode	Positive	Separation of isobaric/isomeric interferece confirmed	Yes
Type of positive (precursor)ion	[M+NH4]+	Model for separation prediction	Yes
Fragments for identification		Additional dimension/techniques	-

Fragment name
Neutral loss of fatty acyl

Isotope correction at MS1	No	Lipid Identification Software	MS-DIAL
Isotope correction at MS2	No	Data manipulation	Smoothing, Centroiding
MS1 verified by standard	No	Nomenclature for intact lipid molecule	Yes
MS2 verified by standard	No	Nomenclature for fragment ions	No
Background check at MS1	Yes	Further identification remarks	-

45) Esterified ketodeoxycholic acid (KDCAE)[M+NH4]+ / Lipid quantification

Quantitative	Yes	Limit of quantification	No
MS Level for quantification	MS1	Normalization to reference	No
Internal lipid standard(s) MS1		Lipid Quantification Software	MS-DIAL

Internal standard	Endogenous subclass
CE 18:1(d7)	KDCAE subclass

Type of quantification	Internal standard amount	Batch correction	No
Response correction	No	Further quantification remarks	-
Type I isotope correction	No		

46) Esterified taurodeoxycholic Acid (TDCAE)[M+NH4]+ / Lipid identification

Lipid class	Esterified taurodeoxycholic Acid (TDCAE)	Background check at MS2	No
Derivatization	-	Did you presume assumptions for identification?	No
MS Level for identification	MS1, MS2	Check isomer overlap	No
Identification level	Molecular species level	RT verified by standard	Yes
Polarity mode	Positive	Separation of isobaric/isomeric interferece confirmed	Yes
Type of positive (precursor)ion	[M+NH4]+	Model for separation prediction	Yes
Fragments for identification		Additional dimension/techniques	-

Fragment name
Neutral loss of fatty acyl

Isotope correction at MS1	No	Lipid Identification Software	MS-DIAL
Isotope correction at MS2	No	Data manipulation	Smoothing, Centroiding
MS1 verified by standard	No	Nomenclature for intact lipid molecule	Yes
MS2 verified by standard	No	Nomenclature for fragment ions	No
Background check at MS1	Yes	Further identification remarks	-

46) Esterified taurodeoxycholic Acid (TDCAE)[M+NH4]+ / Lipid quantification

Quantitative	Yes	Limit of quantification	No
MS Level for quantification	MS1	Normalization to reference	No
Internal lipid standard(s) MS1		Lipid Quantification Software	MS-DIAL

Internal standard	Endogenous subclass
CE 18:1(d7)	TDCAE subclass

Type of quantification	Internal standard amount	Batch correction	No
Response correction	No	Further quantification remarks	-
Type I isotope correction	No		

47) Esterified deoxycholic acid (DCAE)[M+NH4]+ / Lipid identification

Lipid class	Esterified deoxycholic acid (DCAE)	Background check at MS2	No
Derivatization	-	Did you presume assumptions for identification?	No
MS Level for identification	MS1, MS2	Check isomer overlap	No
Identification level	Molecular species level	RT verified by standard	Yes
Polarity mode	Positive	Separation of isobaric/isomeric interferece confirmed	Yes
Type of positive (precursor)ion	[M+NH4]+	Model for separation prediction	Yes
Fragments for identification		Additional dimension/techniques	-

Fragment name
Neutral loss of fatty acyl

Isotope correction at MS1	No	Lipid Identification Software	MS-DIAL
Isotope correction at MS2	No	Data manipulation	Smoothing, Centroiding
MS1 verified by standard	No	Nomenclature for intact lipid molecule	Yes
MS2 verified by standard	No	Nomenclature for fragment ions	No
Background check at MS1	Yes	Further identification remarks	-

47) Esterified deoxycholic acid (DCAE)[M+NH4]+ / Lipid quantification

Quantitative	Yes	Limit of quantification	No
MS Level for quantification	MS1	Normalization to reference	No
Internal lipid standard(s) MS1		Lipid Quantification Software	MS-DIAL

Internal standard	Endogenous subclass
CE 18:1(d7)	DCAE subclass

Type of quantification	Internal standard amount	Batch correction	No
Response correction	No	Further quantification remarks	-
Type I isotope correction	No		

48) Esterified ketolithocholic acid (KLCAE)[M+NH4]+ / Lipid identification

Lipid class	Esterified ketolithocholic acid (KLCAE)	Background check at MS2	No
Derivatization	-	Did you presume assumptions for identification?	No
MS Level for identification	MS1, MS2	Check isomer overlap	No
Identification level	Molecular species level	RT verified by standard	Yes
Polarity mode	Positive	Separation of isobaric/isomeric interferece confirmed	Yes
Type of positive (precursor)ion	[M+NH4]+	Model for separation prediction	Yes
Fragments for identification		Additional dimension/techniques	-

Fragment name
Neutral loss of fatty acyl

Isotope correction at MS1	No	Lipid Identification Software	MS-DIAL
Isotope correction at MS2	No	Data manipulation	Smoothing, Centroiding
MS1 verified by standard	No	Nomenclature for intact lipid molecule	Yes
MS2 verified by standard	No	Nomenclature for fragment ions	No
Background check at MS1	Yes	Further identification remarks	-

48) Esterified ketolithocholic acid (KLCAE)[M+NH4]+ / Lipid quantification

Quantitative	Yes	Limit of quantification	No
MS Level for quantification	MS1	Normalization to reference	No
Internal lipid standard(s) MS1		Lipid Quantification Software	MS-DIAL

Internal standard	Endogenous subclass
CE 18:1(d7)	KLCAE subclass

Type of quantification	Internal standard amount	Batch correction	No
Response correction	No	Further quantification remarks	-
Type I isotope correction	No		

49) Esterified lithocholic acid (LCAE)[M+NH4]+ / Lipid identification

Lipid class	Esterified lithocholic acid (LCAE)	Background check at MS2	No
Derivatization	-	Did you presume assumptions for identification?	No
MS Level for identification	MS1, MS2	Check isomer overlap	No
Identification level	Molecular species level	RT verified by standard	Yes
Polarity mode	Positive	Separation of isobaric/isomeric interferece confirmed	Yes
Type of positive (precursor)ion	[M+NH4]+	Model for separation prediction	Yes
Fragments for identification		Additional dimension/techniques	-

Fragment name
Neutral loss of fatty acyl

Isotope correction at MS1	No	Lipid Identification Software	MS-DIAL
Isotope correction at MS2	No	Data manipulation	Smoothing, Centroiding
MS1 verified by standard	No	Nomenclature for intact lipid molecule	Yes
MS2 verified by standard	No	Nomenclature for fragment ions	No
Background check at MS1	Yes	Further identification remarks	-

49) Esterified lithocholic acid (LCAE)[M+NH4]+ / Lipid quantification

Quantitative	Yes	Limit of quantification	No
MS Level for quantification	MS1	Normalization to reference	No
Internal lipid standard(s) MS1		Lipid Quantification Software	MS-DIAL

Internal standard	Endogenous subclass
CE 18:1(d7)	LCAE subclass

Type of quantification	Internal standard amount	Batch correction	No
Response correction	No	Further quantification remarks	-
Type I isotope correction	No		

50) Ether-linked digalactosyldiacylglycerol (EtherDGDG)[M+CH3COO]- / Lipid identification

Lipid class	Ether-linked digalactosyldiacylglycerol (EtherDGDG)	Background check at MS2	No
Derivatization	-	Did you presume assumptions for identification?	No
MS Level for identification	MS1, MS2	Check isomer overlap	No
Identification level	Molecular species level	RT verified by standard	Yes
Polarity mode	Negative	Separation of isobaric/isomeric interferece confirmed	Yes
Type of negative (precursor)ion	[M+CH3COO]-	Model for separation prediction	Yes
Fragments for identification		Additional dimension/techniques	-

Fragment name
Neutral loss of fatty acyl
Fatty acid fragment

Isotope correction at MS1	No	Lipid Identification Software	MS-DIAL
Isotope correction at MS2	No	Data manipulation	Smoothing, Centroiding
MS1 verified by standard	No	Nomenclature for intact lipid molecule	Yes
MS2 verified by standard	No	Nomenclature for fragment ions	No
Background check at MS1	Yes	Further identification remarks	-

50) Ether-linked digalactosyldiacylglycerol (EtherDGDG)[M+CH3COO]- / Lipid quantification

Quantitative	Yes	Limit of quantification	No
MS Level for quantification	MS1	Normalization to reference	No
Internal lipid standard(s) MS1		Lipid Quantification Software	MS-DIAL

Internal standard	Endogenous subclass
LPC 18:1(d7)	EtherDGDG subclass

Type of quantification	Internal standard amount	Batch correction	No
Response correction	No	Further quantification remarks	-
Type I isotope correction	No		

51) LPC O[M+H]+ / Lipid identification

Lipid class	LPC O	Background check at MS2	No
Derivatization	-	Did you presume assumptions for identification?	No
MS Level for identification	MS1, MS2	Check isomer overlap	No
Identification level	Molecular species level	RT verified by standard	Yes
Polarity mode	Positive	Separation of isobaric/isomeric interferece confirmed	Yes
Type of positive (precursor)ion	[M+H]+	Model for separation prediction	Yes
Fragments for identification		Additional dimension/techniques	-

Fragment name
Characteristic fragments (C5H14NO+)
Characteristic fragments (C2H6O4P+)
Characteristic fragments (C5H15NO4P+)

Isotope correction at MS1	No	Lipid Identification Software	MS-DIAL
Isotope correction at MS2	No	Data manipulation	Smoothing, Centroiding
MS1 verified by standard	No	Nomenclature for intact lipid molecule	Yes
MS2 verified by standard	No	Nomenclature for fragment ions	No
Background check at MS1	Yes	Further identification remarks	-

51) LPC O[M+H]+ / Lipid quantification

Quantitative	Yes	Limit of quantification	No
MS Level for quantification	MS1	Normalization to reference	No
Internal lipid standard(s) MS1		Lipid Quantification Software	MS-DIAL

Internal standard	Endogenous subclass
LPC 18:1(d7)	EtherLPC subclass

Type of quantification	Internal standard amount	Batch correction	No
Response correction	No	Further quantification remarks	-
Type I isotope correction	No		

52) LPE O[M+H]+ / Lipid identification

Lipid class	LPE O	Background check at MS2	No
Derivatization	-	Did you presume assumptions for identification?	No
MS Level for identification	MS1, MS2	Check isomer overlap	No
Identification level	Molecular species level	RT verified by standard	Yes
Polarity mode	Positive	Separation of isobaric/isomeric interferece confirmed	Yes
Type of positive (precursor)ion	[M+H]+	Model for separation prediction	Yes
Fragments for identification		Additional dimension/techniques	-

Fragment name
Neutral loss of C3H8NO4P
Neutral loss of C3H10NO5P

Isotope correction at MS1	No	Lipid Identification Software	MS-DIAL
Isotope correction at MS2	No	Data manipulation	Smoothing, Centroiding
MS1 verified by standard	No	Nomenclature for intact lipid molecule	Yes
MS2 verified by standard	No	Nomenclature for fragment ions	No
Background check at MS1	Yes	Further identification remarks	-

52) LPE O[M+H]+ / Lipid quantification

Quantitative	Yes	Limit of quantification	No
MS Level for quantification	MS1	Normalization to reference	No
Internal lipid standard(s) MS1		Lipid Quantification Software	MS-DIAL

Internal standard	Endogenous subclass
LPE 18:1(d7)	EtherLPE subclass

Type of quantification	Internal standard amount	Batch correction	No
Response correction	No	Further quantification remarks	-
Type I isotope correction	No		

53) Ether-linked lysophosphatidylglycerol (EtherLPG)[M-H]- / Lipid identification

Lipid class	Ether-linked lysophosphatidylglycerol (EtherLPG)	Background check at MS2	No
Derivatization	-	Did you presume assumptions for identification?	No
MS Level for identification	MS1, MS2	Check isomer overlap	No
Identification level	Molecular species level	RT verified by standard	Yes
Polarity mode	Negative	Separation of isobaric/isomeric interferece confirmed	Yes
Type of negative (precursor)ion	[M-H]-	Model for separation prediction	Yes
Fragments for identification		Additional dimension/techniques	-

Fragment name
Phosphoglycerol -H2O fragment
Alkyl Ether fragment

Isotope correction at MS1	No	Lipid Identification Software	MS-DIAL
Isotope correction at MS2	No	Data manipulation	Smoothing, Centroiding
MS1 verified by standard	No	Nomenclature for intact lipid molecule	Yes
MS2 verified by standard	No	Nomenclature for fragment ions	No
Background check at MS1	Yes	Further identification remarks	-

53) Ether-linked lysophosphatidylglycerol (EtherLPG)[M-H]- / Lipid quantification

Quantitative	Yes	Limit of quantification	No
MS Level for quantification	MS1	Normalization to reference	No
Internal lipid standard(s) MS1		Lipid Quantification Software	MS-DIAL

Internal standard	Endogenous subclass
PG 15:0_18:1(d7)	EtherLPG subclass

Type of quantification	Internal standard amount	Batch correction	No
Response correction	No	Further quantification remarks	-
Type I isotope correction	No		

54) Ether-linked monogalactosyldiacylglycerol (EtherMGDG)[M+CH3COO]- / Lipid identification

Lipid class	Ether-linked monogalactosyldiacylglycerol (EtherMGDG)	Background check at MS2	No
Derivatization	-	Did you presume assumptions for identification?	No
MS Level for identification	MS1, MS2	Check isomer overlap	No
Identification level	Molecular species level	RT verified by standard	Yes
Polarity mode	Negative	Separation of isobaric/isomeric interferece confirmed	Yes
Type of negative (precursor)ion	[M+CH3COO]-	Model for separation prediction	Yes
Fragments for identification		Additional dimension/techniques	-

Fragment name
Neutral loss of fatty acyl
Fatty acid fragment

Isotope correction at MS1	No	Lipid Identification Software	MS-DIAL
Isotope correction at MS2	No	Data manipulation	Smoothing, Centroiding
MS1 verified by standard	No	Nomenclature for intact lipid molecule	Yes
MS2 verified by standard	No	Nomenclature for fragment ions	No
Background check at MS1	Yes	Further identification remarks	-

54) Ether-linked monogalactosyldiacylglycerol (EtherMGDG)[M+CH3COO]- / Lipid quantification

Quantitative	Yes	Limit of quantification	No
MS Level for quantification	MS1	Normalization to reference	No
Internal lipid standard(s) MS1		Lipid Quantification Software	MS-DIAL

Internal standard	Endogenous subclass
LPC 18:1(d7)	EtherMGDG subclass

Type of quantification	Internal standard amount	Batch correction	No
Response correction	No	Further quantification remarks	-
Type I isotope correction	No		

55) Ether-linked oxidized phosphatidylcholine (EtherOxPC)[M+CH3COO]- / Lipid identification

Lipid class	Ether-linked oxidized phosphatidylcholine (EtherOxPC)	Background check at MS2	No
Derivatization	-	Did you presume assumptions for identification?	No
MS Level for identification	MS1, MS2	Check isomer overlap	No
Identification level	Molecular species level	RT verified by standard	Yes
Polarity mode	Negative	Separation of isobaric/isomeric interferece confirmed	Yes
Type of negative (precursor)ion	[M+CH3COO]-	Model for separation prediction	Yes
Fragments for identification		Additional dimension/techniques	-

Fragment name
Oxidized fatty acid fragment
Oxidized fatty acid -H2O fragment
Neutral loss of methyl moiety

Isotope correction at MS1	No	Lipid Identification Software	MS-DIAL
Isotope correction at MS2	No	Data manipulation	Smoothing, Centroiding
MS1 verified by standard	No	Nomenclature for intact lipid molecule	Yes
MS2 verified by standard	No	Nomenclature for fragment ions	No
Background check at MS1	Yes	Further identification remarks	-

55) Ether-linked oxidized phosphatidylcholine (EtherOxPC)[M+CH3COO]- / Lipid quantification

Quantitative	Yes	Limit of quantification	No
MS Level for quantification	MS1	Normalization to reference	No
Internal lipid standard(s) MS1		Lipid Quantification Software	MS-DIAL

Internal standard	Endogenous subclass
PC 15:0_18:1(d7)	EtherOxPC subclass

Type of quantification	Internal standard amount	Batch correction	No
Response correction	No	Further quantification remarks	-
Type I isotope correction	No		

56) Ether-linked oxidized phosphatidylethanolamine (EtherOxPE)[M-H]- / Lipid identification

Lipid class	Ether-linked oxidized phosphatidylethanolamine (EtherOxPE)	Background check at MS2	No
Derivatization	-	Did you presume assumptions for identification?	No
MS Level for identification	MS1, MS2	Check isomer overlap	No
Identification level	Molecular species level	RT verified by standard	Yes
Polarity mode	Negative	Separation of isobaric/isomeric interferece confirmed	Yes
Type of negative (precursor)ion	[M-H]-	Model for separation prediction	Yes
Fragments for identification		Additional dimension/techniques	-

Fragment name
Oxidized fatty acid fragment
Oxidized fatty acid -H2O fragment

Isotope correction at MS1	No	Lipid Identification Software	MS-DIAL
Isotope correction at MS2	No	Data manipulation	Smoothing, Centroiding
MS1 verified by standard	No	Nomenclature for intact lipid molecule	Yes
MS2 verified by standard	No	Nomenclature for fragment ions	No
Background check at MS1	Yes	Further identification remarks	-

56) Ether-linked oxidized phosphatidylethanolamine (EtherOxPE)[M-H]- / Lipid quantification

Quantitative	Yes	Limit of quantification	No
MS Level for quantification	MS1	Normalization to reference	No
Internal lipid standard(s) MS1		Lipid Quantification Software	MS-DIAL

Internal standard	Endogenous subclass
PE 15:0_18:1(d7)	EtherOxPE subclass

Type of quantification	Internal standard amount	Batch correction	No
Response correction	No	Further quantification remarks	-
Type I isotope correction	No		

57) PC O[M+CH3COO]- / Lipid identification

Lipid class	PC O	Background check at MS2	No
Derivatization	-	Did you presume assumptions for identification?	No
MS Level for identification	MS1, MS2	Check isomer overlap	No
Identification level	Molecular species level	RT verified by standard	Yes
Polarity mode	Negative	Separation of isobaric/isomeric interferece confirmed	Yes
Type of negative (precursor)ion	[M+CH3COO]-	Model for separation prediction	Yes
Fragments for identification		Additional dimension/techniques	-

Fragment name
Neutral loss of methyl moiety
Fatty acid fragment

Isotope correction at MS1	No	Lipid Identification Software	MS-DIAL
Isotope correction at MS2	No	Data manipulation	Smoothing, Centroiding
MS1 verified by standard	No	Nomenclature for intact lipid molecule	Yes
MS2 verified by standard	No	Nomenclature for fragment ions	No
Background check at MS1	Yes	Further identification remarks	-

57) PC O[M+CH3COO]- / Lipid quantification

Quantitative	Yes	Limit of quantification	No
MS Level for quantification	MS1	Normalization to reference	No
Internal lipid standard(s) MS1		Lipid Quantification Software	MS-DIAL

Internal standard	Endogenous subclass
PC 15:0_18:1(d7)	EtherPC subclass

Type of quantification	Internal standard amount	Batch correction	No
Response correction	No	Further quantification remarks	-
Type I isotope correction	No		

58) PE O[M-H]- / Lipid identification

Lipid class	PE O	Background check at MS2	No
Derivatization	-	Did you presume assumptions for identification?	No
MS Level for identification	MS1, MS2	Check isomer overlap	No
Identification level	Molecular species level	RT verified by standard	Yes
Polarity mode	Negative	Separation of isobaric/isomeric interferece confirmed	Yes
Type of negative (precursor)ion	[M-H]-	Model for separation prediction	Yes
Fragments for identification		Additional dimension/techniques	-

Fragment name
Neutral loss of fatty acyl
Fatty acid fragment

Isotope correction at MS1	No	Lipid Identification Software	MS-DIAL
Isotope correction at MS2	No	Data manipulation	Smoothing, Centroiding
MS1 verified by standard	No	Nomenclature for intact lipid molecule	Yes
MS2 verified by standard	No	Nomenclature for fragment ions	No
Background check at MS1	Yes	Further identification remarks	-

58) PE O[M-H]- / Lipid quantification

Quantitative	Yes	Limit of quantification	No
MS Level for quantification	MS1	Normalization to reference	No
Internal lipid standard(s) MS1		Lipid Quantification Software	MS-DIAL

Internal standard	Endogenous subclass
PE 15:0_18:1(d7)	EtherPE subclass

Type of quantification	Internal standard amount	Batch correction	No
Response correction	No	Further quantification remarks	-
Type I isotope correction	No		

59) Ether-linked phosphatidylglycerol (EtherPG)[M-H]- / Lipid identification

Lipid class	Ether-linked phosphatidylglycerol (EtherPG)	Background check at MS2	No
Derivatization	-	Did you presume assumptions for identification?	No
MS Level for identification	MS1, MS2	Check isomer overlap	No
Identification level	Molecular species level	RT verified by standard	Yes
Polarity mode	Negative	Separation of isobaric/isomeric interferece confirmed	Yes
Type of negative (precursor)ion	[M-H]-	Model for separation prediction	Yes
Fragments for identification		Additional dimension/techniques	-

Fragment name
Phosphoglycerol -H2O
Fatty acid fragment
Alkyl ether +O fragment

Isotope correction at MS1	No	Lipid Identification Software	MS-DIAL
Isotope correction at MS2	No	Data manipulation	Smoothing, Centroiding
MS1 verified by standard	No	Nomenclature for intact lipid molecule	Yes
MS2 verified by standard	No	Nomenclature for fragment ions	No
Background check at MS1	Yes	Further identification remarks	-

59) Ether-linked phosphatidylglycerol (EtherPG)[M-H]- / Lipid quantification

Quantitative	Yes	Limit of quantification	No
MS Level for quantification	MS1	Normalization to reference	No
Internal lipid standard(s) MS1		Lipid Quantification Software	MS-DIAL

Internal standard	Endogenous subclass
PG 15:0_18:1(d7)	EtherPG subclass

Type of quantification	Internal standard amount	Batch correction	No
Response correction	No	Further quantification remarks	-
Type I isotope correction	No		

60) Ether-linked phosphatidylinositol (EtherPI)[M-H]- / Lipid identification

Lipid class	Ether-linked phosphatidylinositol (EtherPI)	Background check at MS2	No
Derivatization	-	Did you presume assumptions for identification?	No
MS Level for identification	MS1, MS2	Check isomer overlap	No
Identification level	Molecular species level	RT verified by standard	Yes
Polarity mode	Negative	Separation of isobaric/isomeric interferece confirmed	Yes
Type of negative (precursor)ion	[M-H]-	Model for separation prediction	Yes
Fragments for identification		Additional dimension/techniques	-

Fragment name
Phosphoinositol -H2O
Fatty acid fragment
Alkyl ether + C3H5O4P fragment

Isotope correction at MS1	No	Lipid Identification Software	MS-DIAL
Isotope correction at MS2	No	Data manipulation	Smoothing, Centroiding
MS1 verified by standard	No	Nomenclature for intact lipid molecule	Yes
MS2 verified by standard	No	Nomenclature for fragment ions	No
Background check at MS1	Yes	Further identification remarks	-

60) Ether-linked phosphatidylinositol (EtherPI)[M-H]- / Lipid quantification

Quantitative	Yes	Limit of quantification	No
MS Level for quantification	MS1	Normalization to reference	No
Internal lipid standard(s) MS1		Lipid Quantification Software	MS-DIAL

Internal standard	Endogenous subclass
PI 15:0_18:1(d7)	EtherPI subclass

Type of quantification	Internal standard amount	Batch correction	No
Response correction	No	Further quantification remarks	-
Type I isotope correction	No		

61) Ether-linked phosphatidylserine (EtherPS)[M-H]- / Lipid identification

Lipid class	Ether-linked phosphatidylserine (EtherPS)	Background check at MS2	No
Derivatization	-	Did you presume assumptions for identification?	No
MS Level for identification	MS1, MS2	Check isomer overlap	No
Identification level	Molecular species level	RT verified by standard	Yes
Polarity mode	Negative	Separation of isobaric/isomeric interferece confirmed	Yes
Type of negative (precursor)ion	[M-H]-	Model for separation prediction	Yes
Fragments for identification		Additional dimension/techniques	-

Fragment name
Neutral loss of C3H5NO2
Neutral loss of 22:6 Acyl and H2O and C3H5NO2
Fatty acid fragment

Isotope correction at MS1	No	Lipid Identification Software	MS-DIAL
Isotope correction at MS2	No	Data manipulation	Smoothing, Centroiding
MS1 verified by standard	No	Nomenclature for intact lipid molecule	Yes
MS2 verified by standard	No	Nomenclature for fragment ions	No
Background check at MS1	Yes	Further identification remarks	-

61) Ether-linked phosphatidylserine (EtherPS)[M-H]- / Lipid quantification

Quantitative	Yes	Limit of quantification	No
MS Level for quantification	MS1	Normalization to reference	No
Internal lipid standard(s) MS1		Lipid Quantification Software	MS-DIAL

Internal standard	Endogenous subclass
PS 15:0_18:1(d7)	EtherPS subclass

Type of quantification	Internal standard amount	Batch correction	No
Response correction	No	Further quantification remarks	-
Type I isotope correction	No		

62) Ether-linked triacylglycerol (EtherTG)[M+NH4]+ / Lipid identification

Lipid class	Ether-linked triacylglycerol (EtherTG)	Background check at MS2	No
Derivatization	-	Did you presume assumptions for identification?	No
MS Level for identification	MS1, MS2	Check isomer overlap	No
Identification level	Molecular species level	RT verified by standard	Yes
Polarity mode	Positive	Separation of isobaric/isomeric interferece confirmed	Yes
Type of positive (precursor)ion	[M+NH4]+	Model for separation prediction	Yes
Fragments for identification		Additional dimension/techniques	-

Fragment name
Neutral loss of fatty acyl and H2O
Neutral loss of alkyl ether

Isotope correction at MS1	No	Lipid Identification Software	MS-DIAL
Isotope correction at MS2	No	Data manipulation	Smoothing, Centroiding
MS1 verified by standard	No	Nomenclature for intact lipid molecule	Yes
MS2 verified by standard	No	Nomenclature for fragment ions	No
Background check at MS1	Yes	Further identification remarks	-

62) Ether-linked triacylglycerol (EtherTG)[M+NH4]+ / Lipid quantification

Quantitative	Yes	Limit of quantification	No
MS Level for quantification	MS1	Normalization to reference	No
Internal lipid standard(s) MS1		Lipid Quantification Software	MS-DIAL

Internal standard	Endogenous subclass
TG 15:0_18:1(d7)_15:0	EtherTG subclass

Type of quantification	Internal standard amount	Batch correction	No
Response correction	No	Further quantification remarks	-
Type I isotope correction	No		

63) Fatty acid ester of hydroxyl fatty acid (FAHFA)[M-H]- / Lipid identification

Lipid class	Fatty acid ester of hydroxyl fatty acid (FAHFA)	Background check at MS2	No
Derivatization	-	Did you presume assumptions for identification?	No
MS Level for identification	MS1, MS2	Check isomer overlap	No
Identification level	Molecular species level	RT verified by standard	Yes
Polarity mode	Negative	Separation of isobaric/isomeric interferece confirmed	Yes
Type of negative (precursor)ion	[M-H]-	Model for separation prediction	Yes
Fragments for identification		Additional dimension/techniques	-

Fragment name
Neutral loss of fatty acyl and H2O
Fatty acid fragment

Isotope correction at MS1	No	Lipid Identification Software	MS-DIAL
Isotope correction at MS2	No	Data manipulation	Smoothing, Centroiding
MS1 verified by standard	No	Nomenclature for intact lipid molecule	Yes
MS2 verified by standard	No	Nomenclature for fragment ions	No
Background check at MS1	Yes	Further identification remarks	-

63) Fatty acid ester of hydroxyl fatty acid (FAHFA)[M-H]- / Lipid quantification

Quantitative	Yes	Limit of quantification	No
MS Level for quantification	MS1	Normalization to reference	No
Internal lipid standard(s) MS1		Lipid Quantification Software	MS-DIAL

Internal standard	Endogenous subclass
FA 18:0(d3)	FAHFA subclass

Type of quantification	Internal standard amount	Batch correction	No
Response correction	No	Further quantification remarks	-
Type I isotope correction	No		

64) Ganglioside GD1a (GD1a)[M-H]- / Lipid identification

Lipid class	Ganglioside GD1a (GD1a)	Background check at MS2	No
Derivatization	-	Did you presume assumptions for identification?	No
MS Level for identification	MS1, MS2	Check isomer overlap	No
Identification level	Species level	RT verified by standard	Yes
Polarity mode	Negative	Separation of isobaric/isomeric interferece confirmed	Yes
Type of negative (precursor)ion	[M-H]-	Model for separation prediction	Yes
Fragments for identification		Additional dimension/techniques	-

Fragment name
Characteristic fragment (C11H16NO8-)

Isotope correction at MS1	No	Lipid Identification Software	MS-DIAL
Isotope correction at MS2	No	Data manipulation	Smoothing, Centroiding
MS1 verified by standard	No	Nomenclature for intact lipid molecule	Yes
MS2 verified by standard	No	Nomenclature for fragment ions	No
Background check at MS1	Yes	Further identification remarks	-

64) Ganglioside GD1a (GD1a)[M-H]- / Lipid quantification

Quantitative	Yes	Limit of quantification	No
MS Level for quantification	MS1	Normalization to reference	No
Internal lipid standard(s) MS1		Lipid Quantification Software	MS-DIAL

Internal standard	Endogenous subclass
LPC 18:1(d7)	GD1a subclass

Type of quantification	Internal standard amount	Batch correction	No
Response correction	No	Further quantification remarks	-
Type I isotope correction	No		

65) Ganglioside GD1b (GD1b)[M-H]- / Lipid identification

Lipid class	Ganglioside GD1b (GD1b)	Background check at MS2	No
Derivatization	-	Did you presume assumptions for identification?	No
MS Level for identification	MS1, MS2	Check isomer overlap	No
Identification level	Species level	RT verified by standard	Yes
Polarity mode	Negative	Separation of isobaric/isomeric interferece confirmed	Yes
Type of negative (precursor)ion	[M-H]-	Model for separation prediction	Yes
Fragments for identification		Additional dimension/techniques	-

Fragment name
Characteristic fragment (C11H16NO8-)

Isotope correction at MS1	No	Lipid Identification Software	MS-DIAL
Isotope correction at MS2	No	Data manipulation	Smoothing, Centroiding
MS1 verified by standard	No	Nomenclature for intact lipid molecule	Yes
MS2 verified by standard	No	Nomenclature for fragment ions	No
Background check at MS1	Yes	Further identification remarks	-

65) Ganglioside GD1b (GD1b)[M-H]- / Lipid quantification

Quantitative	Yes	Limit of quantification	No
MS Level for quantification	MS1	Normalization to reference	No
Internal lipid standard(s) MS1		Lipid Quantification Software	MS-DIAL

Internal standard	Endogenous subclass
LPC 18:1(d7)	GD1b subclass

Type of quantification	Internal standard amount	Batch correction	No
Response correction	No	Further quantification remarks	-
Type I isotope correction	No		

66) Ganglioside GD2 (GD2)[M-H]- / Lipid identification

Lipid class	Ganglioside GD2 (GD2)	Background check at MS2	No
Derivatization	-	Did you presume assumptions for identification?	No
MS Level for identification	MS1, MS2	Check isomer overlap	No
Identification level	Species level	RT verified by standard	Yes
Polarity mode	Negative	Separation of isobaric/isomeric interferece confirmed	Yes
Type of negative (precursor)ion	[M-H]-	Model for separation prediction	Yes
Fragments for identification		Additional dimension/techniques	-

Fragment name
Characteristic fragment (C11H16NO8-)

Isotope correction at MS1	No	Lipid Identification Software	MS-DIAL
Isotope correction at MS2	No	Data manipulation	Smoothing, Centroiding
MS1 verified by standard	No	Nomenclature for intact lipid molecule	Yes
MS2 verified by standard	No	Nomenclature for fragment ions	No
Background check at MS1	Yes	Further identification remarks	-

66) Ganglioside GD2 (GD2)[M-H]- / Lipid quantification

Quantitative	Yes	Limit of quantification	No
MS Level for quantification	MS1	Normalization to reference	No
Internal lipid standard(s) MS1		Lipid Quantification Software	MS-DIAL

Internal standard	Endogenous subclass
LPC 18:1(d7)	GD2 subclass

Type of quantification	Internal standard amount	Batch correction	No
Response correction	No	Further quantification remarks	-
Type I isotope correction	No		

67) GD3[M-H]- / Lipid identification

Lipid class	GD3	Background check at MS2	No
Derivatization	-	Did you presume assumptions for identification?	No
MS Level for identification	MS1, MS2	Check isomer overlap	No
Identification level	Species level	RT verified by standard	Yes
Polarity mode	Negative	Separation of isobaric/isomeric interferece confirmed	Yes
Type of negative (precursor)ion	[M-H]-	Model for separation prediction	Yes
Fragments for identification		Additional dimension/techniques	-

Fragment name
Characteristic fragment (C11H16NO8-)

Isotope correction at MS1	No	Lipid Identification Software	MS-DIAL
Isotope correction at MS2	No	Data manipulation	Smoothing, Centroiding
MS1 verified by standard	No	Nomenclature for intact lipid molecule	Yes
MS2 verified by standard	No	Nomenclature for fragment ions	No
Background check at MS1	Yes	Further identification remarks	-

67) GD3[M-H]- / Lipid quantification

Quantitative	Yes	Limit of quantification	No
MS Level for quantification	MS1	Normalization to reference	No
Internal lipid standard(s) MS1		Lipid Quantification Software	MS-DIAL

Internal standard	Endogenous subclass
LPC 18:1(d7)	GD3 subclass

Type of quantification	Internal standard amount	Batch correction	No
Response correction	No	Further quantification remarks	-
Type I isotope correction	No		

68) GM1[M-H]- / Lipid identification

Lipid class	GM1	Background check at MS2	No
Derivatization	-	Did you presume assumptions for identification?	No
MS Level for identification	MS1, MS2	Check isomer overlap	No
Identification level	Species level	RT verified by standard	Yes
Polarity mode	Negative	Separation of isobaric/isomeric interferece confirmed	Yes
Type of negative (precursor)ion	[M-H]-	Model for separation prediction	Yes
Fragments for identification		Additional dimension/techniques	-

Fragment name
Characteristic fragment (C11H16NO8-)

Isotope correction at MS1	No	Lipid Identification Software	MS-DIAL
Isotope correction at MS2	No	Data manipulation	Smoothing, Centroiding
MS1 verified by standard	No	Nomenclature for intact lipid molecule	Yes
MS2 verified by standard	No	Nomenclature for fragment ions	No
Background check at MS1	Yes	Further identification remarks	-

68) GM1[M-H]- / Lipid quantification

Quantitative	Yes	Limit of quantification	No
MS Level for quantification	MS1	Normalization to reference	No
Internal lipid standard(s) MS1		Lipid Quantification Software	MS-DIAL

Internal standard	Endogenous subclass
LPC 18:1(d7)	GM1 subclass

Type of quantification	Internal standard amount	Batch correction	No
Response correction	No	Further quantification remarks	-
Type I isotope correction	No		

69) GM3[M+NH4]+ / Lipid identification

Lipid class	GM3	Background check at MS2	No
Derivatization	-	Did you presume assumptions for identification?	No
MS Level for identification	MS1, MS2	Check isomer overlap	No
Identification level	Species level	RT verified by standard	Yes
Polarity mode	Positive	Separation of isobaric/isomeric interferece confirmed	Yes
Type of positive (precursor)ion	[M+NH4]+	Model for separation prediction	Yes
Fragments for identification		Additional dimension/techniques	-

Fragment name
Neutral loss of H2O
Neutral loss of H2O and C23H37NO18
Sphingosine -H2O fragment
Sphingosine -2H2O fragment

Isotope correction at MS1	No	Lipid Identification Software	MS-DIAL
Isotope correction at MS2	No	Data manipulation	Smoothing, Centroiding
MS1 verified by standard	No	Nomenclature for intact lipid molecule	Yes
MS2 verified by standard	No	Nomenclature for fragment ions	No
Background check at MS1	Yes	Further identification remarks	-

69) GM3[M+NH4]+ / Lipid quantification

Quantitative	Yes	Limit of quantification	No
MS Level for quantification	MS1	Normalization to reference	No
Internal lipid standard(s) MS1		Lipid Quantification Software	MS-DIAL

Internal standard	Endogenous subclass
LPC 18:1(d7)	GM3 subclass

Type of quantification	Internal standard amount	Batch correction	No
Response correction	No	Further quantification remarks	-
Type I isotope correction	No		

70) Ganglioside GQ1b (GQ1b)[M-2H]2- / Lipid identification

Lipid class	Ganglioside GQ1b (GQ1b)	Background check at MS2	No
Derivatization	-	Did you presume assumptions for identification?	No
MS Level for identification	MS1, MS2	Check isomer overlap	No
Identification level	Species level	RT verified by standard	Yes
Polarity mode	Negative	Separation of isobaric/isomeric interferece confirmed	Yes
Type of negative (precursor)ion	[M-2H]2-	Model for separation prediction	Yes
Fragments for identification		Additional dimension/techniques	-

Fragment name
Characteristic fragment (C11H16NO8-)
Characteristic fragment (C22H33N2O16-)

Isotope correction at MS1	No	Lipid Identification Software	MS-DIAL
Isotope correction at MS2	No	Data manipulation	Smoothing, Centroiding
MS1 verified by standard	No	Nomenclature for intact lipid molecule	Yes
MS2 verified by standard	No	Nomenclature for fragment ions	No
Background check at MS1	Yes	Further identification remarks	-

70) Ganglioside GQ1b (GQ1b)[M-2H]2- / Lipid quantification

Quantitative	Yes	Limit of quantification	No
MS Level for quantification	MS1	Normalization to reference	No
Internal lipid standard(s) MS1		Lipid Quantification Software	MS-DIAL

Internal standard	Endogenous subclass
LPC 18:1(d7)	GQ1b subclass

Type of quantification	Internal standard amount	Batch correction	No
Response correction	No	Further quantification remarks	-
Type I isotope correction	No		

71) Ganglioside GT1b (GT1b)[M-2H]2- / Lipid identification

Lipid class	Ganglioside GT1b (GT1b)	Background check at MS2	No
Derivatization	-	Did you presume assumptions for identification?	No
MS Level for identification	MS1, MS2	Check isomer overlap	No
Identification level	Species level	RT verified by standard	Yes
Polarity mode	Negative	Separation of isobaric/isomeric interferece confirmed	Yes
Type of negative (precursor)ion	[M-2H]2-	Model for separation prediction	Yes
Fragments for identification		Additional dimension/techniques	-

Fragment name
Characteristic fragment (C11H16NO8-)
Characteristic fragment (C22H33N2O16-)

Isotope correction at MS1	No	Lipid Identification Software	MS-DIAL
Isotope correction at MS2	No	Data manipulation	Smoothing, Centroiding
MS1 verified by standard	No	Nomenclature for intact lipid molecule	Yes
MS2 verified by standard	No	Nomenclature for fragment ions	No
Background check at MS1	Yes	Further identification remarks	-

71) Ganglioside GT1b (GT1b)[M-2H]2- / Lipid quantification

Quantitative	Yes	Limit of quantification	No
MS Level for quantification	MS1	Normalization to reference	No
Internal lipid standard(s) MS1		Lipid Quantification Software	MS-DIAL

Internal standard	Endogenous subclass
LPC 18:1(d7)	GT1b subclass

Type of quantification	Internal standard amount	Batch correction	No
Response correction	No	Further quantification remarks	-
Type I isotope correction	No		

72) Glycerophospho N-acyl ethanolamine (GPNAE)[M-H]- / Lipid identification

Lipid class	Glycerophospho N-acyl ethanolamine (GPNAE)	Background check at MS2	No
Derivatization	-	Did you presume assumptions for identification?	No
MS Level for identification	MS1, MS2	Check isomer overlap	No
Identification level	Molecular species level	RT verified by standard	Yes
Polarity mode	Negative	Separation of isobaric/isomeric interferece confirmed	Yes
Type of negative (precursor)ion	[M-H]-	Model for separation prediction	Yes
Fragments for identification		Additional dimension/techniques	-

Fragment name
Characteristic fragment (C3H8PO6-)
Phosphite

Isotope correction at MS1	No	Lipid Identification Software	MS-DIAL
Isotope correction at MS2	No	Data manipulation	Smoothing, Centroiding
MS1 verified by standard	No	Nomenclature for intact lipid molecule	Yes
MS2 verified by standard	No	Nomenclature for fragment ions	No
Background check at MS1	Yes	Further identification remarks	-

72) Glycerophospho N-acyl ethanolamine (GPNAE)[M-H]- / Lipid quantification

Quantitative	Yes	Limit of quantification	No
MS Level for quantification	MS1	Normalization to reference	No
Internal lipid standard(s) MS1		Lipid Quantification Software	MS-DIAL

Internal standard	Endogenous subclass
LPC 18:1(d7)	GPNAE subclass

Type of quantification	Internal standard amount	Batch correction	No
Response correction	No	Further quantification remarks	-
Type I isotope correction	No		

73) Hemibismonoacylglycerophosphate (HBMP)[M+NH4]+ / Lipid identification

Lipid class	Hemibismonoacylglycerophosphate (HBMP)	Background check at MS2	No
Derivatization	-	Did you presume assumptions for identification?	No
MS Level for identification	MS1, MS2	Check isomer overlap	No
Identification level	Molecular species level	RT verified by standard	Yes
Polarity mode	Positive	Separation of isobaric/isomeric interferece confirmed	Yes
Type of positive (precursor)ion	[M+NH4]+	Model for separation prediction	Yes
Fragments for identification		Additional dimension/techniques	-
Isotope correction at MS1	No	Lipid Identification Software	MS-DIAL
Isotope correction at MS2	No	Data manipulation	Smoothing, Centroiding
MS1 verified by standard	No	Nomenclature for intact lipid molecule	Yes
MS2 verified by standard	No	Nomenclature for fragment ions	No
Background check at MS1	Yes	Further identification remarks	-

Fragment name
Dehydro-monoacyl glycerols
Dehydro-diacyl glycerol

73) Hemibismonoacylglycerophosphate (HBMP)[M+NH4]+ / Lipid quantification

Quantitative	Yes	Limit of quantification	No
MS Level for quantification	MS1	Normalization to reference	No
Internal lipid standard(s) MS1		Lipid Quantification Software	MS-DIAL
Type of quantification	Internal standard amount	Batch correction	No
Response correction	No	Further quantification remarks	-
Type I isotope correction	No		

Internal standard	Endogenous subclass
PG 15:0_18:1(d7)	HBMP subclass

74) Hexosylceramide alpha-hydroxy fatty acid-phytospingosine (HexCer_AP)[M+H]+ / Lipid identification

Lipid class	Hexosylceramide alpha-hydroxy fatty acid-phytospingosine (HexCer_AP)	Background check at MS2	No
Derivatization	-	Did you presume assumptions for identification?	No
MS Level for identification	MS1, MS2	Check isomer overlap	No
Identification level	Molecular species level	RT verified by standard	Yes
Polarity mode	Positive	Separation of isobaric/isomeric interferece confirmed	Yes
Type of positive (precursor)ion	[M+H]+	Model for separation prediction	Yes
Fragments for identification		Additional dimension/techniques	-

Fragment name
Neutral loss of hexose
Neutral loss of hexose and H2O
Phytosphingosine
Phytosphingosine -H2O fragment
Phytosphingosine -3H2O fragment

Isotope correction at MS1	No	Lipid Identification Software	MS-DIAL
Isotope correction at MS2	No	Data manipulation	Smoothing, Centroiding
MS1 verified by standard	No	Nomenclature for intact lipid molecule	Yes
MS2 verified by standard	No	Nomenclature for fragment ions	No
Background check at MS1	Yes	Further identification remarks	-

74) Hexosylceramide alpha-hydroxy fatty acid-phytospingosine (HexCer_AP)[M+H]+ / Lipid quantification

Quantitative	Yes	Limit of quantification	No
MS Level for quantification	MS1	Normalization to reference	No
Internal lipid standard(s) MS1		Lipid Quantification Software	MS-DIAL

Internal standard	Endogenous subclass
Cer 18:1;2O/15:0(d7)	HexCer_AP subclass

Type of quantification	Internal standard amount	Batch correction	No
Response correction	No	Further quantification remarks	-
Type I isotope correction	No		

75) Hexosylceramide Esterified omega-hydroxy fatty acid-sphingosine (HexCer_EOS)[M+H]+ / Lipid identification

Lipid class	Hexosylceramide Esterified omega-hydroxy fatty acid-sphingosine (HexCer_EOS)	Background check at MS2	No
Derivatization	-	Did you presume assumptions for identification?	No
MS Level for identification	MS1, MS2	Check isomer overlap	No
Identification level	Molecular species level	RT verified by standard	Yes
Polarity mode	Positive	Separation of isobaric/isomeric interferece confirmed	Yes
Type of positive (precursor)ion	[M+H]+	Model for separation prediction	Yes
Fragments for identification		Additional dimension/techniques	-

Fragment name
Neutral loss of hexose
Neutral loss of hexose and H2O
Sphingosine -H2O fragment
Sphingosine -2H2O fragment
Sphingosine -CH4O2 fragment

Isotope correction at MS1	No	Lipid Identification Software	MS-DIAL
Isotope correction at MS2	No	Data manipulation	Smoothing, Centroiding
MS1 verified by standard	No	Nomenclature for intact lipid molecule	Yes
MS2 verified by standard	No	Nomenclature for fragment ions	No
Background check at MS1	Yes	Further identification remarks	-

75) Hexosylceramide Esterified omega-hydroxy fatty acid-sphingosine (HexCer_EOS)[M+H]+ / Lipid quantification

Quantitative	Yes	Limit of quantification	No
MS Level for quantification	MS1	Normalization to reference	No
Internal lipid standard(s) MS1		Lipid Quantification Software	MS-DIAL

Internal standard	Endogenous subclass
Cer 18:1;2O/15:0(d7)	HexCer_EOS subclass

Type of quantification	Internal standard amount	Batch correction	No
Response correction	No	Further quantification remarks	-
Type I isotope correction	No		

76) Hexosylceramide hydroxyfatty acid-dihydrosphingosine (HexCer_HDS)[M+H]+ / Lipid identification

Lipid class	Hexosylceramide hydroxyfatty acid-dihydrosphingosine (HexCer_HDS)	Background check at MS2	No
Derivatization	-	Did you presume assumptions for identification?	No
MS Level for identification	MS1, MS2	Check isomer overlap	No
Identification level	Molecular species level	RT verified by standard	Yes
Polarity mode	Positive	Separation of isobaric/isomeric interferece confirmed	Yes
Type of positive (precursor)ion	[M+H]+	Model for separation prediction	Yes
Fragments for identification		Additional dimension/techniques	-

Fragment name
Neutral loss of hexose
Neutral loss of hexose and H2O
Sphinganine -H2O fragment
Sphinganine -2H2O fragment
Sphinganine -CH4O2 fragment

Isotope correction at MS1	No	Lipid Identification Software	MS-DIAL
Isotope correction at MS2	No	Data manipulation	Smoothing, Centroiding
MS1 verified by standard	No	Nomenclature for intact lipid molecule	Yes
MS2 verified by standard	No	Nomenclature for fragment ions	No
Background check at MS1	Yes	Further identification remarks	-

76) Hexosylceramide hydroxyfatty acid-dihydrosphingosine (HexCer_HDS)[M+H]+ / Lipid quantification

Quantitative	Yes	Limit of quantification	No
MS Level for quantification	MS1	Normalization to reference	No
Internal lipid standard(s) MS1		Lipid Quantification Software	MS-DIAL

Internal standard	Endogenous subclass
Cer 18:1;2O/15:0(d7)	HexCer_HDS subclass

Type of quantification	Internal standard amount	Batch correction	No
Response correction	No	Further quantification remarks	-
Type I isotope correction	No		

77) Hexosylceramide hydroxyfatty acid-sphingosine (HexCer_HS)[M+H]+ / Lipid identification

Lipid class	Hexosylceramide hydroxyfatty acid-sphingosine (HexCer_HS)	Background check at MS2	No
Derivatization	-	Did you presume assumptions for identification?	No
MS Level for identification	MS1, MS2	Check isomer overlap	No
Identification level	Molecular species level	RT verified by standard	Yes
Polarity mode	Positive	Separation of isobaric/isomeric interferece confirmed	Yes
Type of positive (precursor)ion	[M+H]+	Model for separation prediction	Yes
Fragments for identification		Additional dimension/techniques	-

Fragment name
Neutral loss of hexose
Neutral loss of hexose and H2O
Sphingosine -H2O fragment
Sphingosine -2H2O fragment
Sphingosine -CH4O2 fragment

Isotope correction at MS1	No	Lipid Identification Software	MS-DIAL
Isotope correction at MS2	No	Data manipulation	Smoothing, Centroiding
MS1 verified by standard	No	Nomenclature for intact lipid molecule	Yes
MS2 verified by standard	No	Nomenclature for fragment ions	No
Background check at MS1	Yes	Further identification remarks	-

77) Hexosylceramide hydroxyfatty acid-sphingosine (HexCer_HS)[M+H]+ / Lipid quantification

Quantitative	Yes	Limit of quantification	No
MS Level for quantification	MS1	Normalization to reference	No
Internal lipid standard(s) MS1		Lipid Quantification Software	MS-DIAL

Internal standard	Endogenous subclass
Cer 18:1;2O/15:0(d7)	HexCer_HS subclass

Type of quantification	Internal standard amount	Batch correction	No
Response correction	No	Further quantification remarks	-
Type I isotope correction	No		

78) Hexosylceramide non-hydroxyfatty acid-dihydrosphingosine (HexCer_NDS)[M+H]+ / Lipid identification

Lipid class	Hexosylceramide non-hydroxyfatty acid-dihydrosphingosine (HexCer_NDS)	Background check at MS2	No
Derivatization	-	Did you presume assumptions for identification?	No
MS Level for identification	MS1, MS2	Check isomer overlap	No
Identification level	Molecular species level	RT verified by standard	Yes
Polarity mode	Positive	Separation of isobaric/isomeric interferece confirmed	Yes
Type of positive (precursor)ion	[M+H]+	Model for separation prediction	Yes
Fragments for identification		Additional dimension/techniques	-

Fragment name
Neutral loss of hexose and H2O
Sphinganine -H2O fragment
Sphinganine -2H2O fragment

Isotope correction at MS1	No	Lipid Identification Software	MS-DIAL
Isotope correction at MS2	No	Data manipulation	Smoothing, Centroiding
MS1 verified by standard	No	Nomenclature for intact lipid molecule	Yes
MS2 verified by standard	No	Nomenclature for fragment ions	No
Background check at MS1	Yes	Further identification remarks	-

78) Hexosylceramide non-hydroxyfatty acid-dihydrosphingosine (HexCer_NDS)[M+H]+ / Lipid quantification

Quantitative	Yes	Limit of quantification	No
MS Level for quantification	MS1	Normalization to reference	No
Internal lipid standard(s) MS1		Lipid Quantification Software	MS-DIAL

Internal standard	Endogenous subclass
Cer 18:1;2O/15:0(d7)	HexCer_NDS subclass

Type of quantification	Internal standard amount	Batch correction	No
Response correction	No	Further quantification remarks	-
Type I isotope correction	No		

79) Hexosylceramide non-hydroxyfatty acid-sphingosine (HexCer_NS)[M+H]+ / Lipid identification

Lipid class	Hexosylceramide non-hydroxyfatty acid-sphingosine (HexCer_NS)	Background check at MS2	No
Derivatization	-	Did you presume assumptions for identification?	No
MS Level for identification	MS1, MS2	Check isomer overlap	No
Identification level	Molecular species level	RT verified by standard	Yes
Polarity mode	Positive	Separation of isobaric/isomeric interferece confirmed	Yes
Type of positive (precursor)ion	[M+H]+	Model for separation prediction	Yes
Fragments for identification		Additional dimension/techniques	-

Fragment name
Neutral loss of H2O
Neutral loss of hexose and H2O
Sphingosine -H2O fragment
Sphingosine -2H2O fragment
Sphingosine -CH4O2 fragment

Isotope correction at MS1	No	Lipid Identification Software	MS-DIAL
Isotope correction at MS2	No	Data manipulation	Smoothing, Centroiding
MS1 verified by standard	No	Nomenclature for intact lipid molecule	Yes
MS2 verified by standard	No	Nomenclature for fragment ions	No
Background check at MS1	Yes	Further identification remarks	-

79) Hexosylceramide non-hydroxyfatty acid-sphingosine (HexCer_NS)[M+H]+ / Lipid quantification

Quantitative	Yes	Limit of quantification	No
MS Level for quantification	MS1	Normalization to reference	No
Internal lipid standard(s) MS1		Lipid Quantification Software	MS-DIAL

Internal standard	Endogenous subclass
Cer 18:1;2O/15:0(d7)	HexCer_NS subclass

Type of quantification	Internal standard amount	Batch correction	No
Response correction	No	Further quantification remarks	-
Type I isotope correction	No		

80) Lysocardiolipin (MLCL)[M-H]- / Lipid identification

Lipid class	Lysocardiolipin (MLCL)	Background check at MS2	No
Derivatization	-	Did you presume assumptions for identification?	No
MS Level for identification	MS1, MS2	Check isomer overlap	No
Identification level	Molecular species level	RT verified by standard	Yes
Polarity mode	Negative	Separation of isobaric/isomeric interferece confirmed	Yes
Type of negative (precursor)ion	[M-H]-	Model for separation prediction	Yes
Fragments for identification		Additional dimension/techniques	-

Fragment name
Phosphoglycerol -H2O fragment
Phosphatidic acid
Lysophosphatidic acid
Fatty acid fragment

Isotope correction at MS1	No	Lipid Identification Software	MS-DIAL
Isotope correction at MS2	No	Data manipulation	Smoothing, Centroiding
MS1 verified by standard	No	Nomenclature for intact lipid molecule	Yes
MS2 verified by standard	No	Nomenclature for fragment ions	No
Background check at MS1	Yes	Further identification remarks	-

80) Lysocardiolipin (MLCL)[M-H]- / Lipid quantification

Quantitative	Yes	Limit of quantification	No
MS Level for quantification	MS1	Normalization to reference	No
Internal lipid standard(s) MS1		Lipid Quantification Software	MS-DIAL

Internal standard	Endogenous subclass
PG 15:0_18:1(d7)	MLCL subclass

Type of quantification	Internal standard amount	Batch correction	No
Response correction	No	Further quantification remarks	-
Type I isotope correction	No		

81) Lysodiacylglyceryl-3-O-carboxyhydroxymethylcholine (LDGCC)[M+H]+ / Lipid identification

Lipid class	Lysodiacylglyceryl-3-O-carboxyhydroxymethylcholine (LDGCC)	Background check at MS2	No
Derivatization	-	Did you presume assumptions for identification?	No
MS Level for identification	MS1, MS2	Check isomer overlap	No
Identification level	Molecular species level	RT verified by standard	Yes
Polarity mode	Positive	Separation of isobaric/isomeric interferece confirmed	Yes
Type of positive (precursor)ion	[M+H]+	Model for separation prediction	Yes
Fragments for identification		Additional dimension/techniques	-
Isotope correction at MS1	No	Lipid Identification Software	MS-DIAL
Isotope correction at MS2	No	Data manipulation	Smoothing, Centroiding
MS1 verified by standard	No	Nomenclature for intact lipid molecule	Yes
MS2 verified by standard	No	Nomenclature for fragment ions	No
Background check at MS1	Yes	Further identification remarks	-

Fragment name
Characteristic fragment (C6H14NO2+)

81) Lysodiacylglyceryl-3-O-carboxyhydroxymethylcholine (LDGCC)[M+H]+ / Lipid quantification

Quantitative	Yes	Limit of quantification	No
MS Level for quantification	MS1	Normalization to reference	No
Internal lipid standard(s) MS1		Lipid Quantification Software	MS-DIAL
Type of quantification	Internal standard amount	Batch correction	No
Response correction	No	Further quantification remarks	-
Type I isotope correction	No		

Internal standard	Endogenous subclass
LPC 18:1(d7)	LDGCC subclass

82) Lysogiacylglyceryl trimethylhomoserine (LDGTS)[M+H]+ / Lipid identification

Lipid class	Lysogiacylglyceryl trimethylhomoserine (LDGTS)	Background check at MS2	No
Derivatization	-	Did you presume assumptions for identification?	No
MS Level for identification	MS1, MS2	Check isomer overlap	No
Identification level	Molecular species level	RT verified by standard	Yes
Polarity mode	Positive	Separation of isobaric/isomeric interferece confirmed	Yes
Type of positive (precursor)ion	[M+H]+	Model for separation prediction	Yes
Fragments for identification		Additional dimension/techniques	-

Fragment name
Characteristic fragments (C7H14NO2+)
Characteristic fragments (C10H22NO5+)

Isotope correction at MS1	No	Lipid Identification Software	MS-DIAL
Isotope correction at MS2	No	Data manipulation	Smoothing, Centroiding
MS1 verified by standard	No	Nomenclature for intact lipid molecule	Yes
MS2 verified by standard	No	Nomenclature for fragment ions	No
Background check at MS1	Yes	Further identification remarks	-

82) Lysogiacylglyceryl trimethylhomoserine (LDGTS)[M+H]+ / Lipid quantification

Quantitative	Yes	Limit of quantification	No
MS Level for quantification	MS1	Normalization to reference	No
Internal lipid standard(s) MS1		Lipid Quantification Software	MS-DIAL

Internal standard	Endogenous subclass
LPC 18:1(d7)	LDGTS subclass

Type of quantification	Internal standard amount	Batch correction	No
Response correction	No	Further quantification remarks	-
Type I isotope correction	No		

83) LPC[M+H]+ / Lipid identification

Lipid class	LPC	Background check at MS2	No
Derivatization	-	Did you presume assumptions for identification?	No
MS Level for identification	MS1, MS2	Check isomer overlap	No
Identification level	Molecular species level	RT verified by standard	Yes
Polarity mode	Positive	Separation of isobaric/isomeric interferece confirmed	Yes
Type of positive (precursor)ion	[M+H]+	Model for separation prediction	Yes
Fragments for identification		Additional dimension/techniques	-

Fragment name
Characteristic fragment (C5H15NO4P+)

Isotope correction at MS1	No	Lipid Identification Software	MS-DIAL
Isotope correction at MS2	No	Data manipulation	Smoothing, Centroiding
MS1 verified by standard	Yes	Nomenclature for intact lipid molecule	Yes
MS2 verified by standard	Yes	Nomenclature for fragment ions	No
Background check at MS1	Yes	Further identification remarks	-

83) LPC[M+H]+ / Lipid quantification

Quantitative	Yes	Limit of quantification	No
MS Level for quantification	MS1	Normalization to reference	No
Internal lipid standard(s) MS1		Lipid Quantification Software	MS-DIAL

Internal standard	Endogenous subclass
LPC 18:1(d7)	LPC subclass

Type of quantification	Internal standard amount	Batch correction	No
Response correction	No	Further quantification remarks	-
Type I isotope correction	No		

84) LPA[M-H]- / Lipid identification

Lipid class	LPA	Background check at MS2	No
Derivatization	-	Did you presume assumptions for identification?	No
MS Level for identification	MS1, MS2	Check isomer overlap	No
Identification level	Molecular species level	RT verified by standard	Yes
Polarity mode	Negative	Separation of isobaric/isomeric interferece confirmed	Yes
Type of negative (precursor)ion	[M-H]-	Model for separation prediction	Yes
Fragments for identification		Additional dimension/techniques	-

Fragment name
Phosphoglycerol -H2O fragment

Isotope correction at MS1	No	Lipid Identification Software	MS-DIAL
Isotope correction at MS2	No	Data manipulation	Smoothing, Centroiding
MS1 verified by standard	No	Nomenclature for intact lipid molecule	Yes
MS2 verified by standard	No	Nomenclature for fragment ions	No
Background check at MS1	Yes	Further identification remarks	-

84) LPA[M-H]- / Lipid quantification

Quantitative	Yes	Limit of quantification	No
MS Level for quantification	MS1	Normalization to reference	No
Internal lipid standard(s) MS1		Lipid Quantification Software	MS-DIAL

Internal standard	Endogenous subclass
LPC 18:1(d7)	LPA subclass

Type of quantification	Internal standard amount	Batch correction	No
Response correction	No	Further quantification remarks	-
Type I isotope correction	No		

85) LPE[M+H]+ / Lipid identification

Lipid class	LPE	Background check at MS2	No
Derivatization	-	Did you presume assumptions for identification?	No
MS Level for identification	MS1, MS2	Check isomer overlap	No
Identification level	Molecular species level	RT verified by standard	Yes
Polarity mode	Positive	Separation of isobaric/isomeric interferece confirmed	Yes
Type of positive (precursor)ion	[M+H]+	Model for separation prediction	Yes
Fragments for identification		Additional dimension/techniques	-

Fragment name
Neutral loss of C2H8NO4P

Isotope correction at MS1	No	Lipid Identification Software	MS-DIAL
Isotope correction at MS2	No	Data manipulation	Smoothing, Centroiding
MS1 verified by standard	Yes	Nomenclature for intact lipid molecule	Yes
MS2 verified by standard	Yes	Nomenclature for fragment ions	No
Background check at MS1	Yes	Further identification remarks	-

85) LPE[M+H]+ / Lipid quantification

Quantitative	Yes	Limit of quantification	No
MS Level for quantification	MS1	Normalization to reference	No
Internal lipid standard(s) MS1		Lipid Quantification Software	MS-DIAL

Internal standard	Endogenous subclass
LPE 18:1(d7)	LPE subclass

Type of quantification	Internal standard amount	Batch correction	No
Response correction	No	Further quantification remarks	-
Type I isotope correction	No		

86) LPG[M-H]- / Lipid identification

Lipid class	LPG	Background check at MS2	No
Derivatization	-	Did you presume assumptions for identification?	No
MS Level for identification	MS1, MS2	Check isomer overlap	No
Identification level	Molecular species level	RT verified by standard	Yes
Polarity mode	Negative	Separation of isobaric/isomeric interferece confirmed	Yes
Type of negative (precursor)ion	[M-H]-	Model for separation prediction	Yes
Fragments for identification		Additional dimension/techniques	-

Fragment name
Phosphoglycerol -H2O fragment

Isotope correction at MS1	No	Lipid Identification Software	MS-DIAL
Isotope correction at MS2	No	Data manipulation	Smoothing, Centroiding
MS1 verified by standard	No	Nomenclature for intact lipid molecule	Yes
MS2 verified by standard	No	Nomenclature for fragment ions	No
Background check at MS1	Yes	Further identification remarks	-

86) LPG[M-H]- / Lipid quantification

Quantitative	Yes	Limit of quantification	No
MS Level for quantification	MS1	Normalization to reference	No
Internal lipid standard(s) MS1		Lipid Quantification Software	MS-DIAL

Internal standard	Endogenous subclass
PG 15:0_18:1(d7)	LPG subclass

Type of quantification	Internal standard amount	Batch correction	No
Response correction	No	Further quantification remarks	-
Type I isotope correction	No		

87) LPI[M-H]- / Lipid identification

Lipid class	LPI	Background check at MS2	No
Derivatization	-	Did you presume assumptions for identification?	No
MS Level for identification	MS1, MS2	Check isomer overlap	No
Identification level	Molecular species level	RT verified by standard	Yes
Polarity mode	Negative	Separation of isobaric/isomeric interferece confirmed	Yes
Type of negative (precursor)ion	[M-H]-	Model for separation prediction	Yes
Fragments for identification		Additional dimension/techniques	-

Fragment name
Phosphoinositol -H2O fragment
Characteristic fragment (C9H16O10P-)
Fatty acid fragment

Isotope correction at MS1	No	Lipid Identification Software	MS-DIAL
Isotope correction at MS2	No	Data manipulation	Smoothing, Centroiding
MS1 verified by standard	No	Nomenclature for intact lipid molecule	Yes
MS2 verified by standard	No	Nomenclature for fragment ions	No
Background check at MS1	Yes	Further identification remarks	-

87) LPI[M-H]- / Lipid quantification

Quantitative	Yes	Limit of quantification	No
MS Level for quantification	MS1	Normalization to reference	No
Internal lipid standard(s) MS1		Lipid Quantification Software	MS-DIAL

Internal standard	Endogenous subclass
PI 15:0_18:1(d7)	LPI subclass

Type of quantification	Internal standard amount	Batch correction	No
Response correction	No	Further quantification remarks	-
Type I isotope correction	No		

88) LPS[M-H]- / Lipid identification

Lipid class	LPS	Background check at MS2	No
Derivatization	-	Did you presume assumptions for identification?	No
MS Level for identification	MS1, MS2	Check isomer overlap	No
Identification level	Molecular species level	RT verified by standard	Yes
Polarity mode	Negative	Separation of isobaric/isomeric interferece confirmed	Yes
Type of negative (precursor)ion	[M-H]-	Model for separation prediction	Yes
Fragments for identification		Additional dimension/techniques	-

Fragment name
Neutral loss of C3H6NO2
Phosphoglycerol -H2O fragment

Isotope correction at MS1	No	Lipid Identification Software	MS-DIAL
Isotope correction at MS2	No	Data manipulation	Smoothing, Centroiding
MS1 verified by standard	No	Nomenclature for intact lipid molecule	Yes
MS2 verified by standard	No	Nomenclature for fragment ions	No
Background check at MS1	Yes	Further identification remarks	-

88) LPS[M-H]- / Lipid quantification

Quantitative	Yes	Limit of quantification	No
MS Level for quantification	MS1	Normalization to reference	No
Internal lipid standard(s) MS1		Lipid Quantification Software	MS-DIAL

Internal standard	Endogenous subclass
PS 15:0_18:1(d7)	LPS subclass

Type of quantification	Internal standard amount	Batch correction	No
Response correction	No	Further quantification remarks	-
Type I isotope correction	No		

89) MIPC[M-H]- / Lipid identification

Lipid class	MIPC	Background check at MS2	No
Derivatization	-	Did you presume assumptions for identification?	No
MS Level for identification	MS1, MS2	Check isomer overlap	No
Identification level	Molecular species level	RT verified by standard	Yes
Polarity mode	Negative	Separation of isobaric/isomeric interferece confirmed	Yes
Type of negative (precursor)ion	[M-H]-	Model for separation prediction	Yes
Fragments for identification		Additional dimension/techniques	-

Fragment name
Characteristic fragment (C12H22O14P-)
Phytosphingosine -C2H7NO fragment

Isotope correction at MS1	No	Lipid Identification Software	MS-DIAL
Isotope correction at MS2	No	Data manipulation	Smoothing, Centroiding
MS1 verified by standard	No	Nomenclature for intact lipid molecule	Yes
MS2 verified by standard	No	Nomenclature for fragment ions	No
Background check at MS1	Yes	Further identification remarks	-

89) MIPC[M-H]- / Lipid quantification

Quantitative	Yes	Limit of quantification	No
MS Level for quantification	MS1	Normalization to reference	No
Internal lipid standard(s) MS1		Lipid Quantification Software	MS-DIAL

Internal standard	Endogenous subclass
PI 15:0_18:1(d7)	MIPC subclass

Type of quantification	Internal standard amount	Batch correction	No
Response correction	No	Further quantification remarks	-
Type I isotope correction	No		

90) MG[M+NH4]+ / Lipid identification

Lipid class	MG	Background check at MS2	No
Derivatization	-	Did you presume assumptions for identification?	No
MS Level for identification	MS1, MS2	Check isomer overlap	No
Identification level	Molecular species level	RT verified by standard	Yes
Polarity mode	Positive	Separation of isobaric/isomeric interferece confirmed	Yes
Type of positive (precursor)ion	[M+NH4]+	Model for separation prediction	Yes
Fragments for identification		Additional dimension/techniques	-

Fragment name
Neutral loss of H2O

Isotope correction at MS1	No	Lipid Identification Software	MS-DIAL
Isotope correction at MS2	No	Data manipulation	Smoothing, Centroiding
MS1 verified by standard	Yes	Nomenclature for intact lipid molecule	Yes
MS2 verified by standard	Yes	Nomenclature for fragment ions	No
Background check at MS1	Yes	Further identification remarks	-

90) MG[M+NH4]+ / Lipid quantification

Quantitative	Yes	Limit of quantification	No
MS Level for quantification	MS1	Normalization to reference	No
Internal lipid standard(s) MS1		Lipid Quantification Software	MS-DIAL

Internal standard	Endogenous subclass
MG 18:1(d7)	MG subclass

Type of quantification	Internal standard amount	Batch correction	No
Response correction	No	Further quantification remarks	-
Type I isotope correction	No		

91) MGDG[M+CH3COO]- / Lipid identification

Lipid class	MGDG	Background check at MS2	No
Derivatization	-	Did you presume assumptions for identification?	No
MS Level for identification	MS1, MS2	Check isomer overlap	No
Identification level	Molecular species level	RT verified by standard	Yes
Polarity mode	Negative	Separation of isobaric/isomeric interferece confirmed	Yes
Type of negative (precursor)ion	[M+CH3COO]-	Model for separation prediction	Yes
Fragments for identification		Additional dimension/techniques	-

Fragment name
Fatty acid fragment

Isotope correction at MS1	No	Lipid Identification Software	MS-DIAL
Isotope correction at MS2	No	Data manipulation	Smoothing, Centroiding
MS1 verified by standard	No	Nomenclature for intact lipid molecule	Yes
MS2 verified by standard	No	Nomenclature for fragment ions	No
Background check at MS1	Yes	Further identification remarks	-

91) MGDG[M+CH3COO]- / Lipid quantification

Quantitative	Yes	Limit of quantification	No
MS Level for quantification	MS1	Normalization to reference	No
Internal lipid standard(s) MS1		Lipid Quantification Software	MS-DIAL

Internal standard	Endogenous subclass
LPC 18:1(d7)	MGDG subclass

Type of quantification	Internal standard amount	Batch correction	No
Response correction	No	Further quantification remarks	-
Type I isotope correction	No		

92) Monogalactosylmonoacylglycerol (MGMG)[M+CH3COO]- / Lipid identification

Lipid class	Monogalactosylmonoacylglyc-erol (MGMG)	Background check at MS2	No
Derivatization	-	Did you presume assumptions for identification?	No
MS Level for identification	MS1, MS2	Check isomer overlap	No
Identification level	Molecular species level	RT verified by standard	Yes
Polarity mode	Negative	Separation of isobaric/isomeric interference confirmed	Yes
Type of negative (precursor)ion	[M+CH3COO]-	Model for separation prediction	Yes
Fragments for identification		Additional dimension/techniques	-
Isotope correction at MS1	No	Lipid Identification Software	MS-DIAL
Isotope correction at MS2	No	Data manipulation	Smoothing, Centroiding
MS1 verified by standard	No	Nomenclature for intact lipid molecule	Yes
MS2 verified by standard	No	Nomenclature for fragment ions	No
Background check at MS1	Yes	Further identification remarks	-

Fragment name
Fatty acid fragment

92) Monogalactosylmonoacylglycerol (MGMG)[M+CH3COO]- / Lipid quantification

Quantitative	Yes	Limit of quantification	No
MS Level for quantification	MS1	Normalization to reference	No
Internal lipid standard(s) MS1		Lipid Quantification Software	MS-DIAL
Type of quantification	Internal standard amount	Batch correction	No
Response correction	No	Further quantification remarks	-
Type I isotope correction	No		

Internal standard	Endogenous subclass
LPC 18:1(d7)	MGMG subclass

93) N-acyl ethanolamines (NAE)[M+CH3COO]- / Lipid identification

Lipid class	N-acyl ethanolamines (NAE)	Background check at MS2	No
Derivatization	-	Did you presume assumptions for identification?	No
MS Level for identification	MS1, MS2	Check isomer overlap	No
Identification level	Molecular species level	RT verified by standard	Yes
Polarity mode	Negative	Separation of isobaric/isomeric interferece confirmed	Yes
Type of negative (precursor)ion	[M+CH3COO]-	Model for separation prediction	Yes
Fragments for identification		Additional dimension/techniques	-

Fragment name
Neutral loss of 2H

Isotope correction at MS1	No	Lipid Identification Software	MS-DIAL
Isotope correction at MS2	No	Data manipulation	Smoothing, Centroiding
MS1 verified by standard	No	Nomenclature for intact lipid molecule	Yes
MS2 verified by standard	No	Nomenclature for fragment ions	No
Background check at MS1	Yes	Further identification remarks	-

93) N-acyl ethanolamines (NAE)[M+CH3COO]- / Lipid quantification

Quantitative	Yes	Limit of quantification	No
MS Level for quantification	MS1	Normalization to reference	No
Internal lipid standard(s) MS1		Lipid Quantification Software	MS-DIAL

Internal standard	Endogenous subclass
LPC 18:1(d7)	NAE subclass

Type of quantification	Internal standard amount	Batch correction	No
Response correction	No	Further quantification remarks	-
Type I isotope correction	No		

94) N-acyl glycine (NAGly)[M+NH4]+ / Lipid identification

Lipid class	N-acyl glycine (NAGly)	Background check at MS2	No
Derivatization	-	Did you presume assumptions for identification?	No
MS Level for identification	MS1, MS2	Check isomer overlap	No
Identification level	Molecular species level	RT verified by standard	Yes
Polarity mode	Positive	Separation of isobaric/isomeric interferece confirmed	Yes
Type of positive (precursor)ion	[M+NH4]+	Model for separation prediction	Yes
Fragments for identification		Additional dimension/techniques	-

Fragment name
Glycine
Fatty acyl fragment
Fatty acyl -H2O fragment
Neutral loss of Acyl and H2O

Isotope correction at MS1	No	Lipid Identification Software	MS-DIAL
Isotope correction at MS2	No	Data manipulation	Smoothing, Centroiding
MS1 verified by standard	No	Nomenclature for intact lipid molecule	Yes
MS2 verified by standard	No	Nomenclature for fragment ions	No
Background check at MS1	Yes	Further identification remarks	-

94) N-acyl glycine (NAGly)[M+NH4]+ / Lipid quantification

Quantitative	Yes	Limit of quantification	No
MS Level for quantification	MS1	Normalization to reference	No
Internal lipid standard(s) MS1		Lipid Quantification Software	MS-DIAL

Internal standard	Endogenous subclass
LPC 18:1(d7)	NAGly subclass

Type of quantification	Internal standard amount	Batch correction	No
Response correction	No	Further quantification remarks	-
Type I isotope correction	No		

95) N-acyl glycyl serine (NAGlySer)[M+NH4]+ / Lipid identification

Lipid class	N-acyl glycyl serine (NAGlySer)	Background check at MS2	No
Derivatization	-	Did you presume assumptions for identification?	No
MS Level for identification	MS1, MS2	Check isomer overlap	No
Identification level	Molecular species level	RT verified by standard	Yes
Polarity mode	Positive	Separation of isobaric/isomeric interferece confirmed	Yes
Type of positive (precursor)ion	[M+NH4]+	Model for separation prediction	Yes
Fragments for identification		Additional dimension/techniques	-

Fragment name
Glycylserine
Fatty acyl fragment
Serine
Neutral loss of Acyl and H2O
Acyl Glycine fragment

Isotope correction at MS1	No	Lipid Identification Software	MS-DIAL
Isotope correction at MS2	No	Data manipulation	Smoothing, Centroiding
MS1 verified by standard	No	Nomenclature for intact lipid molecule	Yes
MS2 verified by standard	No	Nomenclature for fragment ions	No
Background check at MS1	Yes	Further identification remarks	-

95) N-acyl glycyl serine (NAGlySer)[M+NH4]+ / Lipid quantification

Quantitative	Yes	Limit of quantification	No
MS Level for quantification	MS1	Normalization to reference	No
Internal lipid standard(s) MS1		Lipid Quantification Software	MS-DIAL

Internal standard	Endogenous subclass
LPC 18:1(d7)	NAGlySer subclass

Type of quantification	Internal standard amount	Batch correction	No
Response correction	No	Further quantification remarks	-
Type I isotope correction	No		

96) N-acyl ornithine (NAOrn)[M+H]+ / Lipid identification

Lipid class	N-acyl ornithine (NAOrn)	Background check at MS2	No
Derivatization	-	Did you presume assumptions for identification?	No
MS Level for identification	MS1, MS2	Check isomer overlap	No
Identification level	Molecular species level	RT verified by standard	Yes
Polarity mode	Positive	Separation of isobaric/isomeric interferece confirmed	Yes
Type of positive (precursor)ion	[M+H]+	Model for separation prediction	Yes
Fragments for identification		Additional dimension/techniques	-

Fragment name
Ornithine -H2O
Characteristic fragment (C4H8N+)
Neutral loss of Acyl and H2O
Neutral loss of Acyl and 2H2O
Fatty acyl -H2O fragment

Isotope correction at MS1	No	Lipid Identification Software	MS-DIAL
Isotope correction at MS2	No	Data manipulation	Smoothing, Centroiding
MS1 verified by standard	No	Nomenclature for intact lipid molecule	Yes
MS2 verified by standard	No	Nomenclature for fragment ions	No
Background check at MS1	Yes	Further identification remarks	-

96) N-acyl ornithine (NAOrn)[M+H]+ / Lipid quantification

Quantitative	Yes	Limit of quantification	No
MS Level for quantification	MS1	Normalization to reference	No
Internal lipid standard(s) MS1		Lipid Quantification Software	MS-DIAL

Internal standard	Endogenous subclass
LPC 18:1(d7)	NAOrn subclass

Type of quantification	Internal standard amount	Batch correction	No
Response correction	No	Further quantification remarks	-
Type I isotope correction	No		

97) N-acyl-lysophosphatidylethanolamine (LNAPE)[M-H]- / Lipid identification

Lipid class	N-acyl-lysophosphatidylethanolamine (LNAPE)	Background check at MS2	No
Derivatization	-	Did you presume assumptions for identification?	No
MS Level for identification	MS1, MS2	Check isomer overlap	No
Identification level	Molecular species level	RT verified by standard	Yes
Polarity mode	Negative	Separation of isobaric/isomeric interferece confirmed	Yes
Type of negative (precursor)ion	[M-H]-	Model for separation prediction	Yes
Fragments for identification		Additional dimension/techniques	-

Fragment name
Phosphoglycerol -H2O fragment
Fatty acid fragment
Neutral loss of Acyl
Neutral loss of Acyl and H2O

Isotope correction at MS1	No	Lipid Identification Software	MS-DIAL
Isotope correction at MS2	No	Data manipulation	Smoothing, Centroiding
MS1 verified by standard	No	Nomenclature for intact lipid molecule	Yes
MS2 verified by standard	No	Nomenclature for fragment ions	No
Background check at MS1	Yes	Further identification remarks	-

97) N-acyl-lysophosphatidylethanolamine (LNAPE)[M-H]- / Lipid quantification

Quantitative	Yes	Limit of quantification	No
MS Level for quantification	MS1	Normalization to reference	No
Internal lipid standard(s) MS1		Lipid Quantification Software	MS-DIAL

Internal standard	Endogenous subclass
PE 15:0_18:1(d7)	LNAPE subclass

Type of quantification	Internal standard amount	Batch correction	No
Response correction	No	Further quantification remarks	-
Type I isotope correction	No		

98) N-acyl-lysophosphatidylserine (LNAPS)[M-H]- / Lipid identification

Lipid class	N-acyl-lysophosphatidylserine (LNAPS)	Background check at MS2	No
Derivatization	-	Did you presume assumptions for identification?	No
MS Level for identification	MS1, MS2	Check isomer overlap	No
Identification level	Molecular species level	RT verified by standard	Yes
Polarity mode	Negative	Separation of isobaric/isomeric interferece confirmed	Yes
Type of negative (precursor)ion	[M-H]-	Model for separation prediction	Yes
Fragments for identification		Additional dimension/techniques	-

Fragment name
Phosphoglycerol -H2O fragment
Neutral loss of Acyl and C3H5NO2

Isotope correction at MS1	No	Lipid Identification Software	MS-DIAL
Isotope correction at MS2	No	Data manipulation	Smoothing, Centroiding
MS1 verified by standard	No	Nomenclature for intact lipid molecule	Yes
MS2 verified by standard	No	Nomenclature for fragment ions	No
Background check at MS1	Yes	Further identification remarks	-

98) N-acyl-lysophosphatidylserine (LNAPS)[M-H]- / Lipid quantification

Quantitative	Yes	Limit of quantification	No
MS Level for quantification	MS1	Normalization to reference	No
Internal lipid standard(s) MS1		Lipid Quantification Software	MS-DIAL

Internal standard	Endogenous subclass
PS 15:0_18:1(d7)	LNAPS subclass

Type of quantification	Internal standard amount	Batch correction	No
Response correction	No	Further quantification remarks	-
Type I isotope correction	No		

99) DMPE[M-H]- / Lipid identification

Lipid class	DMPE	Background check at MS2	No
Derivatization	-	Did you presume assumptions for identification?	No
MS Level for identification	MS1, MS2	Check isomer overlap	No
Identification level	Molecular species level	RT verified by standard	Yes
Polarity mode	Negative	Separation of isobaric/isomeric interferece confirmed	Yes
Type of negative (precursor)ion	[M-H]-	Model for separation prediction	Yes
Fragments for identification		Additional dimension/techniques	-

Fragment name
Fatty acid fragment

Isotope correction at MS1	No	Lipid Identification Software	MS-DIAL
Isotope correction at MS2	No	Data manipulation	Smoothing, Centroiding
MS1 verified by standard	No	Nomenclature for intact lipid molecule	Yes
MS2 verified by standard	No	Nomenclature for fragment ions	No
Background check at MS1	Yes	Further identification remarks	-

99) DMPE[M-H]- / Lipid quantification

Quantitative	Yes	Limit of quantification	No
MS Level for quantification	MS1	Normalization to reference	No
Internal lipid standard(s) MS1		Lipid Quantification Software	MS-DIAL

Internal standard	Endogenous subclass
PE 15:0_18:1(d7)	DMPE subclass

Type of quantification	Internal standard amount	Batch correction	No
Response correction	No	Further quantification remarks	-
Type I isotope correction	No		

100) NGcGM3 (NGcGM3)[M-H]- / Lipid identification

Lipid class	NGcGM3 (NGcGM3)	Background check at MS2	No
Derivatization	-	Did you presume assumptions for identification?	No
MS Level for identification	MS1, MS2	Check isomer overlap	No
Identification level	Molecular species level	RT verified by standard	Yes
Polarity mode	Negative	Separation of isobaric/isomeric interferece confirmed	Yes
Type of negative (precursor)ion	[M-H]-	Model for separation prediction	Yes
Fragments for identification		Additional dimension/techniques	-

Fragment name
Characteristic fragment (C11H16NO9-)

Isotope correction at MS1	No	Lipid Identification Software	MS-DIAL
Isotope correction at MS2	No	Data manipulation	Smoothing, Centroiding
MS1 verified by standard	No	Nomenclature for intact lipid molecule	Yes
MS2 verified by standard	No	Nomenclature for fragment ions	No
Background check at MS1	Yes	Further identification remarks	-

100) NGcGM3 (NGcGM3)[M-H]- / Lipid quantification

Quantitative	Yes	Limit of quantification	No
MS Level for quantification	MS1	Normalization to reference	No
Internal lipid standard(s) MS1		Lipid Quantification Software	MS-DIAL
Type of quantification	Internal standard amount	Batch correction	No
Response correction	No	Further quantification remarks	-
Type I isotope correction	No		

Internal standard	Endogenous subclass
LPC 18:1(d7)	NGcGM3 subclass

101) MMPE[M-H]- / Lipid identification

Lipid class	MMPE	Background check at MS2	No
Derivatization	-	Did you presume assumptions for identification?	No
MS Level for identification	MS1, MS2	Check isomer overlap	No
Identification level	Molecular species level	RT verified by standard	Yes
Polarity mode	Negative	Separation of isobaric/isomeric interferece confirmed	Yes
Type of negative (precursor)ion	[M-H]-	Model for separation prediction	Yes
Fragments for identification		Additional dimension/techniques	-
Isotope correction at MS1	No	Lipid Identification Software	MS-DIAL
Isotope correction at MS2	No	Data manipulation	Smoothing, Centroiding
MS1 verified by standard	No	Nomenclature for intact lipid molecule	Yes
MS2 verified by standard	No	Nomenclature for fragment ions	No
Background check at MS1	Yes	Further identification remarks	-

Fragment name
Fatty acid fragment

101) MMPE[M-H]- / Lipid quantification

Quantitative	Yes	Limit of quantification	No
MS Level for quantification	MS1	Normalization to reference	No
Internal lipid standard(s) MS1		Lipid Quantification Software	MS-DIAL
Type of quantification	Internal standard amount	Batch correction	No
Response correction	No	Further quantification remarks	-
Type I isotope correction	No		

Internal standard	Endogenous subclass
PE 15:0_18:1(d7)	MMPE subclass

102) Oxidized fatty acid (OxFA)[M-H]- / Lipid identification

Lipid class	Oxidized fatty acid (OxFA)	Background check at MS2	No
Derivatization	-	Did you presume assumptions for identification?	No
MS Level for identification	MS1, MS2	Check isomer overlap	No
Identification level	Species level	RT verified by standard	Yes
Polarity mode	Negative	Separation of isobaric/isomeric interferece confirmed	Yes
Type of negative (precursor)ion	[M-H]-	Model for separation prediction	Yes
Fragments for identification		Additional dimension/techniques	-

Fragment name
Neutral loss of H2O

Isotope correction at MS1	No	Lipid Identification Software	MS-DIAL
Isotope correction at MS2	No	Data manipulation	Smoothing, Centroiding
MS1 verified by standard	No	Nomenclature for intact lipid molecule	Yes
MS2 verified by standard	No	Nomenclature for fragment ions	No
Background check at MS1	Yes	Further identification remarks	-

102) Oxidized fatty acid (OxFA)[M-H]- / Lipid quantification

Quantitative	Yes	Limit of quantification	No
MS Level for quantification	MS1	Normalization to reference	No
Internal lipid standard(s) MS1		Lipid Quantification Software	MS-DIAL

Internal standard	Endogenous subclass
FA 18:0(d3)	OxFA subclass

Type of quantification	Internal standard amount	Batch correction	No
Response correction	No	Further quantification remarks	-
Type I isotope correction	No		

103) Oxidized phosphatidylcholine (OxPC)[M+CH3COO]- / Lipid identification

Lipid class	Oxidized phosphatidylcholine (OxPC)	Background check at MS2	No
Derivatization	-	Did you presume assumptions for identification?	No
MS Level for identification	MS1, MS2	Check isomer overlap	No
Identification level	Molecular species level	RT verified by standard	Yes
Polarity mode	Negative	Separation of isobaric/isomeric interferece confirmed	Yes
Type of negative (precursor)ion	[M+CH3COO]-	Model for separation prediction	Yes
Fragments for identification		Additional dimension/techniques	-

Fragment name
Neutral loss of methyl moiety
Fatty acid fragment
Oxidized fatty acid fragment
Oxidized fatty acid -H2O fragment

Isotope correction at MS1	No	Lipid Identification Software	MS-DIAL
Isotope correction at MS2	No	Data manipulation	Smoothing, Centroiding
MS1 verified by standard	No	Nomenclature for intact lipid molecule	Yes
MS2 verified by standard	No	Nomenclature for fragment ions	No
Background check at MS1	Yes	Further identification remarks	-

103) Oxidized phosphatidylcholine (OxPC)[M+CH3COO]- / Lipid quantification

Quantitative	Yes	Limit of quantification	No
MS Level for quantification	MS1	Normalization to reference	No
Internal lipid standard(s) MS1		Lipid Quantification Software	MS-DIAL

Internal standard	Endogenous subclass
PC 15:0_18:1(d7)	OxPC subclass

Type of quantification	Internal standard amount	Batch correction	No
Response correction	No	Further quantification remarks	-
Type I isotope correction	No		

104) Oxidized phosphatidylethanolamine (OxPE)[M-H]- / Lipid identification

Lipid class	Oxidized phosphatidylethanolamine (OxPE)	Background check at MS2	No
Derivatization	-	Did you presume assumptions for identification?	No
MS Level for identification	MS1, MS2	Check isomer overlap	No
Identification level	Molecular species level	RT verified by standard	Yes
Polarity mode	Negative	Separation of isobaric/isomeric interferece confirmed	Yes
Type of negative (precursor)ion	[M-H]-	Model for separation prediction	Yes
Fragments for identification		Additional dimension/techniques	-

Fragment name
Neutral loss of H2O
Fatty acid fragment
Oxidized fatty acid fragment
Oxidized fatty acid -H2O fragment

Isotope correction at MS1	No	Lipid Identification Software	MS-DIAL
Isotope correction at MS2	No	Data manipulation	Smoothing, Centroiding
MS1 verified by standard	No	Nomenclature for intact lipid molecule	Yes
MS2 verified by standard	No	Nomenclature for fragment ions	No
Background check at MS1	Yes	Further identification remarks	-

104) Oxidized phosphatidylethanolamine (OxPE)[M-H]- / Lipid quantification

Quantitative	Yes	Limit of quantification	No
MS Level for quantification	MS1	Normalization to reference	No
Internal lipid standard(s) MS1		Lipid Quantification Software	MS-DIAL

Internal standard	Endogenous subclass
PE 15:0_18:1(d7)	OxPE subclass

Type of quantification	Internal standard amount	Batch correction	No
Response correction	No	Further quantification remarks	-
Type I isotope correction	No		

105) Oxidized phosphatidylglycerol (OxPG)[M-H]- / Lipid identification

Lipid class	Oxidized phosphatidylglycerol (OxPG)	Background check at MS2	No
Derivatization	-	Did you presume assumptions for identification?	No
MS Level for identification	MS1, MS2	Check isomer overlap	No
Identification level	Molecular species level	RT verified by standard	Yes
Polarity mode	Negative	Separation of isobaric/isomeric interferece confirmed	Yes
Type of negative (precursor)ion	[M-H]-	Model for separation prediction	Yes
Fragments for identification		Additional dimension/techniques	-

Fragment name
Fatty acid fragment
Oxidized fatty acid fragment
Oxidized fatty acid -H2O fragment

Isotope correction at MS1	No	Lipid Identification Software	MS-DIAL
Isotope correction at MS2	No	Data manipulation	Smoothing, Centroiding
MS1 verified by standard	No	Nomenclature for intact lipid molecule	Yes
MS2 verified by standard	No	Nomenclature for fragment ions	No
Background check at MS1	Yes	Further identification remarks	-

105) Oxidized phosphatidylglycerol (OxPG)[M-H]- / Lipid quantification

Quantitative	Yes	Limit of quantification	No
MS Level for quantification	MS1	Normalization to reference	No
Internal lipid standard(s) MS1		Lipid Quantification Software	MS-DIAL

Internal standard	Endogenous subclass
PG 15:0_18:1(d7)	OxPG subclass

Type of quantification	Internal standard amount	Batch correction	No
Response correction	No	Further quantification remarks	-
Type I isotope correction	No		

106) Oxidized phosphatidylinositol (OxPI)[M-H]- / Lipid identification

Lipid class	Oxidized phosphatidylinositol (OxPI)	Background check at MS2	No
Derivatization	-	Did you presume assumptions for identification?	No
MS Level for identification	MS1, MS2	Check isomer overlap	No
Identification level	Molecular species level	RT verified by standard	Yes
Polarity mode	Negative	Separation of isobaric/isomeric interferece confirmed	Yes
Type of negative (precursor)ion	[M-H]-	Model for separation prediction	Yes
Fragments for identification		Additional dimension/techniques	-

Fragment name
Phosphoinositol -H2O fragment
Characteristic fragment (C9H14O9P-)
Neutral loss of H2O
Fatty acid fragment
Oxidized fatty acid fragment
Oxidized fatty acid -H2O fragment

Isotope correction at MS1	No	Lipid Identification Software	MS-DIAL
Isotope correction at MS2	No	Data manipulation	Smoothing, Centroiding
MS1 verified by standard	No	Nomenclature for intact lipid molecule	Yes
MS2 verified by standard	No	Nomenclature for fragment ions	No
Background check at MS1	Yes	Further identification remarks	-

106) Oxidized phosphatidylinositol (OxPI)[M-H]- / Lipid quantification

Quantitative	Yes	Limit of quantification	No
MS Level for quantification	MS1	Normalization to reference	No
Internal lipid standard(s) MS1		Lipid Quantification Software	MS-DIAL

Internal standard	Endogenous subclass
PI 15:0_18:1(d7)	OxPI subclass

Type of quantification	Internal standard amount	Batch correction	No
Response correction	No	Further quantification remarks	-
Type I isotope correction	No		

107) Oxidized phosphatidylserine (OxPS)[M-H]- / Lipid identification

Lipid class	Oxidized phosphatidylserine (OxPS)	Background check at MS2	No
Derivatization	-	Did you presume assumptions for identification?	No
MS Level for identification	MS1, MS2	Check isomer overlap	No
Identification level	Molecular species level	RT verified by standard	Yes
Polarity mode	Negative	Separation of isobaric/isomeric interferece confirmed	Yes
Type of negative (precursor)ion	[M-H]-	Model for separation prediction	Yes
Fragments for identification		Additional dimension/techniques	-

Fragment name
Neutral loss of C3H6NO2
Neutral loss of H2O
Fatty acid fragment
Oxidized fatty acid fragment
Oxidized fatty acid -H2O fragment

Isotope correction at MS1	No	Lipid Identification Software	MS-DIAL
Isotope correction at MS2	No	Data manipulation	Smoothing, Centroiding
MS1 verified by standard	No	Nomenclature for intact lipid molecule	Yes
MS2 verified by standard	No	Nomenclature for fragment ions	No
Background check at MS1	Yes	Further identification remarks	-

107) Oxidized phosphatidylserine (OxPS)[M-H]- / Lipid quantification

Quantitative	Yes	Limit of quantification	No
MS Level for quantification	MS1	Normalization to reference	No
Internal lipid standard(s) MS1		Lipid Quantification Software	MS-DIAL

Internal standard	Endogenous subclass
PS 15:0_18:1(d7)	OxPS subclass

Type of quantification	Internal standard amount	Batch correction	No
Response correction	No	Further quantification remarks	-
Type I isotope correction	No		

108) Oxidized triglyceride (OxTG)[M+NH4]+ / Lipid identification

Lipid class	Oxidized triglyceride (OxTG)	Background check at MS2	No
Derivatization	-	Did you presume assumptions for identification?	No
MS Level for identification	MS1, MS2	Check isomer overlap	No
Identification level	Molecular species level	RT verified by standard	Yes
Polarity mode	Positive	Separation of isobaric/isomeric interferece confirmed	Yes
Type of positive (precursor)ion	[M+NH4]+	Model for separation prediction	Yes
Fragments for identification		Additional dimension/techniques	-

Fragment name
Neutral loss of acyl and H2O
Neutral loss of acyl and 2H2O
Neutral loss of acyl and H2O and O

Isotope correction at MS1	No	Lipid Identification Software	MS-DIAL
Isotope correction at MS2	No	Data manipulation	Smoothing, Centroiding
MS1 verified by standard	No	Nomenclature for intact lipid molecule	Yes
MS2 verified by standard	No	Nomenclature for fragment ions	No
Background check at MS1	Yes	Further identification remarks	-

108) Oxidized triglyceride (OxTG)[M+NH4]+ / Lipid quantification

Quantitative	Yes	Limit of quantification	No
MS Level for quantification	MS1	Normalization to reference	No
Internal lipid standard(s) MS1		Lipid Quantification Software	MS-DIAL

Internal standard	Endogenous subclass
TG 15:0_18:1(d7)_15:0	OxTG subclass

Type of quantification	Internal standard amount	Batch correction	No
Response correction	No	Further quantification remarks	-
Type I isotope correction	No		

109) PA[M-H]- / Lipid identification

Lipid class	PA	Background check at MS2	No
Derivatization	-	Did you presume assumptions for identification?	No
MS Level for identification	MS1, MS2	Check isomer overlap	No
Identification level	Molecular species level	RT verified by standard	Yes
Polarity mode	Negative	Separation of isobaric/isomeric interferece confirmed	Yes
Type of negative (precursor)ion	[M-H]-	Model for separation prediction	Yes
Fragments for identification		Additional dimension/techniques	-

Fragment name
Phosphoglycerol -H2O fragment
Fatty acid fragment

Isotope correction at MS1	No	Lipid Identification Software	MS-DIAL
Isotope correction at MS2	No	Data manipulation	Smoothing, Centroiding
MS1 verified by standard	No	Nomenclature for intact lipid molecule	Yes
MS2 verified by standard	No	Nomenclature for fragment ions	No
Background check at MS1	Yes	Further identification remarks	-

109) PA[M-H]- / Lipid quantification

Quantitative	Yes	Limit of quantification	No
MS Level for quantification	MS1	Normalization to reference	No
Internal lipid standard(s) MS1		Lipid Quantification Software	MS-DIAL

Internal standard	Endogenous subclass
LPC 18:1(d7)	PA subclass

Type of quantification	Internal standard amount	Batch correction	No
Response correction	No	Further quantification remarks	-
Type I isotope correction	No		

110) PC[M+CH3COO]- / Lipid identification

Lipid class	PC	Background check at MS2	No
Derivatization	-	Did you presume assumptions for identification?	No
MS Level for identification	MS1, MS2	Check isomer overlap	No
Identification level	Molecular species level	RT verified by standard	Yes
Polarity mode	Negative	Separation of isobaric/isomeric interferece confirmed	Yes
Type of negative (precursor)ion	[M+CH3COO]-	Model for separation prediction	Yes
Fragments for identification		Additional dimension/techniques	-

Fragment name
Neutral loss of methyl moiety
Fatty acid fragment

Isotope correction at MS1	No	Lipid Identification Software	MS-DIAL
Isotope correction at MS2	No	Data manipulation	Smoothing, Centroiding
MS1 verified by standard	Yes	Nomenclature for intact lipid molecule	Yes
MS2 verified by standard	Yes	Nomenclature for fragment ions	No
Background check at MS1	Yes	Further identification remarks	-

110) PC[M+CH3COO]- / Lipid quantification

Quantitative	Yes	Limit of quantification	No
MS Level for quantification	MS1	Normalization to reference	No
Internal lipid standard(s) MS1		Lipid Quantification Software	MS-DIAL

Internal standard	Endogenous subclass
PC 15:0_18:1(d7)	PC subclass

Type of quantification	Internal standard amount	Batch correction	No
Response correction	No	Further quantification remarks	-
Type I isotope correction	No		

111) Phosphatidylethanol (PEtOH)[M-H]- / Lipid identification

Lipid class	Phosphatidylethanol (PEtOH)	Background check at MS2	No
Derivatization	-	Did you presume assumptions for identification?	No
MS Level for identification	MS1, MS2	Check isomer overlap	No
Identification level	Molecular species level	RT verified by standard	Yes
Polarity mode	Negative	Separation of isobaric/isomeric interferece confirmed	Yes
Type of negative (precursor)ion	[M-H]-	Model for separation prediction	Yes
Fragments for identification		Additional dimension/techniques	-

Fragment name
Phosphoethanol
Fatty acid fragment

Isotope correction at MS1	No	Lipid Identification Software	MS-DIAL
Isotope correction at MS2	No	Data manipulation	Smoothing, Centroiding
MS1 verified by standard	No	Nomenclature for intact lipid molecule	Yes
MS2 verified by standard	No	Nomenclature for fragment ions	No
Background check at MS1	Yes	Further identification remarks	-

111) Phosphatidylethanol (PEtOH)[M-H]- / Lipid quantification

Quantitative	Yes	Limit of quantification	No
MS Level for quantification	MS1	Normalization to reference	No
Internal lipid standard(s) MS1		Lipid Quantification Software	MS-DIAL

Internal standard	Endogenous subclass
LPC 18:1(d7)	PEtOH subclass

Type of quantification	Internal standard amount	Batch correction	No
Response correction	No	Further quantification remarks	-
Type I isotope correction	No		

112) PE[M-H]- / Lipid identification

Lipid class	PE	Background check at MS2	No
Derivatization	-	Did you presume assumptions for identification?	No
MS Level for identification	MS1, MS2	Check isomer overlap	No
Identification level	Molecular species level	RT verified by standard	Yes
Polarity mode	Negative	Separation of isobaric/isomeric interferece confirmed	Yes
Type of negative (precursor)ion	[M-H]-	Model for separation prediction	Yes
Fragments for identification		Additional dimension/techniques	-

Fragment name
Characteristic fragment (C5H11NO5P-)
Fatty acid fragment

Isotope correction at MS1	No	Lipid Identification Software	MS-DIAL
Isotope correction at MS2	No	Data manipulation	Smoothing, Centroiding
MS1 verified by standard	Yes	Nomenclature for intact lipid molecule	Yes
MS2 verified by standard	Yes	Nomenclature for fragment ions	No
Background check at MS1	Yes	Further identification remarks	-

112) PE[M-H]- / Lipid quantification

Quantitative	Yes	Limit of quantification	No
MS Level for quantification	MS1	Normalization to reference	No
Internal lipid standard(s) MS1		Lipid Quantification Software	MS-DIAL

Internal standard	Endogenous subclass
PE 15:0_18:1(d7)	PE subclass

Type of quantification	Internal standard amount	Batch correction	No
Response correction	No	Further quantification remarks	-
Type I isotope correction	No		

113) PG[M-H]- / Lipid identification

Lipid class	PG	Background check at MS2	No
Derivatization	-	Did you presume assumptions for identification?	No
MS Level for identification	MS1, MS2	Check isomer overlap	No
Identification level	Molecular species level	RT verified by standard	Yes
Polarity mode	Negative	Separation of isobaric/isomeric interferece confirmed	Yes
Type of negative (precursor)ion	[M-H]-	Model for separation prediction	Yes
Fragments for identification		Additional dimension/techniques	-

Fragment name
Phosphoglycerol -H2O
Fatty acid fragment

Isotope correction at MS1	No	Lipid Identification Software	MS-DIAL
Isotope correction at MS2	No	Data manipulation	Smoothing, Centroiding
MS1 verified by standard	Yes	Nomenclature for intact lipid molecule	Yes
MS2 verified by standard	Yes	Nomenclature for fragment ions	No
Background check at MS1	Yes	Further identification remarks	-

113) PG[M-H]- / Lipid quantification

Quantitative	Yes	Limit of quantification	No
MS Level for quantification	MS1	Normalization to reference	No
Internal lipid standard(s) MS1		Lipid Quantification Software	MS-DIAL

Internal standard	Endogenous subclass
PG 15:0_18:1(d7)	PG subclass

Type of quantification	Internal standard amount	Batch correction	No
Response correction	No	Further quantification remarks	-
Type I isotope correction	No		

114) PI[M-H]- / Lipid identification

Lipid class	PI	Background check at MS2	No
Derivatization	-	Did you presume assumptions for identification?	No
MS Level for identification	MS1, MS2	Check isomer overlap	No
Identification level	Molecular species level	RT verified by standard	Yes
Polarity mode	Negative	Separation of isobaric/isomeric interferece confirmed	Yes
Type of negative (precursor)ion	[M-H]-	Model for separation prediction	Yes
Fragments for identification		Additional dimension/techniques	-

Fragment name
Phosphoinositol -H2O
Characteristic fragment (C9H14O9P-)
Fatty acid fragment

Isotope correction at MS1	No	Lipid Identification Software	MS-DIAL
Isotope correction at MS2	No	Data manipulation	Smoothing, Centroiding
MS1 verified by standard	Yes	Nomenclature for intact lipid molecule	Yes
MS2 verified by standard	Yes	Nomenclature for fragment ions	No
Background check at MS1	Yes	Further identification remarks	-

114) PI[M-H]- / Lipid quantification

Quantitative	Yes	Limit of quantification	No
MS Level for quantification	MS1	Normalization to reference	No
Internal lipid standard(s) MS1		Lipid Quantification Software	MS-DIAL

Internal standard	Endogenous subclass
PI 15:0_18:1(d7)	PI subclass

Type of quantification	Internal standard amount	Batch correction	No
Response correction	No	Further quantification remarks	-
Type I isotope correction	No		

115) Phosphatidylmethanol (PMeOH)[M-H]- / Lipid identification

Lipid class	Phosphatidylmethanol (PMeOH)	Background check at MS2	No
Derivatization	-	Did you presume assumptions for identification?	No
MS Level for identification	MS1, MS2	Check isomer overlap	No
Identification level	Molecular species level	RT verified by standard	Yes
Polarity mode	Negative	Separation of isobaric/isomeric interferece confirmed	Yes
Type of negative (precursor)ion	[M-H]-	Model for separation prediction	Yes
Fragments for identification		Additional dimension/techniques	-

Fragment name
Phosphomethanol
Fatty acid fragment

Isotope correction at MS1	No	Lipid Identification Software	MS-DIAL
Isotope correction at MS2	No	Data manipulation	Smoothing, Centroiding
MS1 verified by standard	No	Nomenclature for intact lipid molecule	Yes
MS2 verified by standard	No	Nomenclature for fragment ions	No
Background check at MS1	Yes	Further identification remarks	-

115) Phosphatidylmethanol (PMeOH)[M-H]- / Lipid quantification

Quantitative	Yes	Limit of quantification	No
MS Level for quantification	MS1	Normalization to reference	No
Internal lipid standard(s) MS1		Lipid Quantification Software	MS-DIAL

Internal standard	Endogenous subclass
LPC 18:1(d7)	PMeOH subclass

Type of quantification	Internal standard amount	Batch correction	No
Response correction	No	Further quantification remarks	-
Type I isotope correction	No		

116) PS[M-H]- / Lipid identification

Lipid class	PS	Background check at MS2	No
Derivatization	-	Did you presume assumptions for identification?	No
MS Level for identification	MS1, MS2	Check isomer overlap	No
Identification level	Molecular species level	RT verified by standard	Yes
Polarity mode	Negative	Separation of isobaric/isomeric interferece confirmed	Yes
Type of negative (precursor)ion	[M-H]-	Model for separation prediction	Yes
Fragments for identification		Additional dimension/techniques	-

Fragment name
Neutral loss of C3H6NO2
Fatty acid fragment

Isotope correction at MS1	No	Lipid Identification Software	MS-DIAL
Isotope correction at MS2	No	Data manipulation	Smoothing, Centroiding
MS1 verified by standard	Yes	Nomenclature for intact lipid molecule	Yes
MS2 verified by standard	Yes	Nomenclature for fragment ions	No
Background check at MS1	Yes	Further identification remarks	-

116) PS[M-H]- / Lipid quantification

Quantitative	Yes	Limit of quantification	No
MS Level for quantification	MS1	Normalization to reference	No
Internal lipid standard(s) MS1		Lipid Quantification Software	MS-DIAL

Internal standard	Endogenous subclass
PS 15:0_18:1(d7)	PS subclass

Type of quantification	Internal standard amount	Batch correction	No
Response correction	No	Further quantification remarks	-
Type I isotope correction	No		

117) Phytosphingosine (PhytoSph)[M+H]+ / Lipid identification

Lipid class	Phytosphingosine (PhytoSph)	Background check at MS2	No
Derivatization	-	Did you presume assumptions for identification?	No
MS Level for identification	MS1, MS2	Check isomer overlap	No
Identification level	Molecular species level	RT verified by standard	Yes
Polarity mode	Positive	Separation of isobaric/isomeric interferece confirmed	Yes
Type of positive (precursor)ion	[M+H]+	Model for separation prediction	Yes
Fragments for identification		Additional dimension/techniques	-

Fragment name
Neutral loss of H2O
Neutral loss of 2H2O
Neutral loss of 3H2O
Neutral loss of CH4O2

Isotope correction at MS1	No	Lipid Identification Software	MS-DIAL
Isotope correction at MS2	No	Data manipulation	Smoothing, Centroiding
MS1 verified by standard	No	Nomenclature for intact lipid molecule	Yes
MS2 verified by standard	No	Nomenclature for fragment ions	No
Background check at MS1	Yes	Further identification remarks	-

117) Phytosphingosine (PhytoSph)[M+H]+ / Lipid quantification

Quantitative	Yes	Limit of quantification	No
MS Level for quantification	MS1	Normalization to reference	No
Internal lipid standard(s) MS1		Lipid Quantification Software	MS-DIAL

Internal standard	Endogenous subclass
Cer 18:1;2O/15:0(d7)	PhytoSph subclass

Type of quantification	Internal standard amount	Batch correction	No
Response correction	No	Further quantification remarks	-
Type I isotope correction	No		

118) Semino lipid (EtherSMGDG)[M-H]- / Lipid identification

Lipid class	Semino lipid (EtherSMGDG)	Background check at MS2	No
Derivatization	-	Did you presume assumptions for identification?	No
MS Level for identification	MS1, MS2	Check isomer overlap	No
Identification level	Molecular species level	RT verified by standard	Yes
Polarity mode	Negative	Separation of isobaric/isomeric interferece confirmed	Yes
Type of negative (precursor)ion	[M-H]-	Model for separation prediction	Yes
Fragments for identification		Additional dimension/techniques	-

Fragment name
Sulfate
Hexosylsulfate
Neutral loss of acyl and H2O

Isotope correction at MS1	No	Lipid Identification Software	MS-DIAL
Isotope correction at MS2	No	Data manipulation	Smoothing, Centroiding
MS1 verified by standard	No	Nomenclature for intact lipid molecule	Yes
MS2 verified by standard	No	Nomenclature for fragment ions	No
Background check at MS1	Yes	Further identification remarks	-

118) Semino lipid (EtherSMGDG)[M-H]- / Lipid quantification

Quantitative	Yes	Limit of quantification	No
MS Level for quantification	MS1	Normalization to reference	No
Internal lipid standard(s) MS1		Lipid Quantification Software	MS-DIAL

Internal standard	Endogenous subclass
Cer 18:1;2O/15:0(d7)	EtherSMGDG subclass

Type of quantification	Internal standard amount	Batch correction	No
Response correction	No	Further quantification remarks	-
Type I isotope correction	No		

119) Semino lipid (SMGDG)[M-H]- / Lipid identification

Lipid class	Semino lipid (SMGDG)	Background check at MS2	No
Derivatization	-	Did you presume assumptions for identification?	No
MS Level for identification	MS1, MS2	Check isomer overlap	No
Identification level	Molecular species level	RT verified by standard	Yes
Polarity mode	Negative	Separation of isobaric/isomeric interferece confirmed	Yes
Type of negative (precursor)ion	[M-H]-	Model for separation prediction	Yes
Fragments for identification		Additional dimension/techniques	-

Fragment name
Sulfate
Hexosylsulfate
Neutral loss of acyl and H2O
Fatty acid fragment

Isotope correction at MS1	No	Lipid Identification Software	MS-DIAL
Isotope correction at MS2	No	Data manipulation	Smoothing, Centroiding
MS1 verified by standard	No	Nomenclature for intact lipid molecule	Yes
MS2 verified by standard	No	Nomenclature for fragment ions	No
Background check at MS1	Yes	Further identification remarks	-

119) Semino lipid (SMGDG)[M-H]- / Lipid quantification

Quantitative	Yes	Limit of quantification	No
MS Level for quantification	MS1	Normalization to reference	No
Internal lipid standard(s) MS1		Lipid Quantification Software	MS-DIAL

Internal standard	Endogenous subclass
Cer 18:1;2O/15:0(d7)	SMGDG subclass

Type of quantification	Internal standard amount	Batch correction	No
Response correction	No	Further quantification remarks	-
Type I isotope correction	No		

120) Sitosterol ester (SISE)[M+NH4]+ / Lipid identification

Lipid class	Sitosterol ester (SISE)	Background check at MS2	No
Derivatization	-	Did you presume assumptions for identification?	No
MS Level for identification	MS1, MS2	Check isomer overlap	No
Identification level	Molecular species level	RT verified by standard	Yes
Polarity mode	Positive	Separation of isobaric/isomeric interferece confirmed	Yes
Type of positive (precursor)ion	[M+NH4]+	Model for separation prediction	Yes
Fragments for identification		Additional dimension/techniques	-

Fragment name
Neutral loss of fatty acid

Isotope correction at MS1	No	Lipid Identification Software	MS-DIAL
Isotope correction at MS2	No	Data manipulation	Smoothing, Centroiding
MS1 verified by standard	No	Nomenclature for intact lipid molecule	Yes
MS2 verified by standard	No	Nomenclature for fragment ions	No
Background check at MS1	Yes	Further identification remarks	-

120) Sitosterol ester (SISE)[M+NH4]+ / Lipid quantification

Quantitative	Yes	Limit of quantification	No
MS Level for quantification	MS1	Normalization to reference	No
Internal lipid standard(s) MS1		Lipid Quantification Software	MS-DIAL

Internal standard	Endogenous subclass
CE 18:1(d7)	SISE subclass

Type of quantification	Internal standard amount	Batch correction	No
Response correction	No	Further quantification remarks	-
Type I isotope correction	No		

121) Sphinganine (DHSph)[M+H]+ / Lipid identification

Lipid class	Sphinganine (DHSph)	Background check at MS2	No
Derivatization	-	Did you presume assumptions for identification?	No
MS Level for identification	MS1, MS2	Check isomer overlap	No
Identification level	Molecular species level	RT verified by standard	Yes
Polarity mode	Positive	Separation of isobaric/isomeric interferece confirmed	Yes
Type of positive (precursor)ion	[M+H]+	Model for separation prediction	Yes
Fragments for identification		Additional dimension/techniques	-

Fragment name
Neutral loss of H2O
Neutral loss of 2H2O
Neutral loss of CH4O2

Isotope correction at MS1	No	Lipid Identification Software	MS-DIAL
Isotope correction at MS2	No	Data manipulation	Smoothing, Centroiding
MS1 verified by standard	No	Nomenclature for intact lipid molecule	Yes
MS2 verified by standard	No	Nomenclature for fragment ions	No
Background check at MS1	Yes	Further identification remarks	-

121) Sphinganine (DHSph)[M+H]+ / Lipid quantification

Quantitative	Yes	Limit of quantification	No
MS Level for quantification	MS1	Normalization to reference	No
Internal lipid standard(s) MS1		Lipid Quantification Software	MS-DIAL

Internal standard	Endogenous subclass
Cer 18:1;2O/15:0(d7)	DHSph subclass

Type of quantification	Internal standard amount	Batch correction	No
Response correction	No	Further quantification remarks	-
Type I isotope correction	No		

122) Sphingomyelin (SM)[M+CH3COO]- / Lipid identification

Lipid class	Sphingomyelin (SM)	Background check at MS2	No
Derivatization	-	Did you presume assumptions for identification?	No
MS Level for identification	MS1, MS2	Check isomer overlap	No
Identification level	Molecular species level	RT verified by standard	Yes
Polarity mode	Negative	Separation of isobaric/isomeric interferece confirmed	Yes
Type of negative (precursor)ion	[M+CH3COO]-	Model for separation prediction	Yes
Fragments for identification		Additional dimension/techniques	-

Fragment name
Neutral loss of methyl moiety
Characteristic fragment (C4H11NO4P-)
Neutral loss of methyl moiety and acyl

Isotope correction at MS1	No	Lipid Identification Software	MS-DIAL
Isotope correction at MS2	No	Data manipulation	Smoothing, Centroiding
MS1 verified by standard	Yes	Nomenclature for intact lipid molecule	Yes
MS2 verified by standard	Yes	Nomenclature for fragment ions	No
Background check at MS1	Yes	Further identification remarks	-

122) Sphingomyelin (SM)[M+CH3COO]- / Lipid quantification

Quantitative	Yes	Limit of quantification	No
MS Level for quantification	MS1	Normalization to reference	No
Internal lipid standard(s) MS1		Lipid Quantification Software	MS-DIAL

Internal standard	Endogenous subclass
SM 18:1;2O/18:1(d9)	SM subclass

Type of quantification	Internal standard amount	Batch correction	No
Response correction	No	Further quantification remarks	-
Type I isotope correction	No		

123) SPB[M+H]+ / Lipid identification

Lipid class	SPB	Background check at MS2	No
Derivatization	-	Did you presume assumptions for identification?	No
MS Level for identification	MS1, MS2	Check isomer overlap	No
Identification level	Molecular species level	RT verified by standard	Yes
Polarity mode	Positive	Separation of isobaric/isomeric interferece confirmed	Yes
Type of positive (precursor)ion	[M+H]+	Model for separation prediction	Yes
Fragments for identification		Additional dimension/techniques	-

Fragment name
Neutral loss of H_2O
Neutral loss of $2H_2O$
Neutral loss of CH_4O_2

Isotope correction at MS1	No	Lipid Identification Software	MS-DIAL
Isotope correction at MS2	No	Data manipulation	Smoothing, Centroiding
MS1 verified by standard	No	Nomenclature for intact lipid molecule	Yes
MS2 verified by standard	No	Nomenclature for fragment ions	No
Background check at MS1	Yes	Further identification remarks	-

123) SPB[M+H]+ / Lipid quantification

Quantitative	Yes	Limit of quantification	No
MS Level for quantification	MS1	Normalization to reference	No
Internal lipid standard(s) MS1		Lipid Quantification Software	MS-DIAL

Internal standard	Endogenous subclass
Cer 18:1;2O/15:0(d7)	Sph subclass

Type of quantification	Internal standard amount	Batch correction	No
Response correction	No	Further quantification remarks	-
Type I isotope correction	No		

124) Sterol sulfate (SSulfate)[M-H]- / Lipid identification

Lipid class	Sterol sulfate (SSulfate)	Background check at MS2	No
Derivatization	-	Did you presume assumptions for identification?	No
MS Level for identification	MS1, MS2	Check isomer overlap	No
Identification level	Molecular species level	RT verified by standard	Yes
Polarity mode	Negative	Separation of isobaric/isomeric interferece confirmed	Yes
Type of negative (precursor)ion	[M-H]-	Model for separation prediction	Yes
Fragments for identification		Additional dimension/techniques	-

Fragment name
Cholesterol sulfate
Sulfate

Isotope correction at MS1	No	Lipid Identification Software	MS-DIAL
Isotope correction at MS2	No	Data manipulation	Smoothing, Centroiding
MS1 verified by standard	No	Nomenclature for intact lipid molecule	Yes
MS2 verified by standard	No	Nomenclature for fragment ions	No
Background check at MS1	Yes	Further identification remarks	-

124) Sterol sulfate (SSulfate)[M-H]- / Lipid quantification

Quantitative	Yes	Limit of quantification	No
MS Level for quantification	MS1	Normalization to reference	No
Internal lipid standard(s) MS1		Lipid Quantification Software	MS-DIAL

Internal standard	Endogenous subclass
LPC 18:1(d7)	SSulfate subclass

Type of quantification	Internal standard amount	Batch correction	No
Response correction	No	Further quantification remarks	-
Type I isotope correction	No		

125) Stigmasterol ester (STSE)[M+NH4]+ / Lipid identification

Lipid class	Stigmasterol ester (STSE)	Background check at MS2	No
Derivatization	-	Did you presume assumptions for identification?	No
MS Level for identification	MS1, MS2	Check isomer overlap	No
Identification level	Molecular species level	RT verified by standard	Yes
Polarity mode	Positive	Separation of isobaric/isomeric interferece confirmed	Yes
Type of positive (precursor)ion	[M+NH4]+	Model for separation prediction	Yes
Fragments for identification		Additional dimension/techniques	-

Fragment name
Neutral loss of fatty acid

Isotope correction at MS1	No	Lipid Identification Software	MS-DIAL
Isotope correction at MS2	No	Data manipulation	Smoothing, Centroiding
MS1 verified by standard	No	Nomenclature for intact lipid molecule	Yes
MS2 verified by standard	No	Nomenclature for fragment ions	No
Background check at MS1	Yes	Further identification remarks	-

125) Stigmasterol ester (STSE)[M+NH4]+ / Lipid quantification

Quantitative	Yes	Limit of quantification	No
MS Level for quantification	MS1	Normalization to reference	No
Internal lipid standard(s) MS1		Lipid Quantification Software	MS-DIAL

Internal standard	Endogenous subclass
CE 18:1(d7)	STSE subclass

Type of quantification	Internal standard amount	Batch correction	No
Response correction	No	Further quantification remarks	-
Type I isotope correction	No		

126) Stigmasterol hexoside (SHex)[M+CH3COO]- / Lipid identification

Lipid class	Stigmasterol hexoside (SHex)	Background check at MS2	No
Derivatization	-	Did you presume assumptions for identification?	No
MS Level for identification	MS1, MS2	Check isomer overlap	No
Identification level	Molecular species level	RT verified by standard	Yes
Polarity mode	Negative	Separation of isobaric/isomeric interferece confirmed	Yes
Type of negative (precursor)ion	[M+CH3COO]-	Model for separation prediction	Yes
Fragments for identification		Additional dimension/techniques	-

Fragment name
Hexose

Isotope correction at MS1	No	Lipid Identification Software	MS-DIAL
Isotope correction at MS2	No	Data manipulation	Smoothing, Centroiding
MS1 verified by standard	No	Nomenclature for intact lipid molecule	Yes
MS2 verified by standard	No	Nomenclature for fragment ions	No
Background check at MS1	Yes	Further identification remarks	-

126) Stigmasterol hexoside (SHex)[M+CH3COO]- / Lipid quantification

Quantitative	Yes	Limit of quantification	No
MS Level for quantification	MS1	Normalization to reference	No
Internal lipid standard(s) MS1		Lipid Quantification Software	MS-DIAL

Internal standard	Endogenous subclass
LPC 18:1(d7)	SHex subclass

Type of quantification	Internal standard amount	Batch correction	No
Response correction	No	Further quantification remarks	-
Type I isotope correction	No		

127) SHexCer[M+H]+ / Lipid identification

Lipid class	SHexCer	Background check at MS2	No
Derivatization	-	Did you presume assumptions for identification?	No
MS Level for identification	MS1, MS2	Check isomer overlap	No
Identification level	Molecular species level	RT verified by standard	Yes
Polarity mode	Positive	Separation of isobaric/isomeric interferece confirmed	Yes
Type of positive (precursor)ion	[M+H]+	Model for separation prediction	Yes
Fragments for identification		Additional dimension/techniques	-

Fragment name
Neutral loss of sulfate
Neutral loss of sulfate and hexose
Sphingosine -H2O fragment
Sphingosine -2H2O fragment
Sphingosine -CH4O2 fragment

Isotope correction at MS1	No	Lipid Identification Software	MS-DIAL
Isotope correction at MS2	No	Data manipulation	Smoothing, Centroiding
MS1 verified by standard	No	Nomenclature for intact lipid molecule	Yes
MS2 verified by standard	No	Nomenclature for fragment ions	No
Background check at MS1	Yes	Further identification remarks	-

127) SHexCer[M+H]+ / Lipid quantification

Quantitative	Yes	Limit of quantification	No
MS Level for quantification	MS1	Normalization to reference	No
Internal lipid standard(s) MS1		Lipid Quantification Software	MS-DIAL

Internal standard	Endogenous subclass
Cer 18:1;2O/15:0(d7)	SHexCer subclass

Type of quantification	Internal standard amount	Batch correction	No
Response correction	No	Further quantification remarks	-
Type I isotope correction	No		

128) SQDG[M+NH4]+ / Lipid identification

Lipid class	SQDG	Background check at MS2	No
Derivatization	-	Did you presume assumptions for identification?	No
MS Level for identification	MS1, MS2	Check isomer overlap	No
Identification level	Molecular species level	RT verified by standard	Yes
Polarity mode	Positive	Separation of isobaric/isomeric interferece confirmed	Yes
Type of positive (precursor)ion	[M+NH4]+	Model for separation prediction	Yes
Fragments for identification		Additional dimension/techniques	-

Fragment name
Neutral loss of C6H10O7S
Dehydro-monoacyl glycerols

Isotope correction at MS1	No	Lipid Identification Software	MS-DIAL
Isotope correction at MS2	No	Data manipulation	Smoothing, Centroiding
MS1 verified by standard	No	Nomenclature for intact lipid molecule	Yes
MS2 verified by standard	No	Nomenclature for fragment ions	No
Background check at MS1	Yes	Further identification remarks	-

128) SQDG[M+NH4]+ / Lipid quantification

Quantitative	Yes	Limit of quantification	No
MS Level for quantification	MS1	Normalization to reference	No
Internal lipid standard(s) MS1		Lipid Quantification Software	MS-DIAL

Internal standard	Endogenous subclass
LPC 18:1(d7)	SQDG subclass

Type of quantification	Internal standard amount	Batch correction	No
Response correction	No	Further quantification remarks	-
Type I isotope correction	No		

129) TG[M+NH4]+ / Lipid identification

Lipid class	TG	Background check at MS2	No
Derivatization	-	Did you presume assumptions for identification?	No
MS Level for identification	MS1, MS2	Check isomer overlap	No
Identification level	Molecular species level	RT verified by standard	Yes
Polarity mode	Positive	Separation of isobaric/isomeric interferece confirmed	Yes
Type of positive (precursor)ion	[M+NH4]+	Model for separation prediction	Yes
Fragments for identification		Additional dimension/techniques	-

Fragment name
Neutral loss of acyl and H2O

Isotope correction at MS1	No	Lipid Identification Software	MS-DIAL
Isotope correction at MS2	No	Data manipulation	Smoothing, Centroiding
MS1 verified by standard	Yes	Nomenclature for intact lipid molecule	Yes
MS2 verified by standard	Yes	Nomenclature for fragment ions	No
Background check at MS1	Yes	Further identification remarks	-

129) TG[M+NH4]+ / Lipid quantification

Quantitative	Yes	Limit of quantification	No
MS Level for quantification	MS1	Normalization to reference	No
Internal lipid standard(s) MS1		Lipid Quantification Software	MS-DIAL

Internal standard	Endogenous subclass
TG 15:0_18:1(d7)_15:0	TG subclass

Type of quantification	Internal standard amount	Batch correction	No
Response correction	No	Further quantification remarks	-
Type I isotope correction	No		

130) Triacylglycerol estolides (TG_EST)[M+NH4]+ / Lipid identification

Lipid class	Triacylglycerol estolides (TG_EST)	Background check at MS2	No
Derivatization	-	Did you presume assumptions for identification?	No
MS Level for identification	MS1, MS2	Check isomer overlap	No
Identification level	Molecular species level	RT verified by standard	Yes
Polarity mode	Positive	Separation of isobaric/isomeric interferece confirmed	Yes
Type of positive (precursor)ion	[M+NH4]+	Model for separation prediction	Yes
Fragments for identification		Additional dimension/techniques	-

Fragment name
Neutral loss of acyl and H2O
Neutral loss of oxidized acyl and H2O
Neutral loss of FAHFA

Isotope correction at MS1	No	Lipid Identification Software	MS-DIAL
Isotope correction at MS2	No	Data manipulation	Smoothing, Centroiding
MS1 verified by standard	No	Nomenclature for intact lipid molecule	Yes
MS2 verified by standard	No	Nomenclature for fragment ions	No
Background check at MS1	Yes	Further identification remarks	-

130) Triacylglycerol estolides (TG_EST)[M+NH4]+ / Lipid quantification

Quantitative	Yes	Limit of quantification	No
MS Level for quantification	MS1	Normalization to reference	No
Internal lipid standard(s) MS1		Lipid Quantification Software	MS-DIAL

Internal standard	Endogenous subclass
TG 15:0_18:1(d7)_15:0	TG_EST subclass

Type of quantification	Internal standard amount	Batch correction	No
Response correction	No	Further quantification remarks	-
Type I isotope correction	No		

131) Hex3Cer[M+H]+ / Lipid identification

Lipid class	Hex3Cer	Background check at MS2	No
Derivatization	-	Did you presume assumptions for identification?	No
MS Level for identification	MS1, MS2	Check isomer overlap	No
Identification level	Molecular species level	RT verified by standard	Yes
Polarity mode	Positive	Separation of isobaric/isomeric interferece confirmed	Yes
Type of positive (precursor)ion	[M+H]+	Model for separation prediction	Yes
Fragments for identification		Additional dimension/techniques	-

Fragment name
Neutral loss of hexose
Neutral loss of 2hexose
Neutral loss of 3hexose
Neutral loss of 3hexose and H2O
Sphingosine -H2O fragment
Sphingosine -2H2O fragment
Sphingosine -CH4O2 fragment

Isotope correction at MS1	No	Lipid Identification Software	MS-DIAL
Isotope correction at MS2	No	Data manipulation	Smoothing, Centroiding
MS1 verified by standard	No	Nomenclature for intact lipid molecule	Yes
MS2 verified by standard	No	Nomenclature for fragment ions	No
Background check at MS1	Yes	Further identification remarks	-

131) Hex3Cer[M+H]+ / Lipid quantification

Quantitative	Yes	Limit of quantification	No
MS Level for quantification	MS1	Normalization to reference	No
Internal lipid standard(s) MS1		Lipid Quantification Software	MS-DIAL

Internal standard	Endogenous subclass
Cer 18:1;2O/15:0(d7)	Hex3Cer subclass

Type of quantification	Internal standard amount	Batch correction	No
Response correction	No	Further quantification remarks	-
Type I isotope correction	No		

132) Vitamin A fatty acid ester (VAE)[M+Na]+ / Lipid identification

Lipid class	Vitamin A fatty acid ester (VAE)	Background check at MS2	No
Derivatization	-	Did you presume assumptions for identification?	No
MS Level for identification	MS1, MS2	Check isomer overlap	No
Identification level	Molecular species level	RT verified by standard	Yes
Polarity mode	Positive	Separation of isobaric/isomeric interferece confirmed	Yes
Type of positive (precursor)ion	[M+Na]+	Model for separation prediction	Yes
Fragments for identification		Additional dimension/techniques	-

Fragment name
Characteristic fragment (C20H29+)
Characteristic fragment (C9H11+)

Isotope correction at MS1	No	Lipid Identification Software	MS-DIAL
Isotope correction at MS2	No	Data manipulation	Smoothing, Centroiding
MS1 verified by standard	No	Nomenclature for intact lipid molecule	Yes
MS2 verified by standard	No	Nomenclature for fragment ions	No
Background check at MS1	Yes	Further identification remarks	-

132) Vitamin A fatty acid ester (VAE)[M+Na]+ / Lipid quantification

Quantitative	Yes	Limit of quantification	No
MS Level for quantification	MS1	Normalization to reference	No
Internal lipid standard(s) MS1		Lipid Quantification Software	MS-DIAL

Internal standard	Endogenous subclass
LPC 18:1(d7)	VAE subclass

Type of quantification	Internal standard amount	Batch correction	No
Response correction	No	Further quantification remarks	-
Type I isotope correction	No		

133) Vitamin D[M+H]+ / Lipid identification

Lipid class	Vitamin D	Background check at MS2	No
Derivatization	-	Did you presume assumptions for identification?	No
MS Level for identification	MS1, MS2	Check isomer overlap	No
Identification level	Species level	RT verified by standard	Yes
Polarity mode	Positive	Separation of isobaric/isomeric interferece confirmed	Yes
Type of positive (precursor)ion	[M+H]+	Model for separation prediction	Yes
Fragments for identification		Additional dimension/techniques	-

Fragment name
Neutral loss of H2O
Neutral loss of 2H2O

Isotope correction at MS1	No	Lipid Identification Software	MS-DIAL
Isotope correction at MS2	No	Data manipulation	Smoothing, Centroiding
MS1 verified by standard	No	Nomenclature for intact lipid molecule	Yes
MS2 verified by standard	No	Nomenclature for fragment ions	No
Background check at MS1	Yes	Further identification remarks	-

133) Vitamin D[M+H]+ / Lipid quantification

Quantitative	Yes	Limit of quantification	No
MS Level for quantification	MS1	Normalization to reference	No
Internal lipid standard(s) MS1		Lipid Quantification Software	MS-DIAL

Internal standard	Endogenous subclass
LPC 18:1(d7)	Vitamin D subclass

Type of quantification	Internal standard amount	Batch correction	No
Response correction	No	Further quantification remarks	-
Type I isotope correction	No		

134) Vitamin E[M+CH3COO]- / Lipid identification

Lipid class	Vitamin E	Background check at MS2	No
Derivatization	-	Did you presume assumptions for identification?	No
MS Level for identification	MS1, MS2	Check isomer overlap	No
Identification level	Species level	RT verified by standard	Yes
Polarity mode	Negative	Separation of isobaric/isomeric interferece confirmed	Yes
Type of negative (precursor)ion	[M+CH3COO]-	Model for separation prediction	Yes
Fragments for identification		Additional dimension/techniques	-

Fragment name
Characteristic fragment (C10H11O2-)

Isotope correction at MS1	No	Lipid Identification Software	MS-DIAL
Isotope correction at MS2	No	Data manipulation	Smoothing, Centroiding
MS1 verified by standard	No	Nomenclature for intact lipid molecule	Yes
MS2 verified by standard	No	Nomenclature for fragment ions	No
Background check at MS1	Yes	Further identification remarks	-

134) Vitamin E[M+CH3COO]- / Lipid quantification

Quantitative	Yes	Limit of quantification	No
MS Level for quantification	MS1	Normalization to reference	No
Internal lipid standard(s) MS1		Lipid Quantification Software	MS-DIAL

Internal standard	Endogenous subclass
LPC 18:1(d7)	Vitamin E subclass

Type of quantification	Internal standard amount	Batch correction	No
Response correction	No	Further quantification remarks	-
Type I isotope correction	No		

135) PE P[M+H]+ / Lipid identification

Lipid class	PE P	Background check at MS2	No
Derivatization	-	Did you presume assumptions for identification?	No
MS Level for identification	MS1, MS2	Check isomer overlap	No
Identification level	Molecular species level	RT verified by standard	Yes
Polarity mode	Positive	Separation of isobaric/isomeric interferece confirmed	Yes
Type of positive (precursor)ion	[M+H]+	Model for separation prediction	Yes
Fragments for identification		Additional dimension/techniques	-

Fragment name
Neutral loss of C2H8NO4P
Alkyl ether +C2H8NO3P fragment
Dehydro-monoacyl glycerols

Isotope correction at MS1	No	Lipid Identification Software	MS-DIAL
Isotope correction at MS2	No	Data manipulation	Smoothing, Centroiding
MS1 verified by standard	No	Nomenclature for intact lipid molecule	Yes
MS2 verified by standard	No	Nomenclature for fragment ions	No
Background check at MS1	Yes	Further identification remarks	-

135) PE P[M+H]+ / Lipid quantification

Quantitative	Yes	Limit of quantification	No
MS Level for quantification	MS1	Normalization to reference	No
Internal lipid standard(s) MS1		Lipid Quantification Software	MS-DIAL

Internal standard	Endogenous subclass
PE 15:0_18:1(d7)	EtherPE subclass

Type of quantification	Internal standard amount	Batch correction	No
Response correction	No	Further quantification remarks	-
Type I isotope correction	No		

Note 4. Lipidomics minimal reporting checklist for lysophospholipid profiling by using trimethylsilyl-diazomethane for HeLa cells.

Separation Workflow

Created by https://lipidomicstandards.org, version v2.3.2

Overall study design

Title of the study	Lysophospholipid profiling by using trimethylsilyl-diazomethane for HeLa cells		
Document creation date	02/07/2024	Corresponding Email	htusgawa@go.tuat.ac.jp
Principle investigator	Hiroshi Tsugawa	Is the workflow targeted or untargeted?	Targeted
Institution	Tokyo University of Agriculture and Technology	Clinical	No

Lipid extraction

Extraction method	1-phase system	1-phase system	Methanol
pH adjustment	None	Were internal standards added prior extraction?	No

Analytical platform

Which solvents were used	(A) acetonitrile (ACN):MeOH:H2O (1:1:3, v/v/v) and (B) ACN:IPA (1:9, v/v). Both the solvents contained 10 nM ethylenediaminetetraacetic acid and 5 mM ammonium acetate.	Mass resolution for detected ion at MS1	High resolution
Number of separation dimensions	One dimension	Resolution at m/z 200 at MS1	25148
Separation type 1	LC	Mass accuracy in ppm at MS1	0.3
Separation mode 1 (liquid)	RP	Mass window for precursor ion isolation (in Da total isolation window)	1
Detector	Mass spectrometer	Mass resolution for detected ion at MS2	High resolution
MS type	QTOF	Resolution at m/z 200 at MS2	26373
MS vendor	SCIEX	Mass accuracy in ppm at MS2	0.67
Ion source	ESI	Was/Were additional dimension/techniques used	No
MS Level	MS1, MS2		

Quality control

Blanks	Yes	Quality control	No
Type of Blanks	Extraction blank, Injection blank		

Method qualification and validation

Method validation	Yes	Precison	Yes
Lipid recovery	Yes	Accuracy	Yes
Dynamic quantification range	No	Guidelines followed	None
Limit of quantitation (LOQ)/Limit of detection (LOD)	No		

Reporting

Are reported raw data uploaded into repository?	Yes	Summary data	Quantification and identification data
Link to repository / ID to entry	http://prime.psc.riken.jp/menta.cgi/prime/drop_index	Raw data	Yes
Are metadata available?	Yes	Additional comments	The resolution and accuracy (ppm) for MS1 are those of m/z 132.9049 which were determined by the mass calibration in SCIEX OS. In addition, the resolution and accuracy for MS2 are those of m/z 185.1284, which were also determined by the mass calibration in SCIEX OS.

Sample Descriptions

HeLa cell with the supplementation of VLC-PUFA (FA 32:6) / Human / Cells

Provided information	Time to freeze (min), Storage time (month), Freeze-thaw cycles	Storage time (month)	1
Temperature handling original sample	4-8 °C	Freeze-thaw cycles	0
Instant sample preparation	No	Additives	None
Time to freeze (min)	10	Were samples stored under inert gas?	No
Snap freezing in liquid N2	Yes	Additional preservation methods	No
Storage temperature	-80 °C	Biobank samples	No

Lipid Class Descriptions

1) LPA[M+H]+ / Lipid identification

Lipid class	LPA	Background check at MS2	Yes
Derivatization	Trimethylsilyl (TMS)-diazomethane	Did you presume assumptions for identification?	No
MS Level for identification	MS1, MS2	Check isomer overlap	Yes
Identification level	Molecular species level	RT verified by standard	Yes
Polarity mode	Positive	Separation of isobaric/isomeric interferece confirmed	Yes
Type of positive (precursor)ion	[M+H]+	Model for separation prediction	No
Fragments for identification		Additional dimension/techniques	-

Fragment name
Product ion of m/z 127.01547 (theoretical value) meaning C2H8O4P (Bismethyl PO4)
Neutral loss of 126.0082 (theoretical value) meaning the loss of bismethyl PO4

Isotope correction at MS1	No	Lipid Identification Software	MS-DIAL
Isotope correction at MS2	No	Data manipulation	Smoothing, Centroiding
MS1 verified by standard	Yes	Nomenclature for intact lipid molecule	Yes
MS2 verified by standard	Yes	Nomenclature for fragment ions	N/A
Background check at MS1	Yes	Further identification remarks	-

1) LPA[M+H]+ / Lipid quantification

Quantitative	Yes	Limit of quantification	No
MS Level for quantification	MS1	Normalization to reference	No
Internal lipid standard(s) MS1		Lipid Quantification Software	MS-DIAL

Internal standard	Endogenous subclass
LPA 17:1	LPA

Type of quantification	Internal standard amount	Batch correction	No
Response correction	No	Further quantification remarks	In the paper, the original peak height are described.
Type I isotope correction	No		

Note 5. Lipidomics minimal reporting checklist for lysophospholipid profiling by using trimethylsilyl-diazomethane for GPAT enzyme assay.

Separation Workflow

Overall study design

Title of the study	Lysophospholipid profiling by using trimethylsilyl-diazomethane for GPAT enzyme assay		
Document creation date	02/07/2024	Corresponding Email	htusgawa@go.tuat.ac.jp
Principle investigator	Hiroshi Tsugawa	Is the workflow targeted or untargeted?	Targeted
Institution	Tokyo University of Agriculture and Technology	Clinical	No

Lipid extraction

Extraction method	Solid-Phase Extraction	Solid-Phase Extraction	Reverse-Phase
pH adjustment	None	Were internal standards added prior extraction?	Yes

Analytical platform

Which solvents were used	(A) acetonitrile (ACN):MeOH:H2O (1:1:3, v/v/v) and (B) ACN:IPA (1:9, v/v). Both the solvents contained 10 nM ethylenediaminetetraacetic acid and 5 mM ammonium acetate.	Mass resolution for detected ion at MS1	High resolution
Number of separation dimensions	One dimension	Resolution at m/z 200 at MS1	24949
Separation type 1	LC	Mass accuracy in ppm at MS1	0.46672
Separation mode 1 (liquid)	RP	Mass window for precursor ion isolation (in Da total isolation window)	1
Detector	Mass spectrometer	Mass resolution for detected ion at MS2	High resolution
MS type	QTOF	Resolution at m/z 200 at MS2	27299
MS vendor	SCIEX	Mass accuracy in ppm at MS2	0.56171
Ion source	ESI	Was/Were additional dimension/techniques used	No
MS Level	MS1, MS2		

Quality control

Blanks	Yes	Quality control	No
Type of Blanks	Extraction blank, Injection blank		

Method qualification and validation

Method validation	Yes	Precison	Yes
Lipid recovery	Yes	Accuracy	Yes
Dynamic quantification range	No	Guidelines followed	None
Limit of quantitation (LOQ)/Limit of detection (LOD)	No		

Reporting

Are reported raw data uploaded into repository?	Yes	Summary data	Quantification and identification data
Link to repository / ID to entry	http://prime.psc.riken.jp/menta.cgi/prime/drop_index	Raw data upload	Yes
Are metadata available?	Yes	Additional comments	The resolution and accuracy (ppm) for MS1 are those of m/z 132.9049 which were determined by the mass calibration in SCIEX OS. In addition, the resolution and accuracy for MS2 are those of m/z 185.1284, which were also determined by the mass calibration in SCIEX OS.

Sample Descriptions

GPAT enzyme assay / The products of GPAT enzyme assay / Other liquid material

Provided information	Storage time (month), Freeze-thaw cycles	Freeze-thaw cycles	0
Temperature handling original sample	Room temperature	Additives	None
Instant sample preparation	Yes	Were samples stored under inert gas?	No
Storage temperature	-80 °C	Additional preservation methods	No
Storage time (month)	1	Biobank samples	No

Lipid Class Descriptions

1) LPA[M+H]+ / Lipid identification

Lipid class	LPA	Background check at MS2	Yes
Derivatization	Trimethylsilyl (TMS)-diazomethane	Did you presume assumptions for identification?	No
MS Level for identification	MS1, MS2	Check isomer overlap	Yes
Identification level	Molecular species level	RT verified by standard	Yes
Polarity mode	Positive	Separation of isobaric/isomeric interferece confirmed	Yes
Type of positive (precursor)ion	[M+H]+	Model for separation prediction	No
Fragments for identification		Additional dimension/techniques	-

Fragment name
Product ion of m/z 127.01547 (theoretical value) meaning C2H8O4P (Bismethyl PO4)
Neutral loss of 126.0082 (theoretical value) meaning the loss of bismethyl PO4

Isotope correction at MS1	No	Lipid Identification Software	MS-DIAL
Isotope correction at MS2	No	Data manipulation	Smoothing, Centroiding
MS1 verified by standard	Yes	Nomenclature for intact lipid molecule	Yes
MS2 verified by standard	Yes	Nomenclature for fragment ions	N/A
Background check at MS1	Yes	Further identification remarks	-

1) LPA[M+H]+ / Lipid quantification

Quantitative	Yes	Limit of quantification	No
MS Level for quantification	MS1	Normalization to reference	No
Internal lipid standard(s) MS1		Lipid Quantification Software	MS-DIAL

Internal standard	Endogenous subclass
LPA 17:1	LPA

Type of quantification	Internal standard amount	Batch correction	No
Response correction	No	Further quantification remarks	-
Type I isotope correction	No		

Note 6. Lipidomics minimal reporting checklist for acyl-CoA profiling for GPAT enzyme assay.

Separation Workflow

Created by https://lipidomicstandards.org, version v2.3.2

Overall study design

Title of the study	Acyl CoA profiling for GPAT enzyme assay		
Document creation date	02/07/2024	Corresponding Email	htusgawa@go.tuat.ac.jp
Principle investigator	Hiroshi Tsugawa	Is the workflow targeted or untargeted?	Targeted
Institution	Tokyo University of Agriculture and Technology	Clinical	No

Lipid extraction

Extraction method	Solid-Phase Extraction	Solid-Phase Extraction	Reverse-Phase
pH adjustment	None	Were internal standards added prior extraction?	Yes

Analytical platform

Which solvents were used	(A) MeOH:H2O (1:4, v/v) with 0.05% NH3 and (B) MeOH:ACN (1:4, v/v) with 0.05% NH3.	Mass resolution for detected ion at MS1	High resolution
Number of separation dimensions	One dimension	Resolution at m/z 200 at MS1	36861
Separation type 1	LC	Mass accuracy in ppm at MS1	0.01
Separation mode 1 (liquid)	RP	Mass window for precursor ion isolation (in Da total isolation window)	1
Detector	Mass spectrometer	Mass resolution for detected ion at MS2	High resolution
MS type	QTOF	Resolution at m/z 200 at MS2	36861
MS vendor	Agilent	Mass accuracy in ppm at MS2	0.01
Ion source	ESI	Was/Were additional dimension/techniques used	No
MS Level	MS1, MS2		

Quality control

Blanks	Yes	Quality control	No
Type of Blanks	Extraction blank, Injection blank		

Method qualification and validation

Method validation	Yes	Precison	Yes
Lipid recovery	Yes	Accuracy	Yes
Dynamic quantification range	No	Guidelines followed	None
Limit of quantitation (LOQ)/Limit of detection (LOD)	No		

Reporting

Are reported raw data uploaded into repository?	Yes	Summary data	Quantification and identification data
Link to repository / ID to entry	http://prime.psc.riken.jp/menta.cgi/prime/drop_index	Raw data upload	Yes
Are metadata available?	Yes	Additional comments	The resolution and accuracy (ppm) for MS1 are those of m/z 118.086255which were determined by the mass calibration in Agilent MassHunter. In addition, the resolution and accuracy for the same as described in MS1 because the TOF-MS/MS check is not performed in the mass calibration process.

Sample Descriptions

GPAT enzyme assay / The products of GPAT enzyme assay / Other liquid material

Provided information	Storage time (month), Freeze-thaw cycles	Freeze-thaw cycles	0
Temperature handling original sample	Room temperature	Additives	None
Instant sample preparation	Yes	Were samples stored under inert gas?	No
Storage temperature	-80 °C	Additional preservation methods	No
Storage time (month)	1	Biobank samples	No

Lipid Class Descriptions

1) Acyl CoA[M+H]+ / Lipid identification

Lipid class	Acyl CoA	Background check at MS2	Yes
Derivatization	-	Did you presume assumptions for identification?	No
MS Level for identification	MS1, MS2	Check isomer overlap	Yes
Identification level	Molecular species level	RT verified by standard	Yes
Polarity mode	Positive	Separation of isobaric/isomeric interferece confirmed	Yes
Type of positive (precursor)ion	[M+H]+	Model for separation prediction	No
Fragments for identification		Additional dimension/techniques	-

Fragment name
Neutral loss of 506.9957461 (theoretical value) meaning the loss of adenosine triphosphate (C10H16N5O13P3)

Isotope correction at MS1	No	Lipid Identification Software	MS-DIAL
Isotope correction at MS2	No	Data manipulation	Smoothing, Centroiding
MS1 verified by standard	Yes	Nomenclature for intact lipid molecule	Yes
MS2 verified by standard	Yes	Nomenclature for fragment ions	N/A
Background check at MS1	Yes	Further identification remarks	-

1) Acyl CoA[M+H]+ / Lipid quantification

Quantitative	Yes	Limit of quantification	No
MS Level for quantification	MS1	Normalization to reference	No
Internal lipid standard(s) MS1		Lipid Quantification Software	MS-DIAL

Internal standard	Endogenous subclass
AcylCoA 17:1	AcylCoA

Type of quantification	Internal standard amount	Batch correction	No
Response correction	No	Further quantification remarks	-
Type I isotope correction	No		
