# Supplementary material for: MS-DIAL 5 multimodal mass spectrometry data mining unveils lipidome complexities
Source: Nat Commun. 2024 Nov 28;15:9903. doi: 10.1038/s41467-024-54137-w (PMC11605090; doi:10.1038/s41467-024-54137-w)
Supplement: Supplementary file 2 — Reporting Summary [file 41467_2024_54137_MOESM2_ESM.pdf]

nature portfolio

Corresponding author(s): Hiroshi Tsugawa, Takeshi Harayama, Makoto Arita

Last updated by author(s): Oct 16, 2024

Reporting Summary

Nature Portfolio wishes to improve the reproducibility of the work that we publish. This form provides structure for consistency and transparency in reporting. For further information on Nature Portfolio policies, see our Editorial Policies and the Editorial Policy Checklist.

Statistics

For all statistical analyses, confirm that the following items are present in the figure legend, table legend, main text, or Methods section.

n/a	Confirmed	
☐	☒	The exact sample size (*n*) for each experimental group/condition, given as a discrete number and unit of measurement
☐	☒	A statement on whether measurements were taken from distinct samples or whether the same sample was measured repeatedly
☐	☒	The statistical test(s) used AND whether they are one- or two-sided<br>*Only common tests should be described solely by name; describe more complex techniques in the Methods section.*
☒	☐	A description of all covariates tested
☐	☒	A description of any assumptions or corrections, such as tests of normality and adjustment for multiple comparisons
☐	☒	A full description of the statistical parameters including central tendency (e.g. means) or other basic estimates (e.g. regression coefficient) AND variation (e.g. standard deviation) or associated estimates of uncertainty (e.g. confidence intervals)
☐	☒	For null hypothesis testing, the test statistic (e.g. F, t, r) with confidence intervals, effect sizes, degrees of freedom and P value noted<br>*Give P values as exact values whenever suitable.*
☒	☐	For Bayesian analysis, information on the choice of priors and Markov chain Monte Carlo settings
☒	☐	For hierarchical and complex designs, identification of the appropriate level for tests and full reporting of outcomes
☒	☐	Estimates of effect sizes (e.g. Cohen's d, Pearson's r), indicating how they were calculated

Our web collection on statistics for biologists contains articles on many of the points above.

Software and code

Policy information about availability of computer code

Data collection	MS-DIAL 5 was used for data analysis. The MS-DIAL source code is available at https://github.com/systemsomicslab/MsdialWorkbench.
Data analysis	The MS-DIAL tutorial and the demonstration data are available at https://systemsomicslab.github.io/msdial5tutorial/ and https://zenodo.org/communities/msdial, respectively. The parameters used were described in Supplementary Table S4 and S10.

For manuscripts utilizing custom algorithms or software that are central to the research but not yet described in published literature, software must be made available to editors and reviewers. We strongly encourage code deposition in a community repository (e.g. GitHub). See the Nature Portfolio guidelines for submitting code & software for further information.

Data

Policy information about availability of data

All manuscripts must include a data availability statement. This statement should provide the following information, where applicable:
- Accession codes, unique identifiers, or web links for publicly available datasets
- A description of any restrictions on data availability
- For clinical datasets or third party data, please ensure that the statement adheres to our policy

The spectral data of the authentic standards are available in MSP format files on the RIKEN DROPMet website (http://prime.psc.riken.jp/menta.cgi/prime/drop_index) under the index number DM0054, and the details are described in the readme file. All raw LC-MS data were available with the same index number as the RIKEN DROPMet. Spatial- and untargeted lipidomics data for eye tissues in mice are available under the index number DM0048. Source Data are provided with this paper. The MS-DIAL source code is available at https://github.com/systemsomicslab/MsdialWorkbench. The MS-DIAL tutorial and the demonstration data are

available at https://systemsomicslab.github.io/msdial5tutorial/ and https://zenodo.org/communities/msdial, respectively.

Human research participants

Policy information about studies involving human research participants and Sex and Gender in Research.

Reporting on sex and gender	not applicable
Population characteristics	not applicable
Recruitment	not applicable
Ethics oversight	not applicable

Note that full information on the approval of the study protocol must also be provided in the manuscript.

Field-specific reporting

Please select the one below that is the best fit for your research. If you are not sure, read the appropriate sections before making your selection.

[x] Life sciences [ ] Behavioural & social sciences [ ] Ecological, evolutionary & environmental sciences

For a reference copy of the document with all sections, see nature.com/documents/nr-reporting-summary-flat.pdf

Life sciences study design

All studies must disclose on these points even when the disclosure is negative.

Sample size	N=4 for mouse and HeLa cells experiments. No statistical method was used to predetermine sample size.
Data exclusions	No data were excluded from the analyses.
Replication	N=4 biological replicates were used for the statistical analyses.
Randomization	The biological samples in MS analysis were randomized.
Blinding	The background was blinded when the samples were analyzed by LC-MS.

Reporting for specific materials, systems and methods

We require information from authors about some types of materials, experimental systems and methods used in many studies. Here, indicate whether each material, system or method listed is relevant to your study. If you are not sure if a list item applies to your research, read the appropriate section before selecting a response.

Materials & experimental systems

n/a	Involved in the study	
[x]	[ ]	Antibodies
[ ]	[x]	Eukaryotic cell lines
[x]	[ ]	Palaeontology and archaeology
[ ]	[x]	Animals and other organisms
[x]	[ ]	Clinical data
[x]	[ ]	Dual use research of concern

Methods

n/a	Involved in the study	
[x]	[ ]	ChIP-seq
[x]	[ ]	Flow cytometry
[x]	[ ]	MRI-based neuroimaging

Eukaryotic cell lines

Policy information about cell lines and Sex and Gender in Research

Cell line source(s)	HeLa
Authentication	Authentication is performed by RIKEN BRC.
Mycoplasma contamination	HeLa cell lines tested negative for mycoplasma contamination
Commonly misidentified lines (See ICLAC register)	not applicable

Animals and other research organisms

Policy information about studies involving animals; ARRIVE guidelines recommended for reporting animal research, and Sex and Gender in Research

Laboratory animals	C57B6J/J
Wild animals	N/A
Reporting on sex	Male mice were used
Field-collected samples	N/A
Ethics oversight	The animal experiments were performed in accordance with the ethical protocol approved by the Tokyo University of Agriculture and Technology (R5-50).

Note that full information on the approval of the study protocol must also be provided in the manuscript.
