# Supplementary material for: MS-DIAL 5 multimodal mass spectrometry data mining unveils lipidome complexities
Source: Nat Commun. 2024 Nov 28;15:9903. doi: 10.1038/s41467-024-54137-w (PMC11605090; doi:10.1038/s41467-024-54137-w)
Supplement: Supplementary file 3 — Description of Additional Supplementary Files [file 41467_2024_54137_MOESM3_ESM.pdf]

Description of Additional Supplementary Files

Supplementary Data 1. Detail of 953 authentic standards.

Supplementary Data 2. Detail of lipid authentic standards or biologically created metabolites.

Supplementary Data 3. Details of LightSPLASH and the lipid description that can be characterized in this study.

Supplementary Data 4. MS-DIAL parameters used for Light SPLASH- and Ultimate SPLASH/in-house standards spectral data.

Supplementary Data 5. Details of ultimate splash, in-house standards, and lipid description are characterized in this study.

Supplementary Data 6. Details of MS-DIAL evaluations using Light SPLASH (13 standards) and Ultimate SPLASH and in-house standard mixture (91 standards).

Supplementary Data 7. MS-DIAL evaluation to characterize the lipid isomers of PC 16:0/18:1(9), PC 16:0/18:1(11), and PC 18:1(9)/16:0 in the mixture of co-eluted authentic standards, mouse brain, and NIST SRM 1950 plasma.

Supplementary Data 8. Annotation results using CID-MS/MS and EAD-MS/MS to profile mouse eye lipidome.

Supplementary Data 9. Database of species/tissue-specific *m/z* and collision-cross section values of lipids.

Supplementary Data 10. MS-DIAL parameters used in the untargeted lipidomics from public data.

Supplementary Data 11. Base sequence and primer details in GPAT1 enzyme assay.
