# Supplementary figures and images for: MS-DIAL 5 multimodal mass spectrometry data mining unveils lipidome complexities
Source: Nat Commun. 2024 Nov 28;15:9903. doi: 10.1038/s41467-024-54137-w (PMC11605090; doi:10.1038/s41467-024-54137-w)

## Slide 1
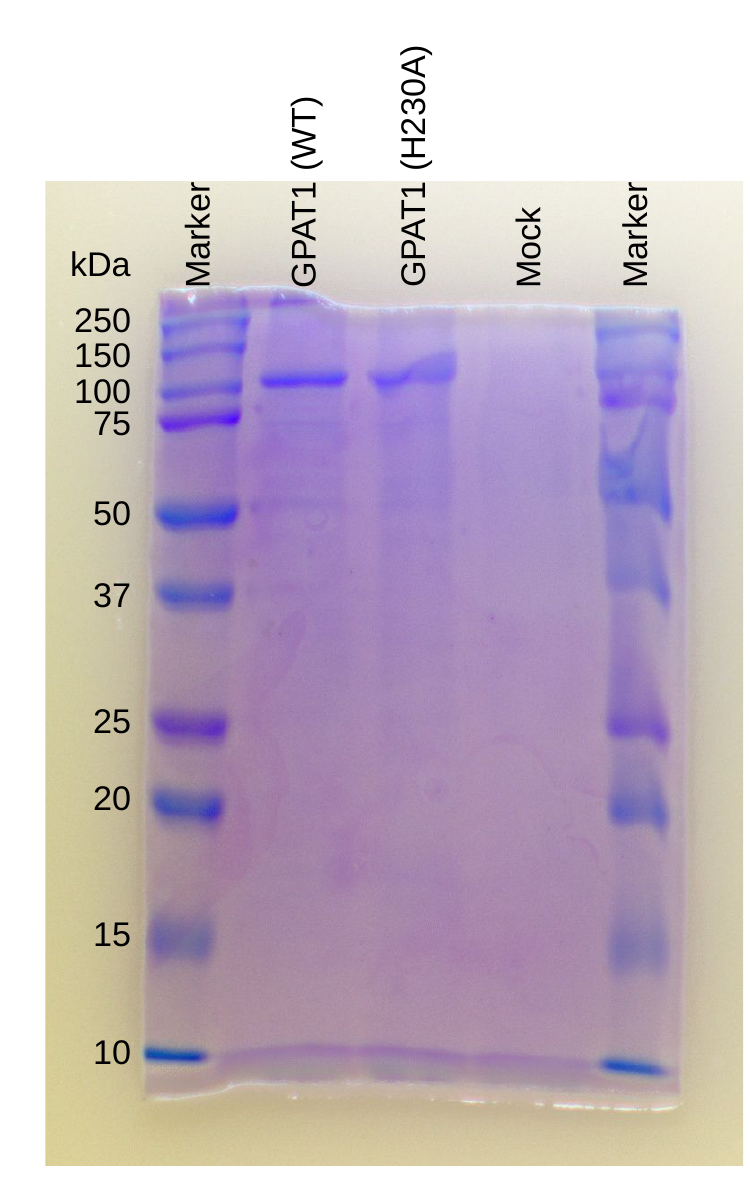

GPAT1 (H230A)
GPAT1 (WT)
Marker
Marker
Mock
kDa
250
150
100
75
50
37
25
20
15
10

Supplement: Supplementary file 16 — Source data [file 41467_2024_54137_MOESM16_ESM.zip › sourcedata/figureS12/figureS12 original image.pptx]
